# DNA damage repair-related methylated genes RRM2 and GAPDH are prognostic biomarkers associated with immunotherapy for lung adenocarcinoma

**DOI:** 10.1590/1678-4685-GMB-2024-0138

**Published:** 2025-05-09

**Authors:** Xinru Mao, Shaban Eljali Saad, Nung Kion Lee, Isabel Lim Fong

**Affiliations:** 1 Universiti Malaysia Sarawak (UNIMAS), Faculty of Medicine and Health Sciences, Department of Paraclinical Sciences, Kota Samarahan, Malaysia.; 2 University of Tripoli, Faculty of Pharmacy, Tripoli, Libya.; 3 Universiti Malaysia Sarawak (UNIMAS), Faculty of Computer Science and Information Technology, Kota Samarahan, Malaysia.

**Keywords:** Lung adenocarcinoma, DNA methylation, DNA damage repair, prognosis, immunotherapy

## Abstract

Research has highlighted the significant role of methylated genes associated with DNA damage repair in pathogenesis of Lung adenocarcinoma (LUAD). However, the potential of DNA damage repair-related gene (DDRG) methylation as a prognostic biomarker remains underexplored. This study aimed to assess the prognostic value of methylated DDRGs in LUAD. Analysis of the TCGA-LUAD dataset revealed differentially expressed genes (DEGs) and differentially methylated genes (DE-MGs), from which methylated DE-DDRGs were identified. An independent prognostic risk model was constructed based on these methylated DE-DDRGs by integrating risk scores with clinical features. Additionally, the study examined responses to immunotherapy. Results indicated that CLU exhibited hypermethylation and elevated expression in LUAD tissues, while eight other genes (BUB1B, SHCBP1, RRM2, RPL39L, TRIP13, GAPDH, ENO1, and CENPM) showed high expression and hypomethylation. Among these, RRM2 and GAPDH were significantly linked to poorer overall survival. Furthermore, single-sample gene set enrichment analysis (ssGSEA) revealed that patients with LUAD in the high-risk group had lower immune scores and less immune cell infiltration. TIDE analysis suggested that patients in the low-risk group may exhibit greater sensitivity to immune checkpoint inhibitor therapy. In conclusion, RRM2 and GAPDH represent promising prognostic and immunotherapeutic biomarkers, offering new avenues for LUAD treatment strategies.

## Introduction

Lung cancer remains the leading cause of cancer-related deaths, presenting a major global public health challenge (Sung et al., 2021). Prognosis and 5-year survival rates for lung cancer are primarily based on the tumor (T), lymph node (N), and distant metastasis (M) staging system, with long-term prognosis worsening as the disease advances (Goldstraw et al., 2016; Woodard et al., 2016; Hwang et al., 2020). Non-small cell lung cancer (NSCLC) accounts for approximately 80% of primary lung cancers, with lung adenocarcinoma (LUAD) being the most common NSCLC subtype (Testa et al., 2018; Chen, H et al., 2019). Early-stage LUAD is typically managed with surgery, radiotherapy, and chemotherapy. However, relapse is common even after complete surgical resection. Although advances in targeted therapy and immunotherapy have improved outcomes for metastatic LUAD (Chaft et al., 2021), the long-term prognosis, risk of metastasis, and recurrence rates remain suboptimal (Dziedzic et al., 2016). Thus, identifying novel biomarkers and therapeutic targets is critical to enhancing the diagnosis and overall prognosis of LUAD.

DNA methylation is an epigenetic mechanism that regulates gene expression and facilitates stable gene silencing (Moore et al., 2013). Aberrant DNA methylation is a key regulatory mechanism in the early stages of carcinogenesis (Sharma et al., 2010). Given that DNA methylation is reversible, it presents a promising therapeutic target (Kulis and Esteller, 2010). Extensive research has explored DNA methylation as a diagnostic biomarker for cancer detection (Tsou et al., 2002; Yang et al., 2019; Cai et al., 2020). Furthermore, DNA methylation patterns can predict clinical outcomes and treatment responses. Notably, DNA methylation rates differ between lung cancer histological subtypes, with genes such as ANK1, APC, CCND2, CDH13, KCNH5, RARβ, and RUNX3 exhibiting significantly higher methylation in LUAD compared to other subtypes (Toyooka et al., 2003; Hawes et al., 2010; Tessema et al., 2017). In LUAD, reduced methylation of FAM83A leads to gene upregulation, which correlates with poor prognosis (Yu et al., 2020). Additionally, a nine-gene methylation signature (including C20orf56, BTG2, C13orf16, DNASE1L1, ZDHHC3, FHDC1, ARF6, ITGB3, and ICAM4) can predict survival and recurrence (Wang et al., 2019). Therefore, DNA methylation studies hold promise for identifying predictive biomarkers for treatment and offering personalized therapeutic strategies.

DNA damage plays a pivotal role in the tumorigenesis of various cancers, including LUAD (Dizdaroglu 2015; Jeggo et al., 2016; Burgess et al., 2020; Papalouka et al., 2023; Tufail 2023), primarily by inducing gene mutations that drive tumor development (Burcher *et al.,* 2021). However, the occurrence of DNA damage also triggers DNA repair pathways that maintain genetic stability. DNA repair genes are essential for preserving normal cellular division and function by identifying and repairing DNA damage. Five major DNA repair pathways - base excision repair (BER), nucleotide excision repair (NER), mismatch repair (MMR), homologous recombination (HR), and non-homologous DNA end joining (NHEJ) - play key roles in maintaining genomic integrity, supplemented by two specialized pathways: direct chemical reversal and interstrand crosslink repair (Chatterjee and Walker, 2017; Tufail 2023). Evidence links DNA repair pathways to tumor progression and therapeutic responses across multiple cancers (Gavande et al., 2016; Tessoulin et al., 2018; Lima et al., 2019; Mei et al., 2019; Groelly et al., 2023). In lung cancer, DNA damage signaling and repair pathways significantly influence prognosis and treatment outcomes (Burgess *et al.,* 2020; Mao et al., 2024). Moreover, DNA repair gene methylation can serve as a predictive marker for chemotherapy and targeted therapy responses, particularly with PARP inhibitors (Bartkova et al., 2005; Asakawa et al., 2010; Kang et al., 2012; Gkotzamanidou et al., 2014; Gao et al., 2016). For instance, PARP1 inhibitors are approved for treating DNA repair-deficient tumors such as ovarian and breast cancers (Ray Chaudhuri et al., 2017).

Alterations in DNA repair genes are diverse in LUAD and have potential as biomarkers reflecting disease status. However, the prognostic relevance of aberrantly methylated DNA damage-repair (DDR) genes remains underexplored. This study investigates the methylation status and expression levels of DDR genes, particularly RRM2 and GAPDH, to evaluate their prognostic significance in LUAD. A risk prediction model was constructed and validated using multiple datasets (GSE31210 and GSE68465), providing a novel approach to biomarker identification in LUAD and laying the groundwork for personalized treatment strategies. Additionally, the role of these genes in the tumor immune microenvironment was explored, offering deeper insights into the complex biology of LUAD.

## Material and Methods

### LUAD-related data source

The RNA sequencing (RNA-seq) data (FPKM) and DNA methylation data used in this study were sourced from the TCGA (https://portal.gdc.cancer.gov/) and UCSC (https://xena.ucsc.edu/public) databases, respectively. After performing sample pairwise checks, RNA-seq and DNA methylation data were obtained for 18 normal and 18 tumor samples. The GSE31210 dataset was downloaded from the Gene Expression Omnibus (GEO) database (http://www.ncbi.nlm.nih.gov/geo/), while the GSE68465 dataset was retrieved from GPL96 (Affymetrix Human Genome U133A Array, [HG-U133A]). The GSE31210 dataset included 226 LUAD samples with complete survival time and status, while the GSE68465 dataset contained 442 LUAD cases with survival data, primarily used for validation of the constructed prognostic signature (Okayama et al., 2012; Yamauchi et al., 2012).

### Collection of DNA damage-repair-related genes (DDRGs)

A total of 243 DNA damage-related genes (Table S1) and 387 DNA repair-related genes (Table S2) were downloaded from the CancerSEA dataset (http://biocc.hrbmu.edu.cn/CancerSEA/). Additionally, 295 DNA damage and repair-related genes were retrieved from MSigDB (https://www.gsea-msigdb.org/gsea/msigdb) (Table S3). A de-duplication merge analysis was conducted across these three gene lists, resulting in a total of 696 DNA damage repair-related genes (DDRGs; Table S4) for further analysis.

### Differential expression analysis

**Figure 1 -:**
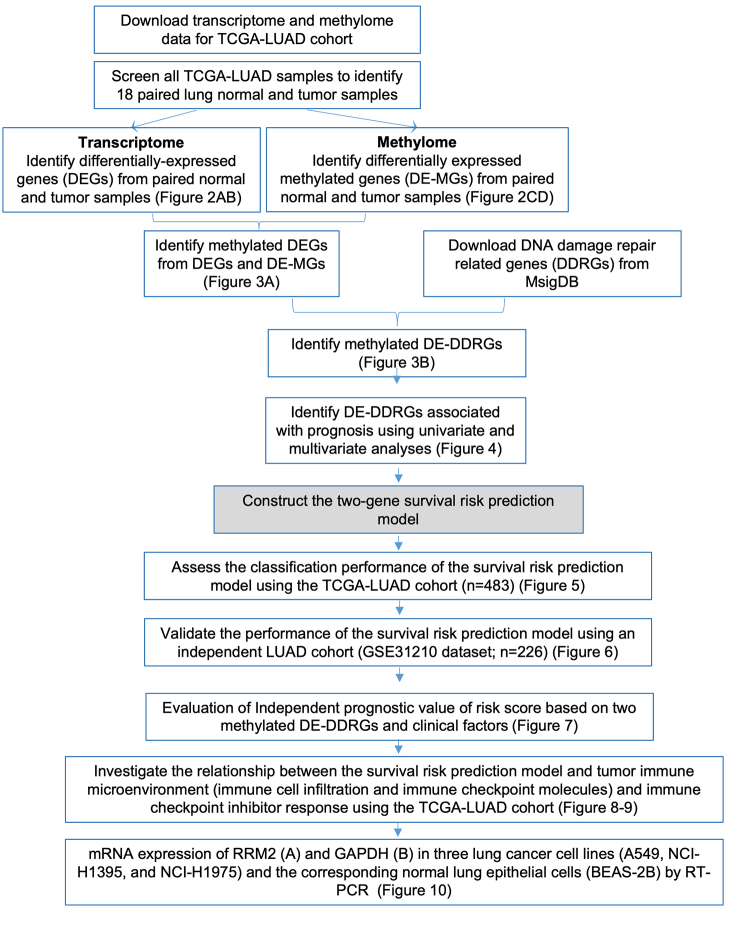
Flowchart of the study design.

The study design is depicted in Figure 1. Differentially expressed genes (DEGs) between normal and tumor samples were identified using the R package limma (v 3.44.3) (Ritchie et al., 2015), applying the criteria |log_2_ fold change (FC)| > 0.5 and adjusted P < 0.05. Differentially methylated CpG sites (DE-MCS) between the 18 paired LUAD and normal samples were identified using the R package ChAMP, with a significance threshold of |deltaBeta| > 0.2 and P < 0.05. Genes annotated by DE-MCS were defined as differentially methylated genes (DE-MGs) for further analysis. DE-MGs with deltaBeta > 0.2 and P < 0.05 were considered hypermethylated genes, while those with deltaBeta < -0.2 and P < 0.05 were classified as hypomethylated genes.

### Overlap analysis

Overlap analysis was performed to identify common factors across gene lists. Upregulated DEGs with hypomethylated genes were subjected to overlap analysis, with the shared genes termed hypomethylated upregulated genes. Similarly, downregulated DEGs with hypermethylated genes were defined as hypermethylated downregulated genes. The union of these two gene lists formed the set of methylated DEGs, which were then intersected with the previously identified DDRGs to identify methylated DE-DDRGs.

### The construction, evaluation, and validation of risk model

To evaluate whether DDR-related MeDEGs (methylated differentially expressed genes) were associated with the survival of patients with LUAD, the expression data (FPKM) of DDR-related MeDEGs were extracted from the TCGA-LUAD dataset and merged with clinical data, including survival follow-up information. This integration resulted in clinical expression data for DDR-related MeDEGs in TCGA-LUAD samples. These clinical expression data were used as the training set to construct the prognostic risk model, while two independent datasets, GSE31210 and GSE68465, served as validation sets to verify the model’s predictive accuracy. Univariate Cox regression analysis was performed on the DDR-related MeDEGs in the TCGA-LUAD training set, with genes exhibiting a hazard ratio (HR) ≠ 1 and P < 0.05 being identified as prognostic candidates (7 genes). These seven genes were further subjected to multivariate Cox regression analysis. The stepwise regression function (step()) was applied to optimize the model, using the Akaike Information Criterion (AIC) as the selection criterion to add or remove variables. The objective was to identify the most optimal regression model with the lowest AIC value.

Genes with P < 0.05 were considered statistically significant in terms of prognostic value. Risk scores were subsequently calculated for each sample in both the training set and the independent external validation sets, based on the expression levels and regression coefficients (coef, derived from the multivariate Cox analysis) of the prognostic genes. The risk score for each sample was determined using the following formula:

$$risk\;score=({coef}_{{gene}_{1}}\times {expression\;value\;of\;gene}_{1})+({coef}_{{gene}_{2}}\times {expression\;value\;of\;gene}_{2})+\cdots ({coef}_{{gene}_{n}}\times {expression\;value\;of\;gene}_{n})$$ (1)

LUAD samples from each dataset were stratified into high- and low-risk groups based on the median risk score value. Samples with risk scores above the median were classified into the high-risk group, while those with scores below the median were assigned to the low-risk group. The median risk score value was computed using the MEDIAN function. Overall survival (OS) differences between high- and low-risk groups in both the training and independent external validation sets were evaluated using Kaplan-Meier (K-M) analysis and the log-rank test, with a P value < 0.05 considered statistically significant. Time-dependent (1-, 2-, and 3-year) ROC curves were generated based on the risk scores, and the prognostic accuracy of the model was assessed by calculating the area under the curve (AUC), where AUC > 0.6 was considered indicative of good predictive performance.

### Independent prognostic analysis of the risk score

Univariate and multivariate Cox regression analyses were conducted to assess whether the risk score remained an independent prognostic factor for LUAD when adjusted for clinical parameters such as age, gender, pathological stage, tumor (T) stage, node (N) stage, and metastasis (M) stage (P < 0.05). Variables with P < 0.05 from multivariate Cox analysis were considered independent prognostic factors for patients with LUAD.

### Construction and evaluation of the nomogram model

All independent prognostic factors were integrated into a nomogram(Iasonos et al., 2008) to predict 1-, 2-, and 3-year OS for patients with LUAD in the TCGA-LUAD dataset. The performance of the nomogram was assessed using Harrell’s concordance index (C-index), and its calibration was evaluated by plotting a calibration curve to compare predicted survival probabilities with observed outcomes.

### ESTIMATE and single sample gene set enrichment analysis (ssGSEA) of the LUAD samples

The Estimation of STromal and Immune cells in MAlignant Tumor tissues using Expression data (ESTIMATE) algorithm (Yoshihara et al., 2013) was applied to predict tumor purity and the infiltration of stromal and immune cells in LUAD samples. The immune score (representing immune cell infiltration), stromal score (indicating stromal content), and ESTIMATE score (reflecting tumor purity) were calculated for each sample. Higher scores corresponded to a greater proportion of the respective components within the tumor immune microenvironment.

To further analyze immune cell infiltration, ssGSEA(Rooney, Shukla *et al.,* 2015 was performed to calculate the enrichment levels of 24 immune-associated gene sets for each LUAD sample using the gsva R package (v 1.36.3) (Wang et al., 2024). Significant differences in immune cell abundances were determined using analysis of variance (ANOVA).

### TIDE and SubMap algorithms

The tumor immune dysfunction and exclusion (TIDE; http://tide.dfci.harvard.edu/) algorithm (Jiang et al., 2018) and subclass mapping (SubMap; https://cloud.genepattern.org/gp) (Hoshida et al., 2007) were utilized to predict the clinical response to PD-1 and CTLA4 immune checkpoint inhibitors in both low- and high-risk score groups (P < 0.05). 

### Cell culture and reagents

The normal human lung epithelial cell line BEAS-2B and three human lung cancer cell lines (A549, NCI-H1975, and NCI-H1395) were obtained from the American Type Culture Collection (Manassas, VA, USA). All cells were cultured in six-well plates with RPMI-1640 medium supplemented with 10% fetal calf serum, 5 mmol/L L-glutamine, 5 mmol/L non-essential amino acids, 100 U/mL penicillin, and 100 U/mL streptomycin (Invitrogen, Carlsbad, CA, USA), at 37 °C in a humidified 5% CO_2_ atmosphere.

### Validation of key gene expression levels

To clarify the expression of prognostic genes in both disease and normal samples, the Wilcoxon rank-sum test (P < 0.05) was employed to analyze the expression of two prognostic genes (RRM2 and GAPDH) across disease and normal samples in both training and validation sets. Based on this, the expression levels of RRM2 and GAPDH mRNAs were assessed in three human lung cancer cell lines and a normal control group. Cells were lysed using TRIzol Reagent (Life Technologies-Invitrogen, Carlsbad, CA, USA), and total RNA was extracted following the manufacturer’s instructions. RNA concentration and purity were quantified using a NanoDrop 2000FC-3100 nucleic acid protein quantifier (Thermo Fisher Scientific, Waltham, MA, USA). The extracted RNA was reverse-transcribed into cDNA using the SureScript-First-strand-cDNA-synthesis-kit (Genecopoeia, Guangzhou, China) prior to RT-PCR. The RT-PCR reaction consisted of 4 µl of the reverse transcription product, 2 µl of 5× BlazeTaq qPCR Mix (Genecopoeia, Guangzhou, China), 0.5 µl of each forward and reverse primer, and 3 µl of nuclease-free water. PCR was performed on a BIO-RAD CFX96 Touch TM PCR detection system (Bio-Rad Laboratories, Inc., USA) under the following conditions: initial denaturation at 95 °C for 30 s, followed by 40 cycles of 95 °C for 10 s, 60 °C for 20 s, and 72 °C for 30 s. The primers used were: RRM2 sense, 5′-CGGAGCCGAAAACTAAAGCA-3′ and anti-sense, 5′-ATGGGGAAGATGACAAAGCG-3′; GAPDH sense, 5′-CCTTCCGTGTTCCTACCCC-3′ and anti-sense, 5′-GCCCAAGATGCCCTTCAGT-3′; β-actin sense, 5′-TCCCTGGAGAAGAGCTATGA-3′ and anti-sense, 5′-AGGAAGGAAGGCTGGAAAAG-3′. All primers were synthesized by Tsingke (Tsingke Biotechnology Co., Ltd., Beijing, China). β-actin was used as an internal control, and the relative expression of RRM2 and GAPDH was calculated using the 2^-ΔΔCt^ method (Livak and Schmittgen, 2001). The experiment was performed in triplicate. Statistical differences between cell lines for RRM2 and GAPDH expression were analyzed using one-way ANOVA, with significance indicated as * for P < 0.05 and ** for P < 0.01, determined *via* GraphPad Prism V9 (GraphPad Software, La Jolla, CA, USA). 

### Statistical analysis

A heatmap illustrating the expression intensity of DEGs, DE-MCS, and prognostic genes was generated using the R package pheatmap (v 0.7.7) (https://CRAN.R-project.org/package=pheatmap). ROC curves were plotted with the R package pROC, and the nomogram was constructed using the R package rms. The ESTIMATE algorithm was applied *via* the R package ESTIMATE. Subgroup differential analyses were performed using the Wilcoxon rank-sum test and the Kruskal-Wallis test. Statistical significance was set at P < 0.05 for all analyses.

## Results

### A total of 5,425 differentially expressed methylated genes (DE-MGs) were recognized

**Figure 2 -:**
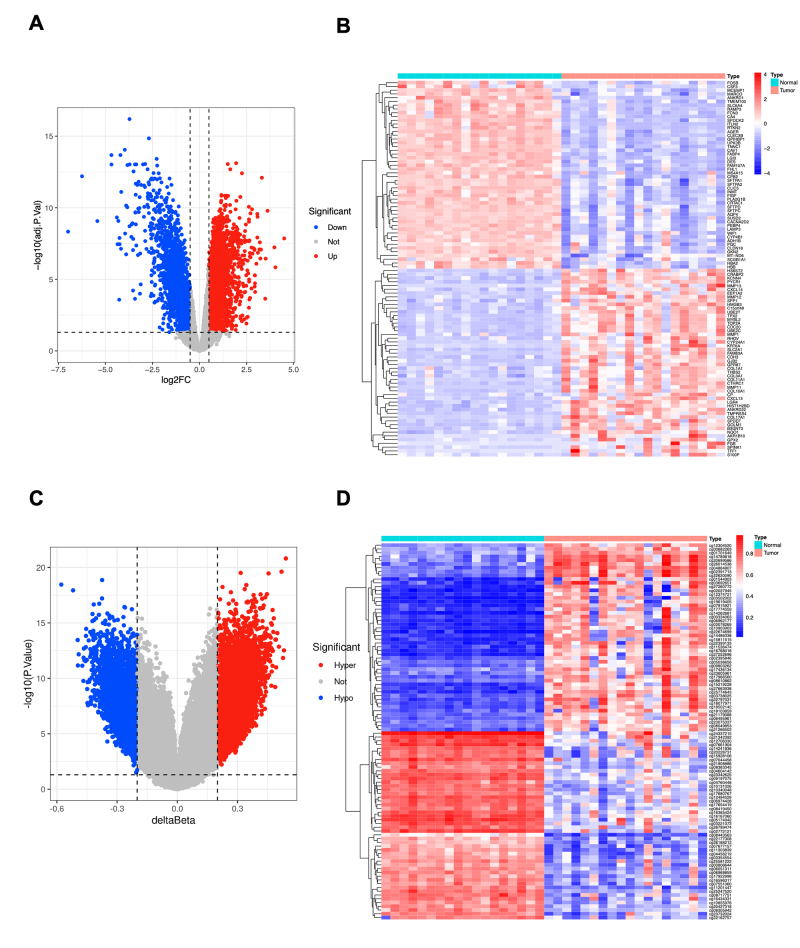
DEGs and DE-MCS in lung adenocarcinoma (LUAD). **A, C.** Volcano plots displaying the distribution of DEGs (A) and DE-MCS (C) in LUAD samples compared to paired normal tissue samples (n = 18). **B, D.** Heatmaps illustrating the gene expression pattern of the top 50 up-regulated and down-regulated genes (B) and the DNA methylation pattern of the top 50 hypermethylated and hypomethylated CpG sites (D) in LUAD samples compared to paired normal tissue samples (n = 18).

RNA expression profiles from 18 paired samples, integrating TCGA-RNA-seq and UCSC-methylation data, were used for differential expression analysis, revealing 1,778 DEGs between normal and LUAD samples. Compared to the normal group, 853 DEGs were up-regulated in the LUAD group, while 925 were down-regulated (Figure 2 A  and 2B; Table S5).

UCSC-methylation data from the same 18 paired samples were employed for DE-MCS analysis. Using the ChAMP package, a total of 20,249 DE-MCSs were identified, with 12,977 hypermethylated and 7,272 hypomethylated sites (Figure 2 C  and 2D). Subsequent annotation of these DE-MCSs yielded 5,425 DE-MGs, including 2,716 hypermethylated genes (Table S6) and 2,709 hypomethylated genes (Table S7).

### CLU, BUB1B, SHCBP1, RRM2, RPL39L, TRIP13, GAPDH, ENO1, and CENPM were identified as methylated DE-DDRGs

**Figure 3 -:**
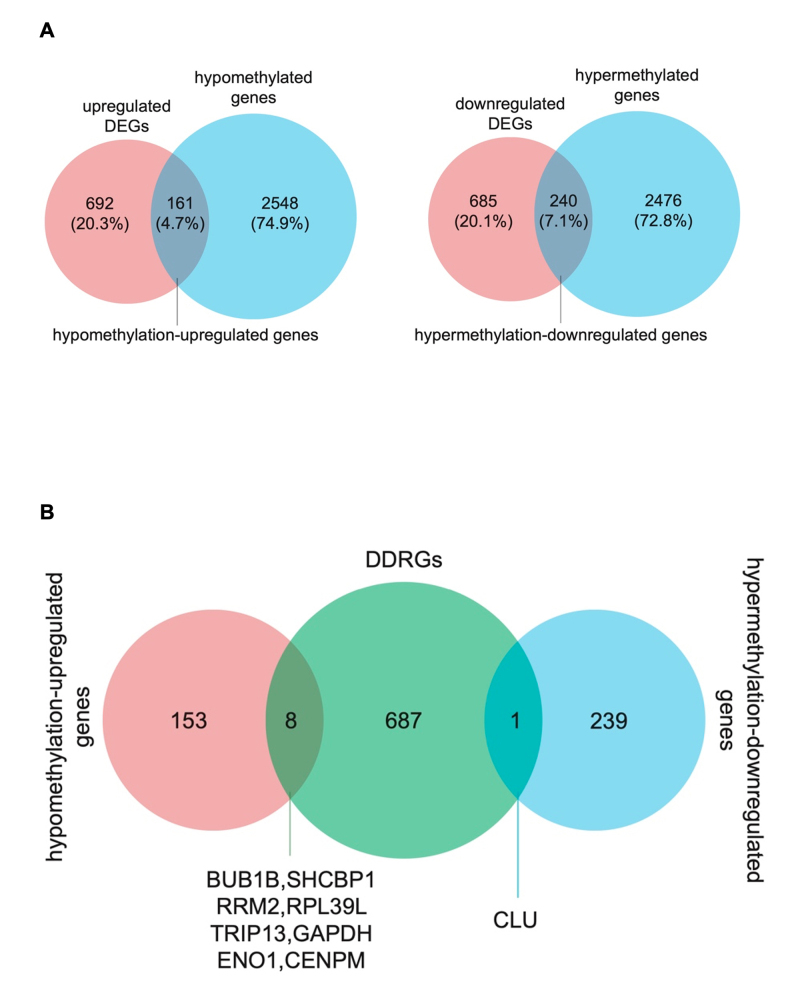
Methylated differentially-expressed DDR genes (methylated DE-DDRGs) in lung adenocarcinoma (LUAD). **A-B.** Venn diagrams showing the intersection between up-regulated, hypomethylated genes and down-regulated, hypermethylated genes (A), and the overlap between methylated DEGs and DDRGs, defining the methylated DE-DDRGs (B).

Intersection analysis of DEGs and DE-MGs was performed to identify genes exhibiting hypomethylated high expression and hypermethylated low expression patterns in LUAD. This analysis identified 161 genes by overlapping the 2,709 hypomethylated and 853 highly expressed genes, and 240 hypermethylated down-regulated genes by overlapping 2,716 hypermethylated and 925 low-expressed genes (Figure 3 A ). These genes, referred to as methylated DEGs, are listed in Table 1. The association between DNA methylation and DDR systems is supported by increasing evidence (Jacinto and Esteller, 2007). Notably, 696 DDRGs were retrieved from the CancerSEA and MSigDB databases and subjected to intersection analysis with the identified methylated DEGs. As shown in Figure 3 B , nine genes were common to both sets: CLU, which was hypermethylated and low-expressed, and BUB1B, SHCBP1, RRM2, RPL39L, TRIP13, GAPDH, ENO1, and CENPM, which were hypomethylated and highly expressed. These nine genes were classified as methylated DE-DDRGs.

**Table 1 -:** The hypomethylated-high expression and hypermethylated-low expression genes in TCGA database*.*

Hypomethylated-high expression genes	Hypermethylated-low expression genes
PYCR1, ZC3HAV1L, STK39, PPAP2C, HN1L, LGR4, KCNN4, SLC35F2, TMEM177, SLC2A1, UBFD1, PC, ANKRD22, SLC39A11, PROM2, RASAL1, EFNA4, FBXO32, ST14, TMEM184A, BUB1B, EPCAM, LPGAT1, OCIAD2, TAF1D, B3GNT3, ENTPD7, CLDN12, PFKP, FHL2, KDELR3, KIF26B, CEP55, C1orf106, STX1A, ZNF217, FAM83A, TMPRSS4, SHCBP1, XDH, CTHRC1, GPR87, EPN3, FAT1, ALG1L, SYNJ2, CHD1L, SRD5A1, GALNT7, VARS, NQO1, GJB2, CHMP4C, RRM2, ABCC3, DSP, PLEKHA6, MYO1E, DLG5, ENAH, HMGA1, PTPRH, STEAP3, AP1S1, KRT80, HN1, BAIAP2L1, GRAMD3, SULF1, BZW2, PSMG3, DBNDD1, ATIC, PKP3, RHOV, SRD5A3, TBL1XR1, TNS4, P2RY6, HKDC1, ADAM28, GPR115, ARNTL2, COL5A1, GMDS, RPL39L, RAMP1, KCNQ3, TRIP13, COL17A1, TMEM156, GAPDH, ADAM12, AIM2, SLC12A7, SERPINB5, ADAMTS12, MYEOV, STEAP2, NFE2L3, MRPL15, EFNA5, EMB, MMP1, PPM1H, FAM83H, BCL9, CPNE7, PERP, FGD6, ADAM9, WISP1, FAM83B, ENO1, ITGA2, CP, POSTN, SYT7, CFB, MFI2, BAIAP2L2, KPNA7, GJB3, KRT6A, TSKU, SPOCK1, MMP13, RPL22L1, CENPM, MUC13, SYT12, KRT15, LYPD1, DUSP4, MET, SPTB, ALDH3B2, EIF4EBP1, HHIPL2, IL23A, PRRX2, GALNT6, FNDC1, HTR3A, SLC7A11, SORL1, PHLDA2, SLC16A3, LY6D, SLAMF7, TCN1, TRIM31, TFF1, IVL, AGR2, LAMC2, TMEM45A, SPRR2D, TSPAN8, CLDN1, TRIM29	ACVRL1, STX11, KANK3, AGER, PDLIM2, S1PR1, WNT3A, CD36, LIN7A, CRTAC1, SPOCK2, EDNRB, PRX, GRK5, EPAS1, TEK, ADAMTS8, NCKAP5, EMCN, CRYAB, RAMP3, ESAM, JAM2, TAL1, TMEM88, PHACTR1, SCUBE1, CLDN5, GLIPR2, DES, TIE1, NPR1, CLEC1A, ADPRH, NECAB1, GDF10, LDB2, KCNK3, CASKIN2, ARHGEF15, CLDN18, LHFP, CNTN6, ST8SIA6, DUOX1, FEZ1, NES, HYAL2, DUOXA1, GRASP, ITIH5, COX7A1, FGR, F10, SOX7, NOTCH4, STARD13, DENND3, ADRB1, MFAP4, FAM150B, CYYR1, DACH1, DLC1, SOX17, GRIA1, C5orf38, PKNOX2, SCN4B, TCF21, RHOJ, SLC19A3, NHSL1, PTPRB, CALCRL, WIF1, TNS1, MAMDC2, SEMA6A, PTH1R, LHFPL3, MME, PPP1R14A, ABCG1, PID1, PEAR1, HSPB6, CLEC14A, BCL6B, FOXF1, LTBP4, NDRG2, LRRK2, HHIP, CD34, CDO1, TOX2, C1orf162, AGTR1, ALPL, SLIT2, TOM1L2, MSRB3, FRMD3, CPAMD8, TACC1, SASH1, SPARCL1, ABCA3, FLI1, LAMC3, GJA4, ADCY4, ENG, IRX2, ADAMTSL3, GATA2, NDST1, NRN1, SYNM, AQP1, PRKCE, BAALC, RSPO4, DPYSL2, CCBE1, PDLIM1, KANK2, LIMCH1, LMO2, DKK2, JDP2, PRKCZ, SLIT3, FERMT2, PKDCC, SULT1C4, SLC9A3R2, NKD2, NDRG4, GATA6, HYAL1, SORBS1, NEDD9, CLDN11, SEPP1, CBX7, TBX4, CD302, CCDC69, CSF3R, SCD5, ALDH1A2, RBP4, LEPR, SH3BP5, MAPK4, CYB5R3, ALOX5, ITPRIP, ITGA8, KCNJ8, C1orf198, THSD1, SCNN1B, SMAD7, SOSTDC1, ARRB1, SMAD6, RNF122, PLEKHO2, NFAM1, TBX3, KLF13, APBB, FGF2, GSTM5, C2orf40, SSTR1, NFIX, MAP3K8, FPR2, HPCAL1, TUBB6, EFEMP1, TNXB, RXRG, PTGER4, SERPING1, MAL, ITGAL, RSPO2, AMOTL1, NR4A3, BTG2, LIFR, HLF, SPTBN1, F2RL3, RGS16, BMP6, FOXF2, ID3, TDRD10, SOD3, GJA5, RFX2, SEMA6D, C7, NPR3, FGFR2, FIBIN, CDKN1C, CA3, HIF3A, LMO7, PCDH17, FZD4, SLC25A25, ZBTB16, IRX1, DUSP1, SRGN, GPR116, FOXA2, VAMP5, HLA-DPB1, SFRP5, TMPRSS2, SCARA5, PDPN, HAS1, HOPX, EGR2, CFTR, NDUFA4L2, NTM, CLU, AKAP12, SLC26A9

**Figure 4 -:**
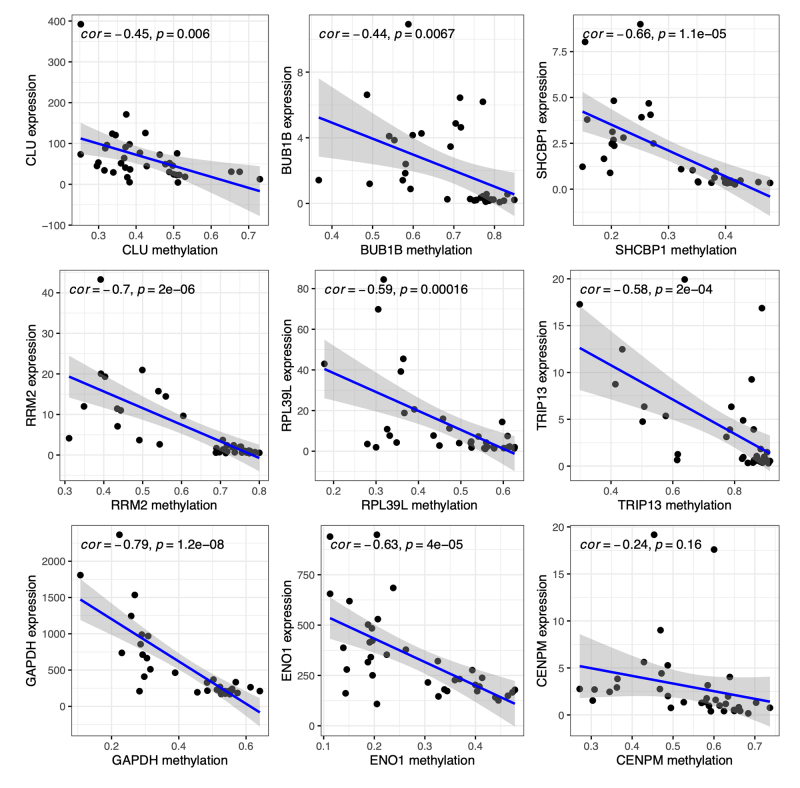
Scatter plots illustrating the Pearson correlation between gene expression (y-axis) and DNA methylation (x-axis) levels of the 9 methylated DE-DDRGs in paired normal and tumor samples (n = 18). Each dot represents data for one of the 36 samples. The blue line indicates the best fit, while the gray shaded area represents the 95% confidence interval (CI). Pearson correlation coefficients and p-values are provided for each gene.

Subsequently, expression profiles of the nine methylated DE-DDRGs from the TCGA-LUAD dataset were extracted and correlated with DNA methylation data. All nine gene expressions showed negative correlations with DNA methylation (Figure 4). Among them, GAPDH exhibited the strongest negative correlation with methylation (cor = -0.79, P = 1.2e-08), followed by RRM2 (cor = -0.7, P = 2e-06), SHCBP1 (cor = -0.66, P = 1.1e-05), and ENO1 (cor = -0.63, P = 4e-05). The remaining genes, RPL39L, TRIP13, CLU, and BUB1B, showed correlation coefficients ranging from -0.59 to -0.44 (all P < 0.05). However, the correlation between CENPM expression and methylation was relatively weak (cor = -0.24, P = 0.16).

### Patients with LUAD in the low-risk group have better survival than those at high risk

**Figure 5 -:**
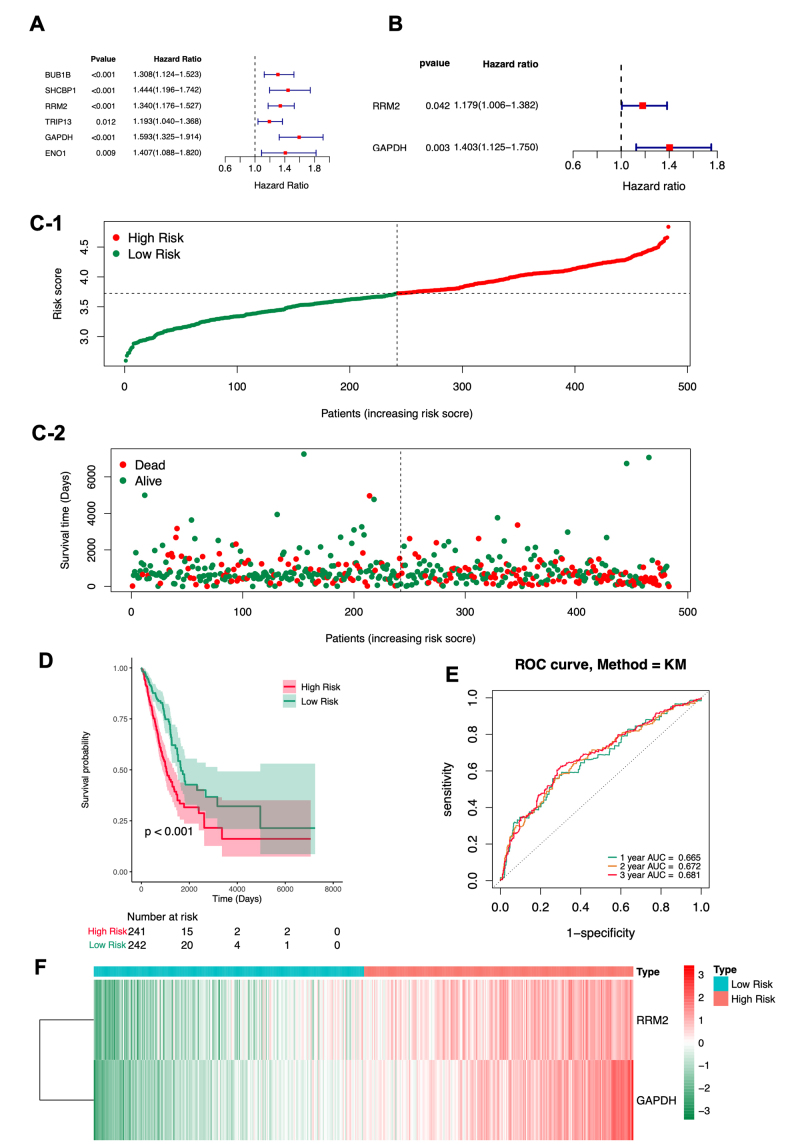
Evaluation of the prognostic prediction model based on the expression of two methylated DE-DDRGs in the training cohort TCGA-LUAD (n = 483). **A-B.** Forest plots from univariate (A) and multivariate (B) Cox regression analyses showing the association between the expression of methylated DE-DDRGs and survival outcomes. **C.** Risk score distribution in TCGA-LUAD patients, ranked in ascending order, with low-risk (green) and high-risk (red) groups identified by the threshold (vertical black line). Survival status and duration for each patient are shown below, with more deceased patients corresponding to higher risk scores. **D.** Kaplan-Meier survival curves comparing low-risk and high-risk groups. **E.** ROC curves for 1-, 2-, and 3-year overall survival. **F.** Heatmap showing the expression of the two methylated DE-DDRGs in patients with LUAD, with red indicating higher expression and green indicating lower expression.

To investigate the relationship between methylated DE-DDRGs and the survival of patients with LUAD, clinical data from the TCGA-LUAD cohort (n = 483) were extracted. Univariate Cox regression analysis, coupled with K-M survival analysis, was performed to identify variables significantly associated with OS in patients with LUAD. The analysis revealed that six of the nine methylated DE-DDRGs (BUB1B, SHCBP1, RRM2, TRIP13, GAPDH, and ENO1) were significantly correlated with OS (all P < 0.05; Figure 5 A ; Table S8). Notably, all of these genes were hypomethylated and highly expressed, with hazard ratios (HR) > 1, suggesting their potential role as pro-oncogenes in LUAD. Subsequently, multivariate Cox regression analysis with stepwise selection identified RRM2 (P = 0.042, HR = 1.179, 95% CI: 1.006-1.382) and GAPDH (P = 0.003, HR = 1.403, 95% CI: 1.125-1.750) as the most optimal predictors for constructing prognostic models in patients with LUAD from the six identified variables (Figure 5 B ; Table S9). The risk score was calculated as follows:

risk score=(0.164973625×expression value of RRM2)+(0.338623053×expression value of GAPDH) (2)

TCGA-LUAD patients were stratified into a high-risk group (n = 241) and a low-risk group (n = 242) based on the median value of the risk score (cutoff = 3.724466) (Table S10). Risk curves indicated that patient mortality increased progressively with higher risk scores (Figure 5 C ). Consistent with this, Kaplan-Meier survival curves demonstrated that patients with LUAD in the high-risk group had significantly poorer OS compared to the low-risk group (P < 0.001; Figure 5 D ). To assess the predictive power of the methylated DE-DDRGs-based risk signature, the AUC of the ROC curve was calculated. The AUC for 1-year OS was 0.665, 0.972 for 2-year OS, and 0.681 for 3-year OS (Figure 5 E ), confirming the risk score’s acceptable predictive validity for OS in patients with LUAD. A heatmap further revealed that both RRM2 and GAPDH exhibited high expression in the high-risk group (Figure 5 F ).

### RRM2 and GAPDH more accurately predict survival in patients with LUAD

**Figure 6 -:**
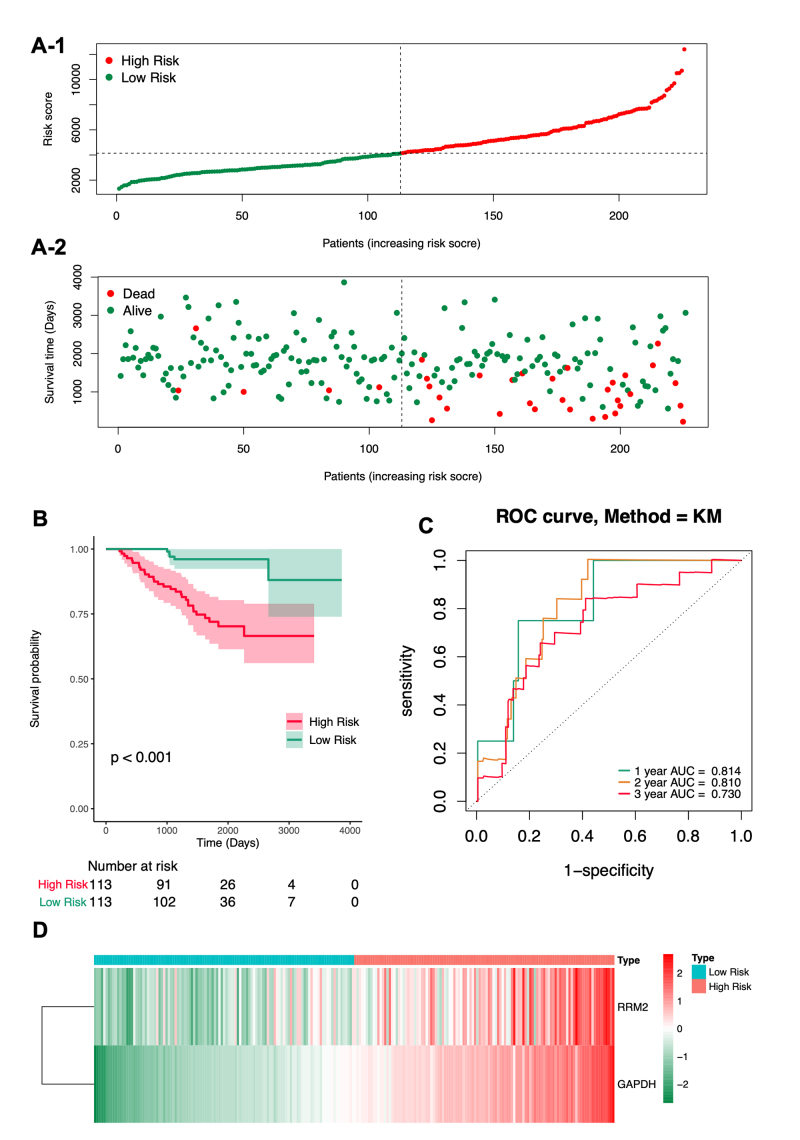
Validation of the prognostic prediction model based on the expression of two methylated DE-DDRGs in the independent external validation cohort GSE31210 (n = 226). **A.** Risk curve based on the risk score for each sample, with green and red representing low- and high-risk patients, respectively. The scatter plot shows survival status, with green dots indicating survival and red dots indicating death. **B.** Kaplan-Meier (KM) plot for overall survival (OS) based on risk score derived from the two-gene signature in the GSE31210 cohort. **C.** ROC curves for 1-, 2-, and 3-year overall survival. **D.** Heatmap displaying the expression levels of methylated DE-DDRGs in the high- and low-risk groups.

The expression profiles of prognostic genes from the GSE31210 dataset were also extracted, and risk scores were calculated for each LUAD sample using the same formula. With a threshold of 4138.758, the 226 LUAD samples were divided into high- and low-risk groups, each containing 113 samples. Survival time, survival status, risk scores, and groupings for patients with LUAD in the independent external validation set are provided in Table S11. Risk curves and scatter plots were used to illustrate the relationship between risk scores and survival status in patients with LUAD. The results demonstrated that LUAD mortality was closely associated with the risk score in the external validation set (Figure 6 A ). Kaplan-Meier survival curves again showed that patients in the low-risk group had significantly better OS than those in the high-risk group (P < 0.001; Figure 6 B ), further supporting the prognostic value of the 2-gene-based risk score. Subsequently, the ROC curve confirmed the robustness of the risk score in predicting OS in patients with LUAD, with AUC values of 0.814 for 1-year OS, 0.810 for 2-year OS, and 0.730 for 3-year OS (Figure 6 C ). Additionally, the GSE68465 validation set was used to further validate the prognostic model. The survival rate of the high-risk group was significantly lower than that of the low-risk group. The ROC curve AUCs for the 1-, 2-, and 3-year time points were all greater than 0.6 (Figure S1; Table S12). A heatmap of the expression of the two methylated DE-DDRGs in LUAD revealed that both RRM2 and GAPDH were highly expressed in the high-risk group (Figure 6 D ). Independent external validation demonstrated that these two genetic markers were effective in predicting the OS of patients with LUAD.

### Nomograms constructed based on pathologic_stage and risk score accurately predict LUAD survival

**Figure 7 -:**
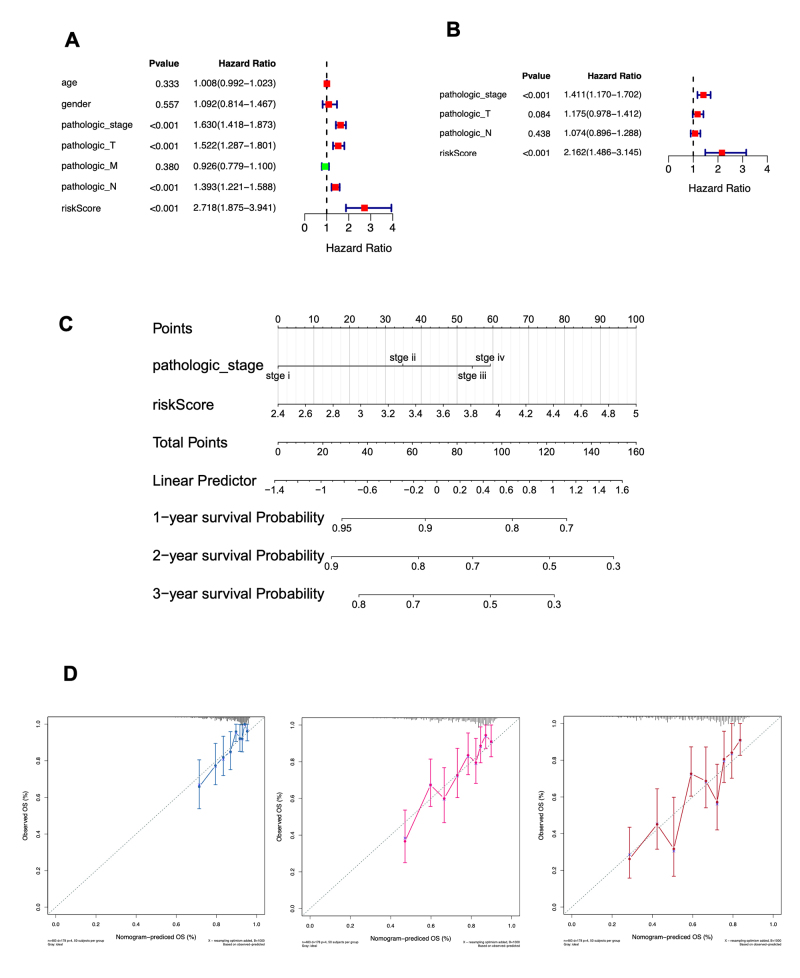
Evaluation of the independent prognostic value of risk score based on two methylated DE-DDRGs and clinical factors. **A-B.** Forest plots showing the results of univariable (A) and multivariable (B) Cox regression analyses for the association between risk scores and other clinical factors. **C.** A nomogram combining risk score and other clinical indicators to predict 1-, 2-, and 3-year OS in patients with LUAD. **D.** Calibration plots assessing the predicted versus actual 1-, 2-, and 3-year survival rates in the TCGA-LUAD cohort. The x-axis and y-axis represent the predicted and actual survival rates, respectively. The solid line indicates the predicted nomogram, and the vertical line represents the 95% confidence interval.

The independent prognostic value of the risk score was evaluated, revealing significant associations with clinical factors. As shown in Figure 7 A , pathologic stage (HR = 1.630, 95% CI = 1.418-1.873, P < 0.001), pathologic T (HR = 1.522, 95% CI = 1.287-1.801, P < 0.001), pathologic N (HR = 1.393, 95% CI = 1.221-1.588, P < 0.001), and risk scores (HR = 2.718, 95% CI = 1.875-3.941, P < 0.001) were all significantly correlated with survival in patients with LUAD. Multivariate Cox analysis of clinical parameters identified the risk score (HR = 2.162, 95% CI = 1.486-3.145, P < 0.001) and pathologic stage (HR = 1.411, 95% CI = 1.170-1.702, P < 0.001) as independent prognostic factors for OS in patients with LUAD (Figure 7 B ). A nomogram was constructed to predict 1-, 2-, and 3-year OS using the TCGA-LUAD dataset and the identified independent prognostic factors (Figure 7 C ). The model demonstrated a C-index of 0.698. Additionally, the calibration curve validated the accuracy of the nomogram’s predictive capability (Figure 7 D ).

### Lower immune cell abundance and immune scores were associated with poorer OS in patients with LUAD.

**Figure 8 -:**
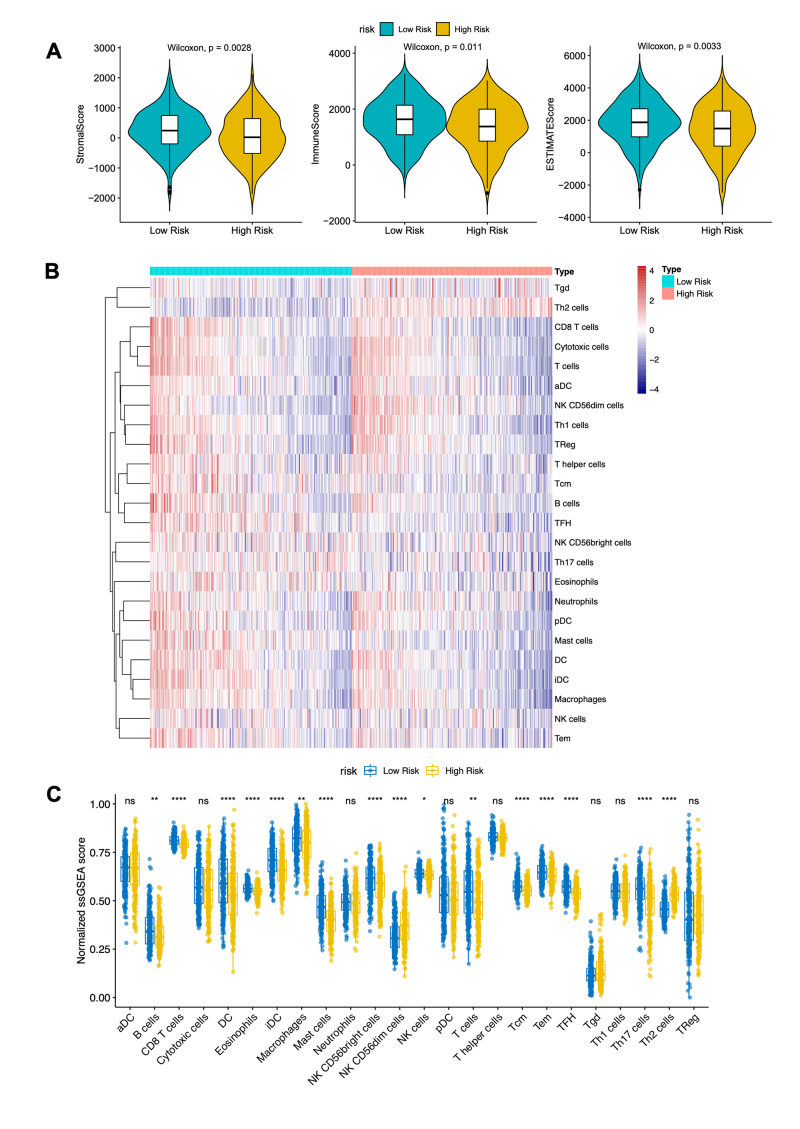
The risk prediction model is associated with differential profiles for the tumor microenvironment. **A.** Box plot illustrating the stromal scores (left), immune scores (middle), and ESTIMATE scores (right) in the high- and low-risk groups. **B.** Heatmap showing the immune cell infiltration patterns between high- and low-risk groups calculated by ssGSEA. **C.** Comparison of normalized ssGSEA scores (NES) of immune cells between the low-risk and high-risk groups, where 14 immune cells showed higher NES in the low-risk group (P < 0.05).

The tumor microenvironment (TME) is well-documented as influencing tumor progress nosis (Liu et al., 2018; Miranda et al., 2019). In our study, significant differences were observed in immune scores, stromal scores, and ESTIMATE scores between the high- and low-risk groups (P < 0.05), with all scores being higher in the low-risk group (Figure 8 A ). To further assess the relationship between the 2-methylated DE-DDRGs-based prognostic signature and immune cell composition in the TME, the abundance of various immune cells was estimated using ssGSEA (Figure 8 B ). A comparison of immune cell abundance between the low- and high-risk groups revealed 16 differentially distributed immune cells (Figure 8 C ). Specifically, in the low-risk group, NK CD56^dim^ cells and Th2 cells were significantly reduced, while B cells, CD8 T cells, DCs, eosinophils, iDCs, macrophages, mast cells, NK CD56^bright^ cells, NK cells, T cells, Tcm, Tem, TFH, and Th17 cells were significantly increased (P < 0.05). These results suggest that risk scores are negatively correlated with immune cell abundance, implying that lower levels of immune cell infiltration, along with lower immune scores, stromal scores, and ESTIMATE scores, are associated with poor OS in patients with LUAD.

### Patients in the low-risk group may benefit from immunotherapy

**Figure 9 -:**
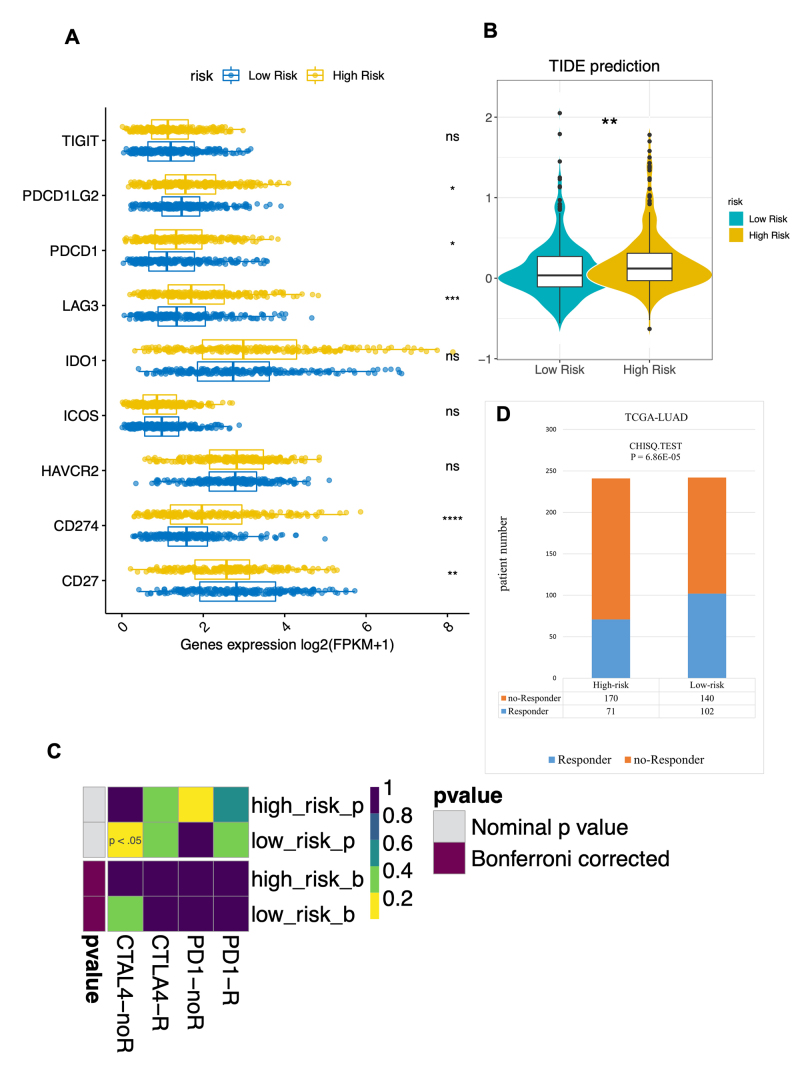
The risk prediction model is associated with differential profiles for immunotherapy. **A.** Gene expression levels of immune checkpoint molecules in the high- and low-risk groups in the TCGA-LUAD cohort. **B.** TIDE scores of the high- and low-risk groups using samples from the TCGA dataset. **C.** Immunotherapy responses between the high- and low-risk groups in the TCGA dataset. **D.** The proportion of patients who responded to immunotherapy in the high- and low-risk groups in the TCGA dataset.

Based on previous findings, a correlation between the risk score and immune markers, particularly well-established immune checkpoints, was hypothesized. To test this, the expression levels of immune checkpoint molecules were compared between the high- and low-risk groups. CD27 was predominantly expressed in the low-risk group, whereas PDCD1LG2, PDCD1 (PD1), LAG3, and CD274 (PD-L1) were significantly elevated in the high-risk group (P < 0.05; Figure 9 A ). Additionally, immunotherapy prediction scores for anti-PD1 and anti-CTLA4 were calculated using the TIDE algorithm. The high-risk group exhibited elevated TIDE scores (Figure 9 B ). Subclass mapping analysis further indicated that the high-risk group showed no response to anti-CTLA4 treatment (Figure 9 C ). Conversely, a higher proportion of patients in the low-risk group responded to immune checkpoint inhibitor therapy (Figure 9 D ). These results suggest that patients with LUAD in the low-risk group may be more susceptible to immune checkpoint inhibitor therapy.

### RRM2 and GAPDH were significantly overexpressed in the disease group

**Figure 10 -:**
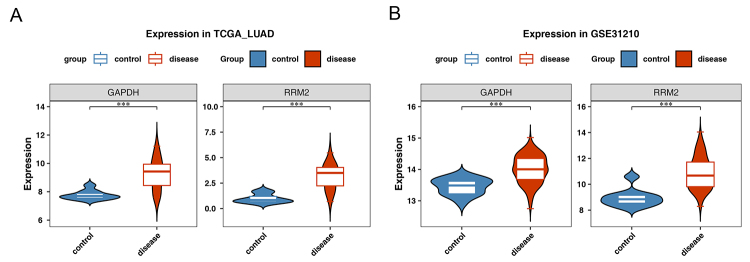
Validation of expression levels of RRM2 and GAPDH in different datasets. **A.** TCGA_LUAD dataset. **B.** GSE31210 dataset.

**Figure 11-:**
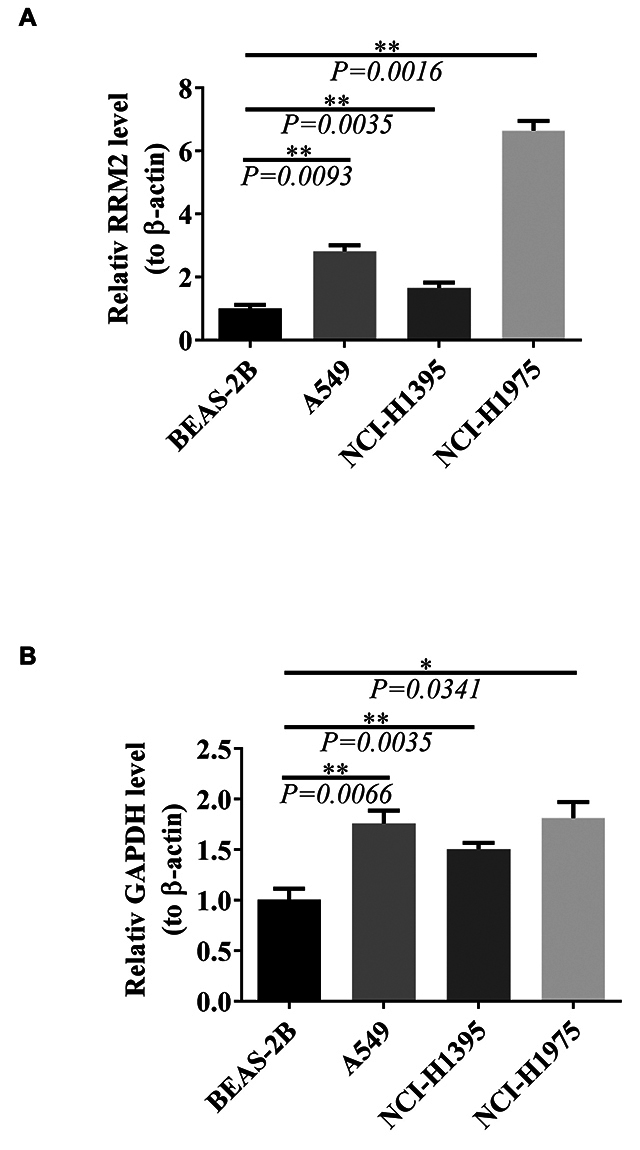
A-B. The mRNA expression of RRM2 (A) and GAPDH (B) in three lung cancer cell lines (A549, NCI-H1395, and NCI-H1975) and the corresponding normal lung epithelial cells (BEAS-2B) by RT-PCR. *P < 0.05; **P < 0.01; ***P < 0.001; ****P < 0.0001.

Gene expression analysis across the training and validation sets confirmed a significant upregulation of RRM2 and GAPDH in the disease group, following the same expression trend (Figure 10). Furthermore, mRNA expression of RRM2 and GAPDH was assessed in three lung cancer cell lines (A549, NCI-H1395, and NCI-H1975) and normal lung epithelial cells (BEAS-2B) *via* RT-PCR, reinforcing the role of these prognostic genes in LUAD. The results showed a significant increase in RRM2 and GAPDH expression in the cancer cell lines compared to BEAS-2B (both P < 0.05) (Figure 11). These findings were consistent with the TCGA database, which also reported higher expression levels of RRM2 and GAPDH in LUAD tissues compared to normal tissues (Table S5).

## Discussion

Bioinformatics methods have made significant strides in the study of lung cancer and related diseases. Whole-genome sequencing of patients with lung cancer has unveiled multiple driver mutations and novel biomarkers, providing a foundation for early diagnosis and targeted therapies (Dang et al., 2021). Transcriptomic analyses, exploring gene expression changes in lung cancer cells, have proven valuable in offering important prognostic information for clinical applications (Tran *et al.,* 2023). With an increasingly sophisticated understanding of the tumor microenvironment, bioinformatics enables the analysis of immune cell infiltration characteristics and its correlation with patient prognosis. This study leverages bioinformatics techniques, utilizing transcriptomic and methylomic data from 18 paired normal and tumor tissue samples from the TCGA-LUAD cohort. A total of 401 aberrantly methylated, DEGs were identified, including 161 hypomethylated, upregulated genes and 240 hypermethylated, downregulated genes. Notably, two DNA damage repair-related genes, RRM2 and GAPDH, were found to be hypomethylated and overexpressed, correlating with poor prognosis. Univariate and multivariate analyses confirmed the prognostic significance of these genes. Furthermore, a risk prediction model based on the two methylated DE-DDRGs was developed and validated using two independent LUAD cohorts, demonstrating that high-risk patients consistently exhibited poorer prognoses. Both univariate and multivariate analyses identified pathological stage and risk score from the prediction model as independent prognostic factors. A nomogram, incorporating both the risk prediction model and pathological stage, was constructed to predict patient survival. Notably, this prognostic model represents the first of its kind for LUAD based on methylated DE-DDRGs.

RRM2 encodes the catalytic subunit of ribonucleotide reductase and is upregulated in response to DNA damage to ensure sufficient deoxynucleoside triphosphates for DNA repair (Zhang et al., 2009; Chen et al., 2019). RRM2 overexpression has been linked to enhanced cell transformation, cancer cell proliferation, and tumor progression (Fan et al., 1996 *,* 1998; Morikawa et al., 2010 a ). Additionally, high RRM2 expression may increase sensitivity to immunotherapy (Zhou et al., 2022). Numerous studies have demonstrated that elevated RRM2 expression correlates with poor survival outcomes and has been identified as a potential prognostic biomarker across various cancer types, including bladder cancer (Morikawa *et al.,* 2010b; Zhou *et al.,* 2022), colorectal cancer (Liu, X et al., 2013), cervical cancer (Wang et al., 2020), NSCLC (Souglakos et al., 2008; Hsu et al., 2011; Ma et al., 2020), breast cancer (Abdel-Rahman et al., 2022; Shi et al., 2022), and hepatocellular carcinoma (Qin et al., 2023). 

GAPDH encodes a key enzyme in glycolysis and interacts with various proteins involved in DNA repair, playing a pivotal role in regulating cell death and carcinogenesis (Colell et al., 2009; Zhang et al., 2015; Kosova et al., 2017; Pacchiana et al., 2022). Overexpression of GAPDH has been linked to poorer prognosis in several solid tumors, including colorectal cancer (CRC) (Tarrado-Castellarnau et al., 2017) and NSCLC (Puzone et al., 2013; Wang, D et al., 2013). Additionally, GAPDH overexpression may inhibit apoptotic signaling pathways (Zhang *et al.,* 2015), thereby promoting tumor cell survival. In contrast, GAPDH knockdown significantly inhibits cell proliferation and migration/invasion *in vivo* (Hao et al., 2015). These findings underscore the critical role of GAPDH in tumor progression. Given its involvement in DNA repair and cell survival, the transcription and translation of RRM2 and GAPDH must be precisely regulated through epigenetic, transcriptional, and post-transcriptional modifications. Our study identified RRM2 and GAPDH as hypomethylated and upregulated genes in LUAD, suggesting that their hypomethylation leads to increased expression, contributing to enhanced tumor aggressiveness, drug resistance, and poor patient prognosis.

Immune checkpoint inhibitors (ICIs) have become standard therapies for NSCLC; however, not all patients respond due to the complex and evolving interactions between cancer cells and the immune system. Numerous studies have explored biomarkers to predict ICI response (Li et al., 2022; Shiravand et al., 2022; Wang, C et al., 2022). A recent review of the molecular mechanisms of DDR in immunity and cancer immunotherapy highlighted that mRNA levels of many DDR factors were negatively correlated with the infiltration of cytotoxic CD8^+^ T cells across different cancer types (Ye et al., 2021). Several studies have demonstrated that alterations in DDR pathway genes influence the response to ICIs across various cancer types. These include changes in MMR status (Le et al., 2015 *,* 2017), deleterious DDR mutations in NSCLC (Rizvi et al., 2015; Ricciuti et al., 2020; Lau et al., 2022), and the involvement of four DDR genes-MRE11A, MSH2, ATM, and POLE-in CRC and bladder cancer (Zhang et al., 2021). Additionally, ERCC1-SNP status has been implicated in NSCLC (Aiello et al., 2020). Our results also demonstrate that high-risk patients with LUAD exhibit distinct tumor immune microenvironments, immune checkpoint profiles, and potentially altered responses to ICI therapy. Conversely, the low-risk group shows greater sensitivity to ICIs. Identifying novel prognostic biomarkers and alternative treatment strategies is essential for optimizing patient survival. Consequently, integrating biomarkers into immunotherapy can improve treatment outcomes. Specifically, the expression levels of RRM2 and GAPDH may predict patient response to immune checkpoint inhibitors, enabling the identification of candidates for immunotherapy. Moreover, monitoring the dynamic changes in these biomarkers could help assess potential risks and adverse reactions, allowing for tailored treatment strategies and personalized care plans.

This study identified RRM2 and GAPDH as potential biomarkers for LUAD. However, despite offering significant insights, several limitations should be noted. First, the ROC analysis of the risk model achieved an AUC value greater than 0.6. While an AUC value above 0.6 indicates predictive potential, it does not strongly suggest clinical applicability. Second, some confounding factors, such as patient age, gender, comorbidities, and lifestyle factors, were not fully accounted for in the analysis. Notably, smoking and non-smoking status significantly impact the biological characteristics of lung cancer and treatment response (Park et al., 2013). To address these limitations, future studies should include larger datasets to enhance the generalizability of the findings and better control for confounding variables. Additionally, this study identified 8 genes that were highly expressed and hypomethylated in LUAD tissues. While RRM2 and GAPDH were primarily selected for the risk score calculation in the multivariate Cox analysis, other genes also warrant attention. Investigating these genes will provide deeper insights into the biological complexity of LUAD and expand the pool of potential biomarkers. Although including more genes may reduce the statistical significance of the model, it can enhance the robustness and applicability of the risk scoring tool, improving its clinical relevance and generalizability. Future studies could incorporate regularized regression techniques, such as LASSO, to control model complexity, explore the roles of additional genes in LUAD, and facilitate personalized treatment strategies. Furthermore, RT-PCR was employed to validate the expression of RRM2 and GAPDH at the cellular level, with results consistent with bioinformatics analysis, reinforcing their potential as prognostic biomarkers. However, RT-PCR alone cannot fully capture the expression patterns of specific cell types or tissue regions. To investigate the expression of target proteins, clinical samples could be collected for immunohistochemistry experiments to observe protein localization in tumor and normal tissues. This approach would provide more clinically relevant data, further validating the bioinformatics-based gene expression results.

## Conclusion

In conclusion, this study highlights two methylated DE-DDRGs and their association with poor prognosis and immunotherapy response in LUAD. These genes have the potential to serve as biomarkers for patient screening, prognostication, and therapeutic decision-making. However, further validation, particularly through larger clinical trials, is necessary to confirm their predictive power and clinical utility.

## Supplementary material

The following online material is available for this article:

Figure S1 - Validation of the prognostic prediction model based on two methylated DE-DDRGs’ expression in the independent external validation cohort GSE68465 (n = 442).

Table S1 - 243 DNA damage-related genes from the CancerSEA dataset.

Table S2 - 387 DNA repair-related genes from the CancerSEA dataset.

Table S3 - 295 DNA damage & repair-related genes from MSigDB.

Table S4 - 696 DDR-related genes (DDRGs; de-duplicated genes;) from the CancerSEA database and MSigDB database.

Table S5 - Differentially expressed genes (DEGs) in lung adenocarcinoma (LUAD) including up-regulated and down-regulated genes .

Table S6 - Hypermethylated genes in lung adenocarcinoma (LUAD).

Table S7 - Hypomethylated genes in lung adenocarcinoma (LUAD).

Table S8 - Summary of the results from the univariate Cox regression analysis in TCGA-LUAD patients.

Table S9 - Summary of the results from the multivariate Cox regression analysis in TCGA-LUAD patients.

Table S10 - Clinical information, risk score, and grouping information related to TCGA-LUAD patients.

Table S11 - Information on survival time, survival status, risk score, and grouping of LUAD patients in the GSE31210 cohort.

Table S12 - Information on survival time, survival status, risk score, and grouping of LUAD patients in the GSE68465 cohort.
