# Supplementary material for: DNA damage repair-related methylated genes RRM2 and GAPDH are prognostic biomarkers associated with immunotherapy for lung adenocarcinoma
Source: Genet Mol Biol. 2025 May 9;48(2):e20240138. doi: 10.1590/1678-4685-GMB-2024-0138 (PMC12063672; doi:10.1590/1678-4685-GMB-2024-0138)
Supplement: Figure S1 - [file 1415-4757-GMB-48-02-e20240138-s1.pdf]

Genetics and Molecular Biology
https://doi.org/10.1590/1678-4685-GMB-2024-0138

Supplementary Material to “DNA damage repair-related methylated genes RRM2 and GAPDH are prognostic biomarkers associated with immunotherapy for lung adenocarcinoma”

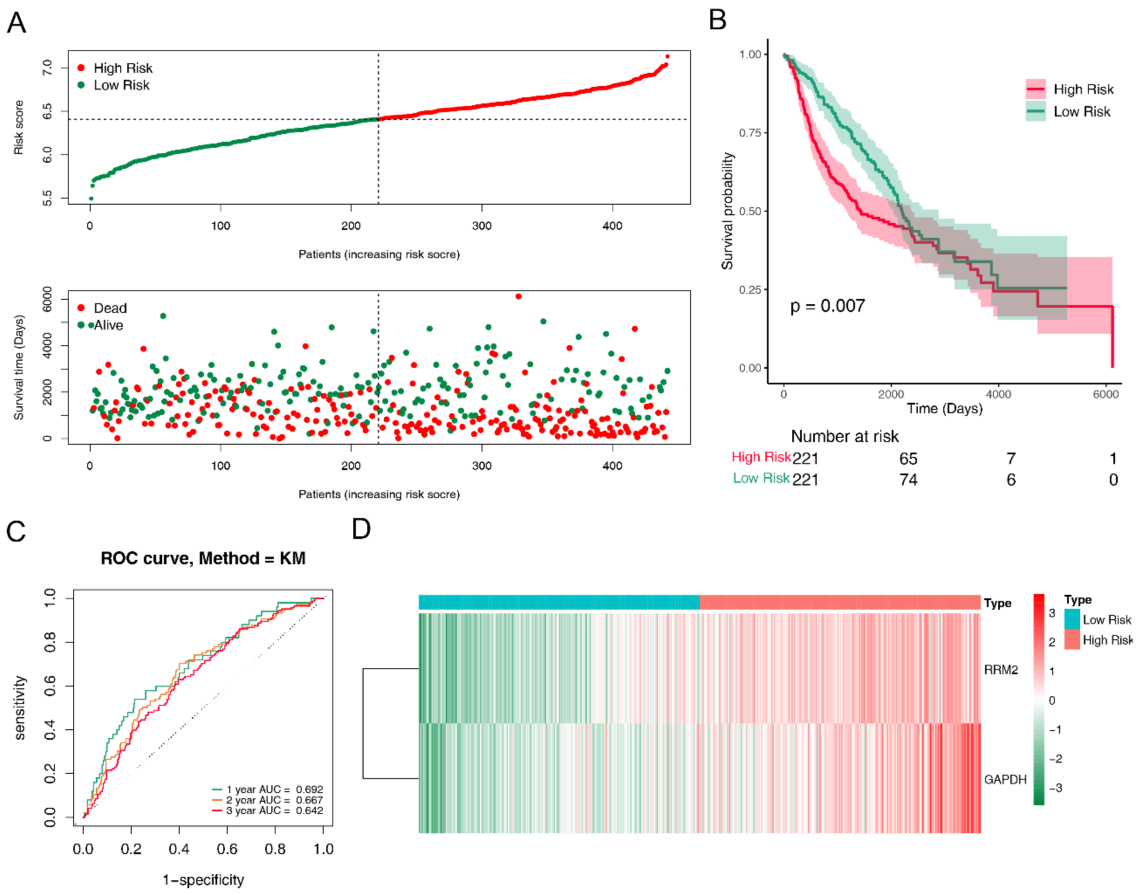

Figure S1 - Validation of the prognostic prediction model based on two methylated DE-DDRGs' expression in the independent external validation cohort GSE68465 (n = 442). A. The risk curve based on the risk score of each sample, with green and red representing low- and high-risk patients, respectively. The scatter plot represents survival status, with green and red dots indicating survival and death, respectively. B. Kaplan-Meier (KM) plot for overall survival (OS) based on the risk score of the two-gene signature in the GSE68465 cohort. C. ROC curves for 1-, 2-, and 3-year overall survival. D. Heatmap showing the expression levels of methylated DE-DDRGs in the high- and low-risk groups.
