# Supplementary material for: DNA damage repair-related methylated genes RRM2 and GAPDH are prognostic biomarkers associated with immunotherapy for lung adenocarcinoma
Source: Genet Mol Biol. 2025 May 9;48(2):e20240138. doi: 10.1590/1678-4685-GMB-2024-0138 (PMC12063672; doi:10.1590/1678-4685-GMB-2024-0138)
Supplement: Table S1 - [file 1415-4757-GMB-48-02-e20240138-s2.pdf]

Genetics and Molecular Biology
https://doi.org/10.1590/1678-4685-GMB-2024-0138

# Supplementary Material to "DNA damage repair-related methylated genes RRM2 and GAPDH are prognostic biomarkers associated with immunotherapy for lung adenocarcinoma"

**Table S1 -** 243 DNA damage-related genes from the CancerSEA dataset.

| No. | Ensemble ID | Symbol | No. dataset | Direction |
|---|---|---|---|---|
| 1 | ENSG00000132646 | PCNA | 11 | positive |
| 2 | ENSG00000117632 | STMN1 | 10 | positive |
| 3 | ENSG00000165304 | MELK | 10 | positive |
| 4 | ENSG00000170312 | CDK1 | 10 | positive |
| 5 | ENSG00000176890 | TYMS | 10 | positive |
| 6 | ENSG00000228716 | DHFR | 10 | positive |
| 7 | ENSG00000077152 | UBE2T | 9 | positive |
| 8 | ENSG00000120802 | TMPO | 9 | positive |
| 9 | ENSG00000123416 | TUBA1B | 9 | positive |
| 10 | ENSG00000135486 | HNRNPA1 | 9 | positive |
| 11 | ENSG00000164032 | H2AFZ | 9 | positive |
| 12 | ENSG00000167088 | SNRPD1 | 9 | positive |
| 13 | ENSG00000175063 | UBE2C | 9 | positive |
| 14 | ENSG00000189403 | HMGB1 | 9 | positive |
| 15 | ENSG00000196230 | TUBB | 9 | positive |
| 16 | ENSG00000198830 | HMGN2 | 9 | positive |
| 17 | ENSG00000088325 | TPX2 | 8 | positive |
| 18 | ENSG00000117724 | CENPF | 8 | positive |
| 19 | ENSG00000123975 | CKS2 | 8 | positive |
| 20 | ENSG00000131747 | TOP2A | 8 | positive |
| 21 | ENSG00000137804 | NUSAP1 | 8 | positive |
| 22 | ENSG00000167325 | RRM1 | 8 | positive |

| No. | Ensemble ID | Symbol | No. dataset | Direction |
|---|---|---|---|---|
| 23 | ENSG00000171848 | RRM2 | 8 | positive |
| 24 | ENSG00000181163 | NPM1 | 8 | positive |
| 25 | ENSG00000198901 | PRC1 | 8 | positive |
| 26 | ENSG00000011426 | ANLN | 7 | positive |
| 27 | ENSG00000066279 | ASPM | 7 | positive |
| 28 | ENSG00000124207 | CSE1L | 7 | positive |
| 29 | ENSG00000126787 | DLGAP5 | 7 | positive |
| 30 | ENSG00000132341 | RAN | 7 | positive |
| 31 | ENSG00000136824 | SMC2 | 7 | positive |
| 32 | ENSG00000138160 | KIF11 | 7 | positive |
| 33 | ENSG00000143228 | NUF2 | 7 | positive |
| 34 | ENSG00000143621 | ILF2 | 7 | positive |
| 35 | ENSG00000149554 | CHEK1 | 7 | positive |
| 36 | ENSG00000151725 | CENPU | 7 | positive |
| 37 | ENSG00000164104 | HMGB2 | 7 | positive |
| 38 | ENSG00000164109 | MAD2L1 | 7 | positive |
| 39 | ENSG00000167900 | TK1 | 7 | positive |
| 40 | ENSG00000168496 | FEN1 | 7 | positive |
| 41 | ENSG00000177889 | UBE2N | 7 | positive |
| 42 | ENSG00000187514 | PTMA | 7 | positive |
| 43 | ENSG00000203760 | CENPW | 7 | positive |
| 44 | ENSG00000005022 | SLC25A5 | 6 | positive |
| 45 | ENSG00000010292 | NCAPD2 | 6 | positive |
| 46 | ENSG00000075218 | GTSE1 | 6 | positive |
| 47 | ENSG00000089157 | RPLP0 | 6 | positive |
| 48 | ENSG00000089685 | BIRC5 | 6 | positive |
| 49 | ENSG00000093009 | CDC45 | 6 | positive |
| 50 | ENSG00000094916 | CBX5 | 6 | positive |
| 51 | ENSG00000104738 | MCM4 | 6 | positive |
| 52 | ENSG00000105011 | ASF1B | 6 | positive |
| 53 | ENSG00000105640 | RPL18A | 6 | positive |

| No. | Ensemble ID | Symbol | No. dataset | Direction |
|---|---|---|---|---|
| 54 | ENSG00000109971 | HSPA8 | 6 | positive |
| 55 | ENSG00000111206 | FOXM1 | 6 | positive |
| 56 | ENSG00000112312 | GMNN | 6 | positive |
| 57 | ENSG00000112742 | TTK | 6 | positive |
| 58 | ENSG00000113810 | SMC4 | 6 | positive |
| 59 | ENSG00000115053 | NCL | 6 | positive |
| 60 | ENSG00000122566 | HNRNPA2B1 | 6 | positive |
| 61 | ENSG00000122952 | ZWINT | 6 | positive |
| 62 | ENSG00000124795 | DEK | 6 | positive |
| 63 | ENSG00000125835 | SNRPB | 6 | positive |
| 64 | ENSG00000128708 | HAT1 | 6 | positive |
| 65 | ENSG00000131153 | GINS2 | 6 | positive |
| 66 | ENSG00000134690 | CDCA8 | 6 | positive |
| 67 | ENSG00000139618 | BRCA2 | 6 | positive |
| 68 | ENSG00000140525 | FANCI | 6 | positive |
| 69 | ENSG00000142945 | KIF2C | 6 | positive |
| 70 | ENSG00000145386 | CCNA2 | 6 | positive |
| 71 | ENSG00000148773 | MKI67 | 6 | positive |
| 72 | ENSG00000149273 | RPS3 | 6 | positive |
| 73 | ENSG00000162607 | USP1 | 6 | positive |
| 74 | ENSG00000163918 | RFC4 | 6 | positive |
| 75 | ENSG00000166451 | CENPN | 6 | positive |
| 76 | ENSG00000166508 | MCM7 | 6 | positive |
| 77 | ENSG00000168393 | DTYMK | 6 | positive |
| 78 | ENSG00000169679 | BUB1 | 6 | positive |
| 79 | ENSG00000171241 | SHCBP1 | 6 | positive |
| 80 | ENSG00000171858 | RPS21 | 6 | positive |
| 81 | ENSG00000173207 | CKS1B | 6 | positive |
| 82 | ENSG00000183856 | IQGAP3 | 6 | positive |
| 83 | ENSG00000008988 | RPS20 | 5 | positive |
| 84 | ENSG00000012048 | BRCA1 | 5 | positive |

| No. | Ensemble ID | Symbol | No. dataset | Direction |
|---|---|---|---|---|
| 85 | ENSG00000065328 | MCM10 | 5 | positive |
| 86 | ENSG00000069275 | NUCKS1 | 5 | positive |
| 87 | ENSG00000076382 | SPAG5 | 5 | positive |
| 88 | ENSG00000079616 | KIF22 | 5 | positive |
| 89 | ENSG00000080986 | NDC80 | 5 | positive |
| 90 | ENSG00000083845 | RPS5 | 5 | positive |
| 91 | ENSG00000085840 | ORC1 | 5 | positive |
| 92 | ENSG00000087586 | AURKA | 5 | positive |
| 93 | ENSG00000094804 | CDC6 | 5 | positive |
| 94 | ENSG00000099901 | RANBP1 | 5 | positive |
| 95 | ENSG00000100316 | RPL3 | 5 | positive |
| 96 | ENSG00000103121 | CMC2 | 5 | positive |
| 97 | ENSG00000104889 | RNASEH2A | 5 | positive |
| 98 | ENSG00000105193 | RPS16 | 5 | positive |
| 99 | ENSG00000105968 | H2AFV | 5 | positive |
| 100 | ENSG00000106355 | LSM5 | 5 | positive |
| 101 | ENSG00000106399 | RPA3 | 5 | positive |
| 102 | ENSG00000109805 | NCAPG | 5 | positive |
| 103 | ENSG00000110958 | PTGES3 | 5 | positive |
| 104 | ENSG00000111602 | TIMELESS | 5 | positive |
| 105 | ENSG00000112081 | SRSF3 | 5 | positive |
| 106 | ENSG00000112118 | MCM3 | 5 | positive |
| 107 | ENSG00000113648 | H2AFY | 5 | positive |
| 108 | ENSG00000114346 | ECT2 | 5 | positive |
| 109 | ENSG00000115875 | SRSF7 | 5 | positive |
| 110 | ENSG00000116161 | CACYBP | 5 | positive |
| 111 | ENSG00000120699 | EXOSC8 | 5 | positive |
| 112 | ENSG00000121152 | NCAPH | 5 | positive |
| 113 | ENSG00000123219 | CENPK | 5 | positive |
| 114 | ENSG00000125944 | HNRNPR | 5 | positive |
| 115 | ENSG00000131462 | TUBG1 | 5 | positive |

| No. | Ensemble ID | Symbol | No. dataset | Direction |
|---|---|---|---|---|
| 116 | ENSG00000131469 | RPL27 | 5 | positive |
| 117 | ENSG00000132780 | NASP | 5 | positive |
| 118 | ENSG00000134057 | CCNB1 | 5 | positive |
| 119 | ENSG00000137154 | RPS6 | 5 | positive |
| 120 | ENSG00000137807 | KIF23 | 5 | positive |
| 121 | ENSG00000139734 | DIAPH3 | 5 | positive |
| 122 | ENSG00000142937 | RPS8 | 5 | positive |
| 123 | ENSG00000143401 | ANP32E | 5 | positive |
| 124 | ENSG00000143476 | DTL | 5 | positive |
| 125 | ENSG00000143933 | CALM2 | 5 | positive |
| 126 | ENSG00000143977 | SNRPG | 5 | positive |
| 127 | ENSG00000144034 | TPRKB | 5 | positive |
| 128 | ENSG00000144381 | HSPD1 | 5 | positive |
| 129 | ENSG00000145425 | RPS3A | 5 | positive |
| 130 | ENSG00000147274 | RBMX | 5 | positive |
| 131 | ENSG00000147403 | RPL10 | 5 | positive |
| 132 | ENSG00000147604 | RPL7 | 5 | positive |
| 133 | ENSG00000149100 | EIF3M | 5 | positive |
| 134 | ENSG00000150753 | CCT5 | 5 | positive |
| 135 | ENSG00000152234 | ATP5F1A | 5 | positive |
| 136 | ENSG00000154473 | BUB3 | 5 | positive |
| 137 | ENSG00000156802 | ATAD2 | 5 | positive |
| 138 | ENSG00000157456 | CCNB2 | 5 | positive |
| 139 | ENSG00000161016 | RPL8 | 5 | positive |
| 140 | ENSG00000163808 | KIF15 | 5 | positive |
| 141 | ENSG00000164611 | PTTG1 | 5 | positive |
| 142 | ENSG00000164754 | RAD21 | 5 | positive |
| 143 | ENSG00000165480 | SKA3 | 5 | positive |
| 144 | ENSG00000165916 | PSMC3 | 5 | positive |
| 145 | ENSG00000167747 | C19orf48 | 5 | positive |
| 146 | ENSG00000168078 | PBK | 5 | positive |

| No. | Ensemble ID | Symbol | No. dataset | Direction |
|---|---|---|---|---|
| 147 | ENSG00000171863 | RPS7 | 5 | positive |
| 148 | ENSG00000172115 | CYCS | 5 | positive |
| 149 | ENSG00000174444 | RPL4 | 5 | positive |
| 150 | ENSG00000175305 | CCNE2 | 5 | positive |
| 151 | ENSG00000179750 | APOBEC3B | 5 | positive |
| 152 | ENSG00000182481 | KPNA2 | 5 | positive |
| 153 | ENSG00000182628 | SKA2 | 5 | positive |
| 154 | ENSG00000197061 | HIST1H4C | 5 | positive |
| 155 | ENSG00000237649 | KIFC1 | 5 | positive |
| 156 | ENSG00000276043 | UHRF1 | 5 | positive |
| 157 | ENSG00000006634 | DBF4 | 4 | positive |
| 158 | ENSG00000014641 | MDH1 | 4 | positive |
| 159 | ENSG00000024526 | DEPDC1 | 4 | positive |
| 160 | ENSG00000049541 | RFC2 | 4 | positive |
| 161 | ENSG00000071539 | TRIP13 | 4 | positive |
| 162 | ENSG00000079246 | XRCC5 | 4 | positive |
| 163 | ENSG00000089009 | RPL6 | 4 | positive |
| 164 | ENSG00000090889 | KIF4A | 4 | positive |
| 165 | ENSG00000091651 | ORC6 | 4 | positive |
| 166 | ENSG00000092853 | CLSPN | 4 | positive |
| 167 | ENSG00000100526 | CDKN3 | 4 | positive |
| 168 | ENSG00000100749 | VRK1 | 4 | positive |
| 169 | ENSG00000105372 | RPS19 | 4 | positive |
| 170 | ENSG00000106144 | CASP2 | 4 | positive |
| 171 | ENSG00000108055 | SMC3 | 4 | positive |
| 172 | ENSG00000108064 | TFAM | 4 | positive |
| 173 | ENSG00000108298 | RPL19 | 4 | positive |
| 174 | ENSG00000109685 | NSD2 | 4 | positive |
| 175 | ENSG00000110700 | RPS13 | 4 | positive |
| 176 | ENSG00000111237 | VPS29 | 4 | positive |
| 177 | ENSG00000118193 | KIF14 | 4 | positive |

| No. | Ensemble ID | Symbol | No. dataset | Direction |
|---|---|---|---|---|
| 178 | ENSG00000119335 | SET | 4 | positive |
| 179 | ENSG00000119403 | PHF19 | 4 | positive |
| 180 | ENSG00000119969 | HELLS | 4 | positive |
| 181 | ENSG00000121211 | MND1 | 4 | positive |
| 182 | ENSG00000121621 | KIF18A | 4 | positive |
| 183 | ENSG00000123485 | HJURP | 4 | positive |
| 184 | ENSG00000124767 | GLO1 | 4 | positive |
| 185 | ENSG00000125743 | SNRPD2 | 4 | positive |
| 186 | ENSG00000126457 | PRMT1 | 4 | positive |
| 187 | ENSG00000129173 | E2F8 | 4 | positive |
| 188 | ENSG00000129195 | PIMREG | 4 | positive |
| 189 | ENSG00000130816 | DNMT1 | 4 | positive |
| 190 | ENSG00000134419 | RPS15A | 4 | positive |
| 191 | ENSG00000137563 | GGH | 4 | positive |
| 192 | ENSG00000138182 | KIF20B | 4 | positive |
| 193 | ENSG00000139343 | SNRPF | 4 | positive |
| 194 | ENSG00000139921 | TMX1 | 4 | positive |
| 195 | ENSG00000142230 | SAE1 | 4 | positive |
| 196 | ENSG00000142534 | RPS11 | 4 | positive |
| 197 | ENSG00000142676 | RPL11 | 4 | positive |
| 198 | ENSG00000142731 | PLK4 | 4 | positive |
| 199 | ENSG00000143947 | RPS27A | 4 | positive |
| 200 | ENSG00000144554 | FANCD2 | 4 | positive |
| 201 | ENSG00000144713 | RPL32 | 4 | positive |
| 202 | ENSG00000146670 | CDCA5 | 4 | positive |
| 203 | ENSG00000149136 | SSRP1 | 4 | positive |
| 204 | ENSG00000152253 | SPC25 | 4 | positive |
| 205 | ENSG00000154518 | ATP5MC3 | 4 | positive |
| 206 | ENSG00000156136 | DCK | 4 | positive |
| 207 | ENSG00000156970 | BUB1B | 4 | positive |
| 208 | ENSG00000161888 | SPC24 | 4 | positive |

| No. | Ensemble ID | Symbol | No. dataset | Direction |
|---|---|---|---|---|
| 209 | ENSG00000161970 | RPL26 | 4 | positive |
| 210 | ENSG00000162063 | CCNF | 4 | positive |
| 211 | ENSG00000162521 | RBBP4 | 4 | positive |
| 212 | ENSG00000163923 | RPL39L | 4 | positive |
| 213 | ENSG00000164985 | PSIP1 | 4 | positive |
| 214 | ENSG00000166441 | RPL27A | 4 | positive |
| 215 | ENSG00000166801 | FAM111A | 4 | positive |
| 216 | ENSG00000166803 | PCLAF | 4 | positive |
| 217 | ENSG00000166851 | PLK1 | 4 | positive |
| 218 | ENSG00000168028 | RPSA | 4 | positive |
| 219 | ENSG00000169607 | CKAP2L | 4 | positive |
| 220 | ENSG00000170144 | HNRNPA3 | 4 | positive |
| 221 | ENSG00000170515 | PA2G4 | 4 | positive |
| 222 | ENSG00000170540 | ARL6IP1 | 4 | positive |
| 223 | ENSG00000170860 | LSM3 | 4 | positive |
| 224 | ENSG00000171320 | ESCO2 | 4 | positive |
| 225 | ENSG00000175216 | CKAP5 | 4 | positive |
| 226 | ENSG00000176208 | ATAD5 | 4 | positive |
| 227 | ENSG00000178999 | AURKB | 4 | positive |
| 228 | ENSG00000182774 | RPS17 | 4 | positive |
| 229 | ENSG00000186468 | RPS23 | 4 | positive |
| 230 | ENSG00000188612 | SUMO2 | 4 | positive |
| 231 | ENSG00000188846 | RPL14 | 4 | positive |
| 232 | ENSG00000196262 | PPIA | 4 | positive |
| 233 | ENSG00000197299 | BLM | 4 | positive |
| 234 | ENSG00000197451 | HNRNPAB | 4 | positive |
| 235 | ENSG00000198056 | PRIM1 | 4 | positive |
| 236 | ENSG00000198755 | RPL10A | 4 | positive |
| 237 | ENSG00000198826 | ARHGAP11A | 4 | positive |
| 238 | ENSG00000221983 | UBA52 | 4 | positive |
| 239 | ENSG00000231500 | RPS18 | 4 | positive |

| No. | Ensemble ID | Symbol | No. dataset | Direction |
|---|---|---|---|---|
| 240 | ENSG00000239672 | NME1 | 4 | positive |
| 241 | ENSG00000265241 | RBM8A | 4 | positive |
| 242 | ENSG00000101439 | CST3 | 4 | negative |
| 243 | ENSG00000120885 | CLU | 4 | negative |
