# Supplementary material for: DNA damage repair-related methylated genes RRM2 and GAPDH are prognostic biomarkers associated with immunotherapy for lung adenocarcinoma
Source: Genet Mol Biol. 2025 May 9;48(2):e20240138. doi: 10.1590/1678-4685-GMB-2024-0138 (PMC12063672; doi:10.1590/1678-4685-GMB-2024-0138)
Supplement: Table S2 - [file 1415-4757-GMB-48-02-e20240138-s3.pdf]

Genetics and Molecular Biology
https://doi.org/10.1590/1678-4685-GMB-2024-0138

# Supplementary Material to "DNA damage repair-related methylated genes RRM2 and GAPDH are prognostic biomarkers associated with immunotherapy for lung adenocarcinoma"

**Table S2 -** 387 DNA repair-related genes from the CancerSEA dataset.

| No. | Ensemble ID | Symbol | No. dataset | Direction |
|---|---|---|---|---|
| 1 | ENSG00000189403 | HMGB1 | 17 | positive |
| 2 | ENSG00000123416 | TUBA1B | 15 | positive |
| 3 | ENSG00000176890 | TYMS | 15 | positive |
| 4 | ENSG00000132646 | PCNA | 14 | positive |
| 5 | ENSG00000164032 | H2AFZ | 14 | positive |
| 6 | ENSG00000198830 | HMGN2 | 14 | positive |
| 7 | ENSG00000077152 | UBE2T | 13 | positive |
| 8 | ENSG00000117632 | STMN1 | 13 | positive |
| 9 | ENSG00000132341 | RAN | 13 | positive |
| 10 | ENSG00000167088 | SNRPD1 | 13 | positive |
| 11 | ENSG00000005022 | SLC25A5 | 12 | positive |
| 12 | ENSG00000168496 | FEN1 | 12 | positive |
| 13 | ENSG00000094804 | CDC6 | 11 | positive |
| 14 | ENSG00000104738 | MCM4 | 11 | positive |
| 15 | ENSG00000166508 | MCM7 | 11 | positive |
| 16 | ENSG00000167900 | TK1 | 11 | positive |
| 17 | ENSG00000173207 | CKS1B | 11 | positive |
| 18 | ENSG00000196230 | TUBB | 11 | positive |
| 19 | ENSG00000196262 | PPIA | 11 | positive |
| 20 | ENSG00000228716 | DHFR | 11 | positive |
| 21 | ENSG00000120802 | TMPO | 10 | positive |
| 22 | ENSG00000128050 | PAICS | 10 | positive |

| No. | Ensemble ID | Symbol | No. dataset | Direction |
|---|---|---|---|---|
| 23 | ENSG00000131153 | GINS2 | 10 | positive |
| 24 | ENSG00000143977 | SNRPG | 10 | positive |
| 25 | ENSG00000144554 | FANCD2 | 10 | positive |
| 26 | ENSG00000164104 | HMGB2 | 10 | positive |
| 27 | ENSG00000164109 | MAD2L1 | 10 | positive |
| 28 | ENSG00000167325 | RRM1 | 10 | positive |
| 29 | ENSG00000239672 | NME1 | 10 | positive |
| 30 | ENSG00000006625 | GGCT | 9 | positive |
| 31 | ENSG00000106355 | LSM5 | 9 | positive |
| 32 | ENSG00000112118 | MCM3 | 9 | positive |
| 33 | ENSG00000128708 | HAT1 | 9 | positive |
| 34 | ENSG00000128951 | DUT | 9 | positive |
| 35 | ENSG00000140525 | FANCI | 9 | positive |
| 36 | ENSG00000144381 | HSPD1 | 9 | positive |
| 37 | ENSG00000165304 | MELK | 9 | positive |
| 38 | ENSG00000171241 | SHCBP1 | 9 | positive |
| 39 | ENSG00000171848 | RRM2 | 9 | positive |
| 40 | ENSG00000012048 | BRCA1 | 8 | positive |
| 41 | ENSG00000065328 | MCM10 | 8 | positive |
| 42 | ENSG00000079246 | XRCC5 | 8 | positive |
| 43 | ENSG00000083845 | RPS5 | 8 | positive |
| 44 | ENSG00000089685 | BIRC5 | 8 | positive |
| 45 | ENSG00000099901 | RANBP1 | 8 | positive |
| 46 | ENSG00000100297 | MCM5 | 8 | positive |
| 47 | ENSG00000105968 | H2AFV | 8 | positive |
| 48 | ENSG00000106399 | RPA3 | 8 | positive |
| 49 | ENSG00000111247 | RAD51AP1 | 8 | positive |
| 50 | ENSG00000112312 | GMNN | 8 | positive |
| 51 | ENSG00000113810 | SMC4 | 8 | positive |
| 52 | ENSG00000122566 | HNRNPA2B1 | 8 | positive |
| 53 | ENSG00000122952 | ZWINT | 8 | positive |

| No. | Ensemble ID | Symbol | No. dataset | Direction |
|---|---|---|---|---|
| 54 | ENSG00000125835 | SNRPB | 8 | positive |
| 55 | ENSG00000131462 | TUBG1 | 8 | positive |
| 56 | ENSG00000134291 | TMEM106C | 8 | positive |
| 57 | ENSG00000139343 | SNRPF | 8 | positive |
| 58 | ENSG00000143476 | DTL | 8 | positive |
| 59 | ENSG00000146731 | CCT6A | 8 | positive |
| 60 | ENSG00000150753 | CCT5 | 8 | positive |
| 61 | ENSG00000163918 | RFC4 | 8 | positive |
| 62 | ENSG00000166803 | PCLAF | 8 | positive |
| 63 | ENSG00000167747 | C19orf48 | 8 | positive |
| 64 | ENSG00000170312 | CDK1 | 8 | positive |
| 65 | ENSG00000177889 | UBE2N | 8 | positive |
| 66 | ENSG00000181163 | NPM1 | 8 | positive |
| 67 | ENSG00000182628 | SKA2 | 8 | positive |
| 68 | ENSG00000011426 | ANLN | 7 | positive |
| 69 | ENSG00000049541 | RFC2 | 7 | positive |
| 70 | ENSG00000073111 | MCM2 | 7 | positive |
| 71 | ENSG00000092470 | WDR76 | 7 | positive |
| 72 | ENSG00000092853 | CLSPN | 7 | positive |
| 73 | ENSG00000093009 | CDC45 | 7 | positive |
| 74 | ENSG00000094916 | CBX5 | 7 | positive |
| 75 | ENSG00000109971 | HSPA8 | 7 | positive |
| 76 | ENSG00000111602 | TIMELESS | 7 | positive |
| 77 | ENSG00000111716 | LDHB | 7 | positive |
| 78 | ENSG00000116161 | CACYBP | 7 | positive |
| 79 | ENSG00000116288 | PARK7 | 7 | positive |
| 80 | ENSG00000119335 | SET | 7 | positive |
| 81 | ENSG00000119969 | HELLS | 7 | positive |
| 82 | ENSG00000120699 | EXOSC8 | 7 | positive |
| 83 | ENSG00000123485 | HJURP | 7 | positive |
| 84 | ENSG00000124207 | CSE1L | 7 | positive |

| No. | Ensemble ID | Symbol | No. dataset | Direction |
|---|---|---|---|---|
| 85 | ENSG00000124795 | DEK | 7 | positive |
| 86 | ENSG00000125743 | SNRPD2 | 7 | positive |
| 87 | ENSG00000131747 | TOP2A | 7 | positive |
| 88 | ENSG00000136824 | SMC2 | 7 | positive |
| 89 | ENSG00000137563 | GGH | 7 | positive |
| 90 | ENSG00000143947 | RPS27A | 7 | positive |
| 91 | ENSG00000149100 | EIF3M | 7 | positive |
| 92 | ENSG00000156802 | ATAD2 | 7 | positive |
| 93 | ENSG00000163468 | CCT3 | 7 | positive |
| 94 | ENSG00000170515 | PA2G4 | 7 | positive |
| 95 | ENSG00000170860 | LSM3 | 7 | positive |
| 96 | ENSG00000172115 | CYCS | 7 | positive |
| 97 | ENSG00000182004 | SNRPE | 7 | positive |
| 98 | ENSG00000182481 | KPNA2 | 7 | positive |
| 99 | ENSG00000197061 | HIST1H4C | 7 | positive |
| 100 | ENSG00000007968 | E2F2 | 6 | positive |
| 101 | ENSG00000069275 | NUCKS1 | 6 | positive |
| 102 | ENSG00000072501 | SMC1A | 6 | positive |
| 103 | ENSG00000076003 | MCM6 | 6 | positive |
| 104 | ENSG00000085840 | ORC1 | 6 | positive |
| 105 | ENSG00000088325 | TPX2 | 6 | positive |
| 106 | ENSG00000089157 | RPLP0 | 6 | positive |
| 107 | ENSG00000089220 | PEBP1 | 6 | positive |
| 108 | ENSG00000100162 | CENPM | 6 | positive |
| 109 | ENSG00000100804 | PSMB5 | 6 | positive |
| 110 | ENSG00000104889 | RNASEH2A | 6 | positive |
| 111 | ENSG00000105011 | ASF1B | 6 | positive |
| 112 | ENSG00000105185 | PDCD5 | 6 | positive |
| 113 | ENSG00000106028 | SSBP1 | 6 | positive |
| 114 | ENSG00000109084 | TMEM97 | 6 | positive |
| 115 | ENSG00000111237 | VPS29 | 6 | positive |

| No. | Ensemble ID | Symbol | No. dataset | Direction |
|---|---|---|---|---|
| 116 | ENSG00000111445 | RFC5 | 6 | positive |
| 117 | ENSG00000111669 | TPI1 | 6 | positive |
| 118 | ENSG00000114346 | ECT2 | 6 | positive |
| 119 | ENSG00000115268 | RPS15 | 6 | positive |
| 120 | ENSG00000115541 | HSPE1 | 6 | positive |
| 121 | ENSG00000117748 | RPA2 | 6 | positive |
| 122 | ENSG00000122406 | RPL5 | 6 | positive |
| 123 | ENSG00000122565 | CBX3 | 6 | positive |
| 124 | ENSG00000124562 | SNRPC | 6 | positive |
| 125 | ENSG00000126787 | DLGAP5 | 6 | positive |
| 126 | ENSG00000130816 | DNMT1 | 6 | positive |
| 127 | ENSG00000134001 | EIF2S1 | 6 | positive |
| 128 | ENSG00000136942 | RPL35 | 6 | positive |
| 129 | ENSG00000138182 | KIF20B | 6 | positive |
| 130 | ENSG00000139618 | BRCA2 | 6 | positive |
| 131 | ENSG00000143106 | PSMA5 | 6 | positive |
| 132 | ENSG00000143401 | ANP32E | 6 | positive |
| 133 | ENSG00000143621 | ILF2 | 6 | positive |
| 134 | ENSG00000146918 | NCAPG2 | 6 | positive |
| 135 | ENSG00000149273 | RPS3 | 6 | positive |
| 136 | ENSG00000149636 | DSN1 | 6 | positive |
| 137 | ENSG00000153044 | CENPH | 6 | positive |
| 138 | ENSG00000157456 | CCNB2 | 6 | positive |
| 139 | ENSG00000160752 | FDPS | 6 | positive |
| 140 | ENSG00000165672 | PRDX3 | 6 | positive |
| 141 | ENSG00000166226 | CCT2 | 6 | positive |
| 142 | ENSG00000166451 | CENPN | 6 | positive |
| 143 | ENSG00000168393 | DTYMK | 6 | positive |
| 144 | ENSG00000169679 | BUB1 | 6 | positive |
| 145 | ENSG00000175063 | UBE2C | 6 | positive |
| 146 | ENSG00000175334 | BANF1 | 6 | positive |

| No. | Ensemble ID | Symbol | No. dataset | Direction |
|---|---|---|---|---|
| 147 | ENSG00000188612 | SUMO2 | 6 | positive |
| 148 | ENSG00000198034 | RPS4X | 6 | positive |
| 149 | ENSG00000203760 | CENPW | 6 | positive |
| 150 | ENSG00000276043 | UHRF1 | 6 | positive |
| 151 | ENSG00000010292 | NCAPD2 | 5 | positive |
| 152 | ENSG00000075142 | SRI | 5 | positive |
| 153 | ENSG00000080986 | NDC80 | 5 | positive |
| 154 | ENSG00000091651 | ORC6 | 5 | positive |
| 155 | ENSG00000092199 | HNRNPC | 5 | positive |
| 156 | ENSG00000100749 | VRK1 | 5 | positive |
| 157 | ENSG00000100911 | PSME2 | 5 | positive |
| 158 | ENSG00000101003 | GINS1 | 5 | positive |
| 159 | ENSG00000103018 | CYB5B | 5 | positive |
| 160 | ENSG00000103121 | CMC2 | 5 | positive |
| 161 | ENSG00000105372 | RPS19 | 5 | positive |
| 162 | ENSG00000105640 | RPL18A | 5 | positive |
| 163 | ENSG00000106144 | CASP2 | 5 | positive |
| 164 | ENSG00000106153 | CHCHD2 | 5 | positive |
| 165 | ENSG00000106462 | EZH2 | 5 | positive |
| 166 | ENSG00000106588 | PSMA2 | 5 | positive |
| 167 | ENSG00000108055 | SMC3 | 5 | positive |
| 168 | ENSG00000109805 | NCAPG | 5 | positive |
| 169 | ENSG00000109919 | MTCH2 | 5 | positive |
| 170 | ENSG00000110700 | RPS13 | 5 | positive |
| 171 | ENSG00000111640 | GAPDH | 5 | positive |
| 172 | ENSG00000112081 | SRSF3 | 5 | positive |
| 173 | ENSG00000112742 | TTK | 5 | positive |
| 174 | ENSG00000113460 | BRIX1 | 5 | positive |
| 175 | ENSG00000114942 | EEF1B2 | 5 | positive |
| 176 | ENSG00000115053 | NCL | 5 | positive |
| 177 | ENSG00000115875 | SRSF7 | 5 | positive |

| No. | Ensemble ID | Symbol | No. dataset | Direction |
|---|---|---|---|---|
| 178 | ENSG00000116120 | FARSB | 5 | positive |
| 179 | ENSG00000117724 | CENPF | 5 | positive |
| 180 | ENSG00000118193 | KIF14 | 5 | positive |
| 181 | ENSG00000122026 | RPL21 | 5 | positive |
| 182 | ENSG00000123219 | CENPK | 5 | positive |
| 183 | ENSG00000123975 | CKS2 | 5 | positive |
| 184 | ENSG00000124614 | RPS10 | 5 | positive |
| 185 | ENSG00000124767 | GLO1 | 5 | positive |
| 186 | ENSG00000125691 | RPL23 | 5 | positive |
| 187 | ENSG00000125944 | HNRNPR | 5 | positive |
| 188 | ENSG00000131469 | RPL27 | 5 | positive |
| 189 | ENSG00000132780 | NASP | 5 | positive |
| 190 | ENSG00000135446 | CDK4 | 5 | positive |
| 191 | ENSG00000135486 | HNRNPA1 | 5 | positive |
| 192 | ENSG00000136518 | ACTL6A | 5 | positive |
| 193 | ENSG00000137154 | RPS6 | 5 | positive |
| 194 | ENSG00000137310 | TCF19 | 5 | positive |
| 195 | ENSG00000137804 | NUSAP1 | 5 | positive |
| 196 | ENSG00000138160 | KIF11 | 5 | positive |
| 197 | ENSG00000138326 | RPS24 | 5 | positive |
| 198 | ENSG00000138376 | BARD1 | 5 | positive |
| 199 | ENSG00000142230 | SAE1 | 5 | positive |
| 200 | ENSG00000142534 | RPS11 | 5 | positive |
| 201 | ENSG00000142937 | RPS8 | 5 | positive |
| 202 | ENSG00000143742 | SRP9 | 5 | positive |
| 203 | ENSG00000144354 | CDCA7 | 5 | positive |
| 204 | ENSG00000145425 | RPS3A | 5 | positive |
| 205 | ENSG00000145912 | NHP2 | 5 | positive |
| 206 | ENSG00000146263 | MMS22L | 5 | positive |
| 207 | ENSG00000147274 | RBMX | 5 | positive |
| 208 | ENSG00000147604 | RPL7 | 5 | positive |

| No. | Ensemble ID | Symbol | No. dataset | Direction |
|---|---|---|---|---|
| 209 | ENSG00000148773 | MKI67 | 5 | positive |
| 210 | ENSG00000151725 | CENPU | 5 | positive |
| 211 | ENSG00000154473 | BUB3 | 5 | positive |
| 212 | ENSG00000154518 | ATP5MC3 | 5 | positive |
| 213 | ENSG00000161016 | RPL8 | 5 | positive |
| 214 | ENSG00000164611 | PTTG1 | 5 | positive |
| 215 | ENSG00000165264 | NDUFB6 | 5 | positive |
| 216 | ENSG00000168028 | RPSA | 5 | positive |
| 217 | ENSG00000169813 | HNRNPF | 5 | positive |
| 218 | ENSG00000171863 | RPS7 | 5 | positive |
| 219 | ENSG00000173436 | MINOS1 | 5 | positive |
| 220 | ENSG00000174371 | EXO1 | 5 | positive |
| 221 | ENSG00000174444 | RPL4 | 5 | positive |
| 222 | ENSG00000174748 | RPL15 | 5 | positive |
| 223 | ENSG00000175768 | TOMM5 | 5 | positive |
| 224 | ENSG00000182774 | RPS17 | 5 | positive |
| 225 | ENSG00000183684 | ALYREF | 5 | positive |
| 226 | ENSG00000183856 | IQGAP3 | 5 | positive |
| 227 | ENSG00000187514 | PTMA | 5 | positive |
| 228 | ENSG00000189057 | FAM111B | 5 | positive |
| 229 | ENSG00000196531 | NACA | 5 | positive |
| 230 | ENSG00000198015 | MRPL42 | 5 | positive |
| 231 | ENSG00000198056 | PRIM1 | 5 | positive |
| 232 | ENSG00000198901 | PRC1 | 5 | positive |
| 233 | ENSG00000231500 | RPS18 | 5 | positive |
| 234 | ENSG00000237649 | KIFC1 | 5 | positive |
| 235 | ENSG00000241468 | ATP5MF | 5 | positive |
| 236 | ENSG00000206503 | HLA-A | 5 | negative |
| 237 | ENSG00000001630 | CYP51A1 | 4 | positive |
| 238 | ENSG00000008988 | RPS20 | 4 | positive |
| 239 | ENSG00000013275 | PSMC4 | 4 | positive |

| No. | Ensemble ID | Symbol | No. dataset | Direction |
|---|---|---|---|---|
| 240 | ENSG00000024526 | DEPDC1 | 4 | positive |
| 241 | ENSG00000041357 | PSMA4 | 4 | positive |
| 242 | ENSG00000071539 | TRIP13 | 4 | positive |
| 243 | ENSG00000074800 | ENO1 | 4 | positive |
| 244 | ENSG00000076382 | SPAG5 | 4 | positive |
| 245 | ENSG00000079616 | KIF22 | 4 | positive |
| 246 | ENSG00000080824 | HSP90AA1 | 4 | positive |
| 247 | ENSG00000084623 | EIF3I | 4 | positive |
| 248 | ENSG00000087191 | PSMC5 | 4 | positive |
| 249 | ENSG00000087586 | AURKA | 4 | positive |
| 250 | ENSG00000089006 | SNX5 | 4 | positive |
| 251 | ENSG00000089009 | RPL6 | 4 | positive |
| 252 | ENSG00000090263 | MRPS33 | 4 | positive |
| 253 | ENSG00000091483 | FH | 4 | positive |
| 254 | ENSG00000092201 | SUPT16H | 4 | positive |
| 255 | ENSG00000099797 | TECR | 4 | positive |
| 256 | ENSG00000100028 | SNRPD3 | 4 | positive |
| 257 | ENSG00000100353 | EIF3D | 4 | positive |
| 258 | ENSG00000100526 | CDKN3 | 4 | positive |
| 259 | ENSG00000100567 | PSMA3 | 4 | positive |
| 260 | ENSG00000100632 | ERH | 4 | positive |
| 261 | ENSG00000100714 | MTHFD1 | 4 | positive |
| 262 | ENSG00000101182 | PSMA7 | 4 | positive |
| 263 | ENSG00000101911 | PRPS2 | 4 | positive |
| 264 | ENSG00000102054 | RBBP7 | 4 | positive |
| 265 | ENSG00000106554 | CHCHD3 | 4 | positive |
| 266 | ENSG00000106628 | POLD2 | 4 | positive |
| 267 | ENSG00000107949 | BCCIP | 4 | positive |
| 268 | ENSG00000108298 | RPL19 | 4 | positive |
| 269 | ENSG00000108384 | RAD51C | 4 | positive |
| 270 | ENSG00000108953 | YWHAE | 4 | positive |

| No. | Ensemble ID | Symbol | No. dataset | Direction |
|---|---|---|---|---|
| 271 | ENSG00000110955 | ATP5F1B | 4 | positive |
| 272 | ENSG00000110958 | PTGES3 | 4 | positive |
| 273 | ENSG00000111206 | FOXM1 | 4 | positive |
| 274 | ENSG00000111678 | C12orf57 | 4 | positive |
| 275 | ENSG00000111775 | COX6A1 | 4 | positive |
| 276 | ENSG00000112306 | RPS12 | 4 | positive |
| 277 | ENSG00000113569 | NUP155 | 4 | positive |
| 278 | ENSG00000113648 | H2AFY | 4 | positive |
| 279 | ENSG00000115484 | CCT4 | 4 | positive |
| 280 | ENSG00000116459 | ATP5PB | 4 | positive |
| 281 | ENSG00000118181 | RPS25 | 4 | positive |
| 282 | ENSG00000120437 | ACAT2 | 4 | positive |
| 283 | ENSG00000120539 | MASTL | 4 | positive |
| 284 | ENSG00000121152 | NCAPH | 4 | positive |
| 285 | ENSG00000122545 | 7-Sep | 4 | positive |
| 286 | ENSG00000123473 | STIL | 4 | positive |
| 287 | ENSG00000126067 | PSMB2 | 4 | positive |
| 288 | ENSG00000129084 | PSMA1 | 4 | positive |
| 289 | ENSG00000129173 | E2F8 | 4 | positive |
| 290 | ENSG00000130520 | LSM4 | 4 | positive |
| 291 | ENSG00000130741 | EIF2S3 | 4 | positive |
| 292 | ENSG00000131470 | PSMC3IP | 4 | positive |
| 293 | ENSG00000132507 | EIF5A | 4 | positive |
| 294 | ENSG00000133119 | RFC3 | 4 | positive |
| 295 | ENSG00000134057 | CCNB1 | 4 | positive |
| 296 | ENSG00000134333 | LDHA | 4 | positive |
| 297 | ENSG00000134375 | TIMM17A | 4 | positive |
| 298 | ENSG00000135624 | CCT7 | 4 | positive |
| 299 | ENSG00000138668 | HNRNPD | 4 | positive |
| 300 | ENSG00000139180 | NDUFA9 | 4 | positive |
| 301 | ENSG00000139734 | DIAPH3 | 4 | positive |

| No. | Ensemble ID | Symbol | No. dataset | Direction |
|---|---|---|---|---|
| 302 | ENSG00000139921 | TMX1 | 4 | positive |
| 303 | ENSG00000140988 | RPS2 | 4 | positive |
| 304 | ENSG00000142945 | KIF2C | 4 | positive |
| 305 | ENSG00000143933 | CALM2 | 4 | positive |
| 306 | ENSG00000144580 | CNOT9 | 4 | positive |
| 307 | ENSG00000144713 | RPL32 | 4 | positive |
| 308 | ENSG00000145386 | CCNA2 | 4 | positive |
| 309 | ENSG00000145592 | RPL37 | 4 | positive |
| 310 | ENSG00000145741 | BTF3 | 4 | positive |
| 311 | ENSG00000145907 | G3BP1 | 4 | positive |
| 312 | ENSG00000146670 | CDCA5 | 4 | positive |
| 313 | ENSG00000147155 | EBP | 4 | positive |
| 314 | ENSG00000147669 | POLR2K | 4 | positive |
| 315 | ENSG00000149136 | SSRP1 | 4 | positive |
| 316 | ENSG00000149554 | CHEK1 | 4 | positive |
| 317 | ENSG00000152253 | SPC25 | 4 | positive |
| 318 | ENSG00000152795 | HNRNPDL | 4 | positive |
| 319 | ENSG00000156261 | CCT8 | 4 | positive |
| 320 | ENSG00000156482 | RPL30 | 4 | positive |
| 321 | ENSG00000156970 | BUB1B | 4 | positive |
| 322 | ENSG00000161057 | PSMC2 | 4 | positive |
| 323 | ENSG00000161888 | SPC24 | 4 | positive |
| 324 | ENSG00000162607 | USP1 | 4 | positive |
| 325 | ENSG00000163002 | NUP35 | 4 | positive |
| 326 | ENSG00000163808 | KIF15 | 4 | positive |
| 327 | ENSG00000163882 | POLR2H | 4 | positive |
| 328 | ENSG00000163923 | RPL39L | 4 | positive |
| 329 | ENSG00000164587 | RPS14 | 4 | positive |
| 330 | ENSG00000164902 | PHAX | 4 | positive |
| 331 | ENSG00000165480 | SKA3 | 4 | positive |
| 332 | ENSG00000165501 | LRR1 | 4 | positive |

| No. | Ensemble ID | Symbol | No. dataset | Direction |
|---|---|---|---|---|
| 333 | ENSG00000165609 | NUDT5 | 4 | positive |
| 334 | ENSG00000165678 | GHITM | 4 | positive |
| 335 | ENSG00000165916 | PSMC3 | 4 | positive |
| 336 | ENSG00000166801 | FAM111A | 4 | positive |
| 337 | ENSG00000167283 | ATP5MG | 4 | positive |
| 338 | ENSG00000167815 | PRDX2 | 4 | positive |
| 339 | ENSG00000168078 | PBK | 4 | positive |
| 340 | ENSG00000168090 | COPS6 | 4 | positive |
| 341 | ENSG00000169139 | UBE2V2 | 4 | positive |
| 342 | ENSG00000169567 | HINT1 | 4 | positive |
| 343 | ENSG00000169607 | CKAP2L | 4 | positive |
| 344 | ENSG00000169714 | CNBP | 4 | positive |
| 345 | ENSG00000170144 | HNRNPA3 | 4 | positive |
| 346 | ENSG00000171320 | ESCO2 | 4 | positive |
| 347 | ENSG00000171858 | RPS21 | 4 | positive |
| 348 | ENSG00000172757 | CFL1 | 4 | positive |
| 349 | ENSG00000172809 | RPL38 | 4 | positive |
| 350 | ENSG00000173418 | NAA20 | 4 | positive |
| 351 | ENSG00000175305 | CCNE2 | 4 | positive |
| 352 | ENSG00000176208 | ATAD5 | 4 | positive |
| 353 | ENSG00000176340 | COX8A | 4 | positive |
| 354 | ENSG00000178035 | IMPDH2 | 4 | positive |
| 355 | ENSG00000178999 | AURKB | 4 | positive |
| 356 | ENSG00000179750 | APOBEC3B | 4 | positive |
| 357 | ENSG00000184445 | KNTC1 | 4 | positive |
| 358 | ENSG00000184983 | NDUFA6 | 4 | positive |
| 359 | ENSG00000186468 | RPS23 | 4 | positive |
| 360 | ENSG00000186871 | ERCC6L | 4 | positive |
| 361 | ENSG00000188313 | PLSCR1 | 4 | positive |
| 362 | ENSG00000188846 | RPL14 | 4 | positive |
| 363 | ENSG00000189043 | NDUFA4 | 4 | positive |

| No. | Ensemble ID | Symbol | No. dataset | Direction |
|---|---|---|---|---|
| 364 | ENSG00000196419 | XRCC6 | 4 | positive |
| 365 | ENSG00000197299 | BLM | 4 | positive |
| 366 | ENSG00000197451 | HNRNPAB | 4 | positive |
| 367 | ENSG00000198554 | WDHD1 | 4 | positive |
| 368 | ENSG00000198826 | ARHGAP11A | 4 | positive |
| 369 | ENSG00000198918 | RPL39 | 4 | positive |
| 370 | ENSG00000204392 | LSM2 | 4 | positive |
| 371 | ENSG00000204628 | RACK1 | 4 | positive |
| 372 | ENSG00000213551 | DNAJC9 | 4 | positive |
| 373 | ENSG00000213585 | VDAC1 | 4 | positive |
| 374 | ENSG00000221983 | UBA52 | 4 | positive |
| 375 | ENSG00000241343 | RPL36A | 4 | positive |
| 376 | ENSG00000265241 | RBM8A | 4 | positive |
| 377 | ENSG00000277791 | PSMB3 | 4 | positive |
| 378 | ENSG00000100345 | MYH9 | 4 | negative |
| 379 | ENSG00000105664 | COMP | 4 | negative |
| 380 | ENSG00000109046 | WSB1 | 4 | negative |
| 381 | ENSG00000130066 | SAT1 | 4 | negative |
| 382 | ENSG00000130522 | JUND | 4 | negative |
| 383 | ENSG00000166340 | TPP1 | 4 | negative |
| 384 | ENSG00000172146 | OR1A1 | 4 | negative |
| 385 | ENSG00000180573 | HIST1H2AC | 4 | negative |
| 386 | ENSG00000187871 | GFRAL | 4 | negative |
| 387 | ENSG00000269028 | MTRNR2L12 | 4 | negative |
