# Supplementary material for: DNA damage repair-related methylated genes RRM2 and GAPDH are prognostic biomarkers associated with immunotherapy for lung adenocarcinoma
Source: Genet Mol Biol. 2025 May 9;48(2):e20240138. doi: 10.1590/1678-4685-GMB-2024-0138 (PMC12063672; doi:10.1590/1678-4685-GMB-2024-0138)
Supplement: Table S3 - [file 1415-4757-GMB-48-02-e20240138-s4.pdf]

Genetics and Molecular Biology
https://doi.org/10.1590/1678-4685-GMB-2024-0138

Supplementary Material to "DNA damage repair-related methylated genes RRM2 and GAPDH are prognostic biomarkers associated with immunotherapy for lung adenocarcinoma"

Table S3 - 295 DNA damage & repair-related genes from MSigDB.

GENE	GENE	GENE	GENE	GENE	GENE	GENE	GENE
53BP1	DCLRE1C	FANCG	MAD2L2	NUDT18	POLN	RIF1	TOP3A
ABH2	DDB1	FANCI	MBD4	OGG1	POLQ	RMI1	TOPBP1
ABRAXAS1	DDB2	FANCJ	MDC1	p53R2	POLZ	RNF168	TP53
ADPRT	DEPC1	FANCL	METNASE	PALB2	PRIMPOL	RNF4	TP53BP1
ADPRTL2	DINB1	FANCM	MGMT	PARG	PRKDC	RNF8	TREX1
ADPRTL3	DJ-1	FANCN	MLH1	PARK7	PRPF19	RPA1	TREX2
ALKBH2	DMC1	FANCO	MLH3	PARP1	PSO4	RPA2	TTDA
ALKBH3	DNA2	FANCP	MMS19	PARP2	PTIP	RPA3	TTDN1
APE1	DNase	FANCT	MMS2	PARP3	RAD1	RPA4	TTRAP
APEX1	DNPH1	FEN1	MMS4L	PARPBP	RAD17	RRM2B	UBC13
APEX2	DNTT	GEN1	MNAT1	PAXIP1	RAD18	SEM1	UBE2A
APLF	DSS1	GIYD1	MPG	PCNA	RAD23A	SETMAR	UBE2B
APTX	DUT	GIYD2	MPLKIP	PDS5B	RAD23B	SHFM1	UBE2N
ATM	EME1	GTF2E2	MRE11A	PER1	RAD24	SHLD1	UBE2T
ATR	EME2	GTF2H1	MSH2	PMS1	RAD30B	SHLD2	UBE2V2
ATRIP	ENDOV	GTF2H2	MSH3	PMS2	RAD50	SHLD3	UNG
ATRX	ERCC1	GTF2H3	MSH4	PMS2L3	RAD51	SHPRH	USP1
BARD1	ERCC2	GTF2H4	MSH5	PMS2P3	RAD51B	SLX1A	UVSSA
BLM	ERCC3	GTF2H5	MSH6	PNKP	RAD51C	SLX1B	WDR48
BRCA1	ERCC4	H2AFX	MTH1	POL4P	RAD51D	SLX4	WRN
BRCA2	ERCC5	H2AX	MTH2	POLA1	RAD52	SMARCA3	XAB2
BRIP1	ERCC6	HCNP	MTH3	POLB	RAD52B	SMC5	XPA
C19orf40	ERCC8	HEL308	MTMR15	POLD1	RAD54B	SMC6	XPB
C1orf86	EXO1	HELQ	MUS81	POLD2	RAD54L	SMUG1	XPC
CAF1	EXO5	HERC2	MUTYH	POLD3	RAD6A	SNM1A	XPD
CCNH	FAAP100	HEX1	MYH	POLD4	RAD6B	SNM1B	XPE
CDK7	FAAP20	HFM1	NABP2	POLE	RAD9A	SPIDR	XPF
CETN2	FAAP24	HLTF	NBN	POLE1	RBBP8	SPO11	XPG

GENE	GENE	GENE	GENE	GENE	GENE	GENE	GENE
CHAF1A	FAM35A	HMCES	NBS1	POLE2	RDM1	SPRTN	XRCC1
CHEK1	FAN1	HUS1	NEIL1	POLE3	RECQ1	SSB1	XRCC2
CHEK2	FANCA	KIAA1530	NEIL2	POLE4	RECQL	SWI5	XRCC3
CLK2	FANCB	KIAA1794	NEIL3	POLG	RECQL4	SWS1	XRCC4
CSA	FANCC	Ku70	NHEJ1	POLH	RECQL5	SWSAP1	XRCC5
CSB	FANCD1	Ku80	NTH1	POLI	REV1	TDG	XRCC6
CtIP	FANCD2	LIG1	NTHL1	POLK	REV1L	TDP1	XRCC9
DCLRE1A	FANCE	LIG3	NUDT1	POLL	REV3L	TDP2	ZSWIM7
DCLRE1B	FANCF	LIG4	NUDT15	POLM	REV7	TFIIH	
