# Supplementary material for: DNA damage repair-related methylated genes RRM2 and GAPDH are prognostic biomarkers associated with immunotherapy for lung adenocarcinoma
Source: Genet Mol Biol. 2025 May 9;48(2):e20240138. doi: 10.1590/1678-4685-GMB-2024-0138 (PMC12063672; doi:10.1590/1678-4685-GMB-2024-0138)
Supplement: Table S4 - [file 1415-4757-GMB-48-02-e20240138-s5.pdf]

Genetics and Molecular Biology
https://doi.org/10.1590/1678-4685-GMB-2024-0138

Supplementary Material to "DNA damage repair-related methylated genes RRM2 and GAPDH are prognostic biomarkers associated with immunotherapy for lung adenocarcinoma"

Table S4 - 696 DDR-related genes (DDRGs; de-duplicated genes;) from the CancerSEA database and MSigDB database.

GENE	GENE	GENE	GENE	GENE	GENE	GENE
ASPM	DTYMK	RPL32	RPS24	DNAJC9	GTF2E2	PRPF19
NUF2	BUB1	CDCA5	SRP9	VDAC1	GTF2H1	PSO4
GTSE1	SHCBP1	SSRP1	CDCA7	RPL36A	GTF2H2	PTIP
CDCA8	RPS21	SPC25	NHP2	PSMB3	GTF2H3	RAD1
RPL3	CKS1B	ATP5MC3	MMS22L	MYH9	GTF2H4	RAD17
RPS16	IQGAP3	BUB1B	NDUFB6	COMP	GTF2H5	RAD18
KIF23	RPS20	SPC24	HNRNPF	WSB1	H2AFX	RAD23A
TPRKB	MCM10	RPL39L	MINOS1	SAT1	H2AX	RAD23B
RPL10	NUCKS1	FAM111A	RPL15	JUND	HCNP	RAD24
ATP5F1A	SPAG5	PCLAF	TOMM5	TPP1	HEL308	RAD30B
RAD21	KIF22	RPSA	ALYREF	OR1A1	HELQ	RAD50
DBF4	NDC80	CKAP2L	FAM111B	HIST1H2AC	HERC2	RAD51
MDH1	RPS5	HNRNPA3	NACA	GFRAL	HEX1	RAD51B
KIF4A	ORC1	PA2G4	MRPL42	MTRNR2L12	HFM1	RAD51D
TFAM	AURKA	LSM3	ATP5MF	DUT	HLTF	RAD52
NSD2	CDC6	ESCO2	HLA-A	PARK7	HMCES	RAD52B
PHF19	RANBP1	ATAD5	CYP51A1	RPA2	HUS1	RAD54B
MND1	CMC2	AURKB	PSMC4	BARD1	KIAA1530	RAD54L
KIF18A	RNASEH2A	RPS17	PSMA4	EXO1	KIAA1794	RAD6A
PRMT1	H2AFV	RPS23	ENO1	POLD2	Ku70	RAD6B
PIMREG	LSM5	SUMO2	HSP90AA1	RAD51C	Ku80	RAD9A
RPS15A	NCAPG	RPL14	EIF3I	UBE2V2	LIG1	RBBP8
RPL11	PTGES3	PPIA	PSMC5	XRCC6	LIG3	RDM1
PLK4	TIMELESS	HNRNPAB	SNX5	53BP1	LIG4	RECQ1
DCK	SRSF3	PRIM1	MRPS33	ABH2	MAD2L2	RECQL
RPL26	MCM3	ARHGAP11A	FH	ABRAXAS1	MBD4	RECQL4
CCNF	H2AFY	UBA52	SUPT16H	ADPRT	MDC1	RECQL5

GENE	GENE	GENE	GENE	GENE	GENE	GENE
RBBP4	ECT2	RPS18	TECR	ADPRTL2	METNASE	REV1
PSIP1	SRSF7	NME1	SNRPD3	ADPRTL3	MGMT	REV1L
RPL27A	CACYBP	RBM8A	EIF3D	ALKBH2	MLH1	REV3L
PLK1	EXOSC8	PCNA	PSMA3	ALKBH3	MLH3	REV7
ARL6IP1	NCAPH	UBE2T	ERH	APE1	MMS19	RIF1
CKAP5	CENPK	CHEK1	MTHFD1	APEX1	MMS2	RMI1
RPL10A	HNRNPR	FEN1	PSMA7	APEX2	MMS4L	RNF168
CST3	TUBG1	UBE2N	PRPS2	APLF	MNAT1	RNF4
CLU	RPL27	BRCA2	RBBP7	APTX	MPG	RNF8
STMN1	NASP	FANCI	CHCHD3	ATM	MPLKIP	RPA1
MELK	CCNB1	USP1	BCCIP	ATR	MRE11A	RPA4
CDK1	RPS6	BRCA1	YWHAE	ATRIP	MSH2	RRM2B
TYMS	DIAPH3	RPA3	ATP5F1B	ATRX	MSH3	SEM1
DHFR	RPS8	XRCC5	C12orf57	BRIP1	MSH4	SETMAR
TMPO	ANP32E	FANCD2	COX6A1	C19orf40	MSH5	SHFM1
TUBA1B	DTL	BLM	RPS12	C1orf86	MSH6	SHLD1
HNRNPA1	CALM2	PAICS	NUP155	CAF1	MTH1	SHLD2
H2AFZ	SNRPG	GGCT	CCT4	CCNH	MTH2	SHLD3
SNRPD1	HSPD1	MCM5	ATP5PB	CDK7	MTH3	SHPRH
UBE2C	RPS3A	RAD51AP1	RPS25	CETN2	MTMR15	SLX1A
HMGB1	RBMX	TMEM106C	ACAT2	CHAF1A	MUS81	SLX1B
TUBB	RPL7	CCT6A	MASTL	CHEK2	MUTYH	SLX4
HMGN2	EIF3M	MCM2	7-Sep	CLK2	MYH	SMARCA3
TPX2	CCT5	WDR76	STIL	CSA	NABP2	SMC5
CENPF	BUB3	LDHB	PSMB2	CSB	NBN	SMC6
CKS2	ATAD2	CCT3	PSMA1	CtIP	NBS1	SMUG1
TOP2A	CCNB2	SNRPE	LSM4	DCLRE1A	NEIL1	SNM1A
NUSAP1	RPL8	E2F2	EIF2S3	DCLRE1B	NEIL2	SNM1B
RRM1	KIF15	SMC1A	PSMC3IP	DCLRE1C	NEIL3	SPIDR
RRM2	PTTG1	MCM6	EIF5A	DDB1	NHEJ1	SPO11
NPM1	SKA3	PEBP1	RFC3	DDB2	NTH1	SPRTN
PRC1	PSMC3	CENPM	LDHA	DEPC1	NTHL1	SSB1
ANLN	C19orf48	PSMB5	TIMM17A	DINB1	NUDT1	SWI5
CSE1L	PBK	PDCD5	CCT7	DJ-1	NUDT15	SWS1
DLGAP5	RPS7	SSBP1	HNRNPD	DMC1	NUDT18	SWSAP1
RAN	CYCS	TMEM97	NDUFA9	DNA2	OGG1	TDG
SMC2	RPL4	RFC5	RPS2	DNase	p53R2	TDP1
KIF11	CCNE2	TPI1	CNOT9	DNPH1	PALB2	TDP2
ILF2	APOBEC3B	RPS15	RPL37	DNTT	PARG	TFIIH
CENPU	KPNA2	HSPE1	BTF3	DSS1	PARP1	TOP3A

GENE	GENE	GENE	GENE	GENE	GENE	GENE
HMGB2	SKA2	RPL5	G3BP1	EME1	PARP2	TOPBP1
MAD2L1	HIST1H4C	CBX3	EBP	EME2	PARP3	TP53
TK1	KIFC1	SNRPC	POLR2K	ENDOV	PARPBP	TP53BP1
PTMA	UHRF1	EIF2S1	HNRNPDL	ERCC1	PAXIP1	TREX1
CENPW	DEPDC1	RPL35	CCT8	ERCC2	PDS5B	TREX2
SLC25A5	RFC2	PSMA5	RPL30	ERCC3	PER1	TTDA
NCAPD2	TRIP13	NCAPG2	PSMC2	ERCC4	PMS1	TTDN1
RPLP0	RPL6	DSN1	NUP35	ERCC5	PMS2	TTRAP
BIRC5	ORC6	CENPH	POLR2H	ERCC6	PMS2L3	UBC13
CDC45	CLSPN	FDPS	RPS14	ERCC8	PMS2P3	UBE2A
CBX5	CDKN3	PRDX3	PHAX	EXO5	PNKP	UBE2B
MCM4	VRK1	CCT2	LRR1	FAAP100	POL4P	UNG
ASF1B	RPS19	BANF1	NUDT5	FAAP20	POLA1	UVSSA
RPL18A	CASP2	RPS4X	GHITM	FAAP24	POLB	WDR48
HSPA8	SMC3	SRI	ATP5MG	FAM35A	POLD1	WRN
FOXM1	RPL19	HNRNPC	PRDX2	FAN1	POLD3	XAB2
GMNN	RPS13	PSME2	COPS6	FANCA	POLD4	XPA
TTK	VPS29	GINS1	HINT1	FANCB	POLE	XPB
SMC4	KIF14	CYB5B	CNBP	FANCC	POLE1	XPC
NCL	SET	CHCHD2	CFL1	FANCD1	POLE2	XPD
HNRNPA2B1	HELLS	EZH2	RPL38	FANCE	POLE3	XPE
ZWINT	HJURP	PSMA2	NAA20	FANCF	POLE4	XPF
DEK	GLO1	MTCH2	COX8A	FANCG	POLG	XPG
SNRPB	SNRPD2	GAPDH	IMPDH2	FANCJ	POLH	XRCC1
HAT1	E2F8	BRIX1	KNTC1	FANCL	POLI	XRCC2
GINS2	DNMT1	EEF1B2	NDUFA6	FANCM	POLK	XRCC3
KIF2C	GGH	FARSB	ERCC6L	FANCN	POLL	XRCC4
CCNA2	KIF20B	RPL21	PLSCR1	FANCO	POLM	XRCC9
MKI67	SNRPF	RPS10	NDUFA4	FANCP	POLN	ZSWIM7
RPS3	TMX1	RPL23	WDHD1	FANCT	POLQ	
RFC4	SAE1	CDK4	RPL39	GEN1	POLZ	
CENPN	RPS11	ACTL6A	LSM2	GIYD1	PRIMPOL	
MCM7	RPS27A	TCF19	RACK1	GIYD2	PRKDC	
