# Supplementary material for: DNA damage repair-related methylated genes RRM2 and GAPDH are prognostic biomarkers associated with immunotherapy for lung adenocarcinoma
Source: Genet Mol Biol. 2025 May 9;48(2):e20240138. doi: 10.1590/1678-4685-GMB-2024-0138 (PMC12063672; doi:10.1590/1678-4685-GMB-2024-0138)
Supplement: Table S5 - [file 1415-4757-GMB-48-02-e20240138-s6.pdf]

Genetics and Molecular Biology
https://doi.org/10.1590/1678-4685-GMB-2024-0138

Supplementary Material to "DNA damage repair-related methylated genes RRM2 and GAPDH are prognostic biomarkers associated with immunotherapy for lung adenocarcinoma"

Table S5 - Differentially expressed genes (DEGs) in lung adenocarcinoma (LUAD) including up-regulated and down-regulated genes.

Gene	logFC	AveExpr	t	P.Value	adj.P.Val	B
LGI3	-3.72111	2.525461	-19.1213	5.00E-21	6.42E-17	37.50719
EMP2	-2.68958	6.08264	-17.1001	2.22E-19	1.43E-15	33.89347
TNNC1	-3.97098	3.884339	-15.9866	2.10E-18	8.96E-15	31.73639
FABP4	-4.68219	3.804835	-15.3895	7.33E-18	2.09E-14	30.52681
CLEC3B	-4.20182	4.644114	-15.3408	8.13E-18	2.09E-14	30.42646
ACADL	-2.27905	1.751335	-14.9711	1.80E-17	3.85E-14	29.65615
ALDH18A1	1.946362	4.229169	14.58366	4.20E-17	7.70E-14	28.83228
RGCC	-2.88848	6.843124	-14.444	5.73E-17	8.87E-14	28.53119
SFXN1	1.501722	2.293601	14.40702	6.22E-17	8.87E-14	28.45098
CAV1	-3.43014	6.679305	-14.2997	7.91E-17	9.36E-14	28.21745
CLIC5	-3.71263	3.654256	-14.2529	8.78E-17	9.36E-14	28.11527
RAMP2	-3.00178	4.736683	-14.2388	9.07E-17	9.36E-14	28.08448
GPIHBP1	-3.3627	2.79287	-14.2189	9.48E-17	9.36E-14	28.04099
UPK3B	-4.67989	3.617371	-14.1766	1.04E-16	9.56E-14	27.94807
ITLN2	-3.82961	2.358108	-14.1098	1.21E-16	1.04E-13	27.80108
ACVRL1	-2.25028	4.082189	-14.0457	1.40E-16	1.12E-13	27.65961
RALGPS2	1.635123	1.783968	13.73763	2.84E-16	2.06E-13	26.97274
STX11	-2.759	3.567355	-13.7294	2.89E-16	2.06E-13	26.95428
CA4	-3.50725	2.399541	-13.5568	4.31E-16	2.91E-13	26.56423
LIMS2	-2.4438	2.454016	-13.4225	5.89E-16	3.78E-13	26.25828
GPT2	2.255107	2.041829	13.37304	6.62E-16	3.92E-13	26.14492
KANK3	-2.16866	2.090539	-13.3665	6.72E-16	3.92E-13	26.12994
AGER	-6.24511	6.842546	-13.1456	1.13E-15	6.31E-13	25.62017
PYCR1	3.313118	3.481443	13.02666	1.50E-15	8.03E-13	25.34321
PECAM1	-2.10703	5.820197	-12.9726	1.71E-15	8.77E-13	25.21679
ADRB2	-2.54095	2.481984	-12.9495	1.81E-15	8.91E-13	25.16263
CDH5	-2.38065	4.340904	-12.919	1.94E-15	9.08E-13	25.09097

Gene	logFC	AveExpr	t	P.Value	adj.P.Val	B
PDLIM2	-1.59263	2.133347	-12.9107	1.98E-15	9.08E-13	25.07154
S1PR1	-2.47391	4.670069	-12.8415	2.34E-15	1.03E-12	24.90839
WNT3A	-2.25356	1.347418	-12.8279	2.42E-15	1.03E-12	24.87643
ROBO4	-2.58663	3.157643	-12.8073	2.54E-15	1.05E-12	24.82759
FHL1	-3.23586	4.207137	-12.6415	3.80E-15	1.52E-12	24.43398
CD36	-2.7052	3.06958	-12.58	4.41E-15	1.70E-12	24.2871
SH2D3C	-2.02493	3.100349	-12.5714	4.50E-15	1.70E-12	24.26654
PLA2G1B	-3.38655	3.159921	-12.476	5.69E-15	2.08E-12	24.03739
BTNL9	-2.67898	2.095267	-12.41	6.69E-15	2.38E-12	23.87846
LIN7A	-1.49122	1.434333	-12.3749	7.29E-15	2.53E-12	23.79347
CRTAC1	-3.10678	3.946636	-12.3537	7.68E-15	2.53E-12	23.74221
SPOCK2	-3.30103	4.909279	-12.3527	7.70E-15	2.53E-12	23.73973
EDNRB	-2.94848	3.680396	-12.3049	8.67E-15	2.78E-12	23.62388
PRX	-2.89759	2.94415	-12.1894	1.15E-14	3.56E-12	23.34247
ECSCR	-1.71959	2.046569	-12.1859	1.16E-14	3.56E-12	23.33384
CAV2	-2.11669	4.885526	-11.9744	1.98E-14	5.90E-12	22.81388
HIGD1B	-2.44533	2.836377	-11.9461	2.12E-14	6.19E-12	22.744
FAM107A	-4.15927	3.682574	-11.8437	2.75E-14	7.83E-12	22.48978
COX4I2	-2.2798	3.480904	-11.7559	3.43E-14	9.57E-12	22.27063
GRK5	-2.17145	2.995259	-11.7393	3.58E-14	9.77E-12	22.22916
EPAS1	-2.74048	6.981237	-11.7307	3.66E-14	9.78E-12	22.20756
SGCA	-1.63643	2.024247	-11.7082	3.88E-14	1.01E-11	22.15114
CLIC3	-2.62056	4.356076	-11.6704	4.27E-14	1.10E-11	22.05625
RTKN2	-3.39006	2.773813	-11.6172	4.89E-14	1.23E-11	21.92258
IARS	1.161652	3.914504	11.59353	5.20E-14	1.28E-11	21.86279
ZC3HAV1L	1.163487	1.516534	11.54048	5.95E-14	1.44E-11	21.72878
STK39	1.414744	3.211604	11.52657	6.17E-14	1.45E-11	21.69357
PHKA1	1.344709	1.822375	11.52276	6.23E-14	1.45E-11	21.68395
RCC1	1.516826	3.212086	11.5076	6.48E-14	1.48E-11	21.64554
TEK	-2.56204	3.178784	-11.4717	7.10E-14	1.60E-11	21.55455
ADAMTS8	-2.72339	2.337499	-11.4542	7.43E-14	1.64E-11	21.50991
PLA2G4F	-2.75497	2.684087	-11.4347	7.82E-14	1.70E-11	21.46048
NCKAP5	-2.25228	1.766182	-11.409	8.35E-14	1.75E-11	21.39508
EMCN	-2.25475	2.858088	-11.4077	8.38E-14	1.75E-11	21.39175
PPAP2C	2.167747	2.243453	11.40369	8.47E-14	1.75E-11	21.38148
CRYAB	-2.01884	2.766362	-11.3842	8.91E-14	1.81E-11	21.33187
RAMP3	-3.17803	4.564289	-11.3739	9.15E-14	1.83E-11	21.30555
ESAM	-2.32264	4.746037	-11.3506	9.72E-14	1.92E-11	21.24594
DPEP2	-1.73716	2.014636	-11.3257	1.04E-13	2.02E-11	21.18226
DARS2	1.397117	2.979856	11.30572	1.09E-13	2.09E-11	21.1312

Gene	logFC	AveExpr	t	P.Value	adj.P.Val	B
FAM189A2	-2.72959	2.966862	-11.2521	1.26E-13	2.37E-11	20.99371
JAM2	-1.86104	2.540828	-11.1646	1.58E-13	2.91E-11	20.76851
SGCG	-1.7697	1.204274	-11.162	1.59E-13	2.91E-11	20.7616
AOC3	-2.45311	4.923394	-11.1566	1.61E-13	2.91E-11	20.74784
TAL1	-1.40525	1.241992	-11.1467	1.65E-13	2.95E-11	20.72222
SLC39A8	-2.56398	5.328591	-11.129	1.73E-13	3.04E-11	20.6765
TRAP1	1.152681	3.110798	11.10392	1.85E-13	3.21E-11	20.61157
PDIA4	1.959561	6.281949	11.09406	1.90E-13	3.25E-11	20.58605
MYCT1	-1.7964	2.792177	-10.9905	2.49E-13	4.19E-11	20.31706
CSTF2	1.141509	3.024767	10.98635	2.52E-13	4.19E-11	20.3063
TMEM88	-2.32687	2.353084	-10.9822	2.55E-13	4.19E-11	20.29552
PHACTR1	-1.79138	2.006231	-10.9735	2.61E-13	4.23E-11	20.2727
SCUBE1	-1.79878	1.179643	-10.962	2.69E-13	4.31E-11	20.24283
CLDN5	-2.77082	4.030344	-10.8259	3.86E-13	6.11E-11	19.88673
STXBP6	-2.13592	1.777541	-10.8207	3.91E-13	6.12E-11	19.87305
PPAT	1.404445	1.667859	10.81188	4.00E-13	6.19E-11	19.84984
RASIP1	-2.00422	2.727605	-10.8052	4.07E-13	6.22E-11	19.8322
SRPK1	1.619622	3.228009	10.79068	4.24E-13	6.39E-11	19.79411
SLC6A4	-3.90047	2.315907	-10.7474	4.75E-13	7.06E-11	19.67999
EPRS	1.31533	4.744731	10.74459	4.79E-13	7.06E-11	19.6727
PLA2G3	-1.10393	0.93433	-10.6882	5.57E-13	8.11E-11	19.52371
HN1L	1.385002	4.582013	10.68313	5.65E-13	8.11E-11	19.51037
SBSPON	-1.72632	1.447984	-10.6788	5.71E-13	8.11E-11	19.49879
FAM46B	-2.21692	3.032094	-10.6732	5.80E-13	8.11E-11	19.48414
GPM6B	-1.52933	1.939422	-10.6719	5.82E-13	8.11E-11	19.48067
ACE	-1.69144	3.155522	-10.6644	5.94E-13	8.19E-11	19.4607
LGR4	2.622801	2.584573	10.64345	6.28E-13	8.57E-11	19.40527
GPD1	-2.93474	2.377653	-10.6372	6.39E-13	8.62E-11	19.3887
GLIPR2	-2.12206	4.472942	-10.6191	6.70E-13	8.96E-11	19.34076
PEBP4	-3.83789	4.54194	-10.6023	7.01E-13	9.18E-11	19.29607
LPL	-2.72255	4.422168	-10.6023	7.01E-13	9.18E-11	19.296
DES	-3.19186	3.397381	-10.5452	8.18E-13	1.06E-10	19.14404
LAMP3	-3.25812	5.921421	-10.5129	8.93E-13	1.15E-10	19.05799
PLAC9	-2.3685	3.158942	-10.4843	9.64E-13	1.22E-10	18.9818
TIE1	-1.61503	3.166882	-10.4818	9.71E-13	1.22E-10	18.97488
LMAN1	1.111781	4.859324	10.4696	1.00E-12	1.25E-10	18.94236
KCNN4	2.789215	2.620022	10.45896	1.03E-12	1.27E-10	18.9139
SLC35F2	1.921276	2.889043	10.45082	1.06E-12	1.29E-10	18.89211
NPR1	-2.42383	2.959879	-10.4235	1.14E-12	1.38E-10	18.81884
PTRF	-1.90413	6.740726	-10.41	1.18E-12	1.41E-10	18.78261

Gene	logFC	AveExpr	t	P.Value	adj.P.Val	B
P3H2	-2.15515	3.478519	-10.4037	1.20E-12	1.42E-10	18.7659
SLC46A2	-2.09412	2.156451	-10.3965	1.22E-12	1.44E-10	18.74655
PAK1	1.333654	3.373112	10.39302	1.23E-12	1.44E-10	18.73717
GYPC	-1.4948	3.535162	-10.3703	1.31E-12	1.52E-10	18.67608
TMEM177	1.055061	1.715101	10.33973	1.43E-12	1.63E-10	18.5939
SLC2A1	3.616154	3.641874	10.33689	1.44E-12	1.63E-10	18.58625
C11orf80	1.074235	2.062632	10.32518	1.49E-12	1.67E-10	18.55469
HSPA12B	-2.1189	2.559481	-10.2984	1.60E-12	1.78E-10	18.48261
CLEC1A	-1.42835	1.633718	-10.2744	1.71E-12	1.89E-10	18.41763
SPAG4	1.64163	1.628954	10.26582	1.75E-12	1.92E-10	18.39455
ADPRH	-1.27108	2.762657	-10.258	1.78E-12	1.94E-10	18.37341
FGFR4	-2.31393	2.871608	-10.2414	1.87E-12	2.00E-10	18.32862
NECAB1	-1.62141	1.462283	-10.2406	1.87E-12	2.00E-10	18.32627
FIGF	-3.22767	3.025408	-10.2386	1.88E-12	2.00E-10	18.32094
AFF3	-1.44617	1.244918	-10.2309	1.92E-12	2.02E-10	18.30023
DAPK2	-1.63081	1.791416	-10.2267	1.94E-12	2.03E-10	18.28860
CACNA2D2	-3.40759	3.974928	-10.2195	1.98E-12	2.05E-10	18.26933
LRRC36	-2.10528	1.695134	-10.2029	2.08E-12	2.13E-10	18.22425
INMT	-3.40581	4.528404	-10.1896	2.15E-12	2.19E-10	18.18818
CFP	-1.2817	1.486608	-10.1673	2.29E-12	2.31E-10	18.12766
FMO2	-2.68948	4.009353	-10.1534	2.38E-12	2.38E-10	18.09004
GOLM1	2.707609	4.762247	10.13778	2.48E-12	2.47E-10	18.04745
RFWD3	1.195187	2.561827	10.11654	2.63E-12	2.60E-10	17.98967
HIST1H2BD	3.07023	3.537229	10.09574	2.79E-12	2.73E-10	17.93302
UBFD1	1.158122	2.870898	10.06111	3.06E-12	2.98E-10	17.83857
RADIL	-1.14717	0.964132	-10.012	3.51E-12	3.39E-10	17.70443
CAT	-1.76815	5.773947	-9.97751	3.86E-12	3.68E-10	17.60989
MFNG	-1.50995	3.16197	-9.97615	3.88E-12	3.68E-10	17.60616
GAS6	-1.83083	4.805803	-9.96969	3.95E-12	3.72E-10	17.58843
CCT3	1.464235	5.713631	9.949694	4.17E-12	3.91E-10	17.53359
DIEXF	1.079089	1.850829	9.913978	4.61E-12	4.28E-10	17.43547
SDPR	-2.95383	5.522717	-9.91068	4.65E-12	4.28E-10	17.42641
TOP2A	3.081702	2.598512	9.909241	4.67E-12	4.28E-10	17.42245
SGPL1	1.136078	3.591056	9.906489	4.70E-12	4.28E-10	17.41488
CLNS1A	1.023557	3.745385	9.897217	4.83E-12	4.36E-10	17.38937
FCN3	-4.39411	3.831929	-9.86362	5.30E-12	4.75E-10	17.29684
KL	-1.58493	1.472184	-9.85672	5.40E-12	4.81E-10	17.27782
HSPD1	1.270585	5.608728	9.845392	5.58E-12	4.93E-10	17.24658
FERMT1	2.125173	1.616667	9.823957	5.92E-12	5.17E-10	17.18741
GDF10	-2.4895	2.249596	-9.81264	6.11E-12	5.30E-10	17.15615

Gene	logFC	AveExpr	t	P.Value	adj.P.Val	B
TFB2M	1.263	3.319643	9.794639	6.43E-12	5.53E-10	17.10639
IARS2	1.198197	4.94484	9.789496	6.52E-12	5.55E-10	17.09217
LDB2	-2.04439	3.126221	-9.78883	6.53E-12	5.55E-10	17.09034
STARD8	-1.47752	2.578581	-9.77312	6.82E-12	5.76E-10	17.04686
TMEM204	-1.62758	4.073938	-9.771	6.86E-12	5.76E-10	17.04097
RMI2	1.481705	1.701147	9.767056	6.94E-12	5.78E-10	17.03006
KCNK3	-2.43026	2.242987	-9.73793	7.53E-12	6.23E-10	16.94935
PC	1.303041	2.087074	9.73233	7.65E-12	6.29E-10	16.93382
ANKRD22	2.629218	2.463858	9.718972	7.94E-12	6.48E-10	16.89675
PTCRA	-1.15121	0.885531	-9.69649	8.46E-12	6.82E-10	16.83431
SLC39A11	1.501207	3.198723	9.688195	8.66E-12	6.92E-10	16.81127
GYPE	-1.15838	0.870004	-9.68734	8.68E-12	6.92E-10	16.8089
PRPS2	1.022694	4.049595	9.669061	9.14E-12	7.23E-10	16.75805
WNT7A	-1.56266	1.219596	-9.66043	9.36E-12	7.37E-10	16.73402
ARHGAP6	-1.19071	1.288021	-9.65272	9.57E-12	7.48E-10	16.71256
CASKIN2	-1.64548	3.503175	-9.6424	9.85E-12	7.66E-10	16.68382
E2F8	1.253568	0.836145	9.638014	9.97E-12	7.70E-10	16.67161
ARHGEF15	-1.9614	2.3294	-9.62456	1.04E-11	7.95E-10	16.63411
KLK10	-2.04354	1.741372	-9.62059	1.05E-11	7.97E-10	16.62303
PGM2L1	1.57659	1.946237	9.619444	1.05E-11	7.97E-10	16.61984
STT3A	1.038322	4.439096	9.615737	1.06E-11	8.01E-10	16.60951
ZBTB41	1.198198	2.341773	9.612761	1.07E-11	8.03E-10	16.60120
HSPB8	-2.16201	4.144685	-9.60897	1.08E-11	8.07E-10	16.59064
HBA2	-4.31219	5.168203	-9.59315	1.13E-11	8.37E-10	16.54647
NUP155	1.383363	2.405714	9.591813	1.14E-11	8.37E-10	16.54274
CLDN18	-5.42904	5.376964	-9.5822	1.17E-11	8.55E-10	16.51588
SLC25A13	1.146085	3.035498	9.572052	1.20E-11	8.75E-10	16.48753
HYOU1	1.47677	4.743395	9.551323	1.27E-11	9.18E-10	16.42956
LHFP	-1.60163	4.499789	-9.54758	1.29E-11	9.18E-10	16.41909
WWC2	-1.74057	3.16559	-9.54713	1.29E-11	9.18E-10	16.41783
PTPN21	-1.8256	2.849534	-9.537	1.33E-11	9.40E-10	16.38947
SLFN13	1.407121	1.762907	9.533653	1.34E-11	9.41E-10	16.3801
UBE2T	2.631959	2.561614	9.529265	1.36E-11	9.41E-10	16.36781
ERG	-1.6384	2.543113	-9.49849	1.48E-11	1.02E-09	16.28157
MYO19	1.01966	1.547587	9.485649	1.53E-11	1.05E-09	16.24553
CNTN6	-1.61522	1.169073	-9.4603	1.65E-11	1.11E-09	16.17434
ST8SIA6	-1.73046	1.339761	-9.45754	1.66E-11	1.12E-09	16.16658
TMOD1	-1.26583	1.454987	-9.43948	1.75E-11	1.17E-09	16.11583
DUOX1	-2.58479	3.451498	-9.43092	1.79E-11	1.19E-09	16.09174
FEZ1	-1.29212	1.837799	-9.42962	1.80E-11	1.19E-09	16.08808

Gene	logFC	AveExpr	t	P.Value	adj.P.Val	B
GKN2	-3.56458	3.176417	-9.42068	1.84E-11	1.21E-09	16.06292
SIRPB1	-1.71244	1.741813	-9.41832	1.86E-11	1.21E-09	16.05629
HSPB7	-1.11765	1.409472	-9.41551	1.87E-11	1.22E-09	16.04837
NES	-1.90713	3.790116	-9.3993	1.96E-11	1.26E-09	16.00269
H1F0	1.248872	6.436931	9.396758	1.97E-11	1.26E-09	15.99554
FBLN5	-2.13229	4.01403	-9.39434	1.99E-11	1.26E-09	15.98873
LYVE1	-2.50471	2.997022	-9.36575	2.16E-11	1.36E-09	15.90810
HYAL2	-1.29733	4.618751	-9.34754	2.27E-11	1.43E-09	15.85669
PPP1R14B	1.826165	4.602424	9.343567	2.30E-11	1.44E-09	15.84546
CPB2	-3.38399	2.774419	-9.34124	2.31E-11	1.44E-09	15.83888
TMEM100	-3.60285	3.476582	-9.33439	2.36E-11	1.46E-09	15.81952
DUOXA1	-1.98726	2.928829	-9.33093	2.38E-11	1.47E-09	15.80975
TRPV2	-1.54295	3.862937	-9.3252	2.42E-11	1.48E-09	15.79355
PROM2	2.463104	2.705355	9.323194	2.43E-11	1.48E-09	15.78788
GRASP	-1.97465	2.972807	-9.32254	2.44E-11	1.48E-09	15.78602
COPA	1.095084	5.681316	9.289459	2.68E-11	1.61E-09	15.69241
SEMA3G	-2.29527	2.488047	-9.27303	2.81E-11	1.68E-09	15.64586
MCEMP1	-3.72158	4.029211	-9.21872	3.28E-11	1.96E-09	15.49176
RASAL1	1.430386	0.845099	9.213551	3.33E-11	1.98E-09	15.47706
VIPR1	-2.65164	2.7013	-9.20547	3.41E-11	2.01E-09	15.45410
LRRC32	-2.20513	4.142418	-9.20448	3.42E-11	2.01E-09	15.45126
ITIH5	-1.53268	1.913855	-9.17812	3.69E-11	2.16E-09	15.37629
FANCG	1.009091	2.010055	9.16848	3.79E-11	2.21E-09	15.34884
KIF11	1.960539	1.756025	9.165848	3.82E-11	2.22E-09	15.34134
MYL9	-1.82772	6.504131	-9.16381	3.84E-11	2.22E-09	15.33553
DHTKD1	1.226983	2.991641	9.158633	3.90E-11	2.24E-09	15.32079
PNPLA6	-1.37193	3.944427	-9.15167	3.98E-11	2.27E-09	15.30095
COX7A1	-1.59367	3.295384	-9.14385	4.07E-11	2.30E-09	15.27866
FGR	-2.01825	3.617062	-9.13597	4.16E-11	2.33E-09	15.25618
KAL1	-2.10441	3.598165	-9.1222	4.33E-11	2.42E-09	15.2169
F10	-1.28921	1.718171	-9.10495	4.55E-11	2.53E-09	15.16767
GPM6A	-2.5175	1.959624	-9.10324	4.58E-11	2.53E-09	15.16279
EFNA4	1.782037	2.808416	9.081244	4.88E-11	2.68E-09	15.09993
ARHGEF26	-2.19346	2.955353	-9.06947	5.04E-11	2.75E-09	15.06626
SERTM1	-2.15976	1.217343	-9.05905	5.20E-11	2.81E-09	15.03645
SOX7	-2.40466	2.339932	-9.05755	5.22E-11	2.81E-09	15.03216
NOTCH4	-1.45259	2.425318	-9.04407	5.43E-11	2.91E-09	14.99355
NOVA2	-1.21543	1.072299	-9.04017	5.49E-11	2.93E-09	14.98240
STARD13	-1.27858	2.300994	-9.03972	5.50E-11	2.93E-09	14.98110
PDIA6	1.166751	5.698917	9.035766	5.56E-11	2.94E-09	14.96978

Gene	logFC	AveExpr	t	P.Value	adj.P.Val	B
HSP90B1	1.033241	7.335402	9.035544	5.56E-11	2.94E-09	14.96915
FBXO32	2.015173	2.477028	9.014631	5.91E-11	3.11E-09	14.90921
DENND3	-1.46381	2.653772	-8.98902	6.37E-11	3.33E-09	14.83573
ZWINT	2.000054	2.555272	8.985008	6.44E-11	3.35E-09	14.82421
MPZL1	1.00499	4.582718	8.984722	6.44E-11	3.35E-09	14.82339
ADRB1	-2.59596	2.276976	-8.95658	6.99E-11	3.62E-09	14.74255
CNOT11	1.003407	4.142026	8.936612	7.41E-11	3.80E-09	14.68512
FXYD1	-1.29426	1.068959	-8.91318	7.93E-11	4.04E-09	14.61767
MFAP4	-2.8927	6.507258	-8.9089	8.03E-11	4.04E-09	14.60535
FEN1	1.452834	3.04526	8.907958	8.05E-11	4.04E-09	14.60263
RASGRP4	-1.09332	1.297262	-8.90724	8.07E-11	4.04E-09	14.60057
EFCC1	-1.94088	2.109053	-8.90589	8.10E-11	4.04E-09	14.59667
FAM150B	-1.8037	1.397962	-8.90384	8.15E-11	4.05E-09	14.59077
SEC14L6	-1.99141	1.973265	-8.89365	8.40E-11	4.16E-09	14.56140
GIMAP6	-1.78718	3.696747	-8.89079	8.47E-11	4.18E-09	14.55314
FGD5	-1.68715	2.797932	-8.88573	8.59E-11	4.22E-09	14.53855
CCNB1	2.420322	2.669806	8.882707	8.67E-11	4.24E-09	14.52985
ST14	1.497999	5.291319	8.878223	8.78E-11	4.27E-09	14.51691
CYYR1	-1.80867	3.209397	-8.8781	8.78E-11	4.27E-09	14.51657
DACH1	-1.48708	1.496549	-8.87262	8.93E-11	4.32E-09	14.50076
EPT1	1.083857	2.431591	8.869105	9.02E-11	4.35E-09	14.49061
CAMP	-1.72503	1.331276	-8.84227	9.75E-11	4.65E-09	14.41313
SFTPC	-6.98715	9.525409	-8.84203	9.76E-11	4.65E-09	14.41243
PLK1	1.909292	1.503628	8.833619	1.00E-10	4.73E-09	14.38813
ST6GALNAC6	-1.1817	4.07677	-8.81977	1.04E-10	4.91E-09	14.34809
TMEM184A	1.431074	1.2825	8.811751	1.07E-10	4.99E-09	14.32490
PUS7	1.106885	2.198806	8.808272	1.08E-10	5.02E-09	14.31484
S1PR4	-1.49693	2.698753	-8.80105	1.10E-10	5.11E-09	14.29394
OSCAR	-2.16152	3.48441	-8.7906	1.13E-10	5.23E-09	14.26370
MCM4	1.996887	2.874955	8.786833	1.15E-10	5.27E-09	14.25278
CD300LG	-2.03116	1.14258	-8.78548	1.15E-10	5.27E-09	14.24886
BUB1B	1.72252	1.196571	8.783821	1.16E-10	5.28E-09	14.24406
XPR1	1.647398	3.395539	8.77513	1.19E-10	5.40E-09	14.21889
DLC1	-2.20348	3.885784	-8.75595	1.25E-10	5.65E-09	14.16329
SFXN4	1.271467	3.09079	8.746136	1.29E-10	5.79E-09	14.13484
DENND2A	-1.23894	2.127574	-8.73902	1.32E-10	5.89E-09	14.11420
SOX17	-1.72609	1.931483	-8.73706	1.33E-10	5.91E-09	14.10849
SPAG5	1.737372	1.53821	8.728456	1.36E-10	6.02E-09	14.08353
FXYD6	-1.63146	2.854219	-8.72217	1.39E-10	6.11E-09	14.06529
ANKRD1	-3.95376	2.861335	-8.70982	1.44E-10	6.31E-09	14.02940

Gene	logFC	AveExpr	t	P.Value	adj.P.Val	B
SLC1A1	-2.22789	3.211817	-8.70676	1.45E-10	6.35E-09	14.02051
PAICS	1.638569	3.265007	8.697854	1.49E-10	6.48E-09	13.99464
MYZAP	-1.98915	1.887747	-8.69702	1.49E-10	6.48E-09	13.99220
GRIA1	-1.56895	1.148609	-8.69524	1.50E-10	6.50E-09	13.98703
CENPF	2.085222	1.473092	8.694078	1.50E-10	6.50E-09	13.98366
EZH2	1.449077	1.465835	8.691835	1.51E-10	6.52E-09	13.97714
C5orf38	-1.97859	2.263879	-8.69063	1.52E-10	6.52E-09	13.97362
DLGAP5	1.878798	1.322379	8.665767	1.63E-10	6.99E-09	13.90131
MMRN2	-1.63126	3.375155	-8.66299	1.65E-10	7.02E-09	13.89322
PKNOX2	-1.25336	1.235376	-8.65559	1.68E-10	7.11E-09	13.87169
GPX3	-2.57546	6.937518	-8.65552	1.68E-10	7.11E-09	13.87148
EIF2S3	1.138021	5.679239	8.652489	1.70E-10	7.15E-09	13.86265
EPCAM	1.446869	6.55476	8.650468	1.71E-10	7.16E-09	13.85677
LPGAT1	1.501659	3.487157	8.649632	1.71E-10	7.16E-09	13.85433
TARS2	1.125154	2.969748	8.642145	1.75E-10	7.30E-09	13.83252
JRKL	1.058146	2.493466	8.634226	1.79E-10	7.40E-09	13.80945
CD52	-2.48347	6.059753	-8.63124	1.81E-10	7.44E-09	13.80076
ECT2	1.883857	2.580814	8.625069	1.84E-10	7.52E-09	13.78276
NEK2	2.160476	1.451102	8.62436	1.85E-10	7.52E-09	13.78069
FHL5	-1.71659	2.005292	-8.62369	1.85E-10	7.52E-09	13.77874
FANCI	1.440046	1.641041	8.610796	1.92E-10	7.75E-09	13.74113
SCN4B	-1.82188	2.005766	-8.60992	1.93E-10	7.75E-09	13.73857
MGAT3	-2.49725	2.371211	-8.59496	2.01E-10	8.07E-09	13.69491
OCIAD2	1.977005	4.248222	8.570787	2.16E-10	8.64E-09	13.62432
SPTBN2	1.750893	1.701476	8.563585	2.21E-10	8.77E-09	13.60327
FAM114A1	1.236911	3.934346	8.563448	2.21E-10	8.77E-09	13.60287
HELLS	1.07771	0.861602	8.561938	2.22E-10	8.78E-09	13.59846
TCF21	-2.27334	2.417242	-8.56025	2.23E-10	8.78E-09	13.59352
GMNN	1.306415	2.440464	8.559935	2.23E-10	8.78E-09	13.59261
ARHGAP31	-1.66131	3.43578	-8.55697	2.25E-10	8.83E-09	13.58392
IL3RA	-1.48024	2.716953	-8.55503	2.26E-10	8.86E-09	13.57827
TAF1D	1.089785	2.692923	8.548647	2.31E-10	9.00E-09	13.5596
RHOJ	-1.49351	2.77776	-8.53189	2.42E-10	9.42E-09	13.51057
CALCOCO1	-1.13787	3.656336	-8.52692	2.46E-10	9.53E-09	13.49604
PRAM1	-1.55405	1.791007	-8.52232	2.49E-10	9.64E-09	13.48255
PLEK2	2.366604	2.58723	8.513227	2.56E-10	9.87E-09	13.45594
TONSL	1.076134	1.226416	8.501623	2.65E-10	1.02E-08	13.42194
ARHGAP44	-1.4884	2.313348	-8.50151	2.65E-10	1.02E-08	13.42162
CD300C	-1.50671	2.496017	-8.49583	2.70E-10	1.03E-08	13.40497
SYNPO	-1.69519	4.36764	-8.49398	2.71E-10	1.03E-08	13.39955

Gene	logFC	AveExpr	t	P.Value	adj.P.Val	B
RRAS	-1.57265	5.780366	-8.49283	2.72E-10	1.03E-08	13.39617
SUSD2	-3.62223	5.253552	-8.4771	2.85E-10	1.07E-08	13.35005
MS4A7	-1.85685	3.988895	-8.47647	2.86E-10	1.07E-08	13.34822
C10orf54	-1.49174	4.130943	-8.46206	2.98E-10	1.12E-08	13.30592
SLC19A3	-1.70342	1.542272	-8.45962	3.00E-10	1.12E-08	13.29875
NHSL1	-1.50982	2.477521	-8.45164	3.07E-10	1.14E-08	13.27533
CSRNP1	-2.29256	5.12113	-8.45083	3.08E-10	1.14E-08	13.27295
NUSAP1	2.047811	2.405581	8.449142	3.10E-10	1.14E-08	13.26800
PTPRB	-2.12876	3.046611	-8.44848	3.10E-10	1.14E-08	13.26605
KIAA1324L	-1.55614	2.605571	-8.44527	3.13E-10	1.15E-08	13.25662
KIF20A	1.802095	1.463212	8.441938	3.16E-10	1.16E-08	13.24685
FAM136A	1.0727	3.350111	8.436986	3.21E-10	1.17E-08	13.23230
CALCRL	-2.06271	3.88073	-8.43501	3.23E-10	1.17E-08	13.22650
BCHE	-1.54433	1.564192	-8.43156	3.26E-10	1.18E-08	13.21637
IGSF9	1.95871	1.30792	8.42392	3.34E-10	1.20E-08	13.19390
CHEK1	1.347802	1.247893	8.423513	3.34E-10	1.20E-08	13.19271
B3GNT3	3.071349	2.123564	8.42098	3.37E-10	1.21E-08	13.18526
CD93	-1.84752	5.106433	-8.40577	3.52E-10	1.25E-08	13.14054
UHRF1	1.661141	1.142506	8.403278	3.55E-10	1.26E-08	13.13321
ENTPD7	1.448012	2.264583	8.397648	3.61E-10	1.28E-08	13.11664
WIF1	-4.25361	4.192882	-8.39206	3.67E-10	1.29E-08	13.10019
WASF3	-1.61819	2.122185	-8.38831	3.71E-10	1.30E-08	13.08915
ITM2C	1.131386	5.096086	8.387479	3.72E-10	1.30E-08	13.08672
TNS1	-2.17975	5.000454	-8.37913	3.81E-10	1.33E-08	13.06214
DAAM2	-1.31031	2.292634	-8.37607	3.85E-10	1.33E-08	13.05312
MAMDC2	-2.33185	2.947087	-8.37552	3.85E-10	1.33E-08	13.05152
FAM111B	1.448182	1.206757	8.373061	3.88E-10	1.33E-08	13.04427
SEMA6A	-1.55075	1.567683	-8.36891	3.93E-10	1.34E-08	13.03203
CLDN12	1.272085	3.059144	8.368747	3.93E-10	1.34E-08	13.03156
VEPH1	-2.15814	2.625719	-8.36793	3.94E-10	1.34E-08	13.02914
C12orf49	-1.20319	4.023273	-8.36509	3.97E-10	1.35E-08	13.02078
CRABP2	4.501835	3.93073	8.352347	4.13E-10	1.40E-08	12.98323
KRT4	-2.56579	2.079881	-8.34937	4.16E-10	1.41E-08	12.97446
SLC9A7	1.356732	1.781755	8.340734	4.27E-10	1.44E-08	12.94900
ODAM	-1.28804	0.951695	-8.3367	4.32E-10	1.45E-08	12.93709
ABI3BP	-2.14368	2.850761	-8.33177	4.39E-10	1.47E-08	12.92256
HEATR1	1.287381	2.425652	8.323689	4.49E-10	1.50E-08	12.89872
KIF4A	1.931512	1.305425	8.323262	4.50E-10	1.50E-08	12.89746
MKI67	2.056028	1.675972	8.321293	4.53E-10	1.50E-08	12.89165
SFTPD	-3.52338	7.66762	-8.31977	4.55E-10	1.50E-08	12.88715

Gene	logFC	AveExpr	t	P.Value	adj.P.Val	B
MCM6	1.417131	3.186064	8.308181	4.71E-10	1.55E-08	12.85294
PTH1R	-1.15652	1.412923	-8.30716	4.72E-10	1.55E-08	12.84992
SHMT2	1.244945	3.66	8.300939	4.81E-10	1.57E-08	12.83156
LHFPL3	-1.26854	0.963437	-8.29396	4.91E-10	1.60E-08	12.81094
PFKP	2.088526	4.16782	8.272312	5.24E-10	1.70E-08	12.74696
GNPNAT1	1.275074	2.930787	8.264034	5.37E-10	1.74E-08	12.72248
CCM2L	-1.58633	1.87079	-8.26326	5.38E-10	1.74E-08	12.72020
PODXL2	2.408957	2.87737	8.252869	5.55E-10	1.78E-08	12.68945
CCNB2	2.020839	1.78346	8.250665	5.59E-10	1.79E-08	12.68293
SPN	-1.74914	2.953845	-8.24418	5.70E-10	1.82E-08	12.66375
MFSD2A	-2.0683	4.100386	-8.24337	5.71E-10	1.82E-08	12.66133
CD300LF	-1.5751	2.928654	-8.23918	5.78E-10	1.84E-08	12.64893
CDCA7	1.760845	1.529266	8.239128	5.78E-10	1.84E-08	12.64879
MRPL3	1.139444	4.646496	8.236209	5.83E-10	1.85E-08	12.64014
PARP1	1.273926	4.462247	8.232158	5.91E-10	1.86E-08	12.62815
GIMAP8	-1.9394	3.354798	-8.23064	5.93E-10	1.86E-08	12.62365
ZNF146	1.057829	4.273779	8.223778	6.05E-10	1.89E-08	12.60333
MME	-1.937	2.513953	-8.22303	6.07E-10	1.89E-08	12.60110
CDC20	2.653686	2.304033	8.219723	6.13E-10	1.90E-08	12.59132
PPP1R14A	-1.95635	3.209354	-8.21765	6.17E-10	1.91E-08	12.58517
NCAPG2	1.348813	1.833269	8.217162	6.18E-10	1.91E-08	12.58373
FHL2	2.21613	2.611358	8.211158	6.29E-10	1.94E-08	12.56594
GINS2	1.609364	1.318184	8.206465	6.38E-10	1.96E-08	12.55203
RSPO1	-1.23631	0.950667	-8.20038	6.49E-10	1.99E-08	12.53401
RXFP1	-1.11804	0.886115	-8.19948	6.51E-10	1.99E-08	12.53134
THBD	-2.29554	4.636185	-8.19931	6.51E-10	1.99E-08	12.53081
TCEAL2	-1.68238	1.500167	-8.19806	6.54E-10	1.99E-08	12.52713
USHBP1	-1.10013	1.037492	-8.1953	6.59E-10	2.00E-08	12.51895
ABCG1	-1.24411	3.531512	-8.193	6.64E-10	2.01E-08	12.5121
RAI2	-1.803	3.217671	-8.18531	6.79E-10	2.06E-08	12.4893
FOLR3	-1.35057	0.977722	-8.18294	6.84E-10	2.06E-08	12.48227
AK1	-1.40896	3.221082	-8.1766	6.97E-10	2.09E-08	12.46345
KDELR3	2.158694	3.54611	8.176339	6.98E-10	2.09E-08	12.46269
SEMA5A	-1.73382	2.660331	-8.1748	7.01E-10	2.09E-08	12.45811
HMGB3	2.536937	3.912037	8.174085	7.03E-10	2.09E-08	12.45600
FLAD1	1.291861	3.136304	8.171229	7.09E-10	2.10E-08	12.44752
PID1	-1.62714	3.212477	-8.17098	7.09E-10	2.10E-08	12.44679
OTUD1	-1.60864	3.667906	-8.16938	7.12E-10	2.10E-08	12.44203
PEAR1	-1.34471	1.853974	-8.1647	7.23E-10	2.12E-08	12.42815
IQGAP3	1.86113	1.358356	8.163301	7.26E-10	2.13E-08	12.42399

Gene	logFC	AveExpr	t	P.Value	adj.P.Val	B
HSPB6	-2.88548	3.129144	-8.16167	7.29E-10	2.13E-08	12.41916
TPX2	2.638088	2.371493	8.153395	7.47E-10	2.18E-08	12.39457
CCT5	1.288844	4.762567	8.152445	7.50E-10	2.18E-08	12.39175
KIF2C	2.035905	1.53635	8.151523	7.52E-10	2.18E-08	12.38901
AGRP	-1.60243	1.400525	-8.14986	7.55E-10	2.19E-08	12.38408
STIL	1.137905	0.919625	8.142938	7.71E-10	2.23E-08	12.36351
PXDC1	-1.27886	4.390161	-8.13322	7.94E-10	2.29E-08	12.33462
TGFBR2	-1.46424	6.225877	-8.12696	8.09E-10	2.33E-08	12.31603
ARRB2	-1.04078	4.015289	-8.11675	8.34E-10	2.39E-08	12.28568
DOK2	-1.76243	3.663947	-8.10351	8.68E-10	2.48E-08	12.24629
ANLN	2.40627	1.8052	8.101555	8.73E-10	2.48E-08	12.24047
CDC6	1.759521	1.298752	8.087978	9.10E-10	2.58E-08	12.20006
NUP62CL	1.222807	1.217818	8.084383	9.19E-10	2.60E-08	12.18935
CCNA2	2.008529	1.837185	8.081567	9.27E-10	2.61E-08	12.18097
COL10A1	3.206509	2.162094	8.080992	9.29E-10	2.61E-08	12.17926
B4GALT2	1.114684	3.491238	8.079281	9.34E-10	2.62E-08	12.17416
AURKA	1.729112	1.981135	8.072472	9.53E-10	2.67E-08	12.15388
CLEC14A	-1.96745	4.543499	-8.07068	9.58E-10	2.68E-08	12.14853
CBX4	1.018039	3.349713	8.061991	9.83E-10	2.74E-08	12.12266
WFS1	-1.63346	4.518643	-8.03135	1.08E-09	2.96E-08	12.03129
UHMK1	1.121593	4.229617	8.029123	1.09E-09	2.98E-08	12.02466
F11	-1.27537	1.096132	-8.02738	1.09E-09	2.99E-08	12.01946
KIF26B	1.679881	1.22611	8.025489	1.10E-09	3.00E-08	12.01381
BCL6B	-1.48857	2.633081	-8.01309	1.14E-09	3.10E-08	11.97682
GPA33	-2.00927	1.431296	-8.00716	1.16E-09	3.15E-08	11.95909
TTK	1.340702	0.937004	8.005099	1.17E-09	3.17E-08	11.95295
TTC39C	1.249028	1.861431	8.000433	1.18E-09	3.20E-08	11.93902
CEP55	2.223039	1.829332	7.998004	1.19E-09	3.22E-08	11.93176
C1orf106	2.041913	2.322379	7.990991	1.22E-09	3.28E-08	11.91082
FOXF1	-2.00374	2.79113	-7.98999	1.22E-09	3.28E-08	11.90783
CYBRD1	-1.73246	5.535518	-7.97355	1.28E-09	3.44E-08	11.8587
TIMELESS	1.304252	2.335046	7.970785	1.29E-09	3.46E-08	11.85043
SAPCD2	1.734971	1.19841	7.967185	1.31E-09	3.49E-08	11.83967
ORC5	1.049538	2.299522	7.961461	1.33E-09	3.53E-08	11.82255
SEZ6L2	1.553728	3.748906	7.961277	1.33E-09	3.53E-08	11.82200
FAM162B	-1.59487	2.38796	-7.96103	1.33E-09	3.53E-08	11.82127
HLA-E	-1.36255	8.830638	-7.95868	1.34E-09	3.53E-08	11.81424
SELP	-1.83658	2.575448	-7.95702	1.35E-09	3.53E-08	11.80927
SFTPA2	-4.24623	10.70812	-7.95674	1.35E-09	3.53E-08	11.80844
SFTPA1	-4.35548	10.55854	-7.95661	1.35E-09	3.53E-08	11.80804

Gene	logFC	AveExpr	t	P.Value	adj.P.Val	B
CCNF	1.022532	1.289087	7.948429	1.38E-09	3.60E-08	11.78357
GIMAP1	-1.43162	1.980141	-7.94748	1.39E-09	3.61E-08	11.78073
LTBP4	-2.08202	4.752657	-7.94303	1.41E-09	3.63E-08	11.76742
SPRYD7	-1.03668	2.878568	-7.93543	1.44E-09	3.71E-08	11.74468
GFPT1	1.358273	4.032903	7.931956	1.46E-09	3.74E-08	11.73427
GLDN	-1.59497	1.604379	-7.92967	1.47E-09	3.76E-08	11.72742
SULT1A2	-1.19551	1.497524	-7.92651	1.48E-09	3.79E-08	11.71797
NDRG2	-1.54779	3.170727	-7.91953	1.51E-09	3.84E-08	11.69707
BHLHA15	1.203769	1.147539	7.904372	1.58E-09	4.00E-08	11.65166
KAT2A	1.203228	2.989894	7.895673	1.62E-09	4.09E-08	11.62559
DNAJC10	1.023383	2.751486	7.888951	1.66E-09	4.15E-08	11.60544
CDT1	1.610536	1.512876	7.888106	1.66E-09	4.15E-08	11.60291
PRDX4	1.573132	5.275587	7.88541	1.67E-09	4.18E-08	11.59482
MAGED1	1.03814	4.949928	7.875572	1.73E-09	4.29E-08	11.56532
PDK1	1.06	1.233127	7.871346	1.75E-09	4.33E-08	11.55264
AQP4	-3.36089	4.958499	-7.87021	1.75E-09	4.33E-08	11.54924
LRRK2	-2.18566	3.750018	-7.8613	1.80E-09	4.42E-08	11.52249
CARD11	1.735398	2.198092	7.85945	1.81E-09	4.44E-08	11.51695
FAM60A	1.124563	3.503982	7.852402	1.85E-09	4.52E-08	11.49580
HHIP	-2.13258	2.245282	-7.84729	1.88E-09	4.57E-08	11.48044
STX1A	1.410029	1.295834	7.846966	1.88E-09	4.57E-08	11.47948
ANGPTL1	-1.45762	1.63019	-7.84573	1.89E-09	4.58E-08	11.47575
APOBR	-1.77961	2.784339	-7.83739	1.94E-09	4.69E-08	11.45072
GINS1	1.455205	1.238024	7.831929	1.97E-09	4.76E-08	11.43432
CD34	-1.49341	3.265631	-7.82987	1.98E-09	4.78E-08	11.42814
KNTC1	1.221598	1.220379	7.82533	2.01E-09	4.84E-08	11.41450
PDGFB	-1.53663	3.483937	-7.81769	2.06E-09	4.93E-08	11.39155
CCDC34	1.118122	1.514778	7.812256	2.09E-09	4.99E-08	11.37521
TRAF4	1.043729	3.82593	7.81077	2.10E-09	5.01E-08	11.37074
CDO1	-1.59252	1.703265	-7.8103	2.10E-09	5.01E-08	11.36932
INCENP	1.123408	2.184229	7.807848	2.12E-09	5.03E-08	11.36196
RGS9	-1.07192	0.970263	-7.80494	2.14E-09	5.07E-08	11.35321
COPB2	1.01656	4.49025	7.802335	2.15E-09	5.10E-08	11.34538
TGM1	-1.23464	1.040397	-7.8013	2.16E-09	5.10E-08	11.34226
ANKRD29	-1.91249	2.585139	-7.79834	2.18E-09	5.13E-08	11.33338
TDRKH	1.154968	2.244671	7.792128	2.22E-09	5.21E-08	11.31469
FIGNL1	1.16167	1.592227	7.790609	2.23E-09	5.23E-08	11.31012
TOX2	-1.4313	2.185578	-7.78481	2.27E-09	5.29E-08	11.29267
ZNF217	1.328771	3.357801	7.778245	2.32E-09	5.39E-08	11.27292
PODXL	-1.25955	4.072552	-7.7756	2.34E-09	5.42E-08	11.26495

Gene	logFC	AveExpr	t	P.Value	adj.P.Val	B
P4HB	1.156062	7.32057	7.773842	2.35E-09	5.44E-08	11.25967
C1orf162	-1.70895	3.615211	-7.77219	2.36E-09	5.45E-08	11.25470
MDK	2.080669	4.96017	7.771919	2.36E-09	5.45E-08	11.25388
ELMO3	1.268266	3.588738	7.770878	2.37E-09	5.46E-08	11.25075
PRC1	1.779116	1.873489	7.754896	2.49E-09	5.72E-08	11.20263
BMPER	-1.24276	1.042229	-7.75445	2.49E-09	5.72E-08	11.20129
FAM83A	3.980175	2.152565	7.746443	2.55E-09	5.84E-08	11.17717
SLC16A11	-1.29589	1.453005	-7.7416	2.59E-09	5.90E-08	11.16259
SNX22	-1.27333	1.485605	-7.73981	2.60E-09	5.92E-08	11.15720
ACTL6A	1.061633	3.642796	7.73565	2.64E-09	5.98E-08	11.14466
AGTR1	-1.10605	1.181753	-7.73555	2.64E-09	5.98E-08	11.14435
TMPRSS4	2.765557	1.664275	7.730265	2.68E-09	6.06E-08	11.12843
RBP2	-1.739	1.227096	-7.72458	2.73E-09	6.15E-08	11.11129
GPR146	-1.02217	1.01263	-7.72351	2.74E-09	6.16E-08	11.10807
DKC1	1.011889	3.679452	7.723084	2.74E-09	6.16E-08	11.10679
SHCBP1	1.378632	1.236055	7.716435	2.80E-09	6.27E-08	11.08674
BRI3BP	1.147202	2.103154	7.715282	2.81E-09	6.28E-08	11.08326
ODF3L1	-1.19442	1.100178	-7.69825	2.96E-09	6.56E-08	11.03189
CCDC85A	-1.14665	1.05494	-7.69816	2.96E-09	6.56E-08	11.03163
ZNF385B	-1.94967	2.336794	-7.69575	2.98E-09	6.60E-08	11.02436
2-Mar	-1.34804	3.432075	-7.69478	2.99E-09	6.61E-08	11.02141
CDH3	2.879077	2.90811	7.68676	3.06E-09	6.76E-08	10.99722
MTFR1	1.196222	2.679466	7.685142	3.08E-09	6.77E-08	10.99234
ITM2A	-1.89179	4.147852	-7.68397	3.09E-09	6.78E-08	10.98879
NME1	1.845172	3.069765	7.683054	3.10E-09	6.79E-08	10.98604
ALPL	-2.5469	5.260142	-7.68226	3.10E-09	6.79E-08	10.98363
NXPH3	-1.10683	1.11391	-7.68071	3.12E-09	6.81E-08	10.97895
FANCF	1.105386	2.119066	7.659957	3.32E-09	7.21E-08	10.91630
TWF1	1.106474	3.83529	7.655816	3.36E-09	7.29E-08	10.90379
HBB	-4.17421	7.11504	-7.65286	3.39E-09	7.34E-08	10.89486
SORD	1.085661	1.772276	7.650439	3.42E-09	7.38E-08	10.88754
SLIT2	-1.86397	2.859992	-7.64938	3.43E-09	7.40E-08	10.88433
TOM1L2	-1.23516	3.242177	-7.64831	3.44E-09	7.41E-08	10.88111
HMMR	1.756052	1.359595	7.646797	3.46E-09	7.43E-08	10.87654
EXO1	1.404202	0.944596	7.644751	3.48E-09	7.45E-08	10.87035
RETN	-2.81968	3.054459	-7.64216	3.51E-09	7.50E-08	10.86253
XDH	1.808237	1.168274	7.638612	3.54E-09	7.55E-08	10.85180
FAM110D	-1.43237	1.624713	-7.63715	3.56E-09	7.58E-08	10.84739
HPS3	1.017016	2.677632	7.635083	3.58E-09	7.61E-08	10.84113
MSRB3	-1.40848	3.241146	-7.62729	3.67E-09	7.78E-08	10.81756

Gene	logFC	AveExpr	t	P.Value	adj.P.Val	B
MCM2	1.847506	2.736737	7.622747	3.72E-09	7.88E-08	10.80383
ASPM	1.438992	0.896009	7.614491	3.82E-09	8.05E-08	10.77886
UBE2C	2.919251	2.69206	7.614006	3.82E-09	8.05E-08	10.77740
PCNA	1.119738	5.224469	7.611722	3.85E-09	8.09E-08	10.77049
FRMD3	-1.16959	1.385394	-7.6097	3.87E-09	8.13E-08	10.76438
CTHRC1	3.183361	3.058328	7.609077	3.88E-09	8.13E-08	10.76248
SKA3	1.203249	0.900907	7.601276	3.97E-09	8.31E-08	10.73888
C15orf48	3.265375	3.81484	7.600457	3.98E-09	8.32E-08	10.73640
NMUR1	-1.14933	0.931518	-7.59775	4.02E-09	8.38E-08	10.72822
CGNL1	-1.87394	3.309337	-7.59478	4.05E-09	8.43E-08	10.71922
NCAPH	1.628822	1.341571	7.587968	4.14E-09	8.56E-08	10.69860
CPAMD8	-1.76171	2.238668	-7.58121	4.22E-09	8.73E-08	10.67814
CKS1B	1.607905	2.921225	7.576855	4.28E-09	8.80E-08	10.66496
GPR87	2.921031	1.67634	7.569733	4.37E-09	8.95E-08	10.64338
MS4A15	-3.53232	3.257803	-7.56444	4.45E-09	9.07E-08	10.62734
MND1	1.05694	0.876119	7.562393	4.47E-09	9.10E-08	10.62115
TACC1	-1.53428	4.212119	-7.5429	4.75E-09	9.58E-08	10.56208
TMEM150B	-1.35351	1.955387	-7.53701	4.84E-09	9.74E-08	10.54422
KIF23	1.351858	1.209294	7.527218	4.98E-09	1.00E-07	10.51452
FUT2	1.82662	1.405084	7.522975	5.05E-09	1.01E-07	10.50166
PVRL4	2.027854	2.984124	7.512997	5.20E-09	1.03E-07	10.47139
GIMAP7	-1.77026	4.165096	-7.51031	5.25E-09	1.04E-07	10.46325
EPN3	1.614978	1.162521	7.50812	5.28E-09	1.05E-07	10.45659
TMED2	1.02024	6.328507	7.506026	5.32E-09	1.05E-07	10.45023
TSPAN7	-2.43535	3.561062	-7.49699	5.47E-09	1.07E-07	10.42280
ADAMTSL4	-1.70695	2.94211	-7.49635	5.48E-09	1.07E-07	10.42088
CBLC	2.404409	2.231583	7.493358	5.53E-09	1.08E-07	10.41178
NPNT	-2.27956	4.279816	-7.48949	5.59E-09	1.09E-07	10.40005
HOOK1	1.042138	2.418255	7.487207	5.63E-09	1.09E-07	10.39311
SLC11A1	-1.65475	2.706414	-7.48633	5.65E-09	1.10E-07	10.39045
CHAF1B	1.029323	1.188528	7.48363	5.69E-09	1.10E-07	10.38225
NLN	1.102333	1.744224	7.479096	5.77E-09	1.12E-07	10.36848
TMEM178A	-1.20628	1.179545	-7.47229	5.90E-09	1.14E-07	10.34781
SASH1	-1.4222	3.043391	-7.46733	5.99E-09	1.15E-07	10.33274
SLCO2A1	-2.2313	4.284818	-7.4651	6.03E-09	1.16E-07	10.32596
SPARCL1	-1.75981	6.622646	-7.45832	6.15E-09	1.18E-07	10.30537
ABCA3	-2.68637	5.708274	-7.45011	6.31E-09	1.20E-07	10.28040
FAT1	1.694176	3.342514	7.44697	6.37E-09	1.21E-07	10.27086
FHDC1	-1.07049	2.548643	-7.43735	6.56E-09	1.24E-07	10.24162
VPS72	1.006476	3.37012	7.436307	6.58E-09	1.24E-07	10.23844

Gene	logFC	AveExpr	t	P.Value	adj.P.Val	B
KIF1C	-1.19057	4.719897	-7.43186	6.67E-09	1.26E-07	10.22493
AMICA1	-1.30058	2.489794	-7.43168	6.68E-09	1.26E-07	10.22438
BUB1	1.552602	1.377758	7.429513	6.72E-09	1.26E-07	10.21778
CANT1	1.054822	4.437388	7.427503	6.76E-09	1.27E-07	10.21167
LMCD1	-1.21317	3.022213	-7.42068	6.91E-09	1.29E-07	10.19091
ALG1L	1.949699	1.685162	7.402808	7.30E-09	1.35E-07	10.13653
DHCR24	-1.1906	7.560178	-7.3992	7.38E-09	1.36E-07	10.12555
TMTC4	1.029528	1.962584	7.396809	7.43E-09	1.37E-07	10.11827
CBX3	1.066938	4.894129	7.395515	7.46E-09	1.37E-07	10.11433
ACKR1	-2.43745	3.618803	-7.39259	7.53E-09	1.38E-07	10.10542
SYNJ2	1.154639	1.637152	7.391898	7.55E-09	1.38E-07	10.10332
FLI1	-1.2642	2.559111	-7.3893	7.61E-09	1.39E-07	10.09541
CHD1L	1.04168	3.912226	7.386945	7.66E-09	1.40E-07	10.08824
COL3A1	3.102972	6.733751	7.386203	7.68E-09	1.40E-07	10.08598
MELK	1.910698	1.416357	7.382339	7.77E-09	1.41E-07	10.07421
LAMC3	-1.4558	2.163373	-7.38184	7.78E-09	1.41E-07	10.07268
SRD5A1	1.293391	1.758097	7.374676	7.96E-09	1.43E-07	10.05087
MYBL2	2.678022	2.213238	7.372476	8.01E-09	1.44E-07	10.04417
EMR1	-1.53056	1.545027	-7.36718	8.14E-09	1.46E-07	10.02805
OLR1	-2.56084	4.482871	-7.36492	8.20E-09	1.47E-07	10.02115
GJA4	-1.2929	2.975537	-7.36358	8.23E-09	1.47E-07	10.01707
PPP1R15A	-1.80152	5.539999	-7.35978	8.33E-09	1.49E-07	10.00548
GALNT7	1.704778	2.904698	7.347468	8.65E-09	1.54E-07	9.967964
DTL	1.4862	1.524457	7.346567	8.67E-09	1.54E-07	9.965219
MARCO	-3.15141	5.885823	-7.3382	8.90E-09	1.58E-07	9.939719
AURKB	1.723366	1.421157	7.338158	8.90E-09	1.58E-07	9.939582
IRAK1	1.134812	4.804344	7.332568	9.06E-09	1.60E-07	9.922537
THBS2	2.817297	3.579161	7.332013	9.07E-09	1.60E-07	9.920843
TMCO1	1.015691	4.325135	7.32536	9.26E-09	1.63E-07	9.900555
PCOLCE2	-1.78844	2.389916	-7.3246	9.28E-09	1.63E-07	9.898230
CDCA8	1.933115	1.80971	7.314479	9.57E-09	1.68E-07	9.867361
NDC80	1.386452	1.121446	7.305282	9.85E-09	1.72E-07	9.839298
CYP4B1	-3.89921	5.039926	-7.29111	1.03E-08	1.79E-07	9.796048
MSH2	1.128262	2.871516	7.289589	1.03E-08	1.80E-07	9.791397
KHDRBS2	-1.36997	1.305615	-7.28879	1.04E-08	1.80E-07	9.788949
ADCY4	-1.17473	1.434846	-7.28632	1.04E-08	1.81E-07	9.781427
CTTN	1.01058	5.113095	7.277196	1.07E-08	1.85E-07	9.753557
CKAP2L	1.239867	0.896086	7.276373	1.08E-08	1.85E-07	9.751041
ATAD2	1.496552	2.219536	7.273247	1.09E-08	1.87E-07	9.741496
PTGDS	-2.12356	5.28276	-7.2718	1.09E-08	1.87E-07	9.737060

Gene	logFC	AveExpr	t	P.Value	adj.P.Val	B
KIF20B	1.011314	1.362938	7.27014	1.10E-08	1.88E-07	9.732004
VARS	1.13719	3.86334	7.263928	1.12E-08	1.90E-07	9.713029
TBC1D2	-1.12481	4.279411	-7.25824	1.14E-08	1.93E-07	9.695661
ENG	-1.82873	5.783655	-7.25806	1.14E-08	1.93E-07	9.695091
PITX1	2.051563	1.186484	7.255923	1.15E-08	1.94E-07	9.688570
RAB19	1.119338	1.068253	7.254573	1.15E-08	1.94E-07	9.684445
LAD1	1.874234	4.498617	7.252673	1.16E-08	1.95E-07	9.678640
ACOXL	-1.27156	1.365323	-7.25244	1.16E-08	1.95E-07	9.677923
WDHD1	1.055496	1.279274	7.240413	1.20E-08	2.01E-07	9.641168
ADH1B	-3.27314	4.239614	-7.23852	1.21E-08	2.02E-07	9.635374
CPD	1.40095	4.229405	7.237362	1.21E-08	2.02E-07	9.631841
NUF2	1.766543	1.222778	7.237191	1.22E-08	2.02E-07	9.631319
P4HA1	1.147739	4.840626	7.233343	1.23E-08	2.05E-07	9.619554
TNS2	-1.53703	3.930166	-7.23158	1.24E-08	2.05E-07	9.614172
ADM2	1.264834	0.917219	7.231303	1.24E-08	2.05E-07	9.613317
SLC27A3	-1.4736	3.250455	-7.22896	1.25E-08	2.06E-07	9.606153
ICAM2	-1.10297	2.765306	-7.22415	1.26E-08	2.09E-07	9.591437
GMFG	-1.44532	4.494588	-7.22384	1.27E-08	2.09E-07	9.590486
SPC24	1.348066	1.196416	7.22091	1.28E-08	2.10E-07	9.581536
GPR4	-1.59375	2.210368	-7.21812	1.29E-08	2.11E-07	9.572990
C11orf96	-2.04481	4.481142	-7.21776	1.29E-08	2.11E-07	9.571904
WISP2	-1.69482	2.3463	-7.21746	1.29E-08	2.11E-07	9.570999
AATK	-1.64556	1.949055	-7.21693	1.29E-08	2.11E-07	9.569366
TSPAN18	-1.59521	2.781525	-7.21623	1.30E-08	2.11E-07	9.567219
PTGES	2.273426	3.003643	7.209822	1.32E-08	2.15E-07	9.547623
BIRC5	2.375026	1.981471	7.2087	1.33E-08	2.15E-07	9.544190
IDH2	1.004117	4.932153	7.201355	1.36E-08	2.20E-07	9.521717
KIFC1	1.674358	1.844364	7.192023	1.40E-08	2.25E-07	9.493158
ERO1L	1.930786	3.99074	7.1882	1.41E-08	2.28E-07	9.481458
KDM5B	1.220067	3.079093	7.186576	1.42E-08	2.28E-07	9.476486
PLK4	1.006276	1.032358	7.181437	1.44E-08	2.31E-07	9.460757
RASGRF1	-1.93737	2.019697	-7.1778	1.46E-08	2.34E-07	9.449624
IRX2	-2.18985	2.939085	-7.17693	1.46E-08	2.34E-07	9.446965
MSI2	1.160893	1.839282	7.1693	1.50E-08	2.39E-07	9.423595
AVL9	1.144432	2.354809	7.165262	1.52E-08	2.42E-07	9.411230
RECQL4	1.576741	1.467754	7.159499	1.54E-08	2.46E-07	9.393578
ADAMTSL3	-1.20156	1.502893	-7.15879	1.55E-08	2.46E-07	9.391413
KIF2A	1.044166	2.695931	7.155205	1.57E-08	2.48E-07	9.380425
MCM10	1.178599	0.772751	7.147196	1.60E-08	2.54E-07	9.355890
NQO1	2.675985	4.666586	7.145044	1.62E-08	2.55E-07	9.349296

Gene	logFC	AveExpr	t	P.Value	adj.P.Val	B
TNNI2	-1.35312	1.643192	-7.1407	1.64E-08	2.58E-07	9.335993
NGEF	1.767274	1.096623	7.139311	1.64E-08	2.59E-07	9.331729
CD101	-1.58794	1.885186	-7.13177	1.68E-08	2.64E-07	9.308633
SOCS2	-2.00402	2.920612	-7.13079	1.69E-08	2.65E-07	9.305613
CENPU	1.504292	1.74181	7.13022	1.69E-08	2.65E-07	9.303868
HJURP	1.696412	1.162173	7.126293	1.71E-08	2.68E-07	9.291832
SHROOM4	-1.36507	2.491703	-7.12532	1.72E-08	2.68E-07	9.288834
MAN2A1	1.009631	2.775431	7.124311	1.72E-08	2.68E-07	9.285757
VWF	-1.98496	5.548549	-7.1222	1.73E-08	2.70E-07	9.279294
P3H4	1.520976	2.468405	7.116017	1.77E-08	2.74E-07	9.260327
ORC1	1.350503	1.088537	7.114419	1.78E-08	2.76E-07	9.255429
MAD2L1	1.367233	1.26919	7.108534	1.81E-08	2.79E-07	9.237381
HDGF	1.132237	6.290585	7.10491	1.83E-08	2.81E-07	9.226269
RAD51	1.110947	1.091646	7.104337	1.83E-08	2.82E-07	9.224511
LGALSL	-1.56455	3.306104	-7.10097	1.85E-08	2.84E-07	9.214187
NDC1	1.059944	2.622185	7.092944	1.90E-08	2.90E-07	9.189567
GJB2	2.706833	1.952064	7.089499	1.92E-08	2.92E-07	9.178997
RFC4	1.391514	2.067454	7.088142	1.93E-08	2.93E-07	9.174832
COL6A6	-1.639	1.86919	-7.08746	1.93E-08	2.93E-07	9.172732
NLRC4	-1.12886	1.835562	-7.0863	1.94E-08	2.94E-07	9.169171
SPI1	-1.81418	4.845139	-7.08287	1.96E-08	2.97E-07	9.158673
EMR3	-1.09213	1.065607	-7.07797	1.99E-08	3.00E-07	9.143629
ABCA8	-1.64521	1.810118	-7.07414	2.01E-08	3.03E-07	9.131872
ASF1B	1.660244	2.112589	7.052863	2.15E-08	3.21E-07	9.066554
PIP5K1B	-1.77398	2.354457	-7.05206	2.15E-08	3.21E-07	9.064101
MOCS1	-1.1076	2.192605	-7.04813	2.18E-08	3.24E-07	9.052023
CHMP4C	1.113656	3.508199	7.046333	2.19E-08	3.25E-07	9.046502
ETV4	1.952569	1.851083	7.044366	2.21E-08	3.27E-07	9.040461
NSUN2	1.054591	3.842654	7.040135	2.24E-08	3.30E-07	9.027466
CFD	-2.38617	5.465383	-7.03774	2.25E-08	3.33E-07	9.020105
PLA1A	-1.54443	2.305506	-7.03652	2.26E-08	3.33E-07	9.016351
GPRIN1	1.37698	1.133226	7.035307	2.27E-08	3.34E-07	9.012636
HBA1	-2.02919	1.388105	-7.03172	2.29E-08	3.38E-07	9.001626
UGGT1	1.092727	3.400448	7.031379	2.30E-08	3.38E-07	9.000571
CDCA5	1.679613	1.573479	7.027704	2.32E-08	3.40E-07	8.989280
RRM2	2.142556	2.060657	7.020421	2.38E-08	3.46E-07	8.966903
GALNT18	-1.36227	3.664565	-7.01977	2.38E-08	3.46E-07	8.964888
PTTG1	1.799413	2.508892	7.013774	2.43E-08	3.51E-07	8.946477
SLC15A3	-1.32971	3.524081	-7.01071	2.45E-08	3.53E-07	8.937048
EEF1A2	3.233223	2.000741	7.009548	2.46E-08	3.54E-07	8.933492

Gene	logFC	AveExpr	t	P.Value	adj.P.Val	B
PPIL1	1.046395	3.873137	7.006394	2.48E-08	3.56E-07	8.923798
CXCL13	3.515315	2.361788	7.003735	2.50E-08	3.58E-07	8.915624
GATA2	-1.63394	2.411005	-7.00273	2.51E-08	3.59E-07	8.912533
MANF	1.168965	4.488094	6.999147	2.54E-08	3.62E-07	8.901521
ABCC3	2.089953	3.192898	6.995523	2.57E-08	3.66E-07	8.89038
NPM3	1.452919	3.68227	6.98153	2.68E-08	3.78E-07	8.847354
TMEM74B	-1.1007	1.374839	-6.9776	2.71E-08	3.82E-07	8.835283
RHBDL2	1.561784	1.340186	6.976769	2.72E-08	3.82E-07	8.832712
ITGB4	2.056332	4.376779	6.976754	2.72E-08	3.82E-07	8.832667
A2M	-1.89635	8.352002	-6.97662	2.72E-08	3.82E-07	8.832254
NINJ2	-1.26621	3.249032	-6.96022	2.87E-08	4.00E-07	8.781815
EPHB2	1.356383	1.33456	6.953583	2.93E-08	4.07E-07	8.761387
NDST1	-1.3048	4.272778	-6.94464	3.01E-08	4.16E-07	8.733858
CD97	-1.41213	5.056863	-6.94325	3.02E-08	4.17E-07	8.729596
DSP	2.417409	4.100932	6.93815	3.07E-08	4.22E-07	8.713893
TPPP	-1.92817	2.540812	-6.93567	3.09E-08	4.25E-07	8.706261
ADTRP	-1.26634	1.754066	-6.93544	3.09E-08	4.25E-07	8.705538
PRKDC	1.515333	3.644304	6.930414	3.14E-08	4.31E-07	8.690079
JUND	-1.71592	6.586188	-6.93029	3.14E-08	4.31E-07	8.689700
TOM1L1	1.062408	2.555793	6.928175	3.17E-08	4.33E-07	8.683187
IKBKE	1.0832	2.067961	6.926146	3.19E-08	4.35E-07	8.676941
CHPF2	1.139202	4.222728	6.925776	3.19E-08	4.35E-07	8.675800
TGFBR3	-1.8844	2.6384	-6.91704	3.28E-08	4.46E-07	8.648912
SLC25A10	1.049469	1.23228	6.913503	3.31E-08	4.50E-07	8.638010
INSIG1	-1.18839	4.161244	-6.90925	3.36E-08	4.56E-07	8.624899
PLEKHA6	1.479044	2.277564	6.897613	3.48E-08	4.70E-07	8.589069
MYO1E	1.310425	3.078922	6.893266	3.53E-08	4.75E-07	8.575678
NRN1	-1.62525	2.605324	-6.89318	3.53E-08	4.75E-07	8.575415
SYNM	-1.10684	1.972598	-6.89209	3.54E-08	4.76E-07	8.572052
NDNF	-2.40284	4.781754	-6.89065	3.56E-08	4.77E-07	8.567634
TK1	2.3027	3.629655	6.886338	3.61E-08	4.83E-07	8.554336
ARHGEF16	1.265922	2.140374	6.879902	3.68E-08	4.92E-07	8.534504
SOX4	1.654141	4.336345	6.878407	3.70E-08	4.93E-07	8.529896
DLG5	1.35087	2.216144	6.875622	3.73E-08	4.97E-07	8.521315
HS6ST2	2.561404	1.764155	6.87354	3.75E-08	4.99E-07	8.514898
LPHN2	-1.43229	3.543813	-6.86854	3.81E-08	5.07E-07	8.499473
AQP1	-2.39974	7.335744	-6.86781	3.82E-08	5.07E-07	8.497252
TSPAN6	1.291089	3.348183	6.851853	4.01E-08	5.32E-07	8.448048
DCAF13	1.01652	2.259269	6.849523	4.04E-08	5.35E-07	8.440863
CKAP5	1.001004	3.448997	6.847888	4.06E-08	5.36E-07	8.435823

Gene	logFC	AveExpr	t	P.Value	adj.P.Val	B
PRKCE	-1.20818	2.242711	-6.84058	4.16E-08	5.47E-07	8.413274
PAFAH1B3	1.529012	3.274385	6.839474	4.17E-08	5.48E-07	8.409878
LMNB1	1.652482	2.931143	6.839026	4.18E-08	5.48E-07	8.408496
ILF2	1.139641	6.173679	6.835973	4.22E-08	5.52E-07	8.399081
TNFRSF21	1.588916	4.73615	6.832432	4.27E-08	5.57E-07	8.38816
CES1	-2.39679	5.182582	-6.83094	4.28E-08	5.59E-07	8.383554
SULT1A1	-1.1254	2.011152	-6.82974	4.30E-08	5.60E-07	8.379847
ENAH	1.075852	3.394538	6.827574	4.33E-08	5.62E-07	8.373178
BAALC	-1.27724	1.337808	-6.82731	4.33E-08	5.62E-07	8.372350
GPR133	-1.54486	2.065274	-6.82425	4.38E-08	5.67E-07	8.362936
RSPO4	-1.51525	1.34663	-6.8221	4.40E-08	5.70E-07	8.356304
RACGAP1	1.244239	2.532013	6.816924	4.48E-08	5.79E-07	8.340325
NCAPG	1.509348	1.129176	6.812975	4.53E-08	5.85E-07	8.328139
DPYSL2	-1.34476	5.27161	-6.81055	4.57E-08	5.89E-07	8.320658
XAGE2B	-1.76536	1.322931	-6.80981	4.58E-08	5.90E-07	8.318365
LILRA5	-1.68283	2.39661	-6.80723	4.61E-08	5.92E-07	8.310411
CCBE1	-1.69201	1.827144	-6.7967	4.77E-08	6.10E-07	8.277913
PDLIM1	-1.34491	6.411164	-6.79411	4.81E-08	6.12E-07	8.269916
KANK2	-1.31216	4.337038	-6.79037	4.86E-08	6.17E-07	8.258385
GORAB	1.022814	2.102021	6.790361	4.86E-08	6.17E-07	8.258356
TYMS	1.476494	2.43644	6.789269	4.88E-08	6.19E-07	8.254985
RHPN1	1.165224	1.947986	6.788508	4.89E-08	6.19E-07	8.252636
LIMCH1	-1.54306	4.615361	-6.78799	4.90E-08	6.20E-07	8.251050
MCM7	1.129592	4.139398	6.786391	4.92E-08	6.22E-07	8.246102
C2	-1.44433	4.04685	-6.78498	4.95E-08	6.24E-07	8.241736
HEG1	-1.64471	4.227071	-6.78197	4.99E-08	6.29E-07	8.232463
LMO2	-1.40602	2.949423	-6.78124	5.00E-08	6.30E-07	8.230193
PSAT1	2.184647	1.980909	6.77992	5.02E-08	6.32E-07	8.226128
SLC15A2	-1.5725	2.866682	-6.77787	5.06E-08	6.34E-07	8.219801
SELPLG	-1.45887	4.65082	-6.77781	5.06E-08	6.34E-07	8.219598
DKK2	-1.35133	1.597613	-6.77599	5.09E-08	6.37E-07	8.214004
DTX2	1.091966	2.519987	6.775372	5.10E-08	6.38E-07	8.212088
JDP2	-1.23259	3.100941	-6.77318	5.13E-08	6.42E-07	8.205312
PRKCZ	-1.05791	2.809073	-6.75557	5.42E-08	6.76E-07	8.150937
SLIT3	-1.89767	2.666925	-6.7494	5.53E-08	6.88E-07	8.131873
PLEKHN1	1.089169	0.874304	6.745284	5.60E-08	6.96E-07	8.119174
CDK1	1.660917	2.148554	6.743246	5.63E-08	7.00E-07	8.112876
FFAR4	-1.15265	1.110265	-6.74069	5.68E-08	7.05E-07	8.104973
ESRP1	1.071634	4.016639	6.734973	5.78E-08	7.15E-07	8.087322
FERMT2	-1.06489	3.466526	-6.73188	5.84E-08	7.21E-07	8.077769

Gene	logFC	AveExpr	t	P.Value	adj.P.Val	B
SLC5A3	1.222213	2.111605	6.729676	5.88E-08	7.26E-07	8.070956
PDZD2	-1.56995	2.176049	-6.72879	5.89E-08	7.27E-07	8.068221
NET1	1.438151	4.67728	6.723025	6.00E-08	7.37E-07	8.050404
HMGA1	2.078997	5.565698	6.720489	6.05E-08	7.42E-07	8.042568
DEPDC1B	1.297104	0.865205	6.718914	6.08E-08	7.42E-07	8.037701
AGTR2	-2.44247	2.289692	-6.71524	6.15E-08	7.50E-07	8.026351
HK3	-1.5758	3.035219	-6.7108	6.23E-08	7.59E-07	8.012641
SNRPE	1.1859	4.521443	6.705587	6.34E-08	7.69E-07	7.996516
PKDCC	-1.42537	2.442	-6.70429	6.36E-08	7.71E-07	7.992499
SULT1C4	-1.30257	1.47232	-6.70199	6.41E-08	7.76E-07	7.985399
STEAP1	2.324726	2.568832	6.687368	6.71E-08	8.10E-07	7.940196
PTGFRN	1.46959	3.442582	6.686318	6.73E-08	8.11E-07	7.936950
ALDH2	-1.33412	4.970598	-6.68548	6.75E-08	8.12E-07	7.934348
MMP11	3.019795	1.759107	6.683181	6.80E-08	8.16E-07	7.927251
C5AR1	-1.93166	4.141017	-6.67863	6.89E-08	8.26E-07	7.913189
KIAA0040	-1.01622	4.176147	-6.67727	6.92E-08	8.29E-07	7.908966
ACKR4	-1.17792	1.191054	-6.6751	6.97E-08	8.33E-07	7.902266
SLC9A3R2	-1.32243	5.170501	-6.66664	7.16E-08	8.47E-07	7.876111
CORO2B	-1.24546	1.463721	-6.66656	7.16E-08	8.47E-07	7.875859
PALD1	-1.10409	2.725228	-6.6647	7.20E-08	8.50E-07	7.870095
KIAA1462	-1.37814	3.516092	-6.66181	7.27E-08	8.57E-07	7.861147
FOXM1	1.868834	1.647099	6.660096	7.30E-08	8.61E-07	7.855861
CCNE1	1.471267	1.12588	6.659219	7.32E-08	8.62E-07	7.853149
NKD2	-1.24581	2.520944	-6.64819	7.58E-08	8.89E-07	7.819047
MANEAL	1.193652	1.967298	6.646455	7.62E-08	8.93E-07	7.813667
PTPRH	1.858051	1.022305	6.641735	7.74E-08	9.04E-07	7.799063
PDXDC1	1.01243	3.91649	6.636423	7.87E-08	9.17E-07	7.782629
ACP5	-1.75614	5.90631	-6.62771	8.08E-08	9.38E-07	7.755681
NDRG4	-1.46656	1.448316	-6.62557	8.14E-08	9.43E-07	7.749041
GATA6	-1.84138	2.865681	-6.62482	8.16E-08	9.44E-07	7.746738
RILPL2	-1.01844	2.880307	-6.62364	8.19E-08	9.47E-07	7.743086
SORBS3	-1.38753	3.977165	-6.6139	8.44E-08	9.74E-07	7.712936
GGCT	1.157354	3.943852	6.612769	8.47E-08	9.76E-07	7.70943
SLC50A1	1.439396	4.470093	6.609133	8.57E-08	9.87E-07	7.698179
ADARB1	-1.32936	2.316014	-6.60126	8.78E-08	1.01E-06	7.673805
STEAP3	1.077392	3.69481	6.598931	8.85E-08	1.01E-06	7.666600
ABCA12	1.295134	0.671447	6.595129	8.95E-08	1.02E-06	7.654829
HLTF	1.161165	2.829973	6.581182	9.35E-08	1.06E-06	7.611646
SLC7A7	-1.32356	3.532222	-6.58108	9.35E-08	1.06E-06	7.611317
LDLR	-2.07583	4.740574	-6.57389	9.57E-08	1.09E-06	7.589063

Gene	logFC	AveExpr	t	P.Value	adj.P.Val	B
C8B	-2.04923	1.57299	-6.57351	9.58E-08	1.09E-06	7.587897
PRKG2	-1.05136	1.179522	-6.57023	9.68E-08	1.09E-06	7.577727
TMEM139	-1.66346	2.250866	-6.56683	9.78E-08	1.10E-06	7.567208
UBASH3B	-1.1361	2.000312	-6.56554	9.82E-08	1.11E-06	7.563207
CALU	1.315097	5.669014	6.562313	9.92E-08	1.12E-06	7.553217
METTL7A	-1.75644	4.96493	-6.55972	1.00E-07	1.12E-06	7.545184
PDK4	-2.2016	4.636174	-6.55849	1.00E-07	1.13E-06	7.541370
ASCC3	1.01895	2.448655	6.552565	1.02E-07	1.15E-06	7.523022
BCL2L15	1.612146	1.096467	6.548126	1.04E-07	1.16E-06	7.509273
KIAA0101	1.559725	1.440707	6.542718	1.05E-07	1.18E-06	7.492521
ARHGAP11A	1.283863	1.340436	6.541861	1.06E-07	1.18E-06	7.489866
COL5A2	2.212602	3.888738	6.53855	1.07E-07	1.19E-06	7.479608
C17orf53	1.163026	0.947908	6.53468	1.08E-07	1.20E-06	7.467619
ANGPT1	-1.49637	2.22995	-6.53184	1.09E-07	1.21E-06	7.458823
HYAL1	-2.12014	3.340889	-6.52505	1.11E-07	1.23E-06	7.437795
SLC1A4	1.067913	2.458289	6.5224	1.12E-07	1.24E-06	7.429572
PILRA	-1.41304	3.359708	-6.51991	1.13E-07	1.24E-06	7.421866
AP1S1	1.179643	4.625115	6.513066	1.16E-07	1.27E-06	7.400648
SORBS1	-1.12823	2.367022	-6.5103	1.17E-07	1.28E-06	7.39208
NEDD9	-1.47571	4.695716	-6.50507	1.19E-07	1.29E-06	7.375878
CENPA	1.410361	1.017461	6.503993	1.19E-07	1.30E-06	7.372532
CLDN11	-1.02723	1.064602	-6.49724	1.22E-07	1.32E-06	7.351586
KRT80	2.056247	2.69902	6.496891	1.22E-07	1.32E-06	7.350519
MAOB	-1.63044	3.398369	-6.49596	1.22E-07	1.32E-06	7.347626
SEPP1	-1.46475	4.986727	-6.4947	1.23E-07	1.33E-06	7.343741
FAM105A	-1.54908	3.657437	-6.49458	1.23E-07	1.33E-06	7.343346
TMED3	1.033805	2.460633	6.494302	1.23E-07	1.33E-06	7.342494
HIST1H2AC	1.583713	4.593671	6.490148	1.24E-07	1.34E-06	7.329618
HSPA5	1.018377	7.433783	6.489313	1.25E-07	1.35E-06	7.327030
CBX7	-1.28293	2.728468	-6.48323	1.27E-07	1.37E-06	7.308161
ESYT3	-1.1081	1.708836	-6.48161	1.28E-07	1.37E-06	7.303165
BARX2	1.730205	1.08351	6.476209	1.30E-07	1.39E-06	7.286409
CADM1	-1.56399	3.801659	-6.46423	1.35E-07	1.44E-06	7.249276
HSD17B6	-2.38179	4.207944	-6.46378	1.35E-07	1.44E-06	7.247871
MSR1	-1.90861	4.080041	-6.46331	1.35E-07	1.44E-06	7.246401
SLC52A2	1.148054	4.056837	6.463175	1.35E-07	1.44E-06	7.245997
SPP1	4.033901	4.690224	6.460286	1.37E-07	1.45E-06	7.237038
ARAP3	-1.10909	2.466306	-6.45957	1.37E-07	1.46E-06	7.234809
CDKN3	1.490119	1.470006	6.457869	1.38E-07	1.46E-06	7.229544
MYADM	-2.18307	6.060235	-6.45633	1.38E-07	1.46E-06	7.224782

Gene	logFC	AveExpr	t	P.Value	adj.P.Val	B
TBX4	-1.64322	2.535563	-6.45596	1.38E-07	1.46E-06	7.223616
UGDH	1.415501	4.329762	6.452286	1.40E-07	1.48E-06	7.212232
EGLN3	1.706424	1.853957	6.444643	1.43E-07	1.51E-06	7.188532
FAM83D	1.579709	1.805016	6.444573	1.43E-07	1.51E-06	7.188316
SMIM22	1.7584	3.364001	6.441165	1.45E-07	1.52E-06	7.177746
CREB3L4	1.374856	2.645604	6.440093	1.46E-07	1.53E-06	7.174419
SMAD9	-1.1902	1.68802	-6.43944	1.46E-07	1.53E-06	7.172391
PRG4	-2.03021	1.868767	-6.4256	1.52E-07	1.58E-06	7.129462
HNF4G	1.26453	0.782566	6.42109	1.54E-07	1.60E-06	7.115481
PCSK9	-1.76908	2.104657	-6.41312	1.58E-07	1.64E-06	7.090746
CLSPN	1.112422	0.790946	6.411116	1.59E-07	1.65E-06	7.084540
FAM65A	-1.38807	3.636077	-6.41025	1.60E-07	1.65E-06	7.081867
HN1	1.496824	4.549405	6.40765	1.61E-07	1.66E-06	7.073787
DEPDC1	1.311632	0.822321	6.405102	1.62E-07	1.67E-06	7.065884
RHBDL1	1.17571	0.889684	6.396346	1.67E-07	1.71E-06	7.038719
PMP22	-1.111	5.352607	-6.3954	1.67E-07	1.71E-06	7.035773
BAIAP2L1	1.027402	3.671666	6.388698	1.71E-07	1.74E-06	7.014991
GRAMD3	1.010212	2.508582	6.387939	1.71E-07	1.74E-06	7.012633
WWTR1	-1.05764	3.999348	-6.38502	1.73E-07	1.76E-06	7.003564
PYCRL	1.095678	2.343017	6.384688	1.73E-07	1.76E-06	7.002547
PROS1	-1.31505	4.208853	-6.38307	1.74E-07	1.76E-06	6.997532
S100A3	-1.75902	2.239363	-6.36601	1.84E-07	1.86E-06	6.944597
CD302	-1.13547	2.882639	-6.36341	1.85E-07	1.87E-06	6.936507
CCDC69	-1.43223	4.033874	-6.36192	1.86E-07	1.88E-06	6.931902
CHRDL1	-2.13691	3.553383	-6.361	1.87E-07	1.88E-06	6.92903
SULF1	2.246738	2.990972	6.359886	1.87E-07	1.89E-06	6.92558
PHYHD1	-1.56914	2.513887	-6.35715	1.89E-07	1.90E-06	6.917095
CYP27A1	-1.76629	5.090881	-6.35503	1.90E-07	1.91E-06	6.910523
TSTA3	1.161817	4.144295	6.353173	1.91E-07	1.92E-06	6.904746
CSF3R	-1.72284	3.00625	-6.34845	1.94E-07	1.95E-06	6.890085
TIMP3	-1.4192	2.754326	-6.34803	1.94E-07	1.95E-06	6.888796
GOLPH3L	1.028886	3.839002	6.345844	1.96E-07	1.96E-06	6.881998
SCD5	-1.48585	2.827767	-6.34363	1.97E-07	1.97E-06	6.875118
CCL23	-1.53438	2.121619	-6.34314	1.97E-07	1.97E-06	6.873607
CACYBP	1.001084	3.721622	6.333329	2.03E-07	2.03E-06	6.843153
ALDH1A2	-1.05038	1.074471	-6.33047	2.05E-07	2.04E-06	6.834264
BZW2	1.161754	4.083922	6.328726	2.06E-07	2.05E-06	6.828864
SLC14A1	-1.17983	0.914077	-6.32581	2.08E-07	2.07E-06	6.819805
KIF18B	1.287475	0.875834	6.32538	2.09E-07	2.07E-06	6.818476
RBP4	-2.19571	2.778225	-6.32344	2.10E-07	2.08E-06	6.81246

Gene	logFC	AveExpr	t	P.Value	adj.P.Val	B
SLC5A9	-1.36745	1.063118	-6.31802	2.14E-07	2.11E-06	6.795641
LEPR	-1.27786	2.318442	-6.31758	2.14E-07	2.11E-06	6.79427
KCNAB2	-1.09071	2.463174	-6.31561	2.15E-07	2.12E-06	6.788148
VMP1	1.221833	4.480484	6.313832	2.16E-07	2.13E-06	6.782626
CDCA2	1.150803	0.807364	6.308428	2.20E-07	2.16E-06	6.765848
SIX4	1.22886	1.138487	6.307972	2.20E-07	2.16E-06	6.764432
FKBP11	1.282764	2.475289	6.30643	2.21E-07	2.17E-06	6.759645
PKMYT1	1.038451	0.861826	6.306352	2.21E-07	2.17E-06	6.759403
SH3BP5	-1.08931	2.626479	-6.30578	2.22E-07	2.17E-06	6.757637
LSR	1.074367	5.538913	6.303649	2.23E-07	2.18E-06	6.751012
CPXM1	1.816983	1.899351	6.302474	2.24E-07	2.19E-06	6.747362
FAM64A	1.244251	0.970299	6.294404	2.30E-07	2.24E-06	6.722305
KIAA0907	1.088376	2.498926	6.289113	2.34E-07	2.27E-06	6.705879
GPC3	-2.52852	4.369495	-6.27999	2.41E-07	2.33E-06	6.677534
SPDEF	2.543075	2.544266	6.274154	2.45E-07	2.37E-06	6.659426
FBP1	-1.67406	6.362608	-6.26741	2.50E-07	2.42E-06	6.638499
EFNA3	1.193337	1.052869	6.262968	2.54E-07	2.44E-06	6.624688
MAPK4	-1.04897	0.973066	-6.2599	2.56E-07	2.47E-06	6.615151
PSMG3	1.112245	3.397414	6.257678	2.58E-07	2.48E-06	6.60826
CCRL2	-1.26914	2.346852	-6.2511	2.64E-07	2.53E-06	6.587824
TROAP	1.455152	0.989215	6.250211	2.64E-07	2.53E-06	6.585072
CFL2	-1.22868	2.896098	-6.24951	2.65E-07	2.53E-06	6.582879
MEX3A	1.523719	1.05422	6.245661	2.68E-07	2.56E-06	6.570939
CYB5R3	-1.10821	5.448124	-6.24292	2.70E-07	2.58E-06	6.56241
TOPBP1	1.007725	2.717578	6.240352	2.73E-07	2.59E-06	6.554449
LYSMD1	1.077169	2.10609	6.240259	2.73E-07	2.59E-06	6.554162
BOP1	1.215911	3.115091	6.235488	2.77E-07	2.63E-06	6.539342
DBNDD1	1.198795	2.002005	6.230682	2.81E-07	2.66E-06	6.524415
LMOD1	-1.35336	3.224213	-6.21671	2.94E-07	2.77E-06	6.481025
ATIC	1.160948	3.967752	6.211565	2.98E-07	2.81E-06	6.465035
GTSE1	1.249983	0.94307	6.210513	2.99E-07	2.82E-06	6.461767
DAB2IP	-1.00199	3.554686	-6.21007	3.00E-07	2.82E-06	6.460375
SLC44A2	-1.03206	5.637271	-6.20811	3.02E-07	2.83E-06	6.4543
TPBG	1.286724	2.075991	6.20365	3.06E-07	2.87E-06	6.440447
CAMK2N1	-1.49354	4.108392	-6.19008	3.19E-07	2.97E-06	6.398289
FAM167B	-1.43203	2.663457	-6.18939	3.20E-07	2.98E-06	6.39616
NCAPD2	1.109112	3.311888	6.188878	3.20E-07	2.98E-06	6.394556
ALOX5	-1.4739	4.661695	-6.1888	3.21E-07	2.98E-06	6.394318
ITPRIP	-1.33064	3.368209	-6.18683	3.23E-07	3.00E-06	6.38818
PKM	1.068763	6.827109	6.17727	3.32E-07	3.07E-06	6.358497

Gene	logFC	AveExpr	t	P.Value	adj.P.Val	B
TACC3	1.141933	2.273448	6.161904	3.49E-07	3.22E-06	6.310755
ITGA8	-1.55156	2.633743	-6.15384	3.58E-07	3.29E-06	6.285698
FAM134B	-1.20594	2.505022	-6.14825	3.64E-07	3.33E-06	6.268324
TPSAB1	-1.8804	3.930859	-6.14621	3.67E-07	3.34E-06	6.261987
KCNJ8	-1.19504	3.030463	-6.14512	3.68E-07	3.35E-06	6.258618
NRGN	-2.40704	4.401889	-6.13534	3.79E-07	3.45E-06	6.228224
CITED2	-1.17938	5.877382	-6.12243	3.95E-07	3.57E-06	6.188095
DSG2	1.752575	4.555888	6.122001	3.96E-07	3.57E-06	6.186776
CXorf36	-1.23239	2.183268	-6.12063	3.97E-07	3.59E-06	6.182519
PKP3	1.232614	3.610811	6.119598	3.99E-07	3.60E-06	6.17931
ID4	-2.04314	3.495492	-6.11757	4.01E-07	3.62E-06	6.172994
PAPSS2	-1.28117	5.189558	-6.11712	4.02E-07	3.62E-06	6.171619
C1orf198	-1.16729	4.95915	-6.11235	4.08E-07	3.66E-06	6.156784
SKA1	1.173263	0.878774	6.111217	4.09E-07	3.67E-06	6.153268
FAM3C	1.208818	3.858657	6.108441	4.13E-07	3.69E-06	6.144644
UNC5CL	1.488241	1.343984	6.104995	4.17E-07	3.73E-06	6.133934
CCDC167	1.308051	3.890173	6.091199	4.36E-07	3.87E-06	6.091068
RHOV	2.587878	2.15607	6.091178	4.36E-07	3.87E-06	6.091003
PALMD	-1.32705	2.067699	-6.08843	4.40E-07	3.90E-06	6.082475
ARC	-1.71898	1.24547	-6.08439	4.45E-07	3.94E-06	6.0699
SRD5A3	1.267459	3.260504	6.084212	4.46E-07	3.94E-06	6.069357
TBL1XR1	1.02642	3.739868	6.082773	4.48E-07	3.96E-06	6.064887
CDC45	1.373926	1.130549	6.082039	4.49E-07	3.96E-06	6.062604
THSD1	-1.30585	1.87575	-6.07849	4.54E-07	4.00E-06	6.051567
SDC2	-1.06409	4.024507	-6.07578	4.58E-07	4.03E-06	6.043168
ANXA3	-1.56101	4.452087	-6.06936	4.67E-07	4.10E-06	6.023199
CYS1	-1.47922	2.033794	-6.06779	4.69E-07	4.12E-06	6.018318
PRR11	1.487681	1.588917	6.066206	4.72E-07	4.13E-06	6.013405
KLF9	-1.2761	4.584683	-6.05784	4.84E-07	4.24E-06	5.987408
TNFSF13	-1.00286	3.900337	-6.05738	4.85E-07	4.24E-06	5.985972
MYOZ1	-1.38791	1.611409	-6.05502	4.88E-07	4.26E-06	5.978634
FUT8	1.086169	2.603452	6.033901	5.22E-07	4.52E-06	5.913024
STYK1	1.405104	1.1747	6.030054	5.28E-07	4.56E-06	5.901071
SCNN1B	-1.78985	3.793948	-6.02633	5.35E-07	4.61E-06	5.889503
NOSTRIN	-1.30414	2.259511	-6.02531	5.36E-07	4.62E-06	5.886327
RECK	-1.01167	2.102193	-6.01673	5.51E-07	4.73E-06	5.859674
GPI	1.013786	4.707561	6.012593	5.58E-07	4.77E-06	5.846815
BNIP3	1.224859	3.634895	6.012082	5.59E-07	4.78E-06	5.845227
SMAD7	-1.01465	3.568884	-6.00825	5.66E-07	4.82E-06	5.833317
BTK	-1.05996	2.439224	-6.00662	5.69E-07	4.84E-06	5.828253

Gene	logFC	AveExpr	t	P.Value	adj.P.Val	B
ENC1	1.132896	3.409536	6.002827	5.76E-07	4.89E-06	5.816468
TPSB2	-1.90504	4.040718	-5.9994	5.82E-07	4.94E-06	5.805829
DSCC1	1.049294	1.350176	5.998139	5.84E-07	4.96E-06	5.801903
CHPF	1.055402	4.798638	5.99357	5.93E-07	5.02E-06	5.787706
SCNN1G	-1.68576	2.581923	-5.98819	6.03E-07	5.10E-06	5.77099
SOSTDC1	-2.55612	2.272447	-5.98181	6.15E-07	5.19E-06	5.751179
ARRB1	-1.25289	3.90319	-5.98005	6.19E-07	5.21E-06	5.745693
TNS4	2.479235	1.610129	5.9798	6.19E-07	5.21E-06	5.74492
DUSP8	-1.27921	2.233483	-5.97814	6.22E-07	5.23E-06	5.739773
MMP12	2.935296	1.98845	5.977824	6.23E-07	5.23E-06	5.738781
TSPAN12	-1.47514	4.070296	-5.97507	6.28E-07	5.27E-06	5.730216
SELENBP1	-2.02895	5.909258	-5.97463	6.29E-07	5.27E-06	5.728866
CXCL14	2.949341	3.216177	5.973439	6.32E-07	5.28E-06	5.725157
FKBP1B	-1.124	1.77004	-5.97055	6.37E-07	5.32E-06	5.716185
CARD16	-1.02182	2.93582	-5.96637	6.46E-07	5.38E-06	5.703186
P2RY6	1.563147	1.367984	5.963678	6.51E-07	5.42E-06	5.694829
PGK1	1.037864	6.571496	5.962893	6.53E-07	5.42E-06	5.692392
SNX25	-1.27105	3.625007	-5.96153	6.56E-07	5.44E-06	5.688167
KLF2	-1.67732	4.347369	-5.9575	6.64E-07	5.49E-06	5.675636
ASNS	1.067977	2.346967	5.956741	6.66E-07	5.50E-06	5.673278
CEMIP	1.854294	2.360692	5.950979	6.78E-07	5.60E-06	5.655376
HBEGF	-2.12831	4.268473	-5.94679	6.87E-07	5.65E-06	5.642345
GIMAP4	-1.31104	4.469649	-5.94366	6.94E-07	5.70E-06	5.632648
SMAD6	-1.55418	2.023235	-5.93142	7.21E-07	5.90E-06	5.594613
HKDC1	1.502023	2.038166	5.931384	7.21E-07	5.90E-06	5.594499
GYG2	1.226822	1.217388	5.926656	7.32E-07	5.97E-06	5.579811
TCF7L1	-1.45056	2.552329	-5.92199	7.43E-07	6.04E-06	5.565328
PXMP4	-1.07767	3.118314	-5.9186	7.51E-07	6.09E-06	5.554793
SEMA3B	-1.91958	3.310622	-5.91776	7.53E-07	6.10E-06	5.552168
DNASE2B	-1.25148	1.037884	-5.91617	7.56E-07	6.13E-06	5.547247
SSR4	1.067538	5.127558	5.911931	7.67E-07	6.20E-06	5.534068
C1QA	-1.77291	7.70653	-5.90933	7.73E-07	6.25E-06	5.525981
ADAM28	1.624586	1.494581	5.904385	7.85E-07	6.33E-06	5.510626
SPRY4	-1.28696	3.819958	-5.9007	7.94E-07	6.39E-06	5.49917
SPINT2	1.024667	6.164697	5.899497	7.97E-07	6.41E-06	5.495443
HIST1H2BK	1.495162	5.670763	5.898391	8.00E-07	6.42E-06	5.492009
GRAMD2	-1.62098	2.454591	-5.89834	8.00E-07	6.42E-06	5.491839
GPR115	1.328606	0.740196	5.890608	8.20E-07	6.57E-06	5.467834
ABCG2	-1.04269	1.665915	-5.88858	8.25E-07	6.61E-06	5.461519
ARNTL2	1.868985	1.826898	5.888433	8.26E-07	6.61E-06	5.461077

Gene	logFC	AveExpr	t	P.Value	adj.P.Val	B
C10orf128	-1.08956	2.531698	-5.88787	8.27E-07	6.61E-06	5.45932
MYOC	-1.54408	1.012893	-5.88274	8.41E-07	6.72E-06	5.443384
CKAP2	1.045886	2.27323	5.879016	8.50E-07	6.78E-06	5.431829
EHD2	-1.56912	5.688589	-5.87527	8.61E-07	6.86E-06	5.420188
GREM1	2.071044	1.154771	5.872631	8.68E-07	6.91E-06	5.411998
RNF122	-1.24367	3.137858	-5.86903	8.78E-07	6.97E-06	5.400802
CABYR	1.204786	0.745675	5.868881	8.78E-07	6.97E-06	5.400351
DTYMK	1.056328	2.99608	5.866899	8.84E-07	7.01E-06	5.394196
PBK	1.668109	1.315601	5.864445	8.90E-07	7.06E-06	5.386572
COL5A1	1.983205	3.823646	5.863164	8.94E-07	7.08E-06	5.382594
EPHX3	1.549025	2.194757	5.860288	9.02E-07	7.14E-06	5.373663
EGFL7	-1.31971	4.300986	-5.84956	9.33E-07	7.36E-06	5.340363
HIST1H4I	1.159974	2.787942	5.848868	9.35E-07	7.37E-06	5.3382
FAM184A	-1.07459	1.617884	-5.84771	9.39E-07	7.39E-06	5.334613
TNFSF12	-1.31617	4.227012	-5.84414	9.49E-07	7.46E-06	5.323527
PTN	-1.3779	2.688991	-5.84178	9.56E-07	7.50E-06	5.316185
AFAP1L1	-1.08713	2.646256	-5.8391	9.64E-07	7.53E-06	5.307856
FABP5	-1.65484	3.722836	-5.83878	9.65E-07	7.54E-06	5.306878
GMDS	1.19033	2.333812	5.835227	9.76E-07	7.61E-06	5.295841
RPL39L	2.084047	2.830116	5.829412	9.94E-07	7.73E-06	5.277783
GNG11	-1.74049	4.202542	-5.81862	1.03E-06	7.95E-06	5.244282
CENPH	1.011093	1.71925	5.816712	1.03E-06	7.99E-06	5.238352
TPI1	1.145858	7.041868	5.812626	1.05E-06	8.08E-06	5.225666
OSTC	1.041221	5.488505	5.811232	1.05E-06	8.10E-06	5.22134
AK4	1.600219	1.377142	5.798065	1.10E-06	8.43E-06	5.180465
TIMP1	1.570995	7.394429	5.791738	1.12E-06	8.59E-06	5.160821
RAMP1	1.532981	3.31663	5.790671	1.12E-06	8.61E-06	5.157512
LRRN3	-1.37257	1.400385	-5.78542	1.14E-06	8.75E-06	5.141198
PPAP2B	-1.03484	4.65478	-5.77879	1.17E-06	8.92E-06	5.120629
ADAMTS1	-1.92533	3.799995	-5.77785	1.17E-06	8.94E-06	5.117729
SPC25	1.117487	1.084117	5.777579	1.17E-06	8.94E-06	5.116873
KCNQ3	1.132451	1.197367	5.774973	1.18E-06	9.00E-06	5.108785
TRIP13	1.69855	1.687786	5.771348	1.19E-06	9.10E-06	5.097534
STAC	-1.34253	2.083887	-5.76998	1.20E-06	9.12E-06	5.093299
PCDH12	-1.14873	2.56331	-5.76778	1.21E-06	9.18E-06	5.086466
SLC25A39	1.012538	4.891107	5.762829	1.23E-06	9.31E-06	5.071095
RNASEH2A	1.034039	2.818708	5.758703	1.24E-06	9.40E-06	5.058293
GADD45B	-1.80204	5.73014	-5.75585	1.25E-06	9.47E-06	5.049436
PLEKHO2	-1.05424	4.31002	-5.75488	1.26E-06	9.49E-06	5.046419
KIAA1524	1.12789	1.148632	5.751987	1.27E-06	9.55E-06	5.037452

Gene	logFC	AveExpr	t	P.Value	adj.P.Val	B
C1QTNF6	1.427411	1.496831	5.749128	1.28E-06	9.62E-06	5.02858
PRF1	-1.51374	3.132053	-5.7307	1.36E-06	1.01E-05	4.9714
COL17A1	2.635529	1.612096	5.725678	1.38E-06	1.02E-05	4.955823
MRPL24	1.107537	4.597723	5.722464	1.39E-06	1.03E-05	4.945853
TMEM106C	1.023797	3.953325	5.721297	1.40E-06	1.04E-05	4.942231
CDH1	1.22742	5.814495	5.720327	1.40E-06	1.04E-05	4.939223
POC1A	1.005454	1.685558	5.715676	1.42E-06	1.05E-05	4.924793
DNAJC22	1.149816	0.710724	5.711751	1.44E-06	1.06E-05	4.912619
NFAM1	-1.10033	2.316852	-5.71013	1.45E-06	1.07E-05	4.907583
SKP2	1.232535	2.098601	5.708326	1.46E-06	1.07E-05	4.901995
KLF6	-1.29652	5.897728	-5.70503	1.47E-06	1.08E-05	4.891784
TMEM156	1.302959	1.060947	5.698899	1.50E-06	1.10E-05	4.872758
GAPDH	1.49801	8.513642	5.698733	1.50E-06	1.10E-05	4.872242
CKAP4	1.006592	4.901927	5.697169	1.51E-06	1.11E-05	4.867392
TBX3	-1.33164	2.33999	-5.69317	1.53E-06	1.12E-05	4.855002
BMP5	-1.53245	2.239327	-5.69298	1.53E-06	1.12E-05	4.854414
DNAJC12	1.652306	1.390655	5.684418	1.57E-06	1.14E-05	4.827848
NEXN	-1.09301	2.559835	-5.68178	1.58E-06	1.15E-05	4.819664
VSIG4	-2.0884	5.425213	-5.68002	1.59E-06	1.16E-05	4.814202
SHE	-1.45035	2.199497	-5.67717	1.61E-06	1.17E-05	4.80539
OIP5	1.063537	0.958681	5.676071	1.61E-06	1.17E-05	4.801969
KLF13	-1.14053	4.168261	-5.66341	1.68E-06	1.21E-05	4.762704
TYROBP	-1.88462	6.750632	-5.65291	1.73E-06	1.25E-05	4.730165
C1QTNF7	-1.19754	1.463635	-5.652	1.74E-06	1.25E-05	4.727335
MARCKSL1	1.586175	5.586068	5.651752	1.74E-06	1.25E-05	4.726575
GPRIN2	-1.64878	2.823967	-5.64425	1.78E-06	1.27E-05	4.70333
LY86	-1.32866	3.679815	-5.63971	1.81E-06	1.29E-05	4.689245
FUT3	1.510774	2.250975	5.63939	1.81E-06	1.29E-05	4.68826
ADAM12	1.558237	1.130825	5.636273	1.83E-06	1.30E-05	4.6786
APBB1	-1.16811	2.770444	-5.63564	1.83E-06	1.30E-05	4.676634
SMPDL3B	1.384236	3.280129	5.631983	1.85E-06	1.31E-05	4.665304
TMEM125	-1.30001	5.352113	-5.63026	1.86E-06	1.32E-05	4.659962
SCIMP	-1.03325	1.997362	-5.62839	1.87E-06	1.33E-05	4.654157
NT5E	1.801972	3.170101	5.618746	1.93E-06	1.36E-05	4.624289
EDN1	-1.67306	4.762707	-5.61433	1.96E-06	1.38E-05	4.610619
GPRC5A	-1.8643	6.524847	-5.60902	1.99E-06	1.40E-05	4.594141
GPX8	1.483811	2.546127	5.599668	2.05E-06	1.44E-05	4.565185
COL11A1	2.636398	1.464378	5.5965	2.07E-06	1.45E-05	4.555372
MATN3	-1.51511	2.535166	-5.59629	2.07E-06	1.45E-05	4.554734
FGF2	-1.07838	1.437126	-5.592	2.10E-06	1.46E-05	4.541449

Gene	logFC	AveExpr	t	P.Value	adj.P.Val	B
ALDOA	1.08578	7.088378	5.591492	2.10E-06	1.46E-05	4.539862
MGP	-1.40348	6.543539	-5.58623	2.14E-06	1.48E-05	4.523571
KCNJ5	-1.26777	1.694401	-5.58487	2.15E-06	1.49E-05	4.519343
FAM101B	-1.10676	3.172961	-5.58424	2.15E-06	1.49E-05	4.517389
NCF2	-1.44371	4.666649	-5.58237	2.17E-06	1.50E-05	4.511599
DNASE1L3	-1.91974	1.722794	-5.58148	2.17E-06	1.50E-05	4.508841
FLRT3	-1.82812	3.942723	-5.57417	2.22E-06	1.53E-05	4.486224
TPPP3	-2.53885	4.518425	-5.57216	2.24E-06	1.54E-05	4.479987
ADAM8	1.913851	2.896981	5.571589	2.24E-06	1.54E-05	4.478229
FAM167A	-1.60442	2.502667	-5.56774	2.27E-06	1.55E-05	4.466301
AIM2	2.119208	1.711775	5.567264	2.27E-06	1.56E-05	4.464837
F8	-1.07914	2.240738	-5.56604	2.28E-06	1.56E-05	4.461036
SLC12A7	1.135758	4.210693	5.563946	2.29E-06	1.57E-05	4.454564
RASL12	-1.15149	2.983595	-5.56343	2.30E-06	1.57E-05	4.452979
GSTM5	-1.08538	1.277388	-5.55744	2.34E-06	1.60E-05	4.434429
LYPLA1	1.197447	4.174381	5.556772	2.35E-06	1.60E-05	4.432357
CXCL16	-1.19641	6.015039	-5.55479	2.36E-06	1.61E-05	4.42622
CCL14	-1.04261	0.987319	-5.55355	2.37E-06	1.61E-05	4.422394
BMP2	-1.63888	3.739281	-5.55111	2.39E-06	1.62E-05	4.414841
AHNAK2	1.628192	1.819149	5.549714	2.40E-06	1.63E-05	4.410509
URB1	1.022824	2.174447	5.544312	2.44E-06	1.65E-05	4.393789
GNL3L	1.029765	2.443078	5.543988	2.44E-06	1.65E-05	4.392789
TBX2	-1.43564	2.986481	-5.5372	2.50E-06	1.69E-05	4.371773
CDCA4	1.047002	2.443056	5.52603	2.59E-06	1.74E-05	4.337219
SERPINB5	1.811523	1.025817	5.517562	2.66E-06	1.78E-05	4.311021
CPED1	-1.11286	2.101231	-5.51666	2.66E-06	1.79E-05	4.308224
CCNO	1.257583	1.250585	5.515489	2.67E-06	1.79E-05	4.304608
MRC1	-1.90372	5.407521	-5.51309	2.69E-06	1.80E-05	4.297199
RNF43	1.27512	2.168704	5.51097	2.71E-06	1.81E-05	4.290629
MXRA5	1.787454	3.146142	5.502074	2.79E-06	1.86E-05	4.263116
ADAMTS12	1.315723	1.517058	5.500782	2.80E-06	1.86E-05	4.25912
ITGAV	1.234884	4.466458	5.50007	2.81E-06	1.87E-05	4.256918
MYEOV	1.961356	1.062221	5.497019	2.83E-06	1.88E-05	4.247482
MVB12B	-1.14516	2.516647	-5.4942	2.86E-06	1.90E-05	4.238769
CYP24A1	2.947331	1.637789	5.493428	2.86E-06	1.90E-05	4.236377
LIMD1	-1.05568	2.83812	-5.47896	3.00E-06	1.98E-05	4.191636
PLLP	-1.35741	2.669628	-5.47779	3.01E-06	1.99E-05	4.188043
FA2H	1.596673	1.908727	5.475414	3.03E-06	2.00E-05	4.180684
PKIG	-1.10544	4.469542	-5.47461	3.04E-06	2.00E-05	4.178199
HIST1H2BG	2.305574	1.458202	5.473898	3.05E-06	2.00E-05	4.175998

Gene	logFC	AveExpr	t	P.Value	adj.P.Val	B
MB	1.565518	1.138744	5.465764	3.12E-06	2.05E-05	4.150858
LIMK1	1.038671	3.215955	5.465572	3.13E-06	2.05E-05	4.150264
SLCO4C1	-1.53764	2.225398	-5.45899	3.19E-06	2.08E-05	4.129936
PSD3	1.127898	1.760786	5.451374	3.27E-06	2.13E-05	4.106391
ALOX5AP	-1.81509	5.346607	-5.44809	3.30E-06	2.15E-05	4.096243
ACY3	1.073819	0.884947	5.444866	3.34E-06	2.17E-05	4.086283
LCOR	1.016417	1.747468	5.440376	3.38E-06	2.19E-05	4.072414
CD37	-1.32429	3.371942	-5.43981	3.39E-06	2.20E-05	4.070673
RPS6KA2	-1.01839	4.209705	-5.43718	3.42E-06	2.21E-05	4.062551
C2orf40	-1.71722	1.817667	-5.43576	3.43E-06	2.22E-05	4.058153
STEAP2	1.372054	1.920453	5.43085	3.49E-06	2.25E-05	4.04299
MT-ND6	-3.1243	9.620147	-5.4298	3.50E-06	2.25E-05	4.039753
SVEP1	-1.21365	2.256052	-5.4291	3.51E-06	2.26E-05	4.037579
EVI2B	-1.26603	4.135779	-5.4231	3.57E-06	2.30E-05	4.019054
FAM43A	-1.20645	2.499699	-5.42215	3.58E-06	2.30E-05	4.016115
MT-ND5	-2.35512	9.393213	-5.4211	3.60E-06	2.30E-05	4.012883
NFE2L3	1.120878	2.793289	5.420865	3.60E-06	2.30E-05	4.012155
CHML	1.33209	2.421919	5.418451	3.63E-06	2.32E-05	4.004702
NETO2	1.158247	1.815491	5.415451	3.66E-06	2.33E-05	3.995439
LAPTM4B	1.257399	5.830649	5.414914	3.67E-06	2.34E-05	3.99378
RAD51AP1	1.177953	1.498112	5.409779	3.73E-06	2.37E-05	3.977927
MYOF	1.038213	4.990622	5.406526	3.76E-06	2.39E-05	3.967887
ARHGAP39	1.030088	1.632264	5.404277	3.79E-06	2.41E-05	3.960943
SSTR1	-1.45894	1.429961	-5.40252	3.81E-06	2.42E-05	3.955531
TGFA	1.268715	2.672699	5.397099	3.88E-06	2.45E-05	3.938789
CYGB	-1.0094	3.077173	-5.39578	3.89E-06	2.46E-05	3.93472
CDK5R1	1.094293	1.071704	5.3913	3.95E-06	2.49E-05	3.920894
DERL3	1.4972	2.113122	5.390323	3.96E-06	2.50E-05	3.917877
DRAM1	-1.45554	5.245043	-5.39003	3.96E-06	2.50E-05	3.916975
ALDH1B1	1.091856	3.363954	5.388615	3.98E-06	2.51E-05	3.912609
IGSF10	-1.56939	1.481809	-5.38485	4.03E-06	2.53E-05	3.900984
TLR4	-1.18757	3.114619	-5.38348	4.05E-06	2.54E-05	3.896767
ITPKA	2.102876	1.26279	5.383377	4.05E-06	2.54E-05	3.896446
LDHA	1.181774	6.372671	5.383224	4.05E-06	2.54E-05	3.895974
LGSN	1.748542	1.00161	5.378855	4.11E-06	2.57E-05	3.882494
SERINC2	1.501106	4.374257	5.37843	4.11E-06	2.57E-05	3.881183
LST1	-1.35814	3.488289	-5.37577	4.15E-06	2.59E-05	3.87298
NFIX	-1.57466	4.224589	-5.36876	4.24E-06	2.64E-05	3.851342
MSMO1	-1.11373	5.180005	-5.36796	4.25E-06	2.64E-05	3.848901
SECISBP2L	-1.01025	3.87829	-5.36675	4.27E-06	2.65E-05	3.845148

Gene	logFC	AveExpr	t	P.Value	adj.P.Val	B
C1QB	-1.78118	7.616469	-5.36666	4.27E-06	2.65E-05	3.844884
OLFML1	-1.07287	2.610931	-5.3605	4.35E-06	2.70E-05	3.825882
MAP3K8	-1.2412	3.128183	-5.36	4.36E-06	2.70E-05	3.824327
KPNA2	1.494482	4.064772	5.359858	4.36E-06	2.70E-05	3.823903
MRPL15	1.134714	4.831625	5.359598	4.36E-06	2.70E-05	3.823099
VSIG2	-2.37136	4.292702	-5.35881	4.37E-06	2.71E-05	3.820657
EFNA5	1.236211	2.353978	5.352966	4.45E-06	2.75E-05	3.802653
OASL	-1.12585	2.501499	-5.35288	4.46E-06	2.75E-05	3.802374
NTN4	-1.25197	4.579555	-5.35286	4.46E-06	2.75E-05	3.802312
PLAU	2.485354	4.65223	5.346937	4.54E-06	2.79E-05	3.784065
GJA1	-1.44156	5.820514	-5.34645	4.55E-06	2.80E-05	3.782568
RHOBTB2	-1.3493	4.241809	-5.34395	4.58E-06	2.82E-05	3.774848
MDFI	1.066277	2.569005	5.330813	4.77E-06	2.92E-05	3.734367
SOWAHB	1.165252	1.932283	5.330313	4.78E-06	2.92E-05	3.732829
HEYL	-1.06538	3.066668	-5.32974	4.79E-06	2.93E-05	3.731061
SIGLEC1	-1.29448	2.620563	-5.32935	4.80E-06	2.93E-05	3.729873
FPR2	-1.84229	2.05022	-5.32662	4.84E-06	2.95E-05	3.721442
EMB	1.34444	3.551168	5.323039	4.89E-06	2.98E-05	3.710413
HPCAL1	-1.12278	4.22016	-5.31769	4.98E-06	3.02E-05	3.693941
HIST1H2AE	2.345721	1.558417	5.315384	5.01E-06	3.04E-05	3.686834
APOA1BP	1.029179	5.302701	5.313215	5.05E-06	3.06E-05	3.68015
CD2AP	1.035557	4.002495	5.312754	5.05E-06	3.06E-05	3.678733
TFAP2A	1.195537	0.721539	5.304632	5.18E-06	3.13E-05	3.653719
TUBB6	-1.26469	4.41592	-5.29572	5.33E-06	3.20E-05	3.626263
MMP1	2.930836	2.364115	5.294697	5.35E-06	3.21E-05	3.623127
RHOD	1.195257	3.613683	5.294495	5.35E-06	3.21E-05	3.622504
EFEMP1	-1.40305	5.664108	-5.29409	5.36E-06	3.22E-05	3.621245
POLB	1.068098	2.471794	5.285478	5.50E-06	3.30E-05	3.594747
EPB41L2	-1.00582	3.10525	-5.28531	5.51E-06	3.30E-05	3.594224
NMU	2.058854	1.222493	5.284704	5.52E-06	3.30E-05	3.592366
ID2	-1.06421	4.637253	-5.28299	5.55E-06	3.31E-05	3.587079
CILP2	1.310837	0.911029	5.276572	5.66E-06	3.37E-05	3.567337
ST6GALNAC5	-1.29796	2.398302	-5.27306	5.72E-06	3.40E-05	3.55654
CTSW	-1.35824	2.890929	-5.26931	5.79E-06	3.44E-05	3.544985
SMKR1	1.134936	1.018713	5.263687	5.89E-06	3.50E-05	3.527694
TNXB	-1.86902	2.452787	-5.26107	5.94E-06	3.52E-05	3.519647
VPREB3	1.153933	0.868009	5.260686	5.95E-06	3.53E-05	3.518463
CASQ2	-1.11899	1.024602	-5.26033	5.96E-06	3.53E-05	3.517363
SRPX2	1.79334	2.643944	5.25995	5.96E-06	3.53E-05	3.516198
PPM1H	1.231081	2.028793	5.259183	5.98E-06	3.53E-05	3.51384

Gene	logFC	AveExpr	t	P.Value	adj.P.Val	B
COL4A3	-1.48644	1.991749	-5.25766	6.01E-06	3.55E-05	3.509164
PGM5	-1.19419	1.907364	-5.25386	6.08E-06	3.58E-05	3.497469
CNN1	-1.17071	3.061123	-5.24671	6.22E-06	3.66E-05	3.475491
CD83	-1.63149	4.094654	-5.24423	6.27E-06	3.68E-05	3.467843
FAM83H	1.029266	3.950004	5.240719	6.33E-06	3.72E-05	3.457061
STOM	-1.03435	7.083223	-5.23912	6.37E-06	3.74E-05	3.452141
HCST	-1.25947	3.53304	-5.23813	6.39E-06	3.74E-05	3.449094
PPAPDC1B	1.043771	3.227613	5.223648	6.68E-06	3.90E-05	3.404594
DOCK4	-1.02354	2.543908	-5.21962	6.77E-06	3.94E-05	3.392213
VIM	-1.16323	7.777221	-5.21863	6.79E-06	3.95E-05	3.389175
ARHGAP40	1.297578	0.823955	5.208886	7.00E-06	4.07E-05	3.359244
CRIP2	-1.15662	4.642012	-5.20634	7.05E-06	4.09E-05	3.351431
RXRG	-1.14984	1.124005	-5.2053	7.08E-06	4.10E-05	3.348221
GLB1L2	1.192856	1.865721	5.199969	7.20E-06	4.17E-05	3.331862
CHST15	1.104561	3.377799	5.197873	7.24E-06	4.19E-05	3.325426
MBOAT1	1.069106	2.893375	5.196829	7.27E-06	4.20E-05	3.322218
VASH1	-1.00554	2.535009	-5.19605	7.29E-06	4.21E-05	3.319818
HIST1H1C	1.921011	5.676702	5.195405	7.30E-06	4.22E-05	3.317848
BCL9	1.141337	2.472679	5.194103	7.33E-06	4.23E-05	3.313851
PTGER4	-1.02374	2.940386	-5.19219	7.37E-06	4.25E-05	3.307978
ITGA11	1.596564	1.3946	5.184927	7.54E-06	4.34E-05	3.285682
PRELP	-1.82285	4.406042	-5.18435	7.56E-06	4.35E-05	3.283922
COL1A1	2.761991	6.123165	5.18062	7.65E-06	4.39E-05	3.272463
CST5	-1.03667	0.915104	-5.17935	7.68E-06	4.40E-05	3.268567
CPNE7	1.264603	0.850005	5.177221	7.73E-06	4.42E-05	3.262033
SERPING1	-1.78091	6.805261	-5.17182	7.86E-06	4.48E-05	3.245476
COLEC12	-1.22593	3.682897	-5.16629	8.00E-06	4.55E-05	3.228487
PLEKHH2	-1.26276	1.883329	-5.16231	8.10E-06	4.61E-05	3.216279
TNFRSF18	1.520878	1.572759	5.161916	8.11E-06	4.61E-05	3.215081
RCAN1	-1.28019	3.445825	-5.16151	8.12E-06	4.61E-05	3.213843
COCH	1.11541	0.695547	5.157954	8.21E-06	4.65E-05	3.202931
CNR1	-1.01148	1.231821	-5.15636	8.25E-06	4.67E-05	3.19803
ZFP36	-2.07206	7.493237	-5.14106	8.65E-06	4.87E-05	3.15113
PERP	1.268618	5.463891	5.140311	8.67E-06	4.87E-05	3.148841
PLXNB3	1.008692	0.846302	5.122564	9.17E-06	5.11E-05	3.094462
MYH11	-1.56277	3.642744	-5.12019	9.24E-06	5.14E-05	3.087187
LYPD3	1.933397	1.696675	5.113001	9.45E-06	5.24E-05	3.065176
CCDC64	1.153334	1.353237	5.112548	9.46E-06	5.25E-05	3.063788
B3GNT8	-1.31816	3.166509	-5.11098	9.51E-06	5.27E-05	3.05898
CLEC4E	-1.30305	1.936408	-5.10826	9.59E-06	5.31E-05	3.050667

Gene	logFC	AveExpr	t	P.Value	adj.P.Val	B
CTSH	-1.58646	6.424907	-5.10826	9.59E-06	5.31E-05	3.050652
H2AFX	1.053367	4.062372	5.104469	9.70E-06	5.36E-05	3.039051
FAP	1.42646	1.391983	5.10332	9.74E-06	5.38E-05	3.035536
FGD6	1.014984	2.297493	5.102864	9.75E-06	5.38E-05	3.034138
ADAM9	1.019042	5.099815	5.101898	9.78E-06	5.39E-05	3.031182
ANP32E	1.036291	4.510383	5.101566	9.79E-06	5.40E-05	3.030165
DDIT4	1.571636	5.5043	5.092636	1.01E-05	5.53E-05	3.002836
MAL	-1.36489	2.506408	-5.09021	1.01E-05	5.56E-05	2.995396
WISP1	1.414744	1.533062	5.087887	1.02E-05	5.60E-05	2.988303
FLVCR2	-1.03199	2.583215	-5.0878	1.02E-05	5.60E-05	2.988049
HIST1H2BC	2.026285	2.639905	5.086033	1.03E-05	5.62E-05	2.982633
ETV5	-1.01385	3.856061	-5.08138	1.04E-05	5.70E-05	2.968386
MICALCL	-1.05221	1.878956	-5.08009	1.05E-05	5.72E-05	2.964461
EXOSC5	1.014559	3.109803	5.078221	1.05E-05	5.74E-05	2.958736
TXNDC17	1.235044	3.35877	5.067825	1.09E-05	5.92E-05	2.926944
ITGAL	-1.24267	3.066936	-5.06168	1.11E-05	6.02E-05	2.908154
FOSB	-3.53274	4.358482	-5.05887	1.12E-05	6.06E-05	2.899582
ADAM15	1.066451	4.719549	5.044397	1.17E-05	6.31E-05	2.855347
FAM83B	1.227862	0.698702	5.040463	1.18E-05	6.38E-05	2.843329
TMEM159	1.055622	4.073681	5.03915	1.19E-05	6.40E-05	2.839319
RSPO2	-1.04647	0.98277	-5.03466	1.21E-05	6.47E-05	2.825592
AMOTL1	-1.09897	3.269017	-5.03463	1.21E-05	6.47E-05	2.825507
SNX30	-1.01591	3.230404	-5.02927	1.23E-05	6.56E-05	2.809145
SCN7A	-1.56431	2.660239	-5.02269	1.25E-05	6.67E-05	2.789049
C2orf54	-1.42852	2.866845	-5.01859	1.27E-05	6.74E-05	2.776538
ENO1	1.040701	8.158642	5.011648	1.30E-05	6.87E-05	2.755365
MFAP2	1.100936	2.51881	5.009929	1.30E-05	6.90E-05	2.75012
VCAN	1.644748	3.539343	5.007975	1.31E-05	6.94E-05	2.744159
TBX15	1.052221	0.808969	5.006332	1.32E-05	6.97E-05	2.739146
CITED4	-1.05451	2.39606	-5.00041	1.34E-05	7.08E-05	2.721094
COMP	2.182622	2.054369	4.993478	1.37E-05	7.21E-05	2.699948
ITGA2	1.581725	3.328726	4.991077	1.38E-05	7.25E-05	2.692627
DEPTOR	1.326013	2.800038	4.990669	1.38E-05	7.26E-05	2.691382
CR2	1.09718	0.695534	4.98971	1.39E-05	7.28E-05	2.688458
CP	2.955849	3.27957	4.975634	1.45E-05	7.57E-05	2.645562
CA9	2.427553	1.343497	4.973926	1.46E-05	7.60E-05	2.640358
OPLAH	1.00899	2.245771	4.97367	1.46E-05	7.61E-05	2.639576
C1orf115	-1.39524	3.624377	-4.9723	1.47E-05	7.63E-05	2.635394
AARD	-1.05929	1.190144	-4.96654	1.49E-05	7.75E-05	2.617862
CYB5A	-1.23843	4.411265	-4.96008	1.52E-05	7.88E-05	2.598178

Gene	logFC	AveExpr	t	P.Value	adj.P.Val	B
HEY1	-1.07691	2.580211	-4.95706	1.54E-05	7.94E-05	2.589003
PVRL1	1.253906	2.320504	4.950967	1.57E-05	8.08E-05	2.570442
CST1	2.384742	1.249316	4.948335	1.58E-05	8.14E-05	2.562433
NR4A3	-1.83519	2.316808	-4.93743	1.63E-05	8.38E-05	2.529264
PPIF	1.006068	4.049677	4.935465	1.64E-05	8.42E-05	2.523273
ABLIM3	-1.01633	2.445071	-4.93319	1.66E-05	8.47E-05	2.516351
TMEM47	-1.15303	3.413603	-4.91885	1.73E-05	8.80E-05	2.472763
THEM5	1.06098	1.899152	4.912541	1.76E-05	8.96E-05	2.453576
ARID5A	-1.17408	3.33338	-4.91156	1.77E-05	8.98E-05	2.450588
MAP7D2	1.416599	0.933287	4.908553	1.79E-05	9.04E-05	2.44146
GSTA4	-1.13912	3.668327	-4.90562	1.80E-05	9.11E-05	2.432553
HORMAD1	1.439899	0.811447	4.902613	1.82E-05	9.18E-05	2.423414
ARHGAP30	-1.004	3.485298	-4.8989	1.84E-05	9.28E-05	2.41214
TLCD1	1.223427	2.959175	4.898135	1.85E-05	9.29E-05	2.409813
CYP27B1	1.114046	0.985945	4.890787	1.89E-05	9.49E-05	2.387503
KRT16	2.025398	1.0702	4.883439	1.93E-05	9.69E-05	2.365198
IL4I1	1.356035	1.844295	4.879965	1.95E-05	9.78E-05	2.354655
POSTN	2.066937	4.597033	4.871071	2.01E-05	0.0001	2.327671
CSF3	-3.16637	2.31092	-4.86869	2.02E-05	0.000101	2.320447
BTG2	-1.55118	6.083441	-4.868	2.03E-05	0.000101	2.318352
CXCL2	-2.55196	5.163486	-4.86565	2.04E-05	0.000101	2.311224
SLC51B	-1.28106	1.90387	-4.86471	2.05E-05	0.000102	2.308392
LIFR	-1.36211	3.467396	-4.86303	2.06E-05	0.000102	2.303287
SLPI	-2.06431	9.077481	-4.86297	2.06E-05	0.000102	2.303107
SLC16A5	-1.09953	3.420754	-4.86284	2.06E-05	0.000102	2.302723
GALNT3	1.127634	3.639279	4.860862	2.07E-05	0.000103	2.29671
STRIP2	1.191075	0.950432	4.858888	2.08E-05	0.000103	2.290728
PI16	-1.60646	1.400328	-4.85652	2.10E-05	0.000104	2.283558
CELF2	-1.02261	3.688992	-4.85651	2.10E-05	0.000104	2.28353
FSTL3	-1.27848	4.25836	-4.85538	2.11E-05	0.000104	2.280106
GPER1	-1.15006	1.633718	-4.84953	2.15E-05	0.000106	2.262365
GOLGA7B	1.088745	1.176164	4.846219	2.17E-05	0.000107	2.25233
GALNT14	1.563183	1.231315	4.845081	2.18E-05	0.000107	2.248883
TMEM45B	1.292108	3.16982	4.845038	2.18E-05	0.000107	2.248753
SCG5	1.286522	1.218006	4.843331	2.19E-05	0.000107	2.24358
ERICH2	-1.25341	2.4043	-4.83767	2.23E-05	0.000109	2.226427
WNT9A	-1.01562	2.065404	-4.8355	2.24E-05	0.00011	2.219852
SYT7	1.587299	1.800521	4.83407	2.25E-05	0.00011	2.21553
CFB	1.365613	1.80896	4.832953	2.26E-05	0.00011	2.212148
MMRN1	-1.44836	2.331766	-4.82221	2.34E-05	0.000114	2.179629

Gene	logFC	AveExpr	t	P.Value	adj.P.Val	B
APOLD1	-1.36665	2.843029	-4.8175	2.37E-05	0.000115	2.165362
HLF	-1.40493	2.378562	-4.81137	2.42E-05	0.000117	2.146827
PPP1R14D	2.07092	1.31526	4.80979	2.43E-05	0.000117	2.14205
SRPX	-1.40184	3.280845	-4.80895	2.43E-05	0.000118	2.139496
MFI2	1.72481	1.288279	4.808262	2.44E-05	0.000118	2.137428
KLK11	-2.00664	2.351791	-4.80772	2.44E-05	0.000118	2.135783
SPTBN1	-1.25198	5.509105	-4.8031	2.48E-05	0.00012	2.121815
F2RL3	-1.31572	2.133981	-4.80038	2.50E-05	0.00012	2.113595
EPHB3	1.182248	2.307891	4.79975	2.50E-05	0.000121	2.111687
BAIAP2L2	1.022948	0.7661	4.798257	2.52E-05	0.000121	2.107174
NR4A1	-2.07354	4.212718	-4.79617	2.53E-05	0.000122	2.100864
FOXA1	1.054048	3.4268	4.788474	2.59E-05	0.000124	2.077608
ATOH8	-1.73484	2.919925	-4.78792	2.60E-05	0.000124	2.075921
PTMS	-1.35976	6.720039	-4.78209	2.65E-05	0.000126	2.058317
KPNA7	1.037005	1.024839	4.778765	2.67E-05	0.000128	2.048279
RGS16	-1.39121	4.167251	-4.77727	2.68E-05	0.000128	2.043768
KLF15	-1.3106	2.3628	-4.77646	2.69E-05	0.000128	2.04133
OLFML2A	-1.00706	2.716605	-4.77439	2.71E-05	0.000129	2.03508
MMP14	1.29249	5.614276	4.765397	2.79E-05	0.000132	2.007921
BMP6	-1.41149	2.303572	-4.76463	2.79E-05	0.000132	2.005607
RNF144B	-1.14302	3.822469	-4.76358	2.80E-05	0.000133	2.002438
CDCP1	1.180638	3.274211	4.763056	2.81E-05	0.000133	2.000854
AC105009.1	1.615403	0.884556	4.761908	2.82E-05	0.000133	1.997389
TMPRSS11E	2.239996	1.195429	4.757712	2.85E-05	0.000135	1.984729
GGTLC1	-2.44495	3.236019	-4.75367	2.89E-05	0.000136	1.972551
CDKN2A	1.317251	1.114964	4.751965	2.90E-05	0.000137	1.967396
CXCR2	-1.17573	1.463053	-4.74821	2.94E-05	0.000138	1.956059
GINS4	1.093736	0.853538	4.736756	3.04E-05	0.000143	1.921543
FOXF2	-1.07908	2.417287	-4.73293	3.08E-05	0.000144	1.91001
ID3	-1.17638	4.880141	-4.73178	3.09E-05	0.000144	1.906555
GJB3	1.997205	1.59093	4.73016	3.11E-05	0.000145	1.901668
NKG7	-1.635	3.65944	-4.72734	3.13E-05	0.000146	1.893162
MTURN	-1.23714	3.285921	-4.72147	3.19E-05	0.000148	1.875501
TDRD10	-1.03748	1.168054	-4.71933	3.21E-05	0.000149	1.869059
KLF4	-1.61848	4.565693	-4.71882	3.22E-05	0.000149	1.867521
CTF1	-1.10452	2.629168	-4.71657	3.24E-05	0.00015	1.860732
SLC2A3	-1.24381	3.912408	-4.71492	3.26E-05	0.000151	1.855764
SFTPB	-2.5887	10.53411	-4.71114	3.29E-05	0.000152	1.844402
RNASE1	-1.58117	8.353891	-4.69745	3.44E-05	0.000158	1.803204
SOD3	-1.52748	4.423007	-4.69506	3.46E-05	0.000159	1.796036

Gene	logFC	AveExpr	t	P.Value	adj.P.Val	B
GJA5	-1.28971	3.682447	-4.69398	3.47E-05	0.000159	1.79278
RFX2	-1.29332	1.827385	-4.69377	3.48E-05	0.000159	1.792149
FERMT3	-1.00578	4.062605	-4.68747	3.54E-05	0.000162	1.773196
METTL7B	1.294765	2.083941	4.67821	3.65E-05	0.000166	1.745379
HIST1H4H	2.235726	2.453015	4.677363	3.66E-05	0.000166	1.742834
SLCO2B1	-1.14555	3.957913	-4.67073	3.73E-05	0.000169	1.722901
FCER1G	-1.53045	6.131	-4.66909	3.75E-05	0.00017	1.717999
KLF10	-1.10502	4.818643	-4.66482	3.80E-05	0.000172	1.705177
EPHX4	1.17373	0.923603	4.655835	3.91E-05	0.000176	1.6782
MYRF	-1.66489	2.727713	-4.64861	3.99E-05	0.000179	1.656522
IL20RB	1.896358	1.27513	4.638998	4.11E-05	0.000184	1.627706
HPGDS	-1.07103	2.03197	-4.63717	4.14E-05	0.000185	1.622217
APLN	-1.47014	2.610598	-4.63401	4.18E-05	0.000187	1.612755
SEC14L4	-1.06169	1.704852	-4.63225	4.20E-05	0.000187	1.607479
GCNT3	2.00915	1.213699	4.630412	4.22E-05	0.000188	1.601974
SEMA6D	-1.01096	1.196767	-4.62867	4.25E-05	0.000189	1.596742
KRT6A	2.684677	1.536084	4.610955	4.48E-05	0.000198	1.543712
C7	-1.96668	4.834735	-4.60644	4.55E-05	0.000201	1.530191
CD24	1.316627	5.20935	4.605077	4.57E-05	0.000202	1.526124
SLC7A8	-1.09797	3.044399	-4.60014	4.64E-05	0.000204	1.511348
SESN1	-1.00774	2.89916	-4.59856	4.66E-05	0.000205	1.506638
UBE2S	1.153588	2.218203	4.596385	4.69E-05	0.000206	1.500124
MMP19	-1.67884	2.917031	-4.59308	4.74E-05	0.000208	1.490238
MNDA	-1.26929	3.962312	-4.58873	4.80E-05	0.000211	1.477247
BIK	1.200911	2.003112	4.587941	4.81E-05	0.000211	1.474884
NPR3	-1.21432	2.156878	-4.58774	4.82E-05	0.000211	1.47427
CXCR1	-1.32515	1.217947	-4.57399	5.02E-05	0.000219	1.433196
CPA3	-1.44361	4.449224	-4.5718	5.06E-05	0.00022	1.426657
PPARG	-1.16951	2.992001	-4.57175	5.06E-05	0.00022	1.426527
SFTA3	-1.60801	4.646408	-4.57054	5.08E-05	0.000221	1.422899
SEMA4B	1.286156	4.053543	4.562874	5.20E-05	0.000226	1.400024
SPINK1	3.492613	2.419364	4.562518	5.20E-05	0.000226	1.398962
MT1M	-2.15806	3.503228	-4.56027	5.24E-05	0.000227	1.392247
PGC	-3.42972	6.317071	-4.55753	5.28E-05	0.000229	1.384078
COL1A2	1.679173	6.519397	4.556803	5.30E-05	0.000229	1.38191
FGFR2	-1.43614	2.807166	-4.55607	5.31E-05	0.00023	1.379724
TSKU	1.202204	3.31081	4.555685	5.31E-05	0.00023	1.378575
SAMHD1	-1.04458	5.231763	-4.55235	5.37E-05	0.000232	1.368631
SPOCK1	1.370755	1.062744	4.545472	5.48E-05	0.000236	1.348121
SYNDIG1L	-1.12743	0.978602	-4.54474	5.49E-05	0.000237	1.345947

Gene	logFC	AveExpr	t	P.Value	adj.P.Val	B
KIAA1683	-1.27568	1.132154	-4.54397	5.51E-05	0.000237	1.343658
IFITM2	-1.0167	6.249034	-4.53996	5.58E-05	0.00024	1.331683
APOL3	-1.09233	3.198807	-4.53247	5.71E-05	0.000244	1.30938
FIBIN	-1.34463	3.438392	-4.53128	5.73E-05	0.000245	1.305827
SLC6A8	1.177161	2.142554	4.521286	5.90E-05	0.000252	1.27608
HIST2H2BE	1.153402	3.344612	4.521181	5.91E-05	0.000252	1.275768
SERTAD1	-1.03617	4.187521	-4.51934	5.94E-05	0.000253	1.270296
MMP13	2.651615	1.441074	4.513612	6.04E-05	0.000257	1.253246
CDKN1C	-1.01937	3.123206	-4.51277	6.06E-05	0.000257	1.250736
XKRX	1.235347	1.282069	4.509882	6.11E-05	0.000259	1.242153
LAPTM5	-1.17455	6.992192	-4.50947	6.12E-05	0.000259	1.240939
LEMD1	1.145198	0.902781	4.507553	6.16E-05	0.000261	1.235227
SCGB1A1	-4.26937	7.151252	-4.50313	6.24E-05	0.000264	1.222065
UGT8	1.282774	1.297315	4.498935	6.32E-05	0.000267	1.209606
HIST1H3H	1.573993	1.460366	4.498087	6.34E-05	0.000267	1.207085
VAMP2	-1.29745	4.498006	-4.49716	6.36E-05	0.000268	1.204321
HIST1H3D	1.958673	1.190906	4.482839	6.64E-05	0.000278	1.161793
SYTL2	1.095213	1.957892	4.477967	6.74E-05	0.000282	1.147331
RPL22L1	1.044877	3.039911	4.47036	6.90E-05	0.000286	1.12476
FDCSP	1.540023	1.08709	4.462569	7.06E-05	0.000293	1.101656
CEBPA	-1.03653	3.311958	-4.45458	7.24E-05	0.000299	1.077982
BEAN1	1.011557	0.728318	4.453609	7.26E-05	0.000299	1.075098
TMEM132A	1.189247	2.097414	4.453503	7.26E-05	0.000299	1.074785
APOC1	-1.99419	6.335365	-4.45054	7.33E-05	0.000302	1.066007
CDKN2B	-1.24488	3.24468	-4.45034	7.33E-05	0.000302	1.065407
PKIB	1.413399	1.61412	4.44437	7.47E-05	0.000306	1.047732
CASS4	-1.00911	1.997412	-4.44182	7.53E-05	0.000308	1.040174
ITLN1	-2.22832	1.621409	-4.43527	7.68E-05	0.000314	1.020808
F2RL1	1.090116	2.896574	4.435063	7.68E-05	0.000314	1.020183
ALAS2	-1.00124	0.728428	-4.43194	7.76E-05	0.000317	1.010952
OGN	-1.62213	2.295067	-4.42486	7.93E-05	0.000322	0.990002
GABARAPL1	-1.00308	4.690961	-4.42157	8.01E-05	0.000325	0.980261
CLIC2	-1.06114	4.082804	-4.42065	8.03E-05	0.000325	0.977547
IL20RA	-1.22292	2.0562	-4.41509	8.16E-05	0.00033	0.961116
FKBP10	1.280934	3.922821	4.413203	8.21E-05	0.000331	0.955543
CENPM	1.149751	1.584101	4.40963	8.30E-05	0.000334	0.944985
CA3	-1.18313	1.648947	-4.40761	8.35E-05	0.000336	0.939034
MTHFD2	1.266348	3.498827	4.405884	8.40E-05	0.000337	0.933921
TOX3	1.394298	1.317503	4.395835	8.66E-05	0.000347	0.904255
MMP28	-1.498	3.636119	-4.39447	8.69E-05	0.000348	0.90022

Gene	logFC	AveExpr	t	P.Value	adj.P.Val	B
NPC2	-1.0924	7.457291	-4.39149	8.77E-05	0.00035	0.891448
S100A2	1.928949	2.39541	4.391145	8.78E-05	0.00035	0.890417
GPR110	1.378347	1.627794	4.390317	8.80E-05	0.000351	0.887975
GCLC	1.687879	2.662157	4.389986	8.81E-05	0.000351	0.886998
MUC13	2.162181	1.254374	4.388841	8.84E-05	0.000353	0.883622
HOXB7	1.168227	1.866924	4.386594	8.90E-05	0.000355	0.876993
C4BPA	-2.35269	6.154331	-4.38058	9.07E-05	0.000361	0.859252
B3GNT7	-1.03103	4.04156	-4.37791	9.14E-05	0.000363	0.851401
COL6A3	1.417068	4.700118	4.360498	9.64E-05	0.00038	0.800108
SLC17A9	1.102472	1.309596	4.356774	9.75E-05	0.000384	0.789149
HIF3A	-1.15993	1.520818	-4.35508	9.80E-05	0.000386	0.784166
SCTR	-1.29201	2.028417	-4.34786	0.0001	0.000392	0.762939
LMO7	-1.16226	4.827344	-4.34649	0.000101	0.000394	0.758904
TUBA1A	-1.07074	5.816912	-4.34644	0.000101	0.000394	0.758759
CILP	1.343703	1.272103	4.344668	0.000101	0.000396	0.75354
IGFBP3	1.610259	4.573452	4.334952	0.000104	0.000406	0.724986
NME4	1.023555	3.835991	4.334847	0.000104	0.000406	0.724678
ELOVL6	1.000315	1.504447	4.334755	0.000104	0.000406	0.724407
SYT12	1.184288	0.636581	4.33397	0.000104	0.000406	0.722101
KRT15	1.536587	1.558841	4.332206	0.000105	0.000408	0.716919
SIX1	1.300717	1.271925	4.32675	0.000107	0.000414	0.700898
ALOX15B	-1.82071	4.289657	-4.32673	0.000107	0.000414	0.70084
GYLTL1B	1.145103	1.985349	4.326113	0.000107	0.000414	0.699027
IL6	-2.39357	3.30403	-4.31945	0.000109	0.000422	0.679475
CERCAM	1.112458	2.592922	4.319339	0.000109	0.000422	0.679143
LYPD1	1.071519	0.709725	4.313551	0.000111	0.000428	0.662165
IL7R	-1.4363	4.375183	-4.31338	0.000111	0.000429	0.661656
FPR1	-1.4889	3.70216	-4.30645	0.000114	0.000436	0.641337
NFKBIA	-1.25986	7.088008	-4.30294	0.000115	0.00044	0.631054
FCN1	-1.15031	2.535731	-4.30213	0.000115	0.000441	0.628677
GNLY	-1.27921	2.089322	-4.29726	0.000117	0.000446	0.614423
SOX18	-1.26824	2.43091	-4.29686	0.000117	0.000447	0.613241
EMP1	-1.464	5.176371	-4.29342	0.000118	0.00045	0.603155
CEBPD	-1.05902	5.917583	-4.27846	0.000124	0.000469	0.559368
RAC3	1.249868	2.097716	4.275736	0.000125	0.000472	0.551415
KLK7	-1.16435	0.860864	-4.27163	0.000126	0.000477	0.53942
PCDH17	-1.06171	2.527483	-4.26482	0.000129	0.000486	0.519504
DUSP4	1.525537	2.620037	4.263965	0.000129	0.000487	0.517007
RGS5	-1.03443	4.181747	-4.25748	0.000132	0.000495	0.498067
DCN	-1.04618	5.84227	-4.24751	0.000136	0.000509	0.468952

Gene	logFC	AveExpr	t	P.Value	adj.P.Val	B
C2CD4A	1.00127	0.910842	4.235016	0.000141	0.000527	0.432528
KCNJ15	-1.25196	3.089246	-4.22401	0.000146	0.000542	0.400473
HIST3H2A	1.668916	2.038658	4.22398	0.000146	0.000542	0.400375
CX3CL1	-1.67072	4.245198	-4.21963	0.000148	0.000548	0.387713
C10orf10	-1.52664	6.042195	-4.21391	0.00015	0.000557	0.371061
PDGFRL	1.156246	2.287129	4.213553	0.00015	0.000557	0.370025
EMP3	-1.07658	4.956291	-4.21259	0.000151	0.000559	0.367237
FZD4	-1.16161	3.062818	-4.2088	0.000152	0.000564	0.356209
MMP9	2.128526	3.791052	4.204723	0.000154	0.00057	0.344342
SLC25A25	-1.20935	3.177773	-4.20257	0.000155	0.000573	0.338076
PLOD2	1.613716	3.065681	4.19662	0.000158	0.000582	0.320795
C1QC	-1.33874	7.426649	-4.18925	0.000162	0.000593	0.29939
POMK	1.398623	1.41983	4.188469	0.000162	0.000594	0.297119
PER1	-1.03366	4.422457	-4.18227	0.000165	0.000603	0.27914
SLC34A2	-1.55201	8.548822	-4.181	0.000166	0.000605	0.275433
COL4A4	-1.07729	2.378329	-4.1791	0.000167	0.000608	0.269923
PFN2	1.485798	3.847731	4.177648	0.000167	0.00061	0.265718
AOC1	1.420873	1.021267	4.167181	0.000173	0.000626	0.23537
SLC7A5	1.578178	3.293179	4.163676	0.000175	0.000632	0.225213
RASL11A	-1.087	2.769391	-4.16296	0.000175	0.000633	0.223146
LAMB3	1.355744	6.145943	4.151261	0.000181	0.000652	0.189266
CDC42EP2	-1.0921	3.155924	-4.15056	0.000182	0.000654	0.187223
SIGLEC14	-1.03255	1.989264	-4.13988	0.000187	0.000673	0.156332
MET	1.307542	4.652182	4.136731	0.000189	0.000678	0.147244
TRIM2	1.283946	3.03712	4.133151	0.000191	0.000684	0.136898
PTK6	1.182774	2.397083	4.126624	0.000195	0.000696	0.118045
FGFBP2	-1.69558	1.58243	-4.12358	0.000197	0.000702	0.109252
C16orf89	-1.89936	5.906272	-4.11697	0.000201	0.000713	0.090182
HLA-DRB1	-1.21302	9.614726	-4.11657	0.000201	0.000714	0.089015
PTPN13	-1.07542	3.276034	-4.11583	0.000201	0.000715	0.086891
S100A12	-1.48399	1.928448	-4.11064	0.000205	0.000725	0.071917
ZBTB16	-1.25324	1.79653	-4.09626	0.000214	0.000753	0.030481
SPTB	1.175744	0.860978	4.095234	0.000214	0.000755	0.027532
MAFF	-1.27224	3.333081	-4.09154	0.000217	0.000761	0.016904
UNC13B	-1.08393	4.385751	-4.07889	0.000225	0.000785	-0.01948
ASPH	1.236048	4.009333	4.067704	0.000232	0.000808	-0.05164
JUNB	-1.08677	7.102188	-4.06727	0.000233	0.000809	-0.05289
IRX1	-1.11542	1.336178	-4.06437	0.000235	0.000816	-0.06121
ATP13A4	-1.61422	3.053616	-4.06285	0.000236	0.000819	-0.06557
DUSP1	-1.26019	8.080833	-4.06279	0.000236	0.000819	-0.06576

Gene	logFC	AveExpr	t	P.Value	adj.P.Val	B
COL7A1	1.151215	0.830516	4.049045	0.000246	0.00085	-0.10518
ALDH3B2	1.00282	0.984141	4.045061	0.000249	0.000858	-0.1166
DNTTIP1	1.180156	4.27517	4.030559	0.00026	0.000892	-0.15813
FCGR3A	-1.27495	5.650071	-4.02188	0.000266	0.000912	-0.18295
EIF4EBP1	1.226265	4.29639	4.020895	0.000267	0.000915	-0.18577
SCEL	-1.59086	4.358847	-4.0199	0.000268	0.000917	-0.1886
ZBED2	-1.4164	2.209481	-4.01889	0.000269	0.000919	-0.19151
MUC20	1.33035	2.08225	4.016095	0.000271	0.000926	-0.19949
HHIPL2	1.636337	0.860401	4.015441	0.000272	0.000927	-0.20136
MROH6	1.159693	1.965387	4.005185	0.00028	0.000951	-0.23065
IL23A	1.373856	1.164685	4.001493	0.000283	0.00096	-0.24119
B4GALNT4	1.123034	0.685909	4.001241	0.000283	0.000961	-0.24191
GPX2	2.627002	2.104365	4.000635	0.000284	0.000962	-0.24364
EGR1	-1.87146	6.467145	-3.99439	0.000289	0.000976	-0.26144
HIST1H2AD	1.894108	1.108238	3.989668	0.000293	0.000988	-0.27491
CXCL17	-1.95126	6.497815	-3.98912	0.000294	0.00099	-0.27648
SCARA3	-1.03534	2.641622	-3.98583	0.000296	0.000999	-0.28584
SRGN	-1.19056	7.4489	-3.98005	0.000302	0.001013	-0.3023
CEACAM1	1.153416	2.290903	3.973102	0.000308	0.001031	-0.32209
HIST1H4K	1.038507	0.741121	3.972908	0.000308	0.001032	-0.32264
ADAMDEC1	1.447193	1.30803	3.970954	0.00031	0.001037	-0.3282
NAPSA	-1.71589	8.228573	-3.96538	0.000315	0.001052	-0.34406
HCAR2	-1.22231	2.312118	-3.96523	0.000315	0.001053	-0.34447
GPR116	-1.46762	5.535346	-3.9588	0.000321	0.00107	-0.36276
ATF3	-1.47615	4.091099	-3.95628	0.000323	0.001077	-0.36991
FOXA2	-1.30709	3.347307	-3.9456	0.000334	0.001108	-0.40025
CD27	1.083431	2.042695	3.944426	0.000335	0.001111	-0.40356
RAB27B	1.007118	2.652396	3.942898	0.000337	0.001115	-0.4079
PRRX2	1.160718	1.478267	3.941831	0.000338	0.001117	-0.41093
TP53INP2	-1.10748	3.689977	-3.94025	0.000339	0.001121	-0.41542
LCN2	1.782294	4.433582	3.936639	0.000343	0.00113	-0.42565
GALNT6	1.281508	2.454805	3.935177	0.000344	0.001134	-0.42979
FNDC1	1.421935	1.77383	3.933801	0.000346	0.001138	-0.43369
HTR3A	1.288186	0.678835	3.932181	0.000347	0.001142	-0.43828
CLEC12A	-1.00727	1.840576	-3.92876	0.000351	0.001152	-0.44796
COL15A1	1.29472	3.052143	3.92568	0.000354	0.001161	-0.4567
VAMP5	-1.02852	5.609815	-3.92085	0.000359	0.001176	-0.47037
HLA-DPB1	-1.08667	7.512863	-3.91187	0.000369	0.001205	-0.49578
HIST1H2AI	1.867134	1.043273	3.911428	0.000369	0.001206	-0.49702
SFRP5	-1.38308	1.503978	-3.91081	0.00037	0.001207	-0.49875

Gene	logFC	AveExpr	t	P.Value	adj.P.Val	B
LRRC15	1.383782	1.648806	3.897593	0.000385	0.001252	-0.53611
BEX5	-1.10869	2.331865	-3.89502	0.000387	0.001261	-0.54338
SLC16A14	1.234818	1.491857	3.892902	0.00039	0.001267	-0.54934
ICAM1	-1.49258	6.700664	-3.87244	0.000414	0.001332	-0.60701
CHI3L2	-1.36515	3.276194	-3.86828	0.000419	0.001347	-0.61872
KRTCAP3	1.151616	4.129446	3.860624	0.000429	0.001373	-0.64026
SERPINE2	1.104717	1.700947	3.860436	0.000429	0.001374	-0.64079
CD74	-1.11713	10.30389	-3.85806	0.000432	0.001382	-0.64747
ELN	-1.03199	4.081313	-3.85183	0.00044	0.001405	-0.66496
MUC4	1.35357	1.378149	3.848106	0.000445	0.001419	-0.67543
FAM101A	1.22131	1.076508	3.844581	0.000449	0.001433	-0.68533
ALDH1A1	-1.5281	5.492809	-3.84164	0.000453	0.001441	-0.69357
SLC7A11	1.444997	1.280746	3.830395	0.000468	0.001485	-0.72511
SCD	-1.11312	6.458344	-3.82677	0.000473	0.001498	-0.73525
KCNK1	1.058967	3.012486	3.807139	0.000501	0.001572	-0.79019
REEP6	1.070945	1.835665	3.807031	0.000501	0.001572	-0.79049
IER2	-1.015	4.875271	-3.80558	0.000503	0.001578	-0.79455
GJB5	1.070513	0.846924	3.793598	0.000521	0.001624	-0.828
HIST1H2AG	1.358052	0.851576	3.792592	0.000523	0.001628	-0.83081
SORL1	1.034973	2.781845	3.785903	0.000533	0.001657	-0.84946
CLDN10	1.56374	1.114627	3.770942	0.000557	0.00172	-0.89113
IL2RA	1.109493	1.671242	3.769763	0.000559	0.001725	-0.89441
HGD	1.288851	1.198506	3.759048	0.000576	0.001773	-0.92421
POU2AF1	1.079599	1.225948	3.756975	0.00058	0.001782	-0.92997
ARSE	1.326393	2.327468	3.746072	0.000598	0.001829	-0.96024
PHLDA2	1.520999	3.410724	3.74473	0.000601	0.001834	-0.96396
ERV3-1	1.067514	2.429022	3.736409	0.000615	0.001876	-0.98703
PLA2G4A	1.266777	2.76959	3.73468	0.000618	0.001883	-0.99182
GBP4	-1.18857	3.80153	-3.72991	0.000627	0.001907	-1.00504
CNFN	1.018345	1.055251	3.729092	0.000629	0.001911	-1.0073
SLC16A3	1.06685	3.671197	3.721985	0.000642	0.001944	-1.02697
CPM	-1.14723	4.669068	-3.72191	0.000642	0.001944	-1.02719
VSTM2L	1.548132	3.650701	3.71755	0.00065	0.001964	-1.03924
SBK1	1.059804	1.424469	3.713423	0.000658	0.001982	-1.05064
DCBLD2	1.19797	3.236025	3.706964	0.00067	0.002011	-1.06849
HIST1H2BH	1.775197	1.025214	3.705337	0.000673	0.002019	-1.07298
CECR1	-1.10403	4.362274	-3.70524	0.000673	0.002019	-1.07324
TMPRSS2	-1.48086	4.171817	-3.70305	0.000678	0.002029	-1.0793
HLA-DPA1	-1.01568	6.933027	-3.70201	0.00068	0.002035	-1.08215
LOXL2	1.249757	2.724464	3.701428	0.000681	0.002037	-1.08377

Gene	logFC	AveExpr	t	P.Value	adj.P.Val	B
HRCT1	-1.06242	1.797955	-3.70051	0.000683	0.002041	-1.08631
SCARA5	-1.55048	1.953849	-3.69939	0.000685	0.002047	-1.08939
SELE	-1.45581	1.336162	-3.69516	0.000693	0.00207	-1.10107
B3GNT6	1.661607	0.969335	3.693874	0.000696	0.002076	-1.1046
MS4A4A	-1.01287	3.748507	-3.69197	0.0007	0.002084	-1.10985
WFDC3	1.038736	0.828694	3.686482	0.000711	0.002111	-1.12497
BASP1	1.390971	4.253768	3.673635	0.000738	0.002183	-1.16033
IL1RL1	-1.71832	2.099377	-3.67337	0.000738	0.002185	-1.16106
CYR61	-1.26138	6.837396	-3.65775	0.000772	0.002275	-1.20397
DIO2	1.057286	0.889194	3.654392	0.00078	0.002295	-1.21317
PRRX1	1.073027	2.469763	3.646068	0.000798	0.002346	-1.23599
SOCS3	-1.52597	6.422764	-3.64237	0.000807	0.002367	-1.24611
PDPN	-1.01155	3.854075	-3.64014	0.000812	0.00238	-1.25223
HAS1	-1.2304	1.096447	-3.62923	0.000838	0.002445	-1.28207
SPRR1B	1.598588	0.886035	3.624494	0.000849	0.002474	-1.29502
AADAC	-1.49479	2.2147	-3.62407	0.00085	0.002476	-1.29617
CD79A	1.579756	2.659804	3.617841	0.000866	0.002517	-1.31319
MAOA	-1.11684	4.944924	-3.61642	0.000869	0.002525	-1.31707
RCN3	1.298316	3.292523	3.614555	0.000874	0.002538	-1.32216
LY6D	1.289771	0.70091	3.610011	0.000885	0.002565	-1.33455
CTGF	-1.15056	7.06349	-3.60317	0.000903	0.00261	-1.3532
QPCT	1.350742	2.517687	3.600238	0.00091	0.002627	-1.36119
HOPX	-1.33924	4.942194	-3.56945	0.000994	0.002832	-1.44486
ID1	-1.18067	5.801369	-3.55924	0.001023	0.002898	-1.47253
CA2	-1.55811	4.376395	-3.5494	0.001052	0.002968	-1.49916
HLA-DRA	-1.11305	10.54703	-3.54916	0.001053	0.002969	-1.49981
FCGR3B	-1.20154	1.551378	-3.54811	0.001056	0.002976	-1.50266
CCL24	-1.48147	2.328714	-3.54595	0.001063	0.00299	-1.50848
DMBT1	-1.85096	4.513419	-3.54431	0.001068	0.003001	-1.51292
SGK1	-1.00677	4.159129	-3.54394	0.001069	0.003003	-1.51392
C11orf86	1.123802	0.579405	3.539033	0.001084	0.003041	-1.52718
NR0B2	-1.16768	1.614283	-3.53783	0.001087	0.00305	-1.53043
SPNS2	-1.06751	3.247782	-3.52781	0.001119	0.003122	-1.55745
IL33	-1.46605	4.257871	-3.52162	0.001139	0.003168	-1.57416
PANX2	1.097508	1.437362	3.520845	0.001141	0.003173	-1.57624
UBD	1.430429	2.360454	3.518905	0.001147	0.003187	-1.58146
RRAD	-1.46206	4.221715	-3.50866	0.001181	0.003267	-1.60904
THY1	1.267166	3.082686	3.507758	0.001184	0.003274	-1.61146
TNNT1	1.49772	1.723206	3.507386	0.001185	0.003277	-1.61246
HIST1H4E	2.280064	1.273833	3.501035	0.001207	0.003325	-1.62952

Gene	logFC	AveExpr	t	P.Value	adj.P.Val	B
HIST1H2BJ	1.528527	1.413281	3.4991	0.001213	0.00334	-1.63472
FNDC4	1.070393	1.996347	3.495383	0.001226	0.003369	-1.6447
LRRN4	-1.51347	3.133258	-3.48694	0.001256	0.003438	-1.66736
C2CD4B	-1.0151	1.580389	-3.4863	0.001258	0.003443	-1.66905
HILPDA	1.102787	2.824886	3.484716	0.001264	0.003456	-1.6733
HIST1H3E	1.102712	1.133495	3.484451	0.001265	0.003457	-1.67401
SLAMF7	1.203736	2.549638	3.468288	0.001324	0.003595	-1.71727
SGPP2	1.196497	3.948788	3.468096	0.001324	0.003596	-1.71779
TCN1	1.941916	1.13246	3.465656	0.001333	0.003619	-1.72431
EGR2	-1.15939	2.56377	-3.46484	0.001336	0.003626	-1.72648
RAB11FIP1	-1.04166	4.539459	-3.45842	0.001361	0.003683	-1.74364
KDM6B	-1.0735	3.347651	-3.45238	0.001384	0.00374	-1.75975
TXNRD1	1.203828	5.096569	3.451088	0.001389	0.003752	-1.76319
CCL19	1.542136	3.379242	3.447672	0.001403	0.003783	-1.7723
PAEP	2.191995	1.415197	3.442387	0.001424	0.003834	-1.78638
NCCRP1	1.225093	0.878685	3.441711	0.001426	0.00384	-1.78818
FOLR1	-1.58965	6.394936	-3.43552	0.001451	0.003896	-1.80465
TRIM31	1.019475	0.691088	3.431761	0.001467	0.003934	-1.81465
FOS	-1.43522	6.935768	-3.43136	0.001468	0.003937	-1.81571
CFTR	-1.06404	1.877036	-3.41071	0.001556	0.004135	-1.87052
HIST1H2AM	1.515985	0.817534	3.408032	0.001567	0.004161	-1.87763
S100P	2.561878	3.502064	3.40787	0.001568	0.004162	-1.87806
NDUFA4L2	-1.04156	2.904155	-3.40544	0.001579	0.004181	-1.88448
HIST2H2BF	1.038026	0.687867	3.396503	0.001619	0.004273	-1.90814
CT83	1.693999	0.940994	3.389458	0.001651	0.004349	-1.92676
NTM	-1.32926	2.157978	-3.38692	0.001663	0.004374	-1.93346
AKR1B10	2.528161	1.390027	3.383788	0.001677	0.004405	-1.94173
BCL2A1	-1.07142	3.762458	-3.37872	0.001701	0.004458	-1.9551
SYT13	1.007991	0.734457	3.376899	0.00171	0.004478	-1.9599
MMP7	1.807019	3.725233	3.369721	0.001744	0.004559	-1.97881
TFF1	2.524399	1.363348	3.355102	0.001816	0.00472	-2.01726
RTN4RL2	1.162321	1.352067	3.343916	0.001874	0.00485	-2.04662
HIST1H2BF	1.459823	0.766865	3.34373	0.001875	0.004851	-2.04711
SMOC1	1.298601	1.085646	3.321875	0.001992	0.005105	-2.10432
AHNAK	-1.12865	6.350703	-3.31022	0.002057	0.005253	-2.13474
CCL2	-1.62253	4.926027	-3.29547	0.002142	0.005447	-2.17317
KRT17	2.176162	3.252888	3.293114	0.002156	0.005477	-2.1793
MT-ND1	-1.91462	10.3436	-3.28868	0.002183	0.005534	-2.19082
PCP4	1.141733	0.855242	3.282171	0.002222	0.005626	-2.20773
MZB1	1.3792	2.643939	3.276262	0.002259	0.005705	-2.22306

Gene	logFC	AveExpr	t	P.Value	adj.P.Val	B
GZMH	-1.00876	2.406784	-3.2719	0.002286	0.005765	-2.23438
TREM1	-1.09259	4.01572	-3.2684	0.002308	0.005816	-2.24344
SLC16A9	1.079907	1.265385	3.243423	0.002472	0.006164	-2.30798
SLC22A3	-1.15499	3.245559	-3.24046	0.002492	0.006209	-2.31563
CEACAM5	2.374121	3.071193	3.240336	0.002493	0.006209	-2.31594
MT-CYB	-1.58607	10.60218	-3.21261	0.002689	0.006632	-2.38724
GAL	1.016966	0.618632	3.196664	0.002808	0.00689	-2.42807
POF1B	1.103319	1.527247	3.192977	0.002836	0.006947	-2.4375
NFKBIZ	-1.18957	3.45758	-3.17935	0.002943	0.00718	-2.4723
SERPINA1	-1.18404	7.459261	-3.17914	0.002945	0.007182	-2.47283
HIST1H1D	1.767006	0.92812	3.16979	0.003021	0.007354	-2.49665
PKP2	1.040669	1.632188	3.166031	0.003052	0.007419	-2.50621
IVL	1.481617	0.930866	3.158331	0.003116	0.007561	-2.52578
CACNG4	-1.1674	2.412927	-3.15248	0.003166	0.007663	-2.54063
IGLL5	1.542753	3.84303	3.138047	0.003292	0.007938	-2.57721
HIST1H2BO	1.547075	0.861402	3.135918	0.003311	0.007973	-2.5826
RND1	-1.54585	3.465437	-3.1272	0.003389	0.008124	-2.60463
PRAME	1.333223	0.693219	3.124355	0.003415	0.008179	-2.61181
ASPN	1.069189	3.350613	3.117997	0.003474	0.008302	-2.62785
HIST1H1E	2.249483	1.23652	3.115241	0.0035	0.008354	-2.6348
AGR2	1.630482	5.704757	3.111245	0.003538	0.008427	-2.64486
APOBEC3B	1.021189	1.494929	3.093501	0.003711	0.008786	-2.68947
LAMC2	1.122763	5.280236	3.082355	0.003823	0.009029	-2.71741
S100A7	1.274391	0.667254	3.075096	0.003898	0.009187	-2.73557
FOXA3	1.072854	0.998645	3.067686	0.003976	0.009345	-2.75409
FGL1	1.358703	0.771215	3.043248	0.004244	0.009898	-2.81496
KRT81	1.564807	0.909832	3.036496	0.00432	0.010056	-2.83173
CDA	1.669313	2.918442	2.99156	0.004867	0.011173	-2.94274
AQP9	-1.03676	2.778342	-2.97078	0.00514	0.011724	-2.99373
TMEM45A	1.126925	2.244714	2.966849	0.005194	0.011814	-3.00336
MT-ND2	-1.92875	10.2882	-2.96377	0.005236	0.011898	-3.01089
TESC	1.663665	2.947498	2.960829	0.005277	0.011977	-3.01808
CXCL3	-1.2883	2.211934	-2.95706	0.005329	0.012084	-3.02729
LTF	1.662752	3.348664	2.955605	0.00535	0.012121	-3.03084
FGB	2.495992	1.401522	2.953853	0.005374	0.012164	-3.03511
PMAIP1	1.174825	2.581296	2.928529	0.005743	0.012907	-3.09674
HIST1H2BE	1.484934	0.854271	2.924195	0.005808	0.013043	-3.10725
FBLN1	-1.05481	5.21343	-2.91359	0.005971	0.013355	-3.13294
TNC	1.259405	4.216809	2.888602	0.006372	0.014116	-3.19322
VSIG1	1.610113	1.464173	2.886803	0.006402	0.014168	-3.19755

Gene	logFC	AveExpr	t	P.Value	adj.P.Val	B
BARX1	1.045376	0.562829	2.883434	0.006458	0.01427	-3.20565
MT-ND4	-1.66134	11.11885	-2.87991	0.006517	0.014384	-3.21411
UCHL1	1.565296	2.390284	2.872739	0.00664	0.014618	-3.23132
ERN2	1.137141	0.901984	2.866723	0.006744	0.01482	-3.24574
TMSB15A	-1.10295	1.501431	-2.8509	0.007025	0.015335	-3.28355
CLU	-1.03916	5.564252	-2.84449	0.007142	0.015567	-3.29885
CD163	-1.06153	4.723509	-2.84168	0.007194	0.015667	-3.30553
HIST2H2AC	1.739733	1.460164	2.837265	0.007276	0.015832	-3.31604
SAA1	1.357038	2.556698	2.836054	0.007299	0.015879	-3.31892
HIST1H4D	1.708717	0.932225	2.829704	0.007419	0.0161	-3.33401
MT-ND3	-1.09373	10.74038	-2.82467	0.007516	0.016273	-3.34595
MT-ND4L	-1.27126	9.667654	-2.81165	0.007771	0.016752	-3.37679
MUC5B	1.946364	2.025	2.800912	0.007988	0.017136	-3.40215
MUC21	1.700174	2.106606	2.793377	0.008143	0.017428	-3.41991
BCAS1	1.159187	1.218227	2.778058	0.008467	0.017999	-3.45591
HIST1H1B	1.773849	0.903892	2.773662	0.008563	0.018172	-3.46622
HIST1H3C	1.457249	0.733628	2.768004	0.008687	0.018399	-3.47947
HIST1H3B	1.681866	0.853195	2.759136	0.008885	0.018759	-3.50021
WFDC2	1.100208	6.362127	2.746938	0.009164	0.019241	-3.52865
SPRR2D	1.011068	0.62919	2.742256	0.009273	0.019435	-3.53955
FCER1A	-1.09634	2.691608	-2.74096	0.009303	0.019489	-3.54256
HLA-DRB5	-1.15354	8.08232	-2.73904	0.009349	0.019571	-3.54702
MT1A	-1.65434	2.963563	-2.72321	0.00973	0.020257	-3.58375
SFRP4	1.059524	3.470261	2.718649	0.009842	0.020478	-3.59431
HIST1H2AL	1.214601	0.624249	2.713432	0.009973	0.020722	-3.60636
AGT	1.218644	1.908901	2.706899	0.010138	0.021031	-3.62144
CST6	-1.0144	3.801892	-2.69796	0.010368	0.021464	-3.64202
MT-ATP8	-1.63411	9.73885	-2.68631	0.010675	0.022001	-3.6688
BHMT2	1.044906	1.302364	2.684493	0.010724	0.022083	-3.67296
PCSK1N	1.033478	1.721536	2.679585	0.010857	0.022327	-3.68421
SCGB3A2	-2.11732	6.909848	-2.66638	0.011221	0.022969	-3.7144
MT-ATP6	-1.51941	10.78112	-2.65839	0.011446	0.023367	-3.73261
PKP1	1.065746	1.637743	2.635325	0.012122	0.024538	-3.78501
CPS1	1.271616	0.920948	2.629648	0.012293	0.024817	-3.79785
CLDN3	1.09141	4.465358	2.615658	0.012726	0.02557	-3.82943
CRLF1	1.236854	1.868703	2.615195	0.012741	0.025592	-3.83047
STEAP4	-1.01575	4.472571	-2.61197	0.012843	0.02578	-3.83774
TGFBI	1.004776	5.061404	2.60889	0.012941	0.025944	-3.84466
SFTA2	-1.15324	7.197325	-2.58239	0.013813	0.027461	-3.90404
HIST1H2BL	1.168174	0.689876	2.574625	0.014078	0.027896	-3.92136

Gene	logFC	AveExpr	t	P.Value	adj.P.Val	B
HIST1H4C	1.721168	0.916167	2.558113	0.014659	0.028922	-3.95807
HIST1H2AJ	1.263401	0.638248	2.556338	0.014722	0.029017	-3.962
HIST1H2BI	1.206441	0.610066	2.552837	0.014848	0.029212	-3.96976
HMOX1	-1.13294	4.991788	-2.54545	0.015118	0.029674	-3.9861
TSPAN8	1.222299	3.521109	2.542301	0.015235	0.029871	-3.99306
CLDN1	1.110033	3.705751	2.532566	0.0156	0.030493	-4.01453
CLDN6	1.303536	0.829026	2.530285	0.015686	0.030639	-4.01956
HIST1H2BM	1.074252	0.537126	2.5205	0.016063	0.031261	-4.04106
TRIM29	1.045903	1.560219	2.511251	0.016427	0.031878	-4.06134
AKAP12	-1.08139	3.591408	-2.50995	0.016478	0.031967	-4.06418
AGR3	-1.4652	5.021298	-2.50616	0.01663	0.032233	-4.07248
INHA	1.122835	0.661454	2.505509	0.016656	0.032279	-4.0739
SCGB3A1	-1.96748	6.366877	-2.50314	0.016752	0.032429	-4.07907
CHI3L1	1.184867	4.501617	2.493136	0.017161	0.033116	-4.1009
S100A4	-1.17117	7.759025	-2.47787	0.017803	0.034124	-4.13408
PPBP	-1.20852	2.006791	-2.47739	0.017823	0.034158	-4.13511
INSL4	1.02067	0.515876	2.474325	0.017955	0.03438	-4.14176
BPIFA1	1.92425	2.282872	2.47266	0.018027	0.034492	-4.14536
HIST1H2AB	1.115096	0.563888	2.465933	0.01832	0.034969	-4.15991
HIST1H4B	1.395743	0.731117	2.436964	0.019632	0.037044	-4.22225
HIST2H2AB	1.215213	0.666003	2.434758	0.019735	0.037222	-4.22697
HIST1H3F	1.175856	0.608226	2.434545	0.019745	0.037236	-4.22743
EDN2	1.105865	1.540703	2.431546	0.019887	0.037463	-4.23385
HIST1H3I	1.195813	0.630988	2.418521	0.020511	0.038465	-4.26165
HIST1H4A	1.099168	0.561148	2.410001	0.020929	0.039152	-4.27978
HIST1H2AH	1.217759	0.643445	2.383814	0.022263	0.041357	-4.33522
HIST1H4F	1.000719	0.50036	2.332022	0.02513	0.046006	-4.44353
HIST1H4L	1.115543	0.564569	2.320632	0.025803	0.047095	-4.46711
SLC26A9	-1.0219	2.804647	-2.31535	0.026121	0.047567	-4.47802
MUC5AC	1.47477	0.997043	2.309678	0.026466	0.047991	-4.48971
C20orf85	-1.60663	2.877341	-2.3009	0.027008	0.048824	-4.50776
