# Supplementary material for: DNA damage repair-related methylated genes RRM2 and GAPDH are prognostic biomarkers associated with immunotherapy for lung adenocarcinoma
Source: Genet Mol Biol. 2025 May 9;48(2):e20240138. doi: 10.1590/1678-4685-GMB-2024-0138 (PMC12063672; doi:10.1590/1678-4685-GMB-2024-0138)
Supplement: Table S6 - [file 1415-4757-GMB-48-02-e20240138-s7.pdf]

Genetics and Molecular Biology
https://doi.org/10.1590/1678-4685-GMB-2024-0138

Supplementary Material to "DNA damage repair-related methylated genes RRM2 and GAPDH are prognostic biomarkers associated with immunotherapy for lung adenocarcinoma"

Table S6 - Hypermethylated genes in lung adenocarcinoma (LUAD).

GENE	GENE	GENE	GENE	GENE	GENE	GENE
SCT	C1orf94	DTNBP1	COMP	SMAD7	KLK13	MLNR
TBX4	IRX4	ZFHX3	CTXN2	BEND4	ADRB1	ST8SIA6
C12orf42	PROCR	KCNA6	ZFHX4	GRIA2	SCUBE3	ZNF804A
PCDH17	SEMA4A	NECAB1	HES3	EHMT1	MOGAT3	PUF60
ST18	HPCAL1	CCDC3	LYPD5	NID2	PRHOXNB	SCFD2
LRBA	CCDC101	PRRX1	GSX1	SGIP1	KCNJ10	MAP3K14
TAOK2	RBPMS2	COCH	TSPAN9	ZBTB16	AMOTL1	OPCML
ZIC4	AJAP1	ZNF366	MAP1A	CYTSA	SOBP	SYNGR3
PITX1	TBPL2	LAYN	GRIN3A	KIF26B	IGLON5	INSC
DOCK9	CNGA3	SLC37A1	MLL2	TRIM54	C12orf65	LOC100128239
ARRB1	NHLH1	ZNRF1	CDH4	LOC643719	C17orf72	GALR3
BAHCC1	PODN	LOC646999	KSR2	MYPN	RGS9BP	GALNTL2
CBLN4	CAPN2	ZNF697	MCOLN2	ACOT11	TP53INP1	GPER
ASAP2	ZNFX1	PHACTR2	FAM135B	PPP2R2C	GRIN2C	F2RL3
PCDHGA4	ALX4	TCF15	HOXA4	MOV10L1	PDE2A	PRKCE
PTPRN2	PKLR	IQSEC1	ST8SIA1	MUC4	MGAT5B	IGFBP2
HOXD12	MIR548F5	PTGER4	MSRB3	ADAP2	ANP32A	KANK4
ZIC1	LYRM4	PRDM16	ADAMTS18	SMO	ITPRIPL1	NDST1
RBMS1	NKX6-1	BCAN	MPO	MYBPC1	MAP3K8	MDGA1
N4BP1	C2orf65	SLC24A4	PTPRU	EPHA8	CORIN	POU3F1
CD36	SLITRK1	ACHE	ITGA8	RPH3A	C4orf38	CUGBP2
IFFO1	MRVI1	HOXB7	SIPA1	CNNM1	MYO1G	LOC284798
FAM102A	SLC12A5	DLX4	F7	THY1	PCDHGA5	NRG2
C1orf198	CALCOCO2	DUSP16	GBX2	SYT6	P2RX1	CDH7
KDR	LHFP	F10	CHL1	FAM115A	SLC22A17	VGLL2
LRP2	PRDM6	C10orf46	AVIL	ZNF529	GRID2	TNRC4
SATB2	NEUROG1	WBSCR17	L1TD1	SOD3	KANK3	SEL1L3
DPP6	PAX2	C7orf41	ASCL2	EPHA5	ADAM22	KIAA0182

GENE	GENE	GENE	GENE	GENE	GENE	GENE
ERICH1	TMEFF2	BDNF	MIR129-2	MPPED2	RAI14	NKAIN4
RGS3	ADAM30	DIP2C	HERC3	ARID3A	ODZ3	TCP11
PEAR1	TMEM88	MMP9	PBX1	NTNG1	NAGK	HRNBP3
HOXD4	GAD1	JAKMIP3	MOBKL2C	KCNMA1	OLFM3	C6orf27
GMDS	P2RY12	RIMS3	VENTX	RASSF2	DGCR10	MIR641
GLI2	PNPLA1	FOXF1	ISL2	GSC2	CLVS1	ZNF830
SKI	HOXA9	MAPK14	PRAC	LOC441177	C21orf88	MAPT
YPEL2	SEPN1	C20orf117	CLRN1	ALOX12P2	C19orf41	EFCAB2
EDNRB	C1QL3	ITIH5	GLRA1	MYLK	PROKR2	PPP1R16A
CLEC14A	COLEC11	HOXB6	ATXN2	UTS2R	MYO15B	EYA2
DCHS2	NFATC2	LVRN	AVPR1A	PDZRN4	CACNA1H	GEFT
TP73	B3GNTL1	MDFI	GPR78	PIF1	TMEM220	PPT2
ATP1A1	CNGB1	ZNF608	RSPH6A	ACSF2	KCNAB1	TMOD3
SIM1	MSC	NKX2-3	SLC6A2	DNAJC6	EYA4	ZNF215
FOXK1	GABRG3	CALCA	EPO	KIF1A	POFUT2	RNF150
NOD1	PHOX2A	DPYSL2	CDH8	ZEB2	INSM1	JAK3
TNS1	CPXM2	DLX5	RUSC1	CBX7	RBP4	LRMP
SKAP2	GRK5	GJA4	KCNG3	TOX2	RFX1	DPYSL5
HOXD10	ZIC5	MAPK4	CLDN18	ADCY9	BTN1A1	CXCR6
CRYL1	LHX3	ASCL4	KCNK9	SLC17A8	ZFR2	GALNTL6
MAP1D	ELL	GPC6	FAM49A	NPY5R	SLC17A9	SH3BP4
CASZ1	MNX1	EBF3	PDGFRA	DAB1	RIMS2	ETV6
AGER	HOXC12	ACTL6B	HERC1	LIN28	FAM19A4	OTOP1
MAP3K3	UVRAG	GDF7	PAX8	UCP1	TLR5	SLC9A1
TAL1	RNF39	TTBK1	CACNA2D4	H2AFY	CR1L	SRGAP3
TFAP2D	FMN2	CRAMP1L	CPXM1	LRIT1	RYR3	FRMD3
PAX9	PRIMA1	LRP5	ROBO3	LPPR3	FARP1	ZNF667
SDK1	HDAC7	MEGF10	GAS1	ARL5C	TCTE1	TSHZ3
PITX2	HOXD13	KLHDC7B	MYO10	CLDN5	KCNN2	ZNF300
DLX6AS	MAP3K11	SYN2	ATOH7	TCERG1L	C12orf66	ELMO1
PINX1	CSMD1	KCNIP4	CHD9	FIBIN	NGF	GABRB1
CDC42	GATA3	SLC32A1	FAM123C	CBX2	PDZRN3	FAM150B
FGF14	CRYBA2	NOL4	CCNY	IGF2AS	CYP26C1	RYR1
KCNJ8	LHX8	NPHS2	GRID1	WNT10A	TNXB	RNF219
C2orf55	MAML3	TBX20	AKAP13	NOTCH1	PARP15	PLK5P
ZSCAN18	SLC9A3R2	C1orf70	ECEL1	SHANK2	SNX29	ADAMTS2
HAS1	HLX	BARX1	SP8	LOC149134	RASGEF1C	CRYM
DCUN1D 2	GRM1	CPT1B	KIAA1409	SETBP1	BARHL1	CRTC1
GP1BB	ADCYAP1	LOC494141	OCA2	AGAP11	CHRNA4	RINL
INO80	NKAIN3	FREM3	NR1I2	HTR5A	HEPACAM	ABCG5

GENE	GENE	GENE	GENE	GENE	GENE	GENE
NDUFA4L 2	SLC7A14	POU2F2	ATP2A3	PACSIN1	GPC5	VCAN
GDNF	FAM38B	NR1H3	GIPC2	COX10	ABHD12B	ZBTB20
5-Sep	MYO1B	HAND1	BTBD3	LECT1	SLC10A5	C1orf190
HOXD9	C4orf31	TBX18	GPBAR1	SLC22A16	KLHDC4	RAB8B
MEGF11	TGFB3	SPEG	ASCL1	CDX1	NLRP6	ANKK1
TBCD	RERE	SH3PXD2A	FAM43B	GPATCH8	NOS1	PRICKLE1
DNAH17	TMEM14E	TRPC7	KLHL33	ITGAL	GUCY2D	ATP6V1B2
MEX3A	CPEB3	PLD5	KLHL29	CACNG2	EDARADD	KIAA1217
WNT3	GABRG2	ABCA3	POU3F3	FCHSD2	GMPPA	TNRC6C
ZNF232	ZFP42	C14orf39	SNCA	ULK4	ITM2B	DOCK6
C7orf49	DRD5	BARHL2	ADAM11	RTKN	SLC30A2	FAM71E2
TRAF7	ANKMY1	MESTIT1	TEK	PISD	ZNF385D	C19orf38
MIR124-2	FOXI2	SLC26A9	ITPKA	HAS3	DKK3	FAF1
SMARCA 5	TBX1	CNTNAP5	PRKCB	GRM8	UNC13A	PPPDE1
CRCP	GPR26	NEUROD1	IGF2BP1	CHODL	ART4	NRG3
EFNA5	ZNF274	GDF2	ST6GAL2	HIST1H4F	ICAM5	MUPCDH
GRIK2	PCDHB17	SLC8A3	GABRA1	GFRA1	PLEKHB1	ASGR1
MEIS1	SHANK3	PRKCZ	LASP1	PHF17	PDS5B	ATG9B
ZFP106	PAX7	SHISA3	CDKN2A	HAPLN4	SLC24A2	ARPC5
IFT140	MAMDC2	VPS13D	CPLX2	ADARB2	DES	TMEM8B
LHX9	MRGPRF	NRG1	C11orf75	ISLR2	EDEM3	TRPA1
LRRK1	HOXB3	SNRPN	CLUAP1	SMTNL2	LOC285419	CREB3L1
NXN	UGT3A1	SASH1	CLVS2	MIR124-3	PTHLH	DUSP27
LOC1583 76	KCNA1	HOXC8	SRL	SLC27A1	LOC1001019 38	SPTY2D1
JARID2	FGF5	SHC1	RXRG	KCNC1	FAM5B	PCBP1
EMX2	11-Mar	TRPC3	MAP4K4	DUOX1	PSMA1	PPYR1
TIE1	ANKRD6	NTRK1	SLC4A4	TOM1L2	COL4A1	DOK5
LHX1	HCG9	TTC28	PPP3CA	CARTPT	HEMGN	TM6SF1
CTBP2	INF2	PRRT1	SYNPR	GDF10	SULT4A1	PLAGL2
TLX3	HOXB1	LRRC16A	PDPN	INCA1	C16orf72	MME
MIER2	SNX20	WIT1	WNK4	DOC2A	TNFSF11	ZNF280B
TULP1	FBRSL1	MIR145	VGLL4	HOXC11	LSM12	HGF
BHMT	ITGB5	CHST12	MIR10A	NPY	KCP	ARPC2
VPS53	CMIP	ROBO1	CCDC102A	PDIA3P	IGF2BP3	MAD1L1
PCDH8	ATP8A2	BSX	LOC80054	C6orf132	C19orf35	TMEM179
ST8SIA3	LMX1A	CPNE7	ZAR1	COL14A1	ITGA5	PDE10A
HOXD11	HOXA7	PARD3B	RARRES2	INA	KCNA2	LDB2
ARHGEF4	SPARCL1	C9orf5	NRSN1	TMPRSS2	JAKMIP1	STK24

GENE	GENE	GENE	GENE	GENE	GENE	GENE
SIPA1L1	ADAM5P	RXRA	NKX2-4	RUNDC2A	COL11A2	FZD7
PCDHA2	FGF8	S1PR5	TRIM62	POM121L2	UBE2QL1	C13orf15
SLC41A3	KIAA1161	SHISA9	NPBWR1	SLC5A8	PHACTR3	CLIC4
ABCG1	KLHL26	LOC200726	MEIS2	CASKIN2	SDK2	TSPYL5
MAP7D1	DBP	ODZ4	C1orf114	ATP5G2	ARPC1B	ASTN1
C6orf147	SGMS2	CCDC152	SHISA7	ITGA6	SYT10	C13orf33
TBC1D24	KLHDC8B	SHH	ALPL	LOC441601	HRH3	OTOP2
MCF2L	RBMS2	ASTN2	B4GALT5	PCDHA1	FSCN1	GRIN2B
HOXA2	SLC12A4	UTRN	AMH	OBSL1	KLHL21	MIR196B
FAM53B	KCNB1	HECW2	NCOR2	NPDC1	PLXND1	HIST1H2BH
NBLA00301	LBXCOR1	FGF12	NPAS4	PPP2R2B	ME3	FAT4
C10orf11	LOC440839	LOC100192378	ADAMTS5	SLC30A10	CD247	ZNF804B
RD3	CAMTA1	SOX7	NPTX2	PRKG1	ARHGAP18	HIF3A
EHBP1	CCDC140	PDLIM1	TMEM26	TUBB6	GABRA2	ARHGAP20
C17orf93	CHRM1	SOX2OT	ZMYND15	SNORD1A	PTH2	NRP2
SPTBN1	EPHA10	C10orf105	CNTNAP2	C2orf84	CCNJL	LEF1
ERN2	EMILIN1	DMRT3	RGS14	LMO2	SLC13A5	KIF1B
IGFBPL1	ZNF208	ONECUT3	GPRC5B	SLC25A33	4-Mar	MC4R
ELAVL4	TBC1D22A	PLEKHN1	ZIC2	SYT3	PTPRO	NDRG4
OTX1	SIX3	CDH22	RAX	PTPRF	KCNH4	SFRP4
OTUD7A	LHX5	RGS20	VIPR2	NEURL	C21orf7	CCNA1
HTR1A	DGKI	C1orf187	PRR18	PCDH21	SLC12A8	SOX5
ADAMTS20	DGKA	PEX14	LTBP2	ATOH1	LIMA1	ACACA
EOMES	NSD1	TRAF3	EHBP1L1	C1QL2	SCRT2	ARNTL
FERD3L	GLB1L2	LOC642597	ISL1	TRIM36	C22orf45	LIMS1
SRCIN1	KRT72	UFSP1	CAST	TDRD10	SLC19A3	SNAP91
MESDC1	ZNF98	ABI3	NEFH	PDE4B	PKHD1	LIFR
FES	C1QL1	BCL11B	GLRX	SLITRK5	DEXI	ITGBL1
GDF1	SLC18A3	ZNF536	LOXL2	PCDHB7	CADM3	GPR37
AMOTL2	CUEDC1	KIF13B	AKAP12	KCNB2	GTF2IRD1	MAFA
B4GALNT1	ANKS1A	BRUNOL4	CASKIN1	SLC17A7	CLEC4G	PRKAR1A
EN1	FLJ12825	ARHGAP26	ALDH1A2	ZSCAN23	PLXNA4	B3GALT2
C16orf80	POMC	EMX2OS	FEV	FNDC3B	RNASE7	FGD6
CPEB2	CTSK	CCK	HBM	HOXB13	TM6SF2	CTSA
HOXC4	PPP1R16B	KLC1	EPS8L1	FGF2	MAPK10	PSEN2
ATP11A	CHKA	ZNF560	SP9	SCARA5	ABCB1	HIST1H3E
NXPH1	SDCCAG8	CCDC36	VAC14	RICH2	FBXL18	C5orf49

GENE	GENE	GENE	GENE	GENE	GENE	GENE
DDX25	MAL	ZNF876P	LRRK2	PGLYRP2	BACH2	ITPRIP
KCNS2	MEI1	KCNA5	COL5A2	C20orf46	ZIK1	DKFZP434H168
SPG7	KLHL2	UBE2V1	COX7A1	UGT3A2	FEZ1	KIF13A
PAX3	ST8SIA5	GABRA5	LOC100188947	IGSF9B	GPR88	CSGALNACT1
LOC404266	CRISPLD2	SHANK1	KCNH5	PLEKHO1	FBXL7	RNF126P1
FLJ42709	LOC619207	HSPB2	WDFY2	SCUBE1	ZDHHC3	EXPH5
RGS16	RFX4	WDR20	PRDM12	FAM19A5	CCDC109A	FRMD4B
ZSCAN12L1	C9orf98	TMC2	C16orf45	CARD14	NEUROD2	ZNF879
PIK3CA	SLCO3A1	GRM3	AIFM3	ZNF660	LAMA2	ZNF703
ZMIZ1	SIPA1L2	LRIG1	DOCK3	CALN1	LOC285954	PLA2G7
PRDM8	ANKRD55	TMEM145	TRIM40	RMST	ALDH1A3	CYP11A1
FOXK2	TSSK6	ZNF577	CACHD1	LAMB1	LRRC3	FSCN2
CMTM2	CSMD3	MAP2K1	UBE2O	TTYH1	LPIN2	MMP16
MSX1	ZNF833	ERCC4	UNC5A	TNIK	ZNF471	ALS2CR12
GNA12	TMEM63C	SPAG6	GPRIN1	CAMKK2	TMEM130	RNF220
FEZF2	C7orf50	PITPNM2	TRABD	PPP1R13L	ZPBP	GRIN1
TFAP2A	IGFBP4	KCNG1	C8orf58	SSH2	EPHB2	KCTD2
LEP	GATA4	ADIPOR1	CALY	FTO	SLC26A5	APBB1IP
HAND2	ZPBP2	PCDHGA1	TMEM90B	POU4F3	GNAL	ASAM
YJEFN3	TNIP1	1-Mar	ACTN1	ANKS1B	WNT3A	SYT9
HS3ST3B1	SLC6A17	CADPS	BCL2L11	RLTPR	GPR120	GULP1
DLX1	RYBP	PTPRM	SHF	THSD7A	DNMT3B	FGD4
HOXD3	LOC285548	C7orf16	DLX2	CTTNBP2	C21orf84	CDH12
KLHL31	SHOX2	ABHD2	HOXA1	PDE4D	NHLH2	VAV3
SOX17	TLX1	MYOD1	LOC100128811	ITGA2B	SYDE1	TNK2
PRDM14	GSC	CMTM8	KCNA4	SLC35F1	SOSTDC1	CD96
EMID2	GPR83	IGSF21	IQCE	KLC2	P4HA3	NOTCH4
CTDSPL	RASAL1	EDN3	TTLL10	UHRF1BP1L	MPV17L	OSBPL5
PCDHB15	SOX1	LOC100189589	NODAL	DYNC1I1	PCDHB5	PKIA
MIR10B	DUOXA2	CHST11	VAMP5	FAM65B	NFASC	NKD2
GAK	VILL	KCNQ1	C7orf52	ZNF238	CD300A	DPF3
A2BP1	GALR1	CDH23	KCNK12	AKT1	PTPN5	GJB2
TBR1	CAPZB	CACNG3	ARID3C	RASA3	SRGN	SRD5A2
VSTM2A	GCM2	RALA	CALB1	PAPPA	SEMA6D	GLI3

GENE	GENE	GENE	GENE	GENE	GENE	GENE
FLJ32063	JDP2	LOC100128731	CRABP1	C5orf62	MSI1	MERTK
ONECUT2	FOXP4	DPYSL3	RARG	UBE2Q1	KCNK10	JAM2
C11orf49	9-Sep	FZD2	ACVRL1	SYT2	GHR	LTBP4
SMPD3	APBA2	RASGRP2	PIK3R5	ZNF154	FAM123A	KCNIP1
GRK7	PCDHB3	FIGLA	DOCK2	MOS	TRIM8	SLC38A2
ALX3	FAM110A	CYTL1	SLC6A7	LRRC3B	RNF112	PREP
LHX2	VARS2	NOS3	TBC1D10C	COL11A1	EFCAB1	CNTD2
PLOD2	FOXG1	CYTH1	FER1L4	ITGB4	PSTPIP1	COL12A1
SCARF1	SLC2A14	DAGLA	MARCKS	ZNF521	THADA	ETS2
TBX15	COL2A1	KLHL1	GATAD2B	MLLT1	TMC1	CATSPER4
SND1	HMX2	RALYL	INPP5B	DPYSL4	MAD2L2	EFEMP1
CLDN19	FOXL2	C19orf51	PTPRG	FLRT2	NTRK3	TACC1
PCDHA6	NGEF	RUNX1	PDE1A	LOC399959	MMP2	STX1B
DUSP5P	CTNND2	CADM2	GAB4	GABBR1	PKD2L2	CDK15
TOLLIP	GRASP	GIT2	ADCY1	WNT6	NPTX1	PRSS27
PITX3	CNIH3	ZMYM4	WDR88	BCAS3	GGPS1	DYDC1
KRT81	C10orf47	SYCP1	NRN1L	ARHGAP10	CCDC108	CD1D
PXN	RREB1	HOPX	RGS13	C7orf58	P2RX2	NEDD4L
HOXA5	LOC145845	GPR149	CACNA1A	KCNA3	DNER	PHF21A
VAX1	CHAD	HCN1	SCHIP1	LRRC2	ZNF389	ZNF781
TSPAN14	RESP18	TMEM90A	FZD10	BCL3	CMAH	LOC151174
CACNA1B	C3orf27	NDUFS2	ARSG	C11orf9	GAL	EPS15L1
ELTD1	GPNMB	NOX4	AKNA	TRIM26	SAMD12	ATP2A1
MAGI2	GPR20	TIMP2	C1orf87	SYT14	SOX9	
CRYGD	LRRFIP1	DLL1	C1orf54	PTPRR	8-Mar	
COL23A1	KCNJ3	HK1	VMO1	MDGA2	C17orf104	
EVX2	CCDC146	GRP	CILP2	LOC401463	PLCG1	
EVX1	CUX1	SLC8A2	WNK2	FGF3	CCDC40	
GPR6	PIGV	APCDD1L	SRRM4	P2RX6	CD302	
NR4A3	HOXA10	FOXD3	SORCS3	ACVR1B	OPRD1	
CA3	SLC30A3	TMEM132D	C8orf73	DIXDC1	MIR184	
PLEKHO2	NRXN2	MAGI1	KCNT1	NTM	LAMC3	
PPFIA4	CABC1	TTC36	SMOC2	RBBP6	TCL1A	
GPR21	HYAL2	ATP2B4	ADAMTS16	SV2C	PAK7	
PDX1	HCK	GMIP	GFPT2	GDAP1	SHISA2	
CUL1	RNU5E	NETO1	P4HA1	VPS37B	ZNF323	
C1orf83	PTGDR	FMN1	FAM83F	CRHR2	SYCN	
PITPNM1	PXK	CORO1C	MMP23A	APC2	PSTPIP2	
TRAPPC9	KCNH7	LPCAT1	DUOXA1	GPR139	CLCF1	
XPO4	RIBC2	SOX11	PP14571	GFOD1	SCD5	

GENE	GENE	GENE	GENE	GENE	GENE	GENE
PCDHB16	ATXN7L1	DLC1	CHST2	SH3BP5	JSRP1	
LMX1B	DPYS	GDF3	MKRN1	CNR2	SLC13A3	
LOC134466	GPR25	PRKCG	CNTN6	MINPP1	LOC646405	
PCDHGA2	RARA	ITGA4	FEZF1	PAQR9	SNCB	
NAALADL1	PID1	PTH1R	SALL3	ZNF578	RBM38	
CPN1	ALOX5	LOC148824	LRAT	ILDR2	MADCAM1	
EEF1A2	ZNF648	ULBP1	CTNNA3	TET1	AKAP7	
SST	OVOL1	DBX1	MPPED1	ALK	MIR196A2	
C6orf201	CYP26A1	CHRDL2	HECW1	NUB1	TBCEL	
ALX1	TXNIP	CTDSP2	PIK3R1	AGTR1	DTX1	
PTF1A	SFRP5	ARHGEF3	FLT1	IRF4	ELAVL3	
NKAPL	RASGRP3	PCGF3	KSR1	APBA1	BAT4	
CACNA1I	IRX1	ABCA4	SLC11A2	MATK	PAK1	
OTX2OS1	PKMYT1	ABCC8	RAPGEF5	RGMA	CECR6	
SBNO2	PCDHB18	CRCT1	SFRP1	IL17REL	C14orf159	
MBNL2	S1PR1	LOC100132215	ASPG	ZNF397OS	LSAMP	
RBM20	BOP1	TSSC1	HOXC13	FGF10	KIFC2	
EBPL	POU4F1	KIAA0564	PNMA2	LOC389458	LOC100130274	
CBLN1	OPLAH	TLX2	PIWIL1	ZNF677	IRF8	
PEX5L	LOC644145	LEPR	UNC5D	NFIX	CYP26B1	
NKX2-5	NYNRIN	MFAP4	C20orf26	KIAA0922	PPM1H	
SCN1B	DRD2	NRXN1	C10orf93	FAM19A3	CTNNAL1	
SIX2	FRZB	DLK1	BCAT1	UNC80	IFI44L	
CCDC105	PGLYRP1	CRMP1	SPHKAP	SLC6A1	DCBLD1	
PRLHR	LOC145474	GJA3	SERPING1	IGF1	IFIT1	
CSDAP1	EGFLAM	PAX5	DSTYK	COX6B2	ZNF473	
SLC45A4	GAL3ST3	GPR123	HOXC9	C5orf38	SLC5A7	
EPB41	TRH	MATN4	ECE2	ASB2	MAP4K1	
PCDHB19P	CDK17	PFN3	HOXB8	RASGRF2	PTGIS	
TCF21	GDF6	AOX1	GFI1	HHIP	KIRREL3	
C1QL4	GSTM5	TNK1	KIAA1239	JAKMIP2	SYT5	
CACNG8	AOC2	TRIM58	HRK	GLDC	ACAN	
FOXB1	VSX1	GRID2IP	RELN	PHLDB2	SOX8	
NKX2-6	TRIM59	GREM1	LOC146336	LRRC8C	GPR137B	
KRCC1	MEOX2	GRIK1	NGB	GP5	LOC157627	
PHACTR1	TSPAN4	PAPOLB	MED24	TRIM71	OR2L13	

GENE	GENE	GENE	GENE	GENE	GENE	GENE
CNRIP1	C1QTNF4	SOX10	HSPG2	PPM1L	KY	
TWIST1	TIGD3	MT3	FLJ45983	CRHR1	FOXI3	
PHOX2B	HS3ST2	GFRA2	EGR2	SCN5A	IL21R	
CD248	C18orf1	VWA5B1	CA10	FAM198B	NID1	
ZDHHC14	HOXA3	SFMBT2	HSPA12A	PALM	TNFRSF11B	
KLF7	NMBR	HS3ST1	FOXR1	PLEKHG7	GAB1	
FOXE3	CYYR1	HOXC10	PHF21B	FAM59B	ADAMTSL3	
ANKRD35	C5orf56	TMPRSS12	HIST1H1A	ARID4B	PCDHA12	
CHAT	SALL1	FAM124B	QRFPR	FGF19	SLC6A3	
LOC645323	C13orf26	TNFAIP8L3	HSH2D	PHF2	ATP1B2	
SLC17A6	KCNH2	DSC3	XPO7	UMOD	C1orf61	
SLC6A5	GNG4	ARHGEF10	NPY2R	ASB18	SARM1	
C3orf21	C8orf56	DIO3	CHRD	JAM3	KLF14	
ADRA2A	DACH1	ANKRD50	PREX1	SRRM3	GABRB2	
NEFM	KCNQ4	PAX1	PCMT1	MMEL1	VWCE	
CRY2	C6orf174	NR5A2	AMIGO3	MAST1	RASL10B	
SLC25A2	ZNF132	CSF3R	CASR	SLC18A2	SCN8A	
DOCK5	LOC650226	SEZ6L2	PRX	NEFL	CIT	
HOXA11AS	TCHH	LRRN2	ACTL9	TMEM59L	CCDC65	
T	SORCS1	AKR7L	STAC2	KCNA7	RFX2	
FLJ40504	SLC38A10	MTMR7	RASL11B	PITPNA	PPP1R14A	
DRD4	ZC3H3	LOC145814	CYB5R3	CELSR3	TMEM132E	
ZNF709	ESAM	SLC44A5	CHST8	BST1	HOXD1	
AMDHD1	GLT1D1	MTSS1	CIDEA	STK40	NBEAL2	
KCNC2	RXFP3	ABR	PIP4K2A	SLC6A15	SLC27A6	
FAM114A1	WDR8	EFNA2	ALOX15	OTOP3	PARM1	
MKX	TBKBP1	CCDC8	SUFU	C11orf87	SH2B2	
NHSL1	TMEM196	ITPKB	KCNK17	ANK2	GLIPR1L1	
LEPREL1	PBX2	FAM171A1	SPARC	PTPRD	KIAA1024	
HDAC4	MIR30B	OSBPL6	KCNN4	ARL10	CPLX1	
NR2F1	ACTA1	TBX3	FBXO39	HFM1	TEPP	
CALCRL	SLC10A4	NPR3	SLC8A1	GPR150	ROR1	
DMRTA2	LHFPL4	TRPM8	CHGA	PPP2R5A	LOC389493	
TACC2	ZNF710	ADCY8	YPEL4	PRR15	GBE1	
GJA5	ASAP1	ADRB3	TWIST2	ADAM12	DSE	
CLDN11	HLA-G	HTR1B	C10orf53	DSCAML1	LIPA	
CD8A	R3HDM2	KDM2B	ELANE	TTYH3	EMILIN2	
CNTNAP1	IL13	FLJ43390	SALL4	NR5A1	CLDN22	

GENE	GENE	GENE	GENE	GENE	GENE	GENE
TBX5	WT1	HOXB4	FKBP5	RIMS4	RING1	
CD34	CCDC81	LRCH1	C20orf56	SLCO5A1	C20orf103	
ADAM19	CHRNB1	NPHS1	GLIPR2	CA12	GATA5	
TSPAN10	LPAR5	DMRT1	TRIM61	C16orf11	C13orf36	
C15orf41	UNCX	SPOCK1	THBS4	OXR1	IQSEC3	
DENND3	ARHGAP22	C5orf4	SVOPL	SCGN	XRCC3	
SIX6	FOXD2	HMX3	AHRR	DTNA	GDA	
TFAP2B	DDAH2	VSX2	NXPH2	GPR44	SALL2	
HIPK2	HOXD8	CLDN14	AFF1	LOC283392	COL1A2	
OTP	ZFYVE28	FBXO34	NOX3	GRIK3	ZNF835	
CDO1	MEF2D	KCTD1	RGS10	KIAA1614	PKDCC	
MBP	SFRP2	TF	OLIG2	FER1L5	SYT11	
LBX1	MYH9	FOXF2	RSPO4	NKD1	HKR1	
GUCA1A	CDKN1A	NTN1	C15orf27	LIN7A	BHLHE22	
EN2	NECAB2	VWC2	ABAT	FERMT2	MKLN1	
OSR2	IRX2	MIR196A1	SCN4B	C3orf72	C1orf173	
FAM19A2	LOC401097	LOC389333	CDK2AP1	AGRN	VGF	
KIF21B	HEATR2	HRH2	DKK2	TNFRSF8	SEMA6A	
RPTOR	CWH43	PPHLN1	PRRT4	ANGPTL2	USP24	
OSR1	FLJ90757	EBF1	PLCXD3	B3GAT1	HIST1H3G	
VSTM2B	FLJ22536	SLIT2	ARVCF	CDC14A	FLNB	
RGS12	LOC150197	ANKRD13B	MACC1	PACS2	ZNF662	
ADAMTS 10	C1orf14	ADRA2C	RAPGEF3	NFAM1	XKR7	
PAX6	PLEKHA6	RHOJ	POLR1A	SCARB2	C3orf26	
CDX2	NINJ1	BHLHA9	RPL22	LOC1001909 40	GPSM3	
CNPY1	CDK5R2	FLT4	AQP1	D4S234E	SPTBN4	
ADRA1A	NEK6	CXCL5	SPOP	NAT8L	TLE3	
FBN2	GRIN2A	C10orf82	ADPRH	PROK2	FOXD1	
OTX2	CECR2	GATA6	TACR3	USP12	ELMO2	
PSKH2	NKX3-2	LIN28B	CLEC2L	FOXA2	LOC283731	
CHST13	PDE4DIP	KCNK2	PPAPDC1A	WFIKKN2	IFFO2	
EPAS1	CCDC69	OPRK1	FLJ42875	NCAN	TRAK2	
TBC1D1	RSPO2	LOC730755	MAN1C1	DSCAM	C6orf145	
SNTG1	OPRM1	FAM181B	YWHAG	CBLN2	C9orf171	
ARHGEF2	PCDHB13	C10orf26	ANTXR2	GRIA1	CUX2	
EMX1	TMPRSS6	TRIM15	HFE2	MGC70857	CREBBP	
GAD2	KANK2	COL5A3	BHLHE23	MSX2P1	CCDC106	
PER2	NRN1	CTNNA2	GPR116	CHRNG	ATRNL1	
ESRRG	ZC3H12D	SORBS1	NAALAD2	PRDM1	TPM4	

GENE	GENE	GENE	GENE	GENE	GENE	GENE
PRDM13	GABRA4	NT5DC1	RAB11FIP4	TGFB1I1	ENSA	
PUS3	C9orf129	TUBA8	LYST	CACNB2	HYAL1	
VAX2	FOXB2	CACNA2D3	KIRREL2	PIP5K1C	CLDN6	
GCNT2	GLB1L3	SVIL	GALR2	BTG2	PCDHB14	
POU4F2	ZBTB7B	ZNF541	CHRM2	AGAP1	CRH	
LIMCH1	GRM6	SPON1	KREMEN2	KLF16	SCNN1B	
SMG6	NDRG2	DAB2	ZSCAN1	SNAR-F	TMEM171	
C20orf27	FPR2	SPRED3	CLEC1A	ST8SIA2	C2orf80	
ZNF837	FAM163A	ACSL6	FAM166B	KCNK3	CREB5	
C14orf23	EXOSC7	KIAA0146	ACCN4	ARHGEF15	ANKRD45	
TMEM13 2C	PENK	SGCZ	CRTAC1	DST	EPC2	
FBXL21	STAG3	NTSR1	WNT2	SEZ6	ANKRD33B	
GHSR	SLC4A11	FYN	CLCC1	CFTR	CCDC37	
HOTAIR	TRIM72	DCC	HLA-DPB2	CLINT1	ADAMTS8	
SULT1C4	SLC25A25	SOX14	FLI1	FGF4	RXFP4	
GCK	ANKFY1	TNNC2	C1orf162	MTNR1B	MFAP3L	
MCM6	ZNF454	ITPR1	TSC22D4	KCNQ1DN	INPP5A	
SSBP3	DIDO1	C1orf200	PREX2	JAK1	ARNT	
PCDHB4	PDLIM2	MMD2	NMS	SLIT3	ATCAY	
PRKAR1B	RAB37	GALNT9	ADCY2	IL1R1	HOXA13	
SYCE1	GJD2	MAML2	FAM129C	KRT222	C2orf89	
GATA2	PRR14	ANKRD34C	TRIM9	KRBA1	CPAMD8	
MYF6	LHX4	SSTR1	GABRB3	CCDC48	SAMD14	
GRM7	TCTEX1D1	UTF1	FGR	CYTH2	SNORD123	
TTC22	C2orf40	AGAP3	CSPG5	ABLIM2	C14orf138	
FZD4	MAPK8IP2	HOXB9	IGDCC3	RGS22	EPN2	
PCSK1	FGFR2	WNT16	NES	ANK1	LRP4	
SLC12A7	STMN2	CACNG7	MCAM	SH3YL1	BMP6	
NR2E1	ETS1	RNF122	SLC35F3	SCRT1	BICC1	
LIMD2	CCR7	PSD3	FOXL1	ZNF385A	SETD3	
FOXE1	ABCC1	HIST1H2BK	N4BP2L1	NTF3	MGA	
KIAA0247	IKZF1	FLJ41350	PSMA8	C2orf72	TDRD5	
HPSE2	KCNH3	EXT1	ALDH5A1	EFR3B	NT5C1A	
THRA	FOXC2	RBM24	ATG7	PWWP2B	KLF13	
FSTL4	ZNF598	EMILIN3	INS-IGF2	TRIM67	NEDD9	
ID3	MED13L	FAM54B	NFYC	SEPW1	MLLT11	
ZDHHC24	RAP1GAP	NBEA	SMAD6	TLX1NB	TMTC1	
ZNF438	PTCHD2	ZNF492	NCRNA0009 2	USP10	SYNPO2L	
LBX2	CCND3	BOLA3	PRMT8	CYP2A7	C22orf46	

GENE	GENE	GENE	GENE	GENE	GENE	GENE
FRMD4A	ARID1B	HBQ1	THSD1	KIAA1324	FAM110B	
ZNF718	SEC31B	MIR125B1	MOXD1	NPR2	BAI1	
RUNX3	GRIA4	IPCEF1	LPAR6	GAS7	RPGRIP1	
MGC14436	C19orf57	CD200	MYO3A	CLU	FGL2	
SPATA18	SUV420H1	FAM78B	BCL6B	PTPRZ1	PROCA1	
ONECUT1	SIM2	LINGO3	ST7	C21orf29	WWP2	
DBX2	LOC100129354	HOXA11	NRCAM	FAM26F	RUSC2	
OLIG3	ZBTB4	CDH13	PITPNC1	PCDH20	C7	
TRAF3IP2	MGC45800	CRHBP	HTR7	CD59	DIP2A	
LOC254559	CPEB1	PCDH10	BMP4	C1orf53	NXPH4	
KIAA1804	SLITRK3	ZAP70	STARD13	PHYHIP	MTMR3	
SPOCK3	TPM1	LHFPL3	LRFN5	LOC100287216	MT1G	
GPHN	HSPA2	PMEPA1	TERT	SLC38A11	WIF1	
ERC1	SNTG2	MRPL44	LASS3	STXBP5L	XKR6	
AKAP2	TAC1	KLHL5	RTP1	LPPR5	HMGCS1	
RBPMS	C1QTNF3	MPP5	FAM168A	SYNM	NCAM1	
KCNS1	DUOX2	MUC12	ALDH1L2	BAALC	CCBE1	
ANKRD43	FAM83A	FAM19A1	ACTN2	LTC4S	LYPD3	
ACVR1	PRCD	MCF2L2	GABRD	RUNDC3A	ENPP2	
ANKRD30B	BNC1	COL9A1	HTR6	FAM194B	MICAL3	
MAN1A1	ARHGEF17	KIF19	MIR548H4	HLF	FAM193B	
ACCN1	B3GNT9	MCHR2	RABGGTA	TRNT1	FOXP1	
DLEU7	FAIM2	CRYAB	ENG	RYR2	HS3ST3A1	
PTPRB	NHLRC1	FAM196A	PKNOX2	CACNA1E	COL5A1	
ZNF177	C12orf68	PIK3CG	MYO16	CPNE5	FAM5C	
SLC22A18	EBF2	MTUS1	STIM2	LMO7	RFTN1	
NKX2-8	SLFN12L	SLC6A11	ADCY4	HTR4	CYMP	
FBLL1	PRPF40B	GNA11	PCDHGB4	TANC2	IZUMO1	
NKX6-2	DNMT3A	HSPB6	HOXB2	PROX2	ADAM23	
EMCN	CDH2	PTPRN	NKX2-2	APBB1	SNED1	
STX11	LY6H	SSTR4	ZNF382	CTDSP1	GSG1L	
