# Supplementary material for: DNA damage repair-related methylated genes RRM2 and GAPDH are prognostic biomarkers associated with immunotherapy for lung adenocarcinoma
Source: Genet Mol Biol. 2025 May 9;48(2):e20240138. doi: 10.1590/1678-4685-GMB-2024-0138 (PMC12063672; doi:10.1590/1678-4685-GMB-2024-0138)
Supplement: Table S7 - [file 1415-4757-GMB-48-02-e20240138-s8.pdf]

Genetics and Molecular Biology
https://doi.org/10.1590/1678-4685-GMB-2024-0138

Supplementary Material to "DNA damage repair-related methylated genes RRM2 and GAPDH are prognostic biomarkers associated with immunotherapy for lung adenocarcinoma"

Table S7 - Hypomethylated genes in lung adenocarcinoma (LUAD).

GENE	GENE	GENE	GENE	GENE	GENE	GENE
COASY	NUDT13	KIAA1026	OR8B8	FCN1	TMEM45A	MRPL15
MIR1204	ADAMTS14	SMURF1	ST14	HN1	FAM49A	SCGB1D1
P2RY13	FAM131B	EBF3	ACAP2	GFAP	SETBP1	RARRES1
MUC1	FBXL18	PRKCH	TTLL8	OR51E2	SLC6A3	LPGAT1
ITGA8	OCA2	SLC8A1	RBM12B	FAM155A	TRIM40	ITGB6
SNN	KCNN1	NLRP3	VOPP1	SEPX1	KATNB1	PREX2
TSPAN18	EHMT2	CCDC102B	ZCCHC14	NPPB	MIR592	WISP2
ATIC	LAMC2	DGKA	GLYCAM1	IL22RA2	TIGIT	ZNHIT6
MGAT5B	FOSL1	RAET1K	GSDMB	BCKDHB	PLXDC2	TNNI1
PHC3	SERPING1	KERA	SPRR2C	TRPV3	COL5A1	SPRR2G
44531	GKN1	SFRS8	FCRL6	FUCA1	C17orf102	RGS5
BCAR3	PLEKHA6	ADAP1	C7orf47	ITK	SLC2A13	FAM83H
TNN	SLC35F1	SNHG4	PIGT	TPRG1	MKLN1	DNM3
CAMK1G	ARFRP1	LOC84740	TMEM177	MFI2	PCYT2	A1CF
CDKAL1	RAB40C	SDK1	TIPIN	SLC33A1	LHFP	MGC23284
GFRA2	FPR1	EP400	SLC27A4	FIBCD1	NPR3	CRY2
PKM2	ZNF589	OR51B6	GNASAS	KLK2	KRTAP4-1	AGPAT3
DOCK4	SIK3	FAM180A	MEPE	RUVBL1	NHLRC1	RIMS1
FAM78B	TNFAIP8L2	REG3A	TNFRSF25	ITPK1	SMYD3	ABCA9
RRS1	MIR487A	LOC728276	ARMC2	TSPAN9	HK3	FAM71D
ANO2	AUTS2	SLC38A10	MMP13	LCE1B	HTRA1	CHRNA7
CAPSL	MAL2	FLJ46361	C1orf38	OXSR1	ADAMTS17	RBM22
NTM	LRRN2	PCYT1A	C14orf4	KIF26A	TRIM10	EBF1
APBB2	ANK1	SLC9A9	OR2T6	SYT11	FAM196B	NCOA1
SHROOM3	BPHL	GNA13	GTF2H3	ART1	SOX15	OR8K5
C20orf160	CHDH	MUC13	C1orf150	OR6N2	GPR81	PPFIA4
SH3BP4	TMTC1	SORCS3	FAM172A	ADCY8	MSGN1	C10orf82
REG1B	COL21A1	RGS10	PKD1L1	PUSL1	OR5L2	SH3RF1

GENE	GENE	GENE	GENE	GENE	GENE	GENE
SLC7A14	AMPD3	CGN	KY	FAM119B	CFB	OR5P2
MIR657	ADAM12	PCGF1	MIR1197	ABCD3	HNRNPU	TSPAN8
TMEM156	DCD	SH2D4A	HNF4A	XDH	C3orf77	PLCL2
OAS1	KRTAP6-2	DIP2C	KRTAP19-5	TMEM200A	TOX2	EXOC3
OR10S1	LNX1	TTC39B	CHMP4C	FAM83B	CD300C	WHSC1
KRTAP7-1	KRTAP24-1	SORBS1	C1QTNF7	POSTN	PROM2	C6orf170
ZNF385A	CCDC43	GTF2E2	TCHHL1	KCTD21	KEL	OS9
SAR1A	SCN10A	CHTF18	RPL3	KRTAP21-2	IQUB	ZNF404
OPCML	ALX4	MRPL38	TEKT5	ETS1	ZNF804B	LAMC3
CLTC	ASCC1	CACNA2D1	TRAF1	LMTK3	ERG	MZF1
GSR	MRPS36	OR14I1	DKK3	OR9K2	CNIH3	MDGA2
FOXK1	TSLP	KIAA0922	ESYT2	LHCGR	HLA-DQB2	ADAMTS7
CNTNAP5	SFN	C4orf26	APPL2	SDK2	HIVEP2	CYLC2
CD80	LPP	TMEM169	BMPER	TARBP1	RIOK3	FMO6P
SH3RF3	RAB11FIP1	MMP2	TGFBR3	C1orf210	GALNT6	PGK2
ABCC3	NRXN3	ZFP41	MET	SHANK1	OR51B4	LOC285830
VHL	ENPP6	XPO6	XYLT1	GPC6	GTF3C1	ABCC11
CASP9	KCNQ2	PPTC7	SNX18	SEC16B	LCE4A	GMDS
HDAC4	TRIP4	RNASE7	SF3B3	CD300LG	PDE10A	FLT1
TSTD1	ABTB2	NEK11	TULP1	SIRPG	TMCC2	HIBADH
LUC7L3	DLEU2	FGF18	FCRL3	SNRK	SPTA1	CCNYL1
RELN	GTF2I	MYH11	ZNF80	SLC45A4	PLEKHM3	WIPF3
AEBP1	DNAJC5B	MEST	KHDRBS2	PYGB	PPAPDC1A	RP1
ITGA2	ADCY3	NFE2L3	MOGAT2	WISP1	CXCL2	PPAP2C
SLC34A3	WSCD2	TULP4	TRIM6-TRIM34	OSMR	KCMF1	CTNND2
PBX1	ZNF469	ZNF536	FCN2	SFXN5	TPPP2	AMFR
SNHG3-RCC1	BTBD17	OR1J4	MIR411	LOC100130872-SPON2	GPR158	UBE2O
LDB2	RGS6	OR8G1	KCNK18	CPEB3	PTCD2	TDO2
NR2F2	TNNT2	C14orf37	SYN3	LOC285768	BRSK2	FAM129A
TNS4	FBXO5	WDR86	SDCCAG8	FAM190B	PZP	ODZ4
PLEKHG6	DAK	MYL5	C1orf106	C2orf78	PLEKHG4B	MLPH
SHISA9	SPRED2	OR6B2	ESPN	TXNDC3	C7orf53	DERL1
TNFRSF10A	C20orf196	CCL13	PMVK	TBX6	OR51M1	KRTAP10-8
RIMBP2	CCND1	BUB1B	APLNR	SGCD	TCHH	B3GALT5
PARP4	SLC39A11	LRFN2	VARS	OGDHL	EPHB4	TNRC6A
GRAMD4	DEFB121	MIR921	ABCC12	GPLD1	SORCS1	KRT15
DPP6	RER1	COL9A3	PTGER3	PDE4D	TERT	DNER
GPR56	DNTT	XKR4	C7orf16	NALCN	SLC16A3	OR52A1

GENE	GENE	GENE	GENE	GENE	GENE	GENE
C1orf95	TMEM184A	HCRTR1	XIRP2	KCNA4	DCLK2	CASZ1
CALN1	KCND3	AFG3L1	MS4A4A	RIMS3	RGS17	C5orf39
C10orf110	SFT2D3	OR4D11	AFMID	PLEKHA7	ST6GAL2	PTCHD2
ELF1	TMOD3	HTR1E	LY6D	CBX2	OR6K3	TEX264
TRIM29	CPNE1	MASP1	MIR377	SLC38A8	IL1B	CNR2
S100A10	KCNQ1	SLC12A1	NOD2	OR51B5	CCL8	ISX
C7orf34	GP2	C10orf90	AGTPBP1	CRP	RGS7	KNDC1
FHIT	CLN6	HHLA2	BAI1	RNASE11	YOD1	C7
PMEPA1	KIAA1143	JPH3	SNORD114-31	SNORA63	PXMP3	COPS8
TTC15	IRF2	CLDN1	TTLL1	OR56A1	TLN1	NDUFA4
SHCBP1	DAPP1	MED1	RFFL	GAP43	PPM1L	ZNRD1
TCN1	LOXHD1	DDR1	OVCH1	GATA4	APOBEC3A	BTBD9
PRRX2	TMCC1	LSP1	SVIL	OR56A3	EPS8	CACNB2
MIR21	OR10W1	THSD7B	LIG3	ITLN2	ELAVL4	AQP11
CLCC1	HRNBP3	TCF7L2	WDR25	IQCK	OR8D1	USP24
KRT80	FILIP1	PILRB	SCN8A	LOC388965	VTI1A	CCR2
EZR	PRLR	SERTAD2	CPLX4	IFLTD1	LRP12	VIPR2
TPCN2	TRPM2	KIAA0513	DUSP13	PDGFRA	ADD2	ATP2A2
KIAA1609	SIRT6	SQSTM1	LELP1	MIR190B	C6orf176	SLC26A9
MOXD2	EFHD2	SPRR2D	GMCL1L	LOC100128542	MYO1D	WWOX
RAPGEF1	TBXAS1	KLHL29	LPPR4	CD1C	SAP30BP	MUC6
NKD2	CNTNAP2	KIR3DX1	EFCAB2	PRSS27	ALG1L	LRP5
YWHAZ	AOX2P	FGGY	SORBS2	PRIMA1	ZNF532	MYH8
SNORD93	OPN3	S100A8	NUAK1	RGS12	BAG3	SNORD113-5
C16orf78	GMPR	C16orf73	MFSD4	OR2AT4	CYP11B1	PAK6
COL18A1	PTPRH	TCL1B	NLRP4	HSF2BP	PDE11A	C15orf38
TBC1D16	HDAC5	TWF2	RBP3	ZNF783	ARHGAP26	FRS2
TNR	FAIM3	CCL14-CCL15	CDH22	BLCAP	IGFL3	LTBP3
DPYD	TMEM19	OR8A1	FCRL2	C7orf10	FAM18A	DNAH2
UNC5D	NCRNA00114	MYH1	STK31	SYT4	OR52B2	MFHAS1
ZDHHC14	PIGW	COL29A1	HABP2	PGCP	HS6ST3	SLC1A3
POMT2	LRRC8D	GRID1	NLRP7	EMP3	PPP2R2B	AGPAT4
TRAF7	IL16	C4BPB	TEX14	LOC202781	SLC4A1	LOC100216001
ZNF217	TRAF2	HIVEP3	PFN1	BRPF1	IL18	ESRRG
CST9	FNDC5	OPTC	ENGASE	ST6GALNAC3	TBC1D22B	C8orf34

GENE	GENE	GENE	GENE	GENE	GENE	GENE
FGF6	MIR19A	CHD2	LOC13446 6	PIP5K1A	ESPNL	SNORD47
SEMA5B	PALLD	UNC5A	OR2Y1	TLR4	GOLSYN	RBCK1
NRP2	PPARGC1B	OR4K5	ACTR3	YIPF7	LYPLA2	PDE4B
KRT8	IL7R	SBSN	SLC35F3	EHD4	HDAC11	ZNF250
CASP8	GPR115	C1orf226	MIR122	DOCK2	DLG5	LRRFIP1
CBFA2T3	VAV2	TMEM80	RPL30	WISP3	OR5AS1	MEGF11
MPPED2	NOTCH4	OLIG1	DSP	TG	GHR	ATP5C1
NAV2	OR1F2P	ZNF831	GRXCR1	ACTN2	ACSM1	STEAP3
KRTAP11-1	TSHZ2	UBE2V2	IGSF21	PARD6B	NOS1AP	HAS1
TPRXL	WEE1	LOC643406	OR5P3	GALP	CHD1L	FAT3
CMKLR1	D4S234E	SH3GL3	CTBP2	OR51B2	TAS2R40	LMLN
C11orf60	COL11A2	BRE	ZFPM2	ZBTB20	CPNE7	CD58
SLC2A1	ITGB2	ADAMTS16	DBH	HLA-DOA	RAI1	TNIP3
CCL22	PPAN-P2RY11	ATXN1	RPL13AP5	KIAA0146	GAS5	C6orf27
MYEOV	SCTR	SPTLC3	OR2G6	GPR26	SERPINB11	SIRPD
ARL16	BATF	GSTP1	SYT12	PTHLH	DRD3	C1orf125
SCN4A	KCNN4	OR2M3	C1QB	OBSL1	STRADA	MIR1283-2
TRIM15	KIR2DL4	RALA	PARK7	GNG7	FKBP2	CENPJ
CUX1	DNAH9	RPS8	AOAH	OR10C1	SNORD70	ADCY10
FHL2	FAM13A	VANGL1	STK39	C21orf34	ZNF517	NPAS2
GLIS1	MAML3	KRTAP15-1	MICALL2	C20orf85	IRF8	ZSCAN12
TAGLN3	KRTAP13-2	NPY1R	TRYX3	MYH7	FAM111A	OR2T4
CSMD1	LOC100128 191	COL6A6	FMN2	TRIM41	NPVF	CSNK1G3
SPSB1	CASQ1	ANXA4	MIR299	PLCH2	MGAM	CLEC4E
MIRLET7C	SRCRB4D	CLSTN1	ENDOD1	THBS4	OSBPL3	LSAMP
FAT1	DPF3	LMTK2	ENTPD6	PAG1	SP1	NLRP13
ANKRD46	ZNRF2	DEFB118	TBX19	PRDX1	DLGAP2	MEG8
TMEM132C	MRGPRD	UNC93A	TNIP2	MEF2D	CLTB	TYR
PIP4K2A	LOC339568	RAMP1	CD1E	LCP2	UBE2QL1	TRIM42
LTBP1	UNC5B	RBM15B	FAR2	NFKBIB	TRAF3IP3	LCE1D
RICS	FARP1	RNU5E	MAN1C1	ANKRD33B	TMEM33	GRM3
ENO1	DSCAM	SERINC5	CPLX2	MMP1	TBC1D22A	FNDC1
CDH13	OR10A3	NOLC1	OSGIN2	CAPG	SLAIN2	AP1S1
HDAC9	LFNG	IL1F9	NMNAT3	CCL18	OR10K2	C16orf11
LRIG3	JUP	RGS20	SRPK2	DGKI	LILRA2	ABL2
USP2	HSPA14	SPOCK1	GJB3	LRRC55	CACNA2D3	FAAH
ABLIM1	CEP55	LOC730811	PHACTR1	KALRN	OR4D2	NEIL3
DUT	RAG1AP1	UNC93B1	SEPHS2	LMO7	SH3PXD2B	MIR549

GENE	GENE	GENE	GENE	GENE	GENE	GENE
FAM83A	FMNL2	ABI1	MIR487B	SPATS2L	PLD1	C19orf41
TLL2	KRTAP19-8	C16orf72	WDR35	ASTN1	ELMOD1	NECAB1
DLGAP1	ASPSCR1	TM4SF4	NLRP11	ST6GALNAC5	OR5T1	CEBPG
SMCHD1	MPPED1	HCK	DHX9	VGLL3	MAMDC2	COQ3
HCG27	SOBP	NKPD1	SDC4P	XBP1	PRKD1	NHEJ1
ARPM1	FAM105B	CD207	LHFPL3	TMEM44	LCE1F	ARHGAP10
GRIN2A	MS4A15	C21orf29	GRIK4	SHISA6	MRPS25	CRIM1
RPS15	ZNF662	KPRP	MIR206	C14orf49	44256	LPIN1
CHRNA9	FSCN2	IL12RB2	TLK1	MIR1185-2	COL28A1	ZFP2
TMC8	SLCO3A1	RTN1	WDFY3	IGFBP5	GIMAP4	DSC1
MFRP	GRM8	MYOCD	KPNA7	D2HGDH	KIAA1549	MERTK
MAGI2	CDH4	APRT	NMI	RABGAP1L	CLP1	LAMA4
FAM178B	SEMG1	TMEM51	CRYBB1	RPL23A	PON3	CHRNA1
IRS1	ADAP2	RRM2	CLIP2	MS4A2	ENAH	FGF23
SOLH	AGAP1	FAM19A4	ZNF511	NAPEPLD	AP3B2	OR6B3
PTK2B	SLAMF7	GRIN2B	CHST6	ELFN1	MBOAT2	LCE2D
C17orf48	PVT1	VPS37C	FNDC7	SPATA16	ZSCAN1	HIF1A
AGPAT9	LOC644649	SLC22A6	P2RY6	KRT2	VANGL2	FBXL16
ANTXR1	OR5A1	FEZ1	RCAN2	IL17RD	MMD	TUBB1
LY96	CSDE1	PREX1	CADM3	THAP11	GPR113	MYH15
AIP	OR9Q1	SLC17A1	MIR298	SELE	IL22	CAPRIN2
G6PC	GPR39	LAMA3	ACOT7	TECTA	FLG2	LARP1B
C14orf182	LOC340094	DEFB134	NPAS4	EIF4EBP1	LOC646627	LRRC32
CCL26	CD68	ACVR1B	CCDC150	ZFAND2A	NNMT	PERP
KRT5	YAP1	PRMT3	BAIAP2L2	RASAL1	LOC349114	OR2T10
HCCA2	OR6T1	KCNIP4	OR10G9	OAT	FAM154A	ZBTB46
GHITM	CSMD2	IGF1R	LOC28520 5	UGT3A1	C17orf58	ATP2B4
CACNA1C	GPR4	CPZ	ARHGDIA	CTSB	ELMO1	BAT3
SAMD14	FRMD6	LOC285780	EPCAM	NPEPPS	LOC732275	NPFFR1
SMAD2	STON1-GTF2A1L	PANK4	FAM53A	REPS1	ACTL6B	CHORDC1
KRTAP21-1	DIRC3	NEUROD4	RABGGTB	SLC19A2	KRT1	KIAA0802
FGD6	GPM6A	FAM135B	LOC64200 6	SEC63	KRT6B	FAM24A
KIAA1217	PTPRN2	TRAF5	RP1L1	TM2D3	SLC20A1	LRRC24
MST1R	SUGT1L1	ARL13B	SHANK2	HLA-DOB	FBRSL1	SLC7A13
SLC41A3	DLG4	CRMP1	DCAF4L2	ACOX3	ST3GAL3	PPP2R2D
CHRM2	FAM20C	ATF7	ATP1B1	KCNN2	S100A12	C10orf107
PEX6	ITPR2	REG1A	HECW2	SEMA3A	MTHFD1	COIL
SCHIP1	TUFT1	HCFC1R1	CPAMD8	OR6X1	OR4D6	OR4A16

GENE	GENE	GENE	GENE	GENE	GENE	GENE
MYCBPAP	RASAL2	PLXDC1	SNORD114-23	ATP8A2	LAMP3	CREM
VAT1L	ESR1	ACBD5	FOXP1	UPP1	GPR155	SFRS1
CCDC6	ARNTL2	PYCR1	SLC6A12	PLEKHA5	NPM1	ASCL4
TRPM8	ADAMTSL1	MSR1	STAU2	PIGB	RPL13	GCHFR
MGAT5	LRRC3B	RFX4	SNORD115-10	SCN1A	UFSP1	ARID5B
CUEDC1	TNRC6B	ZFHX4	ISG20L2	HBBP1	CLCN1	PIK3R1
MYT1L	OR51L1	LCP1	CCDC109B	MMP16	TINAGL1	ANKS1B
MYLK	AFF3	C6orf118	OR51D1	KCNA1	OR10G4	C11orf41
ATG9B	C20orf103	SNRPA	PACRG	CLIC6	WBSCR17	GRB2
UAP1L1	CENPM	OR10X1	TNFRSF9	CMTM8	SH3BP5	HS3ST4
HNRNPF	RNF145	C5orf37	AMTN	C2orf28	C8orf84	BDNFOS
SPI1	MREG	PITPNM2	SH2D2A	ABHD6	MTPN	EXT1
FAM92A3	NAGPA	SEMA6D	SPEF2	UGT3A2	CDC42SE2	PPP1R3D
WDR90	FGF1	TBCD	OR5B2	FLJ43860	IL1R2	NUP62
NUP43	EPHA10	TM9SF4	ENTPD7	TCP11L1	PLCL1	RSRC2
OR2B11	SLC8A3	TMEM225	FRMPD2	LCE1C	ROR2	NOS1
CAMK1D	HSPA2	HPVC1	CHCHD6	USH2A	HHLA1	TP63
RPTOR	GLT25D2	LOC399959	CAPN2	MIR1179	TTLL2	BSN
CTNNA2	GON4L	COPS7A	GLYAT	RAB25	CRYL1	DEPDC7
TMEM49	ACCN1	KIFC3	FAM189A1	CELSR1	LOC100130987	SNORD113-4
RIT1	DUS1L	USP29	TRERF1	KIAA1274	CACNG3	ATAD5
HEPACAM	BBC3	GRIN1	GRIK5	MIR380	GEM	INADL
MAP2K6	BAIAP2	TRIM58	TRAF3	GRIA4	TMEM39A	CCDC84
HMGA2	GPR139	DUSP14	IL21	AZGP1	GNS	GRHL2
NDRG4	RASA3	GIMAP1	PIK3CB	TTC7A	C2orf62	GPR183
C17orf73	SERPINA10	NID2	DRD2	C6orf106	MIR1207	ARHGAP5
GK2	FAM38B	COL8A1	EPHA4	SLC7A11	PARK2	IKZF1
TXNDC11	HPYR1	KIAA1199	SNORD114-6	SNX10	LUZP2	CTTNBP2
SYT7	C1orf107	MYOM2	OSBP2	CHST4	SV2B	SST
NR5A2	SPRR3	ARPP-21	MMEL1	HECW1	KRT71	INPP5B
PARD3	IVL	OR6P1	CYP11B2	44266	TRIT1	IL4R
BTNL2	AGA	ARNT2	PPM1G	ANKRD24	ZC3HAV1L	LEPR
C20orf114	RPS6KA2	OC90	CD164L2	PTPN22	DZIP1L	CNBD1
EMB	PRKG1	CCDC57	CD1D	KIF16B	TXN	HMGB4
NRP1	CXCR1	PROX1	COL13A1	C2orf90	PBRM1	DRGX
KSR1	RAB17	PDE1A	WDR36	ABCA2	HBP1	KC6

GENE	GENE	GENE	GENE	GENE	GENE	GENE
CPA4	COBL	C11orf93	SLC12A9	TMEM163	E2F6	MXI1
IQCE	ADAMTS12	COX6C	TCL1A	OR10V1	EYA4	KIF26B
ADAMTS8	NDRG1	NRAP	TTLL6	ROBO1	OR6K2	COL16A1
APCS	OVOL1	CDH8	GAS7	DOCK1	ALDH3B2	RBL1
TAF1B	SUB1	GNA15	MACF1	HTR3A	RASGRP3	MIR155HG
SCOC	KRTAP13-3	NTF3	NOTO	DEFB126	C12orf74	UBE2Z
PAX4	AHR	HLA-DPB2	EPB41L2	IQSEC3	C1orf204	SNIP1
CYYR1	CACNA2D4	FAM131C	GALNTL2	GDPD4	KCNN3	SIRPB1
ANKRD10	LOC100134 259	DUXA	JAZF1	NXN	NOL10	OGDH
C10orf93	CUX2	AGBL1	RBM20	RIN3	PRICKLE2	SRD5A1
CC2D2A	TBPL2	TRIM31	RANBP3L	ROD1	CP	GPR125
PIP	TNNT3	OR2G2	SLC34A1	SULT2B1	RNLS	PLCB2
SYTL1	AGXT2	NTRK3	KRTAP19- 1	KIAA0406	RARB	LRCH1
CEACAM19	MYH4	SH2D4B	SLC16A1	C10orf128	C3orf26	CLDN8
BNC2	CX3CR1	PAK7	LOC15056 8	CMA1	C1orf92	CYHR1
EIF4E	IL21R	MAP3K9	HHIPL2	SNORD113-6	ETV6	USP36
RIN1	TPK1	MIR543	MSRB3	CPA3	CNKSR3	DNAJB6
HELB	PLCD1	MIR495	LOC90246	ABCC2	UBASH3B	CTNS
TBL1XR1	GEMIN5	LRIT1	BGLAP	ETV3L	ATP6V0D1	ZSCAN18
TMEM63A	C7orf58	OR2G3	NCOR2	TRPV2	ITPR1	OR6K6
PFKFB2	ITIH2	GHRH	CYFIP1	PATE2	CNPY1	ATP10A
MIR520C	PRKAG2	RAPGEF4	VTCN1	SPRR1A	UBFD1	TNFRSF1A
SERPINB5	PET112L	OLFML2B	TMEM40	PDILT	TRAPPC9	PRKAR1B
GPR111	DEFB135	CCDC40	LRP11	TRHR	C21orf2	LHX6
MTRR	FAM19A5	EMR4P	LOC14869 6	COL22A1	FAM13C	AP3S2
RCSD1	ENTPD8	MMP26	RNASE13	EMX1	NSMCE2	SLC6A19
HMCN1	GYPC	OR51F2	RALYL	C11orf94	MAEA	RPL22L1
C1orf101	FBXL22	MIR154	RNASE3	STK38L	YTHDC1	HMGB1
KCNT1	FOXN2	OR8D4	PEG3	CNST	BMP1	GRAP2
RGNEF	PLEKHG5	KCNK12	MITF	FRMD1	SLC5A10	RYR1
HPCAL4	CES8	PAPPA2	LOC28386 7	MOBKL2C	NCOA2	RSPH3
SCN11A	KRT26	TBC1D14	PLEC1	SPATA19	FAM107B	C6
T-SP1	LCE3E	KCNA5	ARID1A	FAM38A	MYH6	AKR1D1
PRKCB	OVCH2	OR51G2	PRR21	APOLD1	DKFZp566F0 947	C8orf44
PLXNA4	IMMP2L	VDAC3	RPS18	CD109	NF1	PLEKHH2
NRG3	SPTBN1	PLA2G4C	TMCO7	MIR670	MIR412	IGFBP7

GENE	GENE	GENE	GENE	GENE	GENE	GENE
SLA	DGUOK	UBXN11	ZBTB7C	INPP4B	RASGRP2	STL
TMIE	ALLC	MIR515-2	SLC22A18AS	AGR2	BEND7	EFCAB9
ADAM28	TTF2	TAF1D	CACNA1E	RNF149	CDYL	TMEM131
SLC12A7	IL23A	C4orf34	CALD1	FSCN1	SPEG	DLGAP3
TGFB1I1	CACNA1H	TSPAN5	PLD6	ADPRH	OR51S1	INTS3
FAM55B	HRH1	NCRNA00162	ANO1	LCE1A	FLJ33360	SNORD16
MOCS1	CSNK1G1	GABBR1	OLFM4	OR10T2	AMPH	ATF3
ASAM	PNOC	GPX6	FCRLB	PROCA1	PANX1	RPL6
GAS2L1	NRG2	MRGPRX4	GCNT2	GEFT	FLJ39653	BRCA1
HNRNPA1L2	RUNDC3A	ACP6	C16orf45	GPSM2	HLX	COL6A1
TTC39A	MYO3A	SNORD114-29	OR10A6	ANO4	HS3ST3A1	BCLAF1
UPF2	BTBD16	LOC440335	JARID2	TBC1D21	UNC84A	PGAM1
TMIGD1	PEAR1	ELK3	LGALS2	ZCCHC4	HNMT	SFMBT2
NRXN1	FOXI1	AADACL3	SAMD12	KIR3DL2	DBNDD1	ADAM10
ZFHX3	SORL1	DKFZp434L192	RPH3A	LMF1	LEUTX	VWC2L
C3orf59	FAM113B	C1orf105	WHAMM	GJB2	SNCA	EDEM1
ACTA2	PSORS1C1	LPO	PITX3	C4orf19	ZBTB11	CAPN8
ROBO4	SORBS3	ZNF648	OR11L1	GPC5	BANF2	TATDN1
GALNT9	ELF3	BAALC	ABR	LOC100130017	GABRA6	ITLN1
SFRS13A	HMGA1	CDH23	MOG	DENND5A	ATP1A2	KMO
CLIC3	GDF15	KIRREL3	KRTAP2-4	NAAA	LOC339524	FUBP1
PTP4A2	ZC3H12A	TNP1	KIR3DP1	DCC	PKP4	ADCY1
GAL3ST4	OR8B2	WDR81	LGR4	ZNF341	CST7	RFESD
SLC39A12	EMR3	SV2C	NCKAP5	MIR654	PDZRN3	METTL9
ZC3H12D	TRIO	KCNMB1	C18orf1	OR8J3	LOC100128977	GARNL3
IL1A	LY6G6E	NAA25	DUSP27	ZSWIM1	TSKU	C11orf40
PKP3	SMYD5	SYNJ2	HLA-L	RASGRF2	FXYD3	FAM82A1
GALNT7	MIR329-2	RPLP0P2	PIK3CD	MCTP1	SPINK5L3	MDC1
CACNA1B	CHRNA6	ZBTB25	THSD4	MNDA	TFPI	TRIP13
PKNOX2	LRRC4C	SNHG11	OR9A4	C19orf6	EFTUD1	FRAS1
C1QTNF1	PPP2CB	KRT6A	FAM160B1	OR51A7	MIR134	SERPINB13
LOC285194	CD44	HSD17B8	SELENBP1	CST5	ZC3HAV1	CCL16
ARHGEF3	IFFO2	KCNJ2	BPIL1	ATF7IP	PLA2G10	RPL24
TCERG1L	SMAD3	C5orf49	DRAM1	PTPRE	NAALADL2	LOC100124692

GENE	GENE	GENE	GENE	GENE	GENE	GENE
PANX3	CPNE5	RYR2	KCNA6	NPAS3	C20orf118	MIR376B
FBXL7	AFAP1L2	B3GNT3	C3orf67	KCNQ3	TRPV6	PI4KA
BBOX1	POLR3A	OR1A2	HOOK2	ACPP	NKAIN2	OR2AK2
OR8D2	C20orf199	SLC39A2	CD164	SLC22A8	UMOD	LAIR1
UBTD1	TAS2R38	SPATA8	TMPRSS4	ZYG11A	NCRNA00157	THNSL2
PHLDA2	DLC1	KCNK9	C20orf54	AICDA	OPRM1	PTDSS2
PYCARD	NPS	LOC645323	UBE4B	PHACTR3	R3HCC1	TTC33
B3GNTL1	RNF207	OR10J3	GLYATL2	TRNT1	PEX14	LOH12CR1
SECTM1	CDK14	OR9Q2	RAC1	ARHGAP9	HBE1	CAMKK2
TRIM14	DNMT3B	DST	SEMG2	ZCCHC8	PABPC4L	ICK
KCNJ6	VAMP5	CPO	EFCAB1	TINAG	EPHB6	RUNX1T1
EEF1A1	TCF25	SLC35F2	SLC26A8	HMOX2	OR2D2	HRNR
SPSB4	ZNF827	ITGB7	LOH12CR2	U58	RUFY1	AKT3
RASGEF1B	KCNJ1	ALOX5AP	AKNAD1	GRB10	NTNG1	SLC30A8
OBSCN	OR6N1	ORAI2	NPW	CTNNBIP1	PSMD14	OR2T11
TNFAIP8	NUDT1	LMX1A	FAM167A	CDK5RAP1	EGFLAM	WWC1
DEGS1	FCRLA	LEKR1	KIAA1409	SLC35F4	NELL1	ZNF7
C1orf94	LRIT2	CNTNAP4	TTLL11	XKR6	C6orf150	ILDR2
SLC25A38	C10orf81	HTATIP2	AJAP1	RNF121	OR5AU1	LOC257358
NLRP10	ADCYAP1R1	SYT8	IL5RA	C1orf229	HUS1	C1orf9
GPR87	ASAP2	LOC253724	DPYSL3	RASGRF1	SH2B2	EVC2
AATK	ICMT	ANK2	ADCY9	PATE3	DCBLD1	LRRIQ4
MAP1LC3B2	GRIA1	BUD31	SELPLG	C8orf22	KRT73	VWC2
GRK5	IFFO1	HS3ST2	LGALS8	GPRC5B	SP6	SIK1
SNAPC5	C22orf25	OR1C1	TBC1D9	HN1L	MICALCL	HTR1D
MCC	MYT1	OAZ1	SLC45A3	GUCY1A2	KDELR3	RAP2B
PDZD2	CLUAP1	C2orf60	ELOVL5	MAD1L1	TPSG1	
TMBIM4	NMS	GPX5	ARMC7	KRTDAP	RWDD2B	
SLC12A5	MIR488	KLHDC8A	IGF2BP3	LOC285692	C9orf173	
CXCR5	LOC154822	PRKCE	METTL11B	OR2L8	C13orf28	
LY9	SGK3	GLI2	SH3RF2	DCK	TSPYL6	
INTS6	PSMG3	TDRG1	OR4F6	UNC13C	SNORD87	
GIPC3	LOC100132111	TSPAN10	RHOJ	MGC27382	UBAC2	
NQO1	VAV3	TTYH3	LOC285954	DYNC1I1	PPARGC1A	
EIF2AK1	AMN1	CPA5	MYO3B	RECQL5	AACS	
PSMB2	IRF7	SRD5A3	OR4C16	ENOSF1	OR5D16	
MIR496	PC	SCGB1D2	AP3S1	MIR548I4	WIPF1	

GENE	GENE	GENE	GENE	GENE	GENE	GENE
ECHDC2	C5orf32	BTF3L1	POM121L12	OR5L1	SNORA64	
NECAB2	OR6Q1	ACOXL	CLRN3	KIAA0748	KCNA7	
HMGCL	CLEC16A	COL4A1	TMOD1	FTO	CLDN12	
EBI3	DAAM1	STK24	MAFK	LAPTM5	TRPV5	
KRT7	OR2C3	MDS2	ADAMTS10	RAB37	LRTM2	
KRTAP8-1	ADARB2	C8orf85	MIR758	PTPN21	C11orf85	
HSD17B2	DOCK10	IL31RA	SLC36A2	CACNA1G	RNF11	
TNXB	CAMTA1	ZNHIT1	SNX29	ZMYND8	PFDN2	
VTRNA1-2	WDR64	GALNT2	CTHRC1	SLC35B3	KRTAP20-3	
MORN1	MIR770	TPO	ADAM9	TCEA1	HKDC1	
MIR548G	FBXL13	ZBTB32	LCE6A	LOC284009	DCP2	
GREB1	CNTN2	SNORD113-2	ANKH	SPTB	FRMD4B	
BSND	FBN2	TTPAL	TMEM90A	LOC145820	STEAP2	
TCL6	HACE1	TTC38	RAI14	PHRF1	PTGDR	
CALHM3	DAB1	CCDC19	DPP10	OR52L1	CUBN	
ARHGAP15	PLAT	OR8B4	ACTN1	PFDN5	EVI2A	
CHD5	WBP2NL	ADA	SNORD18A	RNF214	CADPS	
MYH16	PPM1H	MS4A3	PADI1	SLC25A41	IL17A	
FLOT1	PCDH21	ERICH1	BSG	SULF1	OR8K3	
GRHL3	ACSBG1	NFE2L2	CES7	LAMA5	NID1	
TERC	MIR526A2	OR51F1	NCOA7	OSBPL6	CXCL12	
C3orf32	PTPN14	CDH20	MGC42105	IGDCC4	RPS6KC1	
ADCY2	TRPM5	DACH1	ITPKB	HEATR7B2	OR8H1	
EXOC4	CLEC2B	FLT4	TAS1R3	KCTD19	PLXNA1	
IRF2BP1	C6orf48	UNKL	MFSD7	COL9A1	FLG	
CLEC7A	MAPK15	GFRA1	VSTM2A	ATF6B	FCRL4	
SLIT3	FASTK	DEFB119	CLDN17	CBLB	FAM110B	
CABLES1	KCNH1	A2BP1	FYB	FRMD4A	ATP13A5	
AMHR2	IER5	OR52A4	RBMXL2	HAVCR1	B3GALT4	
CARS	CSF1R	C1orf111	SNAP47	GRAMD3	EFNA5	
EIF2C2	FN1	CYP3A43	SYNPO2	HSD17B12	CIITA	
OR4D1	TIAM2	OR6M1	CSNK1A1L	SMOC2	STX1A	
CMIP	SLC5A4	BAIAP2L1	STXBP1	PPP2R3A	MCM3	
SLC2A9	MCF2L	SNHG12	ACYP2	NUP153	RARRES3	
RUNX1	OR5B12	DUSP4	FGD5	GDPD5	TMEM108	
MYO1E	SYN2	ASB13	ACCSL	LOC388796	PRR5-ARHGAP8	

GENE	GENE	GENE	GENE	GENE	GENE	GENE
AHSP	OR2W5	OR8B12	FAM55D	PSCA	N4BP1	
ODZ2	FAM173B	ZP4	MS4A13	ECE1	GIF	
LCE2A	EFNA4	MIR493	LOC728606	PDGFC	TCEB1	
SLC22A18	EGFR	ATXN7L1	MAPK13	ADAMTS3	CAMTA2	
APOBEC1	GABRG3	GABRB3	ZNF365	CHRM3	GPR162	
OR2A5	KIAA0182	NDUFAF3	C3orf25	PTCH2	PTPRG	
SELI	SIPA1L1	CLPTM1L	C1orf14	FSTL4	MIR889	
CCR7	PTN	DNAJB13	KIF2B	CREB3L2	GORASP2	
44259	FBXO32	OR2W1	C1orf68	PRDM16	CD38	
C1orf128	EIF2AK4	KIF13A	SH3PXD2A	TECR	NEFM	
DSC2	INPP5A	LRRC7	ASB1	PDYN	C20orf79	
BZW2	TMEM132D	FLJ22536	SORCS2	RND3	TMEM48	
GAPDH	OR6Y1	HNRNPA3P1	VPS52	FSHR	JAM3	
BCAT1	ZNF423	TRABD	CRTAC1	PSMB11	MAP4	
S100A11	KCNIP1	C1RL	CHGA	MARK4	BPIL3	
BCL9	TIMP2	LCE3D	LOC400794	FAM171A1	ARL14	
GRIP1	C18orf20	PXDN	C8orf73	RHOV	MIR200B	
NAV1	ATRNL1	RPL39L	PURG	APOL5	BNC1	
TRAM1	LILRA4	HCRT	HTR5A	ANKDD1A	PMP22	
KCNMA1	DNAH14	GLI3	CIB3	PFKP	IGFL2	
CPVL	BEND5	RORA	RNF19A	CAPN11	AIG1	
STAT4	ANKRD22	OPN5	CA10	EDN3	TCF15	
CLSTN2	CRCP	MYO7B	OR10J5	ACTR1B	BMP10	
CDH11	CLNK	CREB5	KCP	INS-IGF2	ANK3	
SLURP1	TCAP	CDCP2	CCL11	HCG22	TSGA14	
CACNG5	ELL3	COL17A1	FURIN	MFAP5	C1orf158	
COX10	OCIAD2	NMUR2	KCNU1	KLF12	CD1B	
KRTAP20-1	SLC4A11	DLG2	GTF2IRD1	PARP12	NCRUPAR	
F11R	LOC150622	SLC26A4	OR4D5	AIM1	TM4SF19	
MX2	PRNP	TRIM4	GPR123	CYTH1	RPSAP52	
TRIM27	FAM19A2	NFASC	LOC285733	LVRN	STK19	
PFKFB3	CRTAM	OR10J1	SNCAIP	SPRR4	IGF2R	
CCDC162	TLN2	LYPD1	EAPP	LYN	SRGAP1	
KRTAP13-1	SLC6A18	AIM2	KCNH5	KIF25	SNORD24	
ZBED2	NEDD9	LY86	GYG1	CCDC46	TFF1	
LOC100130331	OR10Z1	CACNA1A	C1orf183	ANXA2	LOC730755	

GENE	GENE	GENE	GENE	GENE	GENE	GENE
EPN3	OR51E1	S100A7L2	CD84	PRAMEF12	PPP1R13L	
PRKCA	C2orf3	POTEA	THADA	PDE9A	SLC17A2	
LOC731789	COL4A2	EIF3G	TRPC7	NEK10	LOC100133612	
REG1P	PATE1	APOB	LPA	SCGB1D4	MIR1295	
44448	SUFU	GBAP1	RPS3	RGS4	ALDH3B1	
TMEM132B	SNTG2	BHLHE40	TMEM151A	TGM5	SEC23B	
FAM40A	C22orf43	TMPRSS13	SPATS2	LOC286094	SLC4A4	
VAC14	FLJ43663	TUBA1C	MAP4K4	MAP3K4	DMRT3	
