# Supplementary material for: DNA damage repair-related methylated genes RRM2 and GAPDH are prognostic biomarkers associated with immunotherapy for lung adenocarcinoma
Source: Genet Mol Biol. 2025 May 9;48(2):e20240138. doi: 10.1590/1678-4685-GMB-2024-0138 (PMC12063672; doi:10.1590/1678-4685-GMB-2024-0138)
Supplement: Table S8 - [file 1415-4757-GMB-48-02-e20240138-s9.pdf]

Genetics and Molecular Biology
https://doi.org/10.1590/1678-4685-GMB-2024-0138

# Supplementary Material to "DNA damage repair-related methylated genes RRM2 and GAPDH are prognostic biomarkers associated with immunotherapy for lung adenocarcinoma"

**Table S8 -** Summary of the results from the univariate Cox regression analysis in TCGA-LUAD patients.

| gene | KM.Pvalue | HR | HR.95L | HR.95H | pvalue |
|---|---|---|---|---|---|
| BUB1B | 0.000625 | 1.308031 | 1.123680 | 1.522626 | 0.000531 |
| SHCBP1 | 0.001585 | 1.443509 | 1.196051 | 1.742166 | 0.000130 |
| RRM2 | 0.000271 | 1.339812 | 1.175718 | 1.526809 | 1.14E-05 |
| TRIP13 | 0.005282 | 1.192771 | 1.039981 | 1.368009 | 0.011719 |
| GAPDH | 0.002148 | 1.592680 | 1.325413 | 1.913842 | 6.84E-07 |
| ENO1 | 0.044467 | 1.406935 | 1.087539 | 1.820134 | 0.009357 |
