# Supplementary material for: DNA damage repair-related methylated genes RRM2 and GAPDH are prognostic biomarkers associated with immunotherapy for lung adenocarcinoma
Source: Genet Mol Biol. 2025 May 9;48(2):e20240138. doi: 10.1590/1678-4685-GMB-2024-0138 (PMC12063672; doi:10.1590/1678-4685-GMB-2024-0138)
Supplement: Table S9 - [file 1415-4757-GMB-48-02-e20240138-s10.pdf]

Genetics and Molecular Biology
https://doi.org/10.1590/1678-4685-GMB-2024-0138

Supplementary Material to “DNA damage repair-related methylated genes RRM2 and GAPDH are prognostic biomarkers associated with immunotherapy for lung adenocarcinoma”

Table S9 - Summary of the results from the multivariate Cox regression analysis in TCGA-LUAD patients.

ID	coef	HR	HR.95L	HR.95H	pvalue
RRM2	0.164974	1.179362	1.006266	1.382233	0.041640
GAPDH	0.338623	1.403014	1.124856	1.749957	0.002669
