# Supplementary material for: DNA damage repair-related methylated genes RRM2 and GAPDH are prognostic biomarkers associated with immunotherapy for lung adenocarcinoma
Source: Genet Mol Biol. 2025 May 9;48(2):e20240138. doi: 10.1590/1678-4685-GMB-2024-0138 (PMC12063672; doi:10.1590/1678-4685-GMB-2024-0138)
Supplement: Table S10 - [file 1415-4757-GMB-48-02-e20240138-s11.pdf]

Genetics and Molecular Biology
https://doi.org/10.1590/1678-4685-GMB-2024-0138

Supplementary Material to “DNA damage repair-related methylated genes RRM2 and GAPDH are prognostic biomarkers associated with immunotherapy for lung adenocarcinoma”

Table S10 - Clinical information, risk score, and grouping information related to TCGA-LUAD patients.

ID	futime	fustat	age	gender	stage	T	M	N	RRM2	GAPDH	riskScore	Risk
TCGA-05-4244-01A	0	0	70	male	iv	T2	M1	N2	3.924315	9.964048	4.021465	High
TCGA-05-4249-01A	1523	0	67	male	i	T2	M0	N0	1.798772	8.406302	3.143318	Low
TCGA-05-4250-01A	121	1	79	female	iii	T3	M0	N1	4.885141	9.542872	4.037356	High
TCGA-05-4382-01A	607	0	68	male	i	T2	M0	N0	2.784996	9.684646	3.738895	High
TCGA-05-4384-01A	426	0	66	male	iii	T2	M0	N2	1.821073	9.112219	3.386036	Low
TCGA-05-4389-01A	1369	0	70	male	i	T1	M0	N0	3.712687	9.658092	3.882948	High
TCGA-05-4390-01A	1126	0	58	female	i	T2	M0	N0	4.249345	9.975734	4.079043	High
TCGA-05-4395-01A	0	1	76	male	iii	T4	M0	N2	4.26542	12.21144	4.838755	High
TCGA-05-4396-01A	303	1	76	male	iii	T4	M0	N1	2.477436	8.623859	3.328949	Low
TCGA-05-4397-01A	731	1	65	male	ii	T2	M0	N1	5.483371	9.41279	4.091999	High
TCGA-05-4398-01A	1431	0	47	female	iii	T4	M0	N3	4.061957	11.17554	4.45441	High
TCGA-05-4402-01A	244	1	57	female	iv	T2	M1	NX	3.198369	10.30563	4.017372	High
TCGA-05-4403-01A	578	0	76	male	i	T2	M0	N0	2.82346	9.276899	3.607168	Low
TCGA-05-4405-01A	610	0	74	female	i	T2	M0	N0	2.361295	8.135477	3.144411	Low
TCGA-05-4410-01A	0	0	62	male	i	T2	M0	N0	2.325899	8.825656	3.372283	Low
TCGA-05-4415-01A	91	1	57	male	iii	T4	M0	N2	4.634166	10.98116	4.482991	High
TCGA-05-4417-01A	455	0	51	female	i	T2	M0	N0	2.858344	9.42849	3.664255	Low
TCGA-05-4418-01A	274	1	69	male	iii	T3	M0	N2	3.978039	10.9374	4.359926	High
TCGA-05-4420-01A	912	0	41	male	i	T2	M0	N0	5.280241	9.062578	3.939898	High
TCGA-05-4422-01A	365	0	68	male	i	T2	M0	N0	2.206526	9.141226	3.459448	Low
TCGA-05-4424-01A	913	0	70	male	ii	T3	M0	N0	3.562576	9.424538	3.779097	High
TCGA-05-4425-01A	669	0	70	female	iv	T2	M1	N0	2.914946	8.71334	3.431427	Low
TCGA-05-4426-01A	791	0	71	male	i	T2	M0	N0	3.435127	10.64233	4.170442	High
TCGA-05-4427-01A	791	0	65	female	ii	T2	M0	N1	3.835093	8.854723	3.631102	Low
TCGA-05-4430-01A	761	0	59	female	i	T2	M0	N0	2.825304	9.143135	3.562177	Low
TCGA-05-4432-01A	761	0	66	male	ii	T2	M0	N1	3.771014	8.953401	3.653946	Low
TCGA-05-4433-01A	730	0	82	male	i	T2	M0	N0	3.596754	9.1636	3.696376	Low
TCGA-05-4434-01A	457	1	67	female	iv	T4	M1	N1	4.0851	10.44665	4.211411	High
TCGA-05-5420-01A	457	0	67	male	iii	T2	M0	N2	3.919126	9.132426	3.739002	High

ID	futime	fustat	age	gender	stage	T	M	N	RRM2	GAPDH	riskScore	Risk
TCGA-05-5423-01A	151	0	65	male	ii	T2	M0	N1	2.322328	8.034552	3.103807	Low
TCGA-05-5425-01A	882	0	68	male	ii	T2	M0	N1	4.344195	9.101102	3.79852	High
TCGA-05-5428-01A	670	0	57	male	ii	T1	M0	N1	4.048244	9.548747	3.901279	High
TCGA-05-5429-01A	275	1	60	male	iii	T3	M0	N2	4.193922	10.00255	4.07898	High
TCGA-05-5715-01A	62	0	69	female	i	T2	M0	N0	3.041032	9.333185	3.662122	Low
TCGA-35-3615-01A	14	0	57	male	i	T2	M0	N0	1.561022	9.357478	3.426185	Low
TCGA-35-4122-01A	225	0	69	male	i	T1	M0	N0	4.66352	10.41087	4.29472	High
TCGA-35-4123-01A	182	0	38	male	i	T1	M0	N0	4.505647	10.72243	4.374176	High
TCGA-35-5375-01A	264	0	61	male	iii	T2	M0	N2	3.983972	10.67265	4.271257	High
TCGA-38-4625-01A	2973	0	66	female	i	T2	M0	N0	4.620372	9.874652	4.106024	High
TCGA-38-4627-01A	1147	1	64	female	ii	T1	M0	N1	2.740433	9.666989	3.725564	High
TCGA-38-4628-01A	1492	1	65	female	ii	T2	M0	N1	3.527256	8.433282	3.437608	Low
TCGA-38-4629-01A	864	1	68	male	ii	T3	M0	N0	3.489232	10.71511	4.204014	High
TCGA-38-4630-01A	1073	1	75	female	i	T2	M0	N0	5.070905	9.509333	4.056645	High
TCGA-38-4631-01A	354	1	72	female	i	T2	M0	N0	5.430311	10.40949	4.420751	High
TCGA-38-4632-01A	1357	1	42	male	iv	T2	M1	N1	4.395025	9.921225	4.084619	High
TCGA-38-7271-01A	800	1	72	female	i	T1	M0	N0	2.508818	8.541539	3.306251	Low
TCGA-38-A44F-01A	133	0	80	male	i	T2	M0	N0	2.000292	9.470592	3.536956	Low
TCGA-44-2655-01A	1324	0	65	female	i	T1	M0	N0	1.644669	8.213385	3.052569	Low
TCGA-44-2656-01A	1429	0	59	male	i	T2	M0	N0	3.456356	7.727089	3.186778	Low
TCGA-44-2657-01A	1351	0	74	female	i	T2	M0	NX	2.647325	8.570664	3.338963	Low
TCGA-44-2659-01A	1367	0	65	female	ii	T1	M0	N1	1.595682	8.503134	3.142603	Low
TCGA-44-2661-01A	1159	0	69	female	i	T1	M0	N0	2.689429	8.316958	3.259998	Low
TCGA-44-2662-01A	1280	0	65	male	i	T2	M0	N0	4.484388	9.841316	4.072302	High
TCGA-44-2665-01A	1301	0	55	female	ii	T2	M0	N1	2.232904	9.743763	3.667833	Low
TCGA-44-2666-01A	97	1	43	male	i	T2	M0	N0	3.204245	7.818804	3.176243	Low
TCGA-44-2668-01A	761	1	51	male	i	T2	M0	N0	4.065208	10.58508	4.255005	High
TCGA-44-3396-01A	1130	0	74	female	iii	T2	M0	N2	4.175984	9.120243	3.777252	High
TCGA-44-3398-01A	1163	0	77	female	i	T1	M0	N0	3.296832	10.11958	3.970614	High
TCGA-44-3917-01A	1183	0	33	female	i	T2	M0	N0	4.312605	9.020811	3.766121	High
TCGA-44-3918-01A	1036	0	60	female	i	T1	M0	N0	3.470812	7.927348	3.256975	Low
TCGA-44-3919-01A	1026	1	71	female	i	T1	M0	N0	3.617395	9.540547	3.827424	High
TCGA-44-4112-01A	808	1	60	female	i	T2	M0	N0	3.510317	9.993884	3.963269	High
TCGA-44-5643-01A	1013	0	53	male	iii	T2	M0	N2	4.506192	11.51814	4.643709	High
TCGA-44-6145-01A	595	0	62	female	i	T1	M0	N0	3.635435	9.526637	3.82569	High
TCGA-44-6146-01A	728	0	64	male	ii	T3	M0	N0	2.224825	7.711543	2.978344	Low
TCGA-44-6147-01A	845	0	67	female	i	T1	M0	NX	1.863711	7.605092	2.882723	Low
TCGA-44-6148-01A	704	0	60	male	i	T1	M0	N0	0.694646	8.363273	2.946595	Low
TCGA-44-6774-01A	658	0	56	female	iii	T1	M0	N2	3.453834	8.935475	3.595549	Low
TCGA-44-6775-01A	705	0	72	female	i	T2	MX	N0	3.860746	8.548367	3.531595	Low

ID	futime	fustat	age	gender	stage	T	M	N	RRM2	GAPDH	riskScore	Risk
TCGA-44-6776-01A	2616	0	60	female	i	T1	MX	N0	1.736114	8.531704	3.175445	Low
TCGA-44-6777-01A	987	1	85	female	i	T2	MX	NX	2.784277	9.861928	3.798808	High
TCGA-44-6778-01A	1864	0	59	male	i	T1	MX	N0	3.586818	8.679939	3.530958	Low
TCGA-44-6779-01A	500	1	50	female	ii	T2	MX	N1	4.930516	10.62883	4.412571	High
TCGA-44-7659-01A	691	0	70	male	i	T1	MX	N0	1.477769	8.023573	2.96076	Low
TCGA-44-7660-01A	592	0	72	male	i	T2	MX	N0	3.376744	10.58046	4.139863	High
TCGA-44-7661-01A	557	1	69	female	i	T2	M0	N0	4.429629	10.37881	4.245277	High
TCGA-44-7662-01A	218	0	61	male	i	T2	MX	N0	3.903666	9.51994	3.867673	High
TCGA-44-7667-01A	1097	0	49	female	ii	T3	MX	N0	5.875283	10.86684	4.649028	High
TCGA-44-7669-01A	574	1	59	male	ii	T1	MX	N1	5.503315	10.81355	4.569618	High
TCGA-44-7670-01A	882	0	47	female	ii	T1	M0	N1	5.022158	9.575053	4.070857	High
TCGA-44-7671-01A	889	0	64	male	i	T2	M0	N0	2.126198	9.630925	3.61202	Low
TCGA-44-7672-01A	719	0	52	female	i	T1	M0	N0	3.624139	10.01555	3.989384	High
TCGA-44-8117-01A	385	0	54	female	i	T2	M0	N0	2.633293	9.343701	3.598417	Low
TCGA-44-8119-01A	285	0	73	male	ii	T3	M0	N0	3.880509	10.12118	4.067447	High
TCGA-44-8120-01A	260	0	58	male	i	T2	M0	N0	1.876807	8.876832	3.315524	Low
TCGA-44-A479-01A	486	0	73	female	i	T2	MX	N0	3.694019	8.592535	3.519046	Low
TCGA-44-A47A-01A	466	0	78	female	i	T2	MX	N0	3.383422	9.599452	3.808771	High
TCGA-44-A47B-01A	287	0	79	male	i	T2	M0	N0	2.782038	8.715636	3.410278	Low
TCGA-44-A47G-01A	351	0	73	female	i	T1	M0	N0	3.748679	9.582713	3.863361	High
TCGA-44-A4SS-01A	415	0	73	male	i	T1	M0	N0	3.845612	9.636701	3.897634	High
TCGA-44-A4SU-01A	409	1	67	female	i	T1	MX	N0	2.663168	8.566548	3.340183	Low
TCGA-49-4486-01A	2318	1	72	male	i	T1	M0	N0	1.094096	9.292904	3.327289	Low
TCGA-49-4487-01A	855	1	72	female	i	T1	M0	N0	4.057785	10.11886	4.095906	High
TCGA-49-4488-01A	869	1	74	female	i	T1	MX	N0	3.524414	9.530548	3.808699	High
TCGA-49-4490-01A	385	1	45	female	iii	T3	M0	N2	2.667354	9.555696	3.675822	Low
TCGA-49-4494-01A	1081	1	77	male	iii	T3	M0	N2	4.281809	10.30178	4.194807	High
TCGA-49-4501-01A	1421	1	67	female	i	T2	M0	N0	2.677808	8.574511	3.345295	Low
TCGA-49-4505-01A	428	1	61	female	ii	T2	M0	N1	1.580691	9.909667	3.616414	Low
TCGA-49-4506-01A	999	1	68	female	ii	T2	M0	N1	4.589772	11.162	4.536903	High
TCGA-49-4507-01A	268	1	73	female	iii	T3	M0	N1	3.940932	11.2074	4.445232	High
TCGA-49-4510-01A	896	1	51	female	ii	T2	M0	N1	0.858442	8.642756	3.068257	Low
TCGA-49-4512-01A	905	1	69	female	iii	T2	MX	N2	2.362683	9.340534	3.552701	Low
TCGA-49-4514-01A	1700	0	79	female	i	T1	M0	N0	3.58765	10.19986	4.045776	High
TCGA-49-6742-01A	488	1	70	male	ii	T2	M0	N1	4.53812	9.357448	3.917318	High
TCGA-49-6743-01A	1621	0	81	female	iii	T1	MX	N2	4.834644	9.271968	3.937291	High
TCGA-49-6744-01A	1683	0	64	female	ii	T2	MX	N1	2.80049	9.185003	3.572261	Low
TCGA-49-6745-01A	522	0	82	male	iii	T2	M0	N2	4.456594	9.37872	3.911071	High
TCGA-49-6761-01A	354	0	68	female	iii	T1	MX	N2	3.203015	9.595311	3.777607	High
TCGA-49-6767-01A	677	0	46	female	ii	T3	MX	N0	4.141578	10.01228	4.07364	High

ID	futime	fustat	age	gender	stage	T	M	N	RRM2	GAPDH	riskScore	Risk
TCGA-49-AAQV-01A	677	1	63	female	ii	T1	MX	N1	3.929503	9.284152	3.792092	High
TCGA-49-AAR0-01A	4765	0	57	male	i	T1	MX	N0	3.041411	9.311681	3.654902	Low
TCGA-49-AAR2-01A	2224	0	64	male	i	T2	MX	N0	2.970806	9.732464	3.785741	High
TCGA-49-AAR3-01A	1893	0	69	male	ii	T2	MX	N1	4.180534	10.02764	4.085267	High
TCGA-49-AAR4-01A	879	1	51	male	iii	T2	MX	N2	3.683436	9.01336	3.659801	Low
TCGA-49-AAR9-01A	260	1	61	male	ii	T3	MX	N0	5.093135	10.52688	4.404876	High
TCGA-49-AARE-01A	1229	1	51	female	i	T1	MX	N0	4.124853	9.683084	3.959408	High
TCGA-49-AARN-01A	1135	1	56	female	i	T1	MX	N0	3.282712	9.116148	3.628499	Low
TCGA-49-AARO-01A	3759	0	39	female	i	T1	MX	N0	3.176334	10.05735	3.929663	High
TCGA-49-AARQ-01A	6732	0	41	female	i	T2	MX	N0	4.475822	10.47911	4.286862	High
TCGA-49-AARR-01A	4992	0	68	male	i	T1	MX	N0	1.390551	7.904534	2.906062	Low
TCGA-4B-A93V-01A	300	1	52	female	i	T1	M0	N0	2.243105	10.08875	3.786338	High
TCGA-50-5044-01A	624	1	72	female	iii	T4	M0	N1	4.555746	11.35825	4.597745	High
TCGA-50-5049-01A	3094	0	70	male	i	T2	M0	N0	3.12125	9.172765	3.621034	Low
TCGA-50-5051-01A	478	1	42	female	iii	T2	M0	N2	2.748511	9.193378	3.566521	Low
TCGA-50-5055-01A	1830	1	79	female	ii	T1	M0	N1	3.023923	9.256808	3.633436	Low
TCGA-50-5066-01A	1442	0	72	male	i	T2	M0	N0	4.543977	10.15113	4.187041	High
TCGA-50-5068-01A	1499	1	59	female	ii	T2	MX	N1	3.330667	8.698978	3.495147	Low
TCGA-50-5072-01A	250	1	74	male	iii	T2	M0	N2	4.971997	10.31766	4.314046	High
TCGA-50-5930-01A	282	1	47	male	iii	T2	M0	N2	3.014127	8.999488	3.544686	Low
TCGA-50-5931-01A	434	1	75	female	i	T2	M0	N0	4.343787	10.82162	4.381061	High
TCGA-50-5932-01A	1235	1	75	male	ii	T2	M0	N1	2.359948	8.86048	3.389692	Low
TCGA-50-5933-01A	2393	1	72	male	iii	T4	M0	N2	3.412489	9.479381	3.772908	High
TCGA-50-5935-01A	653	1	86	female	i	T1	M0	N0	1.427584	7.846092	2.892381	Low
TCGA-50-5936-01A	257	1	58	male	iii	T2	M0	N2	3.699123	10.28288	4.092278	High
TCGA-50-5939-01A	460	1	85	male	i	T2	M0	N0	3.949374	11.20852	4.447007	High
TCGA-50-5941-01A	1474	0	55	female	iii	T2	M0	N2	3.579678	10.19823	4.04391	High
TCGA-50-5942-01A	1847	0	67	female	i	T1	M0	N0	0.996174	7.577007	2.730092	Low
TCGA-50-5944-01A	1750	0	69	female	i	T1	M0	N0	2.716903	8.845927	3.443652	Low
TCGA-50-5946-01A	1617	0	62	male	i	T1	MX	N0	5.171253	9.024289	3.908953	High
TCGA-50-6590-01A	1288	1	72	female	i	T2	M0	N0	4.000264	11.31718	4.492195	High
TCGA-50-6591-01A	119	1	63	female	iv	T2	M1	N0	5.047526	10.17012	4.276545	High
TCGA-50-6592-01A	777	1	71	female	i	T2	M0	N0	4.672257	10.26185	4.2457	High
TCGA-50-6593-01A	336	1	49	female	iii	T1	M0	N2	3.088868	9.183349	3.619275	Low
TCGA-50-6594-01A	370	1	79	female	iii	T3	M0	N2	4.850856	9.701761	4.085503	High
TCGA-50-6595-01A	189	1	74	female	iii	T2	M0	N2	4.38721	10.49905	4.278993	High
TCGA-50-6597-01A	1268	1	79	female	i	T2	M0	N0	1.931373	8.92626	3.341263	Low
TCGA-50-6673-01A	22	1	84	female	i	T1	M0	N0	3.497807	9.05932	3.644741	Low
TCGA-50-7109-01A	308	1	60	male	i	T1	M0	N0	3.637533	9.382876	3.777355	High
TCGA-50-8457-01A	1125	0	63	female	i	T1	M0	N0	0.98145	8.20394	2.939957	Low

ID	futime	fustat	age	gender	stage	T	M	N	RRM2	GAPDH	riskScore	Risk
TCGA-50-8459-01A	1119	0	68	male	ii	T3	M0	N0	1.974515	9.589942	3.573118	Low
TCGA-50-8460-01A	829	0	74	male	i	T1	M0	N0	2.379839	9.755252	3.695964	Low
TCGA-53-7624-01A	1043	1	40	female	iv	T2	M1	N0	4.930855	9.75826	4.117833	High
TCGA-53-7626-01A	929	1	76	female	ii	T1	M0	N1	2.61724	8.521947	3.317503	Low
TCGA-53-7813-01A	424	0	51	female	iii	T4	M0	N0	2.782853	9.223336	3.582331	Low
TCGA-53-A4EZ-01A	1071	0	63	male	ii	T2	MX	N1	4.083506	8.845099	3.668825	Low
TCGA-55-1592-01A	701	1	65	male	i	T2	M0	N0	3.744757	8.802812	3.598621	Low
TCGA-55-1594-01A	1178	0	68	male	iii	T2	M0	N2	5.061809	9.538195	4.064918	High
TCGA-55-1596-01A	2065	0	55	male	ii	T2	M0	N1	4.525977	9.715844	4.036676	High
TCGA-55-6543-01A	435	0	60	female	i	T1	MX	N0	1.925012	9.257307	3.452314	Low
TCGA-55-6642-01A	2449	0	63	male	i	T2	MX	N0	3.548107	9.463377	3.789862	High
TCGA-55-6712-01A	171	1	71	male	ii	T2	MX	N1	3.001906	10.42682	4.025996	High
TCGA-55-6968-01A	1293	1	61	male	iv	T1	M1	N0	5.23561	9.448682	4.063279	High
TCGA-55-6970-01A	464	1	67	female	iii	T2	MX	N2	4.109825	9.209922	3.796705	High
TCGA-55-6971-01A	1400	0	59	female	i	T2	MX	N0	3.253503	9.517655	3.759639	High
TCGA-55-6972-01A	1632	1	72	male	i	T2	M0	N0	1.662146	8.677874	3.212738	Low
TCGA-55-6975-01A	118	1	61	male	ii	T2	M0	N1	4.924456	10.21173	4.270334	High
TCGA-55-6978-01A	176	1	81	male	ii	T2	MX	N0	4.840876	9.896178	4.149691	High
TCGA-55-6979-01A	237	1	59	female	ii	T2	M0	N1	3.513542	9.033119	3.638464	Low
TCGA-55-6980-01A	2109	0	56	male	i	T1	M0	N0	1.529301	9.609088	3.506153	Low
TCGA-55-6981-01A	1379	1	53	female	iii	T1	M0	N2	3.910829	10.46895	4.190213	High
TCGA-55-6982-01A	995	1	79	female	ii	T2	M0	N1	3.531775	10.17378	4.027727	High
TCGA-55-6983-01A	2823	0	81	male	ii	T2	M0	N1	2.955523	9.292521	3.634245	Low
TCGA-55-6984-01A	760	1	71	female	ii	T2	M0	N1	3.888102	10.74312	4.279303	High
TCGA-55-6985-01A	1233	0	58	female	i	T2	MX	N0	3.484549	9.373239	3.748854	High
TCGA-55-6986-01A	3261	0	74	female	i	T2	M0	N0	2.791989	9.368773	3.633087	Low
TCGA-55-6987-01A	2137	0	77	male	i	T1	M0	N0	3.438944	10.29938	4.054942	High
TCGA-55-7227-01A	952	1	77	male	iii	T3	MX	N1	2.79808	9.071556	3.533447	Low
TCGA-55-7281-01A	872	0	70	female	i	T1	M0	N0	2.40627	9.334965	3.558005	Low
TCGA-55-7283-01A	609	0	76	female	iii	T3	MX	N2	3.187791	8.338629	3.349553	Low
TCGA-55-7284-01B	243	1	74	male	ii	T3	MX	N0	2.075734	8.672986	3.279314	Low
TCGA-55-7570-01A	824	0	60	male	i	T1	MX	N0	4.725512	10.28176	4.261226	High
TCGA-55-7573-01A	487	0	72	female	i	T1	MX	N0	1.529991	8.239036	3.042336	Low
TCGA-55-7574-01A	995	1	64	female	i	T2	M0	N0	2.71888	8.98623	3.491488	Low
TCGA-55-7576-01A	670	0	54	male	i	T2	M0	N0	4.017784	9.948627	4.031663	High
TCGA-55-7724-01A	705	0	76	female	i	T2	MX	N0	3.522996	9.382367	3.758287	High
TCGA-55-7725-01A	442	0	68	female	i	T1	MX	N0	2.103819	8.937783	3.373614	Low
TCGA-55-7726-01A	652	0	72	female	i	T1	MX	N0	3.739621	10.11586	4.042401	High
TCGA-55-7727-01A	119	0	70	male	iii	T1	MX	N2	3.884768	8.480125	3.51245	Low
TCGA-55-7728-01A	704	0	64	female	i	T2	MX	N0	2.172294	9.24928	3.490391	Low

ID	futime	fustat	age	gender	stage	T	M	N	RRM2	GAPDH	riskScore	Risk
TCGA-55-7815-01A	773	0	76	male	i	T2	MX	N0	3.326732	8.426931	3.402376	Low
TCGA-55-7816-01A	468	1	49	female	iv	TX	MX	NX	1.746946	8.482858	3.160691	Low
TCGA-55-7903-01A	567	0	64	male	i	T1	MX	N0	4.716139	10.43168	4.310445	High
TCGA-55-7907-01A	343	1	77	male	ii	T2	MX	N1	2.998059	9.158825	3.59599	Low
TCGA-55-7910-01A	1040	0	50	female	ii	T2	M0	N0	4.093654	9.425104	3.866903	High
TCGA-55-7911-01A	537	0	70	female	i	T1	MX	N0	5.017449	10.4309	4.359889	High
TCGA-55-7913-01B	561	1	61	female	i	T1	MX	N0	3.677005	9.595314	3.855803	High
TCGA-55-7914-01A	187	1	71	female	ii	T1	MX	N1	3.089507	8.584528	3.416606	Low
TCGA-55-7994-01A	603	0	81	male	ii	T3	MX	N0	5.003131	10.02021	4.218458	High
TCGA-55-7995-01A	889	0	73	female	i	T1	M0	N0	4.181429	10.52991	4.255497	High
TCGA-55-8085-01A	904	0	64	male	i	T1	M0	N0	3.846358	10.19059	4.085317	High
TCGA-55-8087-01A	462	0	59	female	i	T2	MX	N0	0.913459	7.849755	2.808804	Low
TCGA-55-8089-01A	702	1	56	male	i	T1	M0	N0	4.575428	9.971573	4.131429	High
TCGA-55-8090-01A	598	1	80	male	i	T1	M0	N0	3.595592	10.05986	3.999677	High
TCGA-55-8091-01A	600	0	74	male	i	T2	MX	N0	2.732663	9.758837	3.755385	High
TCGA-55-8092-01A	154	1	75	male	ii	T3	MX	N0	2.632717	8.85382	3.432437	Low
TCGA-55-8094-01A	541	0	51	male	iv	T2	M1	N0	4.776251	9.960009	4.160644	High
TCGA-55-8096-01A	719	1	67	female	i	T2	MX	N0	2.81834	9.628058	3.725234	High
TCGA-55-8097-01A	476	0	60	female	i	T1	MX	N0	1.015163	7.843897	2.823599	Low
TCGA-55-8203-01A	547	0	69	female	i	T1	M0	N0	3.646523	9.471912	3.808988	High
TCGA-55-8204-01A	515	0	87	female	i	T2	MX	N0	3.978628	10.6079	4.248449	High
TCGA-55-8205-01A	599	0	76	female	ii	T2	M0	N0	5.097378	9.667085	4.114431	High
TCGA-55-8206-01A	888	0	56	male	i	T1	M0	N0	1.486052	7.899575	2.920137	Low
TCGA-55-8207-01A	977	0	73	male	i	T2	MX	N0	1.199941	8.840673	3.191614	Low
TCGA-55-8208-01A	674	0	73	female	i	T1	M0	N0	3.883845	9.341726	3.804056	High
TCGA-55-8299-01A	469	1	61	female	i	T1	MX	N0	3.861527	9.701568	3.922225	High
TCGA-55-8301-01A	534	0	58	male	i	T2	MX	N0	2.984205	9.901825	3.845301	High
TCGA-55-8302-01A	478	0	54	male	i	T2	MX	N0	3.950046	10.48319	4.201502	High
TCGA-55-8505-01A	440	0	62	male	iii	T1	MX	N2	4.195166	10.26741	4.168874	High
TCGA-55-8506-01A	11	0	62	female	ii	T3	MX	N0	2.660062	9.293055	3.585683	Low
TCGA-55-8507-01A	418	0	53	male	i	T1	MX	N0	3.571607	9.143827	3.685531	Low
TCGA-55-8508-01A	617	0	60	female	ii	T2	MX	N1	2.978953	9.581164	3.735852	High
TCGA-55-8510-01A	539	0	55	female	i	T2	MX	N0	2.978561	8.982775	3.533159	Low
TCGA-55-8511-01A	552	0	73	female	i	T2	MX	N0	3.686443	9.029791	3.665861	Low
TCGA-55-8512-01A	607	1	41	male	iv	T1	M1	N1	1.03258	8.148331	2.929561	Low
TCGA-55-8513-01A	791	0	77	female	ii	T3	MX	N0	1.747929	8.263025	3.086413	Low
TCGA-55-8514-01A	520	0	70	female	i	T2	MX	N0	1.067551	8.945362	3.205224	Low
TCGA-55-8614-01A	536	0	76	male	i	T2	MX	N0	2.817472	9.16609	3.568658	Low
TCGA-55-8615-01A	446	0	67	male	iii	T3	MX	N2	2.905377	9.300859	3.628796	Low
TCGA-55-8616-01A	48	0	58	female	i	T2	M0	N0	1.775149	8.809827	3.276063	Low

ID	futime	fustat	age	gender	stage	T	M	N	RRM2	GAPDH	riskScore	Risk
TCGA-55-8619-01A	416	0	72	female	ii	T3	MX	N0	1.952627	8.645457	3.249683	Low
TCGA-55-8620-01A	375	1	60	male	iv	T1	M1	N1	4.243831	10.21074	4.157713	High
TCGA-55-8621-01A	515	0	75	female	i	T1	MX	N0	2.516175	8.847706	3.41114	Low
TCGA-55-A48X-01A	689	0	63	female	ii	T1	M0	N1	2.94764	8.530727	3.374984	Low
TCGA-55-A48Y-01A	630	0	69	male	ii	T2	M0	N0	2.997252	9.041212	3.55603	Low
TCGA-55-A48Z-01A	651	0	60	female	iii	T1	MX	N3	3.182073	9.63677	3.788191	High
TCGA-55-A490-01A	99	1	78	male	ii	T2	MX	N0	3.278364	10.41779	4.068546	High
TCGA-55-A491-01A	626	0	81	female	i	T1	MX	N0	4.022953	9.539155	3.893859	High
TCGA-55-A492-01A	596	0	70	female	i	T1	MX	N0	1.025683	9.486041	3.381403	Low
TCGA-55-A493-01A	28	0	54	female	i	T2	M0	N0	4.835517	10.5839	4.381685	High
TCGA-55-A494-01A	481	0	61	female	i	T2	MX	N0	4.311954	9.572972	3.952988	High
TCGA-55-A4DF-01A	440	1	88	male	i	T1	MX	N0	4.402024	9.799213	4.044457	High
TCGA-55-A4DG-01A	608	0	71	male	i	T1	MX	N0	1.153295	7.357646	2.681732	Low
TCGA-55-A57B-01A	546	0	80	female	i	T1	M0	N0	2.420021	8.599472	3.311219	Low
TCGA-62-8394-01A	139	1	65	female	iii	T4	M0	N2	4.501756	9.384106	3.920346	High
TCGA-62-8395-01A	1216	0	80	female	ii	T3	M0	N0	1.555639	9.154869	3.356689	Low
TCGA-62-8397-01A	1289	0	70	female	ii	T3	M0	N0	1.645441	9.913421	3.628367	Low
TCGA-62-8398-01A	444	1	55	male	iii	T2	M0	N2	4.401082	11.06677	4.473527	High
TCGA-62-8399-01A	2696	0	62	male	iii	T2	M0	N2	3.431464	8.946681	3.595653	Low
TCGA-62-8402-01A	1498	1	73	female	iii	T2	M0	N2	4.937291	10.04227	4.215067	High
TCGA-62-A46O-01A	1454	1	65	female	i	T2	M0	N0	5.533939	9.293882	4.060077	High
TCGA-62-A46P-01A	594	1	65	male	i	T2	M0	N0	1.458694	9.023954	3.296365	Low
TCGA-62-A46R-01A	1725	1	54	female	i	T2	M0	N0	3.220027	7.480066	3.064142	Low
TCGA-62-A46S-01A	1653	1	73	male	i	T2	M0	N0	1.850684	8.352171	3.133552	Low
TCGA-62-A46V-01A	2199	0	78	female	i	T2	M0	N0	3.343448	9.375016	3.726177	High
TCGA-62-A46Y-01A	414	1	70	female	iii	T2	M0	N2	3.536858	9.412114	3.770647	High
TCGA-62-A470-01A	1194	1	84	male	i	T2	M0	N0	2.687818	9.331321	3.60322	Low
TCGA-62-A471-01A	1246	0	64	male	ii	T2	M0	N1	4.291959	10.38312	4.224022	High
TCGA-62-A472-01A	910	0	70	male	ii	T3	M0	N0	3.709352	9.786102	3.925745	High
TCGA-64-1676-01A	1728	0	58	male	i	T1	M0	N0	3.18924	9.581063	3.770509	High
TCGA-64-1677-01A	628	1	77	female	iii	T2	M0	N2	4.304276	10.07955	4.12326	High
TCGA-64-1679-01A	2488	0	58	female	iii	T1	M0	N2	3.371692	10.03052	3.952806	High
TCGA-64-1680-01A	1126	0	63	male	iv	T2	M1	N2	3.308153	8.961094	3.580191	Low
TCGA-64-1681-01A	1167	1	61	female	i	T1	M0	N0	2.390177	8.674584	3.33173	Low
TCGA-64-5774-01A	2676	0	60	male	i	T2	M0	N0	5.231526	9.966824	4.23806	High
TCGA-64-5775-01A	62	1	71	male	iii	T4	M0	N0	4.403973	11.144	4.500153	High
TCGA-64-5778-01A	1305	0	60	male	i	T2	M0	N0	3.692558	9.014105	3.661559	Low
TCGA-64-5779-01A	864	0	61	male	iii	T2	M0	N2	3.542881	9.044137	3.647035	Low
TCGA-64-5781-01A	1559	0	55	female	i	T2	M0	N0	4.028966	10.11439	4.089639	High
TCGA-64-5815-01A	866	0	74	male	ii	T2	M0	N1	3.251106	10.53576	4.103998	High

ID	futime	fustat	age	gender	stage	T	M	N	RRM2	GAPDH	riskScore	Risk
TCGA-67-3770-01A	610	0	70	female	i	T1	M0	N0	2.362696	8.440713	3.248003	Low
TCGA-67-3771-01A	610	0	77	female	i	T1	M0	N0	2.982745	9.16911	3.596946	Low
TCGA-67-3772-01A	573	0	82	female	i	T2	M0	N0	2.359791	9.234022	3.516156	Low
TCGA-67-3773-01A	427	0	84	female	i	T2	M0	N0	2.077526	8.991322	3.387406	Low
TCGA-67-3774-01A	385	0	73	female	i	T2	M0	N0	2.397429	8.107559	3.140919	Low
TCGA-67-6215-01A	174	0	52	female	i	T2	M0	N0	3.302877	9.505694	3.763735	High
TCGA-67-6216-01A	141	0	57	female	i	T1	M0	N0	3.176529	9.774921	3.834057	High
TCGA-67-6217-01A	422	0	73	female	ii	T2	M0	N1	1.196577	8.067567	2.929268	Low
TCGA-69-7760-01A	202	0	73	male	ii	T3	M0	N0	2.987053	9.604264	3.74501	High
TCGA-69-7761-01A	186	0	84	male	i	T2	MX	N0	3.548861	9.158973	3.686908	Low
TCGA-69-7763-01A	690	0	69	male	i	T1	M0	N0	1.998851	8.723429	3.283712	Low
TCGA-69-7764-01A	414	0	75	male	i	T1	M0	N0	3.029458	8.520844	3.385135	Low
TCGA-69-7973-01A	230	0	42	female	i	T2	M0	N0	4.281077	9.574705	3.948481	High
TCGA-69-7974-01A	184	0	54	female	iii	T2	MX	N2	4.370167	9.874465	4.064684	High
TCGA-69-7978-01A	134	0	59	male	ii	T2	MX	N1	2.777548	9.645663	3.724466	Low
TCGA-69-7979-01A	408	0	71	female	i	T2	MX	N0	2.880729	9.432442	3.669287	Low
TCGA-69-7980-01A	411	0	70	female	i	T1	M0	N0	3.71928	9.687707	3.894064	High
TCGA-69-8253-01A	426	0	59	female	ii	T1	MX	N1	1.279957	9.651712	3.479451	Low
TCGA-69-8255-01A	129	0	71	male	i	T1	M0	N0	4.714252	10.39819	4.298794	High
TCGA-69-8453-01A	813	0	77	male	ii	T3	MX	N0	3.053847	9.261473	3.639952	Low
TCGA-69-A59K-01A	591	0	60	female	ii	T3	M0	N0	3.933586	10.37382	4.161753	High
TCGA-71-6725-01A	256	0	48	female	i	T2	M0	N0	2.220605	8.478707	3.237427	Low
TCGA-71-8520-01A	210	1	60	female	i	T2	M0	N0	4.230346	9.057274	3.764897	High
TCGA-73-4658-01A	1600	1	80	female	i	T2	M0	N0	2.9268	9.642368	3.747973	High
TCGA-73-4659-01A	711	1	66	male	iii	T2	M0	N2	4.398244	9.984935	4.106723	High
TCGA-73-4662-01A	2515	0	65	female	i	T1	M0	N0	2.641554	8.402874	3.281193	Low
TCGA-73-4666-01A	800	0	52	female	iv	T1	M1	N0	5.193806	11.0151	4.586808	High
TCGA-73-4668-01A	467	0	66	female	ii	T2	M0	N1	3.782411	9.450833	3.824268	High
TCGA-73-4670-01A	131	0	69	female	iv	T2	M1	N0	4.98933	11.33231	4.66049	High
TCGA-73-4675-01A	922	1	59	male	iii	T3	M0	N1	2.730483	10.03085	3.847134	High
TCGA-73-4676-01A	281	1	45	male	ii	T2	M0	N1	5.468585	9.951452	4.271963	High
TCGA-73-7498-01A	1189	0	58	female	i	T1	M0	N0	0.968843	8.086545	2.898124	Low
TCGA-73-7499-01A	1531	1	81	female	i	T2	M0	N0	4.493689	8.792666	3.718739	Low
TCGA-73-A9RS-01A	340	1	41	male	ii	T3	M0	N0	6.034703	9.581358	4.240036	High
TCGA-78-7143-01A	4961	1	62	female	i	T2	M0	N0	4.001763	8.82269	3.647751	Low
TCGA-78-7145-01A	826	1	52	female	iv	T4	M1	N1	5.028279	9.341854	3.992901	High
TCGA-78-7146-01A	173	1	71	female	iii	T2	M0	N2	4.553495	10.95513	4.460866	High
TCGA-78-7147-01A	586	1	67	female	ii	T2	M0	N1	3.775161	9.135861	3.716415	Low
TCGA-78-7148-01A	626	1	71	male	ii	T2	M0	N1	3.992116	10.07847	4.071396	High
TCGA-78-7149-01A	3940	0	71	male	iii	T4	M0	N0	1.452826	9.422965	3.430511	Low

ID	futime	fustat	age	gender	stage	T	M	N	RRM2	GAPDH	riskScore	Risk
TCGA-78-7150-01A	666	1	59	male	ii	T2	M0	N1	5.053222	11.0075	4.561042	High
TCGA-78-7152-01A	1215	1	65	male	i	T2	M0	N0	2.610867	8.924243	3.452679	Low
TCGA-78-7153-01A	3635	0	65	female	i	T2	M0	N0	1.625666	8.548938	3.163059	Low
TCGA-78-7154-01A	593	1	72	male	iii	T3	M0	N2	3.992603	10.56882	4.23752	High
TCGA-78-7155-01A	1171	1	68	male	i	T2	M0	N0	5.282828	8.920661	3.892269	High
TCGA-78-7156-01A	976	1	62	male	iv	T4	M1	N1	1.422035	8.611447	3.150633	Low
TCGA-78-7158-01A	179	1	59	female	iii	T4	M0	N2	2.610074	9.291427	3.576885	Low
TCGA-78-7159-01A	1974	0	60	female	i	T1	M0	NX	3.413759	9.556314	3.799168	High
TCGA-78-7160-01A	697	1	61	male	iv	T4	M1	N2	4.418561	9.428178	3.921544	High
TCGA-78-7161-01A	291	1	69	female	ii	T3	M0	N0	1.629477	8.997719	3.315656	Low
TCGA-78-7162-01A	3169	1	75	male	i	T1	M0	N0	1.834253	8.293991	3.11114	Low
TCGA-78-7163-01A	7248	0	60	male	i	T2	M0	N0	1.724817	9.569198	3.5249	Low
TCGA-78-7166-01A	258	1	84	male	ii	T2	M0	N1	3.720294	10.65351	4.221273	High
TCGA-78-7167-01A	2681	1	77	male	iv	T2	M1	N0	0.552501	8.907378	3.107392	Low
TCGA-78-7220-01A	807	1	53	female	iii	T2	M0	N2	5.208941	9.672825	4.134779	High
TCGA-78-7535-01A	949	1	45	male	i	T2	M0	N0	2.877929	9.318331	3.630184	Low
TCGA-78-7536-01A	244	1	69	male	iii	T2	M0	N2	4.369835	10.08557	4.136115	High
TCGA-78-7537-01A	1622	1	72	male	i	T2	M0	N0	1.264366	8.51438	3.091752	Low
TCGA-78-7539-01A	791	0	75	female	ii	T2	M0	N0	1.417285	8.555977	3.131066	Low
TCGA-78-7540-01A	1197	1	66	female	i	T2	M0	N0	2.413951	8.547926	3.292763	Low
TCGA-78-7542-01A	321	1	56	male	i	T2	M0	N0	4.501632	10.88371	4.428125	High
TCGA-78-7633-01A	1528	1	67	male	i	T2	M0	N0	1.95212	8.37738	3.158822	Low
TCGA-78-8640-01A	7062	0	59	male	ii	T1	M0	N1	3.984005	11.17528	4.441464	High
TCGA-78-8648-01A	1209	1	58	female	ii	T3	M0	N0	3.104487	9.100345	3.593745	Low
TCGA-78-8655-01A	2360	0	77	female	i	T1	M0	N0	2.875399	9.283781	3.618067	Low
TCGA-78-8660-01A	321	1	69	male	ii	T2	M0	N1	4.650855	10.95979	4.478505	High
TCGA-78-8662-01A	3361	1	53	female	i	T2	M0	N0	3.453543	10.13172	4.000576	High
TCGA-83-5908-01A	824	0	59	female	i	T1	M0	N0	5.261525	8.566357	3.768779	High
TCGA-86-6562-01A	376	1	52	male	ii	T2	M0	N1	4.240446	9.774275	4.009357	High
TCGA-86-6851-01A	179	0	73	female	ii	T1	M0	N1	3.231184	9.648284	3.800192	High
TCGA-86-7701-01A	947	0	66	male	iv	T2	M1	N0	3.787948	9.94871	3.993774	High
TCGA-86-7711-01A	1046	1	70	male	ii	T2	M0	N1	5.353285	10.39083	4.401727	High
TCGA-86-7713-01A	1157	0	70	male	ii	T2	M0	N0	3.502155	9.301683	3.727527	High
TCGA-86-7714-01A	625	1	61	female	iii	T1	M0	N2	1.998503	9.143739	3.425981	Low
TCGA-86-7953-01A	997	0	69	female	i	T1	M0	N0	4.367088	9.344259	3.884636	High
TCGA-86-7954-01A	605	0	68	female	i	T2	M0	N0	3.711023	9.371889	3.785759	High
TCGA-86-7955-01A	1072	0	62	male	i	T2	M0	N0	4.653371	10.66084	4.377689	High
TCGA-86-8054-01A	1148	0	61	male	ii	T2	M0	N1	5.797469	9.68979	4.237616	High
TCGA-86-8055-01A	124	1	79	male	ii	T2	M0	N1	3.992166	9.790229	3.973799	High
TCGA-86-8056-01A	139	0	63	female	iii	T4	M0	N0	1.967955	8.207489	3.103906	Low

ID	futime	fustat	age	gender	stage	T	M	N	RRM2	GAPDH	riskScore	Risk
TCGA-86-8073-01A	740	0	58	male	i	T2	M0	N0	2.558576	7.957296	3.116621	Low
TCGA-86-8074-01A	24	0	62	female	ii	T1	M0	N1	3.956157	9.235848	3.780132	High
TCGA-86-8075-01A	694	1	66	female	i	T2	M0	N0	4.00262	9.5548	3.895802	High
TCGA-86-8076-01A	993	0	42	male	i	T1	M0	N0	1.898978	9.910662	3.66926	Low
TCGA-86-8278-01A	944	0	63	female	ii	T2	M0	N1	3.661836	9.439811	3.800644	High
TCGA-86-8279-01A	949	0	46	male	ii	T2	M0	N1	2.692811	9.527219	3.670379	Low
TCGA-86-8280-01A	701	0	54	female	ii	T2	M0	N0	2.700953	9.123959	3.535169	Low
TCGA-86-8281-01A	0	0	75	male	i	T1	M0	NX	1.357507	8.609031	3.139169	Low
TCGA-86-8358-01A	653	0	44	male	i	T2	M0	N0	6.165825	9.628557	4.27765	High
TCGA-86-8359-01A	444	1	52	male	iii	T3	M0	N2	2.563004	8.60419	3.336405	Low
TCGA-86-8585-01A	353	0	57	male	i	T2	M0	N0	2.937267	10.58495	4.06888	High
TCGA-86-8668-01A	423	0	61	female	i	T1	M0	N0	1.869134	8.820477	3.295175	Low
TCGA-86-8669-01A	938	0	64	male	i	T1	M0	N0	2.206787	9.349374	3.529975	Low
TCGA-86-8671-01A	839	0	72	female	ii	T2	M0	N1	2.755462	8.754069	3.418908	Low
TCGA-86-8672-01A	19	1	59	male	ii	T3	M0	N0	3.070149	10.23134	3.971062	High
TCGA-86-8673-01A	862	0	61	male	i	T2	M0	N0	3.491023	9.412642	3.763264	High
TCGA-86-8674-01A	806	0	50	male	ii	T2	M0	N1	2.68561	9.759209	3.747748	High
TCGA-86-A456-01A	896	0	78	female	i	T1	M0	N0	2.26515	8.661736	3.306753	Low
TCGA-86-A4D0-01A	116	1	48	male	ii	T2	M0	N0	4.211076	9.933859	4.05855	High
TCGA-86-A4JF-01A	737	1	56	male	ii	T3	M0	N0	4.767976	10.01349	4.177388	High
TCGA-86-A4P7-01A	415	0	63	female	i	T2	M0	N0	2.773147	8.525232	3.344336	Low
TCGA-86-A4P8-01A	805	0	59	female	iii	T1	MX	N2	0.96595	7.56921	2.722465	Low
TCGA-91-6828-01A	323	0	70	male	i	T1	M0	N0	2.726507	8.107621	3.195229	Low
TCGA-91-6829-01A	1258	1	78	male	i	T2	MX	N0	3.205783	9.270626	3.668117	Low
TCGA-91-6830-01A	60	0	65	female	ii	T1	MX	N1	3.042996	10.13755	3.934824	High
TCGA-91-6831-01A	310	0	66	male	i	T2	MX	N0	5.25663	9.748941	4.168421	High
TCGA-91-6835-01A	79	0	81	female	i	T1	M0	N0	3.645482	8.306224	3.414087	Low
TCGA-91-6836-01A	417	0	52	female	i	T2	MX	N0	6.067079	9.944652	4.368396	High
TCGA-91-6840-01A	372	0	59	female	i	T1	M0	N0	3.229818	8.161343	3.296454	Low
TCGA-91-6847-01A	842	0	62	female	i	T2	MX	N0	4.185577	10.22668	4.153498	High
TCGA-91-6848-01A	224	0	59	male	iii	T2	MX	N2	4.769573	9.667637	4.060538	High
TCGA-91-6849-01A	35	0	75	female	iii	T2	MX	N2	1.418304	8.944707	3.262867	Low
TCGA-91-7771-01A	492	0	62	male	ii	T3	MX	N0	2.446788	9.31893	3.55926	Low
TCGA-91-8496-01A	505	0	63	female	i	T2	MX	NX	1.496022	8.465125	3.113291	Low
TCGA-91-8497-01A	434	1	75	female	i	T1	MX	N0	1.372247	8.696969	3.171379	Low
TCGA-91-8499-01A	36	0	76	female	i	T1	MX	N0	4.164407	10.74227	4.324596	High
TCGA-91-A4BC-01A	44	0	59	male	ii	T2	MX	N0	3.376711	8.881826	3.564659	Low
TCGA-91-A4BD-01A	603	0	78	male	ii	T1	MX	N1	1.700095	9.127227	3.37116	Low
TCGA-93-7347-01A	683	0	76	female	i	T1	MX	N0	2.419822	8.876316	3.404932	Low
TCGA-93-7348-01A	531	0	75	female	i	T1	MX	N0	2.696887	8.111729	3.191734	Low

ID	futime	fustat	age	gender	stage	T	M	N	RRM2	GAPDH	riskScore	Risk
TCGA-93-8067-01A	186	0	77	male	i	T2	MX	N0	3.690342	10.62418	4.206402	High
TCGA-93-A4JN-01A	718	0	71	male	iv	T2	M1	N0	4.147491	8.640563	3.610121	Low
TCGA-93-A4JO-01A	33	1	70	male	i	T1	MX	N0	2.461613	9.717825	3.696781	Low
TCGA-93-A4JP-01A	578	0	64	male	iv	TX	M1	NX	2.552662	8.249586	3.214622	Low
TCGA-93-A4JQ-01A	526	0	49	male	i	T1	MX	N0	3.370099	9.146318	3.653131	Low
TCGA-95-7039-01A	1272	0	54	female	ii	T3	MX	N0	4.097773	10.33076	4.174257	High
TCGA-95-7043-01A	503	1	63	female	i	T1	MX	N0	4.098995	9.412131	3.863391	High
TCGA-95-7562-01A	87	1	71	male	ii	T2	M0	N1	4.507708	10.64328	4.347714	High
TCGA-95-7567-01A	568	0	61	male	ii	T2	M0	N1	3.230832	9.799856	3.851459	High
TCGA-95-7944-01A	377	0	71	male	i	T1	M0	N0	3.43754	10.22054	4.028015	High
TCGA-95-7947-01A	477	0	67	male	i	T1	M0	N0	3.333402	9.499787	3.76677	High
TCGA-95-7948-01A	476	0	42	female	i	T2	M0	N0	2.319982	8.735016	3.340614	Low
TCGA-95-8039-01A	830	0	72	male	i	T1	MX	N0	3.006557	8.957171	3.529107	Low
TCGA-95-8494-01A	84	0	67	male	ii	T2	M0	N1	4.463053	10.4018	4.258575	High
TCGA-95-A4VK-01A	651	0	74	female	iii	T2	M0	N2	2.974184	9.00837	3.541104	Low
TCGA-95-A4VN-01A	553	0	62	female	ii	T2	M0	N1	4.809307	9.636694	4.056616	High
TCGA-95-A4VP-01A	605	0	66	female	iii	T2	M0	N2	2.704862	8.863637	3.447663	Low
TCGA-97-7546-01A	1285	0	76	female	i	T1	MX	N0	1.668647	7.715989	2.888094	Low
TCGA-97-7547-01A	1965	0	67	female	i	T2	MX	N0	1.265209	8.127756	2.960972	Low
TCGA-97-7552-01A	1932	0	70	male	i	T2	MX	N0	2.561929	8.607342	3.337295	Low
TCGA-97-7553-01A	1870	0	58	female	i	T1	MX	N0	3.286083	8.573031	3.445143	Low
TCGA-97-7554-01A	775	0	83	female	iii	T2	M0	N2	2.959329	8.825398	3.476695	Low
TCGA-97-7937-01A	564	0	65	male	i	T2	MX	N0	2.299588	9.314672	3.533534	Low
TCGA-97-7938-01A	18	1	76	female	i	T1	MX	N0	1.600643	6.891001	2.597516	Low
TCGA-97-7941-01A	484	0	72	female	i	T1	MX	N0	1.824925	9.211881	3.42042	Low
TCGA-97-8171-01A	568	0	81	male	iv	T2	M1	N2	2.887625	9.612081	3.731254	High
TCGA-97-8172-01A	545	0	75	female	i	T2	M0	N0	1.294422	8.376879	3.05015	Low
TCGA-97-8174-01A	164	1	67	male	ii	T2	M0	N0	1.2154	8.234305	2.988834	Low
TCGA-97-8175-01A	551	0	55	female	i	T2	M0	N0	3.398126	10.41501	4.087365	High
TCGA-97-8176-01A	468	1	63	male	iii	T3	M0	N1	4.231399	10.47796	4.24615	High
TCGA-97-8177-01A	499	0	59	female	i	T2	M0	N0	2.713055	9.773775	3.757208	High
TCGA-97-8179-01A	435	0	72	male	i	T1	M0	N0	2.559335	8.915338	3.441162	Low
TCGA-97-8547-01A	657	0	78	female	iii	T2	MX	N2	2.993675	9.921731	3.853604	High
TCGA-97-8552-01A	626	0	55	female	i	T1	MX	N0	1.279892	8.149829	2.970868	Low
TCGA-97-A4LX-01A	614	0	81	male	i	T2	M0	N0	3.330821	9.277267	3.690994	Low
TCGA-97-A4M0-01A	652	0	60	female	i	T2	M0	N0	1.72147	9.134384	3.37711	Low
TCGA-97-A4M1-01A	601	0	52	female	i	T1	M0	N0	1.141568	7.640744	2.775661	Low
TCGA-97-A4M2-01A	624	0	66	male	i	T1	M0	N0	1.254365	8.163324	2.971227	Low
TCGA-97-A4M3-01A	540	0	69	female	i	T1	M0	N0	1.834548	9.136762	3.39657	Low
TCGA-97-A4M5-01A	634	0	83	male	i	T1	M0	N0	2.002675	8.666919	3.265207	Low

ID	futime	fustat	age	gender	stage	T	M	N	RRM2	GAPDH	riskScore	Risk
TCGA-97-A4M6-01A	568	0	45	female	i	T1	M0	N0	2.658364	8.505794	3.318818	Low
TCGA-97-A4M7-01A	629	0	74	male	i	T1	M0	N0	2.412669	8.95382	3.429996	Low
TCGA-99-7458-01A	747	0	74	female	iii	T4	M0	N0	2.374619	9.144388	3.48825	Low
TCGA-99-8025-01A	1060	0	72	female	iii	T3	M0	N2	2.829489	8.974239	3.505675	Low
TCGA-99-8028-01A	1118	0	50	female	i	T1	M0	N0	2.638256	9.493606	3.649996	Low
TCGA-99-8032-01A	44	0	61	male	i	T1	M0	N0	2.87459	9.613268	3.729506	High
TCGA-99-8033-01A	656	1	74	female	iv	TX	M1	NX	3.863486	10.74173	4.27477	High
TCGA-99-AA5R-01A	658	0	70	female	i	T1	M0	N0	1.914159	8.101906	3.059278	Low
TCGA-J2-8192-01A	739	0	65	female	ii	T2	MX	N1	2.3294	8.904776	3.399652	Low
TCGA-J2-8194-01A	724	0	69	female	ii	T3	MX	N0	1.545451	9.428533	3.447677	Low
TCGA-J2-A4AD-01A	550	1	61	female	i	T1	MX	N0	3.633575	9.894987	3.950115	High
TCGA-J2-A4AE-01A	1079	0	77	female	i	T1	MX	N0	2.57772	8.087187	3.163764	Low
TCGA-J2-A4AG-01A	988	0	66	female	i	T1	MX	N0	2.139153	8.471338	3.221494	Low
TCGA-L4-A4E5-01A	578	0	48	female	i	T1	M0	N0	4.371322	10.01094	4.111089	High
TCGA-L4-A4E6-01A	435	0	67	male	i	T1	M0	N0	1.419039	7.983988	2.937666	Low
TCGA-L9-A443-01A	193	1	63	female	i	T1	MX	N0	2.581589	9.194513	3.539368	Low
TCGA-L9-A444-01A	307	0	60	female	i	T1	MX	N0	2.976687	8.985533	3.533783	Low
TCGA-L9-A50W-01A	442	1	75	male	ii	T1	MX	N1	1.782565	9.008194	3.344458	Low
TCGA-L9-A5IP-01A	58	1	40	female	iv	T3	M1	N2	4.408481	9.795951	4.044418	High
TCGA-L9-A743-01A	664	0	56	male	ii	T2	M0	N1	3.380054	8.898562	3.570878	Low
TCGA-L9-A7SV-01A	565	0	69	male	ii	T2	M0	N1	3.093283	7.389552	3.012583	Low
TCGA-L9-A8F4-01A	476	0	64	female	i	T2	MX	N0	3.679593	9.141755	3.702645	Low
TCGA-MN-A4N1-01A	827	0	60	male	ii	T2	M0	N1	4.422068	10.22514	4.191992	High
TCGA-MN-A4N4-01A	1175	0	57	male	i	T1	M0	N0	1.739345	10.00653	3.675387	Low
TCGA-MN-A4N5-01A	84	0	63	male	i	T1	M0	N0	4.039814	9.066678	3.736649	High
TCGA-MP-A4SV-01A	2620	1	67	male	i	T2	M0	N0	3.666504	9.684278	3.884196	High
TCGA-MP-A4SW-01A	1778	1	53	male	ii	T2	M0	N1	3.126145	9.521619	3.739971	High
TCGA-MP-A4SY-01A	1501	1	61	male	ii	T2	M0	N1	3.429564	10.40914	4.090561	High
TCGA-MP-A4T4-01A	2617	1	68	female	ii	T2	M0	N1	3.871333	9.139666	3.733569	High
TCGA-MP-A4T6-01A	1790	1	76	female	iii	T1	MX	N2	1.673001	8.880717	3.283216	Low
TCGA-MP-A4T7-01A	167	1	75	female	iv	T2	M1	N0	3.450122	10.11964	3.995922	High
TCGA-MP-A4T8-01A	161	1	68	male	iii	T2	M0	N2	3.339025	9.888724	3.899401	High
TCGA-MP-A4T9-01A	1265	1	54	female	iii	T2	MX	N2	3.942044	8.787725	3.626059	Low
TCGA-MP-A4TA-01A	950	1	75	female	i	T1	M0	N0	4.120932	9.207178	3.797608	High
TCGA-MP-A4TC-01A	74	1	77	male	iii	T1	M0	N2	3.560283	9.428335	3.780004	High
TCGA-MP-A4TD-01A	307	1	71	male	iii	T2	M0	N2	2.193046	9.275233	3.502602	Low
TCGA-MP-A4TE-01A	896	1	56	male	ii	T2	MX	N0	2.861152	10.4445	4.008762	High
TCGA-MP-A4TF-01A	336	1	58	female	ii	T2	M0	N0	4.303156	10.72242	4.340765	High
TCGA-MP-A4TH-01A	741	0	70	female	i	T1	M0	N0	1.413651	7.997094	2.941216	Low
TCGA-MP-A4TI-01A	429	1	72	male	ii	T2	M0	N1	3.804212	10.62631	4.225907	High

ID	futime	fustat	age	gender	stage	T	M	N	RRM2	GAPDH	riskScore	Risk
TCGA-MP-A4TJ-01A	339	1	62	female	i	T1	M0	N0	3.386002	8.923451	3.580287	Low
TCGA-MP-A4TK-01A	582	1	56	female	ii	T2	MX	N1	3.88386	9.210656	3.759675	High
TCGA-MP-A5C7-01A	2248	0	76	female	i	T2	M0	N0	1.326084	8.297983	3.028657	Low
TCGA-NJ-A4YF-01A	2161	0	50	female	i	T1	M0	N0	2.824948	9.115922	3.552903	Low
TCGA-NJ-A4YG-01A	2261	0	65	male	i	T2	M0	N0	1.898885	8.86729	3.315935	Low
TCGA-NJ-A4YI-01A	4	1	87	female	iii	T2	M0	N2	2.09424	8.553164	3.241793	Low
TCGA-NJ-A4YP-01A	50	0	52	male	i	T2	M0	N0	2.97884	9.494317	3.706425	Low
TCGA-NJ-A4YQ-01A	1432	0	69	female	i	T1	M0	N0	3.903737	9.670758	3.918755	High
TCGA-NJ-A55A-01A	15	0	76	female	i	T2	M0	N0	2.008524	7.804915	2.974278	Low
TCGA-NJ-A55O-01A	13	0	56	female	ii	T1	M0	N1	3.079817	9.51307	3.729433	High
TCGA-NJ-A55R-01A	603	0	67	male	i	T1	MX	N0	2.852981	9.737081	3.767867	High
TCGA-NJ-A7XG-01A	617	0	49	male	iii	T4	M0	N1	1.261012	8.984201	3.250291	Low
TCGA-O1-A52J-01A	1798	1	74	female	i	T1	MX	N0	2.115893	8.095636	3.090435	Low
TCGA-S2-AA1A-01A	513	0	68	female	i	T1	M0	N0	1.690707	8.932979	3.303835	Low
