# Supplementary material for: DNA damage repair-related methylated genes RRM2 and GAPDH are prognostic biomarkers associated with immunotherapy for lung adenocarcinoma
Source: Genet Mol Biol. 2025 May 9;48(2):e20240138. doi: 10.1590/1678-4685-GMB-2024-0138 (PMC12063672; doi:10.1590/1678-4685-GMB-2024-0138)
Supplement: Table S11 - [file 1415-4757-GMB-48-02-e20240138-s12.pdf]

Genetics and Molecular Biology
https://doi.org/10.1590/1678-4685-GMB-2024-0138

Supplementary Material to "DNA damage repair-related methylated genes RRM2 and GAPDH are prognostic biomarkers associated with immunotherapy for lung adenocarcinoma"

Table S11 - Information on survival time, survival status, risk score, and grouping of LUAD patients in the GSE31210 cohort.

ID	futime	fustat	RRM2	GAPDH	riskScore	risk
GSM773540	437	1	2183.513	19815.83	7070.318	High Risk
GSM773541	743	0	2552.547	21406.63	7669.881	High Risk
GSM773542	2601	0	2382.527	24163.53	8575.384	High Risk
GSM773543	1173	0	3425.058	17756.18	6577.695	High Risk
GSM773544	2369	0	638.7321	19496.25	6707.254	High Risk
GSM773545	1879	0	1383.579	15227.13	5384.512	High Risk
GSM773546	606	0	3418.775	18344.93	6776.022	High Risk
GSM773547	300	1	891.9826	19022.66	6588.663	High Risk
GSM773548	1919	0	1616.804	14749.36	5261.204	High Risk
GSM773549	1835	0	888.5214	16047.34	5580.581	High Risk
GSM773550	1828	0	937.9267	14952.07	5217.848	High Risk
GSM773551	1841	1	911.9627	12325.94	4324.296	High Risk
GSM773552	639	1	11276.44	25578.05	10521.63	High Risk
GSM773553	1311	1	3236.748	14200.8	5342.696	High Risk
GSM773554	1098	0	1894.934	11641.53	4254.704	High Risk
GSM773555	1189	0	8761.688	17248.59	7286.217	High Risk
GSM773558	1856	0	7172.269	24561.27	9500.247	High Risk
GSM773559	3067	0	11583.34	30992.86	12405.84	High Risk
GSM773560	1142	0	8144.796	18754.72	7694.458	High Risk
GSM773562	1610	0	12503.62	12207.13	6196.384	High Risk
GSM773563	2014	0	9248.728	15185.04	6667.802	High Risk
GSM773564	1826	0	1089.26	13504.5	4752.634	High Risk
GSM773565	2191	0	6581.664	19734.54	7768.372	High Risk
GSM773566	3342	0	1845.921	13196.27	4773.09	High Risk
GSM773568	2925	0	2648.691	17345.07	6310.406	High Risk
GSM773570	1724	0	1621.376	11799.73	4263.145	High Risk

ID	futime	fustat	RRM2	GAPDH	riskScore	risk
GSM773571	2765	0	1175.682	17495.76	6118.425	High Risk
GSM773572	1529	0	5038.598	15090.49	5941.223	High Risk
GSM773573	221	1	6702.686	28348.08	10705.08	High Risk
GSM773574	546	1	4233.117	14374.62	5565.931	High Risk
GSM773575	346	1	2624.56	18927.15	6842.154	High Risk
GSM773576	853	1	800.3239	12807.43	4468.923	High Risk
GSM773578	817	0	2825.411	17211.5	6294.327	High Risk
GSM773579	425	1	2908.168	13935.79	5198.751	High Risk
GSM773580	1281	0	1553.186	13122.92	4699.958	High Risk
GSM773581	1044	0	5679.134	21695.32	8283.442	High Risk
GSM773582	565	0	7694.678	23275.05	9150.887	High Risk
GSM773583	1059	1	4457.677	18070.2	6854.385	High Risk
GSM773584	1625	1	2948.8	16588.83	6103.836	High Risk
GSM773585	1695	1	5084.338	21662.03	8174.046	High Risk
GSM773586	2976	0	8811.655	20752.45	8480.948	High Risk
GSM773587	1143	1	3535.337	11236.27	4388.096	High Risk
GSM773588	1682	0	1392.308	13913.84	4941.241	High Risk
GSM773589	1242	1	2910.242	19218.62	6987.981	High Risk
GSM773590	2252	0	2385.476	13960.71	5120.957	High Risk
GSM773591	943	1	5466.545	19208.06	7406.129	High Risk
GSM773592	1346	1	3597.136	15481.07	5835.68	High Risk
GSM773593	1482	1	4010.646	14093.26	5433.953	High Risk
GSM773597	259	1	1597.853	12183.23	4389.125	High Risk
GSM773598	2289	0	1878.804	21367.45	7545.465	High Risk
GSM773599	1916	0	3498.291	14798.63	5588.284	High Risk
GSM773600	2048	0	4509.907	12854.3	5096.779	High Risk
GSM773601	1475	0	8419.987	23304.97	9280.676	High Risk
GSM773603	636	0	7306.324	18965.44	7627.486	High Risk
GSM773604	1275	0	7262.816	19136.42	7678.205	High Risk
GSM773610	729	0	1077.095	12149.94	4291.942	High Risk
GSM773613	2009	0	11681.46	13660.48	6552.886	High Risk
GSM773615	3411	0	979.8915	14674.61	5130.816	High Risk
GSM773616	1806	0	14186.97	24122.19	10508.81	High Risk
GSM773617	1937	0	1170.806	14837.49	5217.467	High Risk
GSM773621	1597	0	6140.201	17595.08	6971.072	High Risk
GSM773627	2050	0	1733.335	13575.95	4883.083	High Risk
GSM773628	1256	0	5888.029	10355.5	4477.981	High Risk
GSM773630	1154	0	16318.91	14743.47	7684.669	High Risk
GSM773633	1596	0	5280.411	10577.5	4452.912	High Risk

ID	futime	fustat	RRM2	GAPDH	riskScore	risk
GSM773636	1637	0	1476.256	17272.49	6092.407	High Risk
GSM773637	2034	0	1443.681	11927.11	4276.965	High Risk
GSM773639	779	1	4155.286	18984.63	7114.145	High Risk
GSM773640	540	1	1337.57	17379.15	6105.646	High Risk
GSM773641	1317	0	1693.081	14983.7	5353.138	High Risk
GSM773642	1980	0	1625.432	16701.13	5923.541	High Risk
GSM773643	702	1	2647.885	14930.62	5492.684	High Risk
GSM773644	564	1	1467.637	13047.51	4660.31	High Risk
GSM773645	788	1	2735.576	16510.17	6042.022	High Risk
GSM773646	1463	0	1852.758	14949.39	5367.865	High Risk
GSM773647	1345	1	1328.729	12300.2	4384.336	High Risk
GSM773649	2687	0	5469.236	19355.08	7456.357	High Risk
GSM773651	2668	0	5758.982	22956.8	8723.78	High Risk
GSM773652	1936	0	1192.122	16962	5940.392	High Risk
GSM773654	2671	0	2305.678	12968.12	4771.679	High Risk
GSM773655	1482	0	1007.621	11888.46	4191.936	High Risk
GSM773658	1229	1	10178.97	23686.17	9699.946	High Risk
GSM773659	2263	1	5929.145	21707.05	8328.662	High Risk
GSM773660	1770	0	2458.313	17377.77	6290.072	High Risk
GSM773661	1433	1	3595.089	19873.34	7322.667	High Risk
GSM773663	1988	0	743.013	14866.97	5156.876	High Risk
GSM773665	630	1	3961.766	19446.18	7238.513	High Risk
GSM773666	1537	0	3532.829	14328.63	5434.828	High Risk
GSM773667	1429	1	2981.175	13085.61	4922.903	High Risk
GSM773669	2486	0	1523.482	14850.31	5279.993	High Risk
GSM773673	1017	0	4531.141	14690.92	5722.204	High Risk
GSM773674	1041	0	7053.833	18379.04	7387.263	High Risk
GSM773675	1859	0	2026.61	13076.17	4762.23	High Risk
GSM773676	1717	0	2938.351	15259.61	5652.007	High Risk
GSM773677	1494	0	2131.179	15677.43	5660.328	High Risk
GSM773679	2364	0	1505.272	15324.18	5437.45	High Risk
GSM773680	1733	0	1028.987	13702.17	4809.625	High Risk
GSM773683	2913	0	5628.561	17056.83	6704.399	High Risk
GSM773688	1964	0	1415.796	12173.98	4355.959	High Risk
GSM773697	1998	0	5987.94	11968.9	5040.796	High Risk
GSM773702	3189	0	1705.581	12662.16	4569.074	High Risk
GSM773708	1412	0	734.6344	12322.61	4293.913	High Risk
GSM773713	1426	0	2307.207	11840.91	4390.233	High Risk
GSM773714	1674	0	1751.899	15492.82	5535.242	High Risk

ID	futime	fustat	RRM2	GAPDH	riskScore	risk
GSM773716	1856	0	4617.449	15990.66	6176.564	High Risk
GSM773720	1114	0	770.9567	13412.82	4669.079	High Risk
GSM773723	1726	0	1867.19	13236.47	4790.21	High Risk
GSM773724	1629	0	1934.824	12907.56	4689.991	High Risk
GSM773727	1446	0	2008.242	13300.9	4835.299	High Risk
GSM773746	1947	0	2488.329	13212.97	4884.727	High Risk
GSM773749	2409	0	1924.021	11341	4157.737	High Risk
GSM773751	2430	0	2584.794	15410.05	5644.62	High Risk
GSM773756	1849	0	2543.053	13594.66	5023	High Risk
GSM773556	3863	0	2278.36	9695.859	3659.111	Low Risk
GSM773557	1475	0	2597.018	9741.436	3727.114	Low Risk
GSM773561	1825	0	662.3989	7550.712	2666.123	Low Risk
GSM773567	1328	0	1402.841	10531.07	3797.495	Low Risk
GSM773569	2880	0	1760.214	8768.159	3259.49	Low Risk
GSM773577	2661	1	2240.158	6467.617	2559.651	Low Risk
GSM773594	2386	0	378.1273	8553.192	2958.689	Low Risk
GSM773595	1560	0	691.7334	8493.741	2990.294	Low Risk
GSM773596	1001	1	428.345	8199.847	2847.323	Low Risk
GSM773602	1539	0	457.301	9260.665	3211.317	Low Risk
GSM773605	2005	0	1899.746	11240.73	4119.779	Low Risk
GSM773606	1965	0	286.3409	8848.169	3043.433	Low Risk
GSM773607	2425	0	606.755	7230.334	2548.456	Low Risk
GSM773608	1566	0	340.4119	8027.501	2774.456	Low Risk
GSM773609	990	0	538.6639	7750.908	2713.501	Low Risk
GSM773611	2354	0	601.7399	9069.609	3170.45	Low Risk
GSM773612	818	0	3235.666	9797.54	3851.473	Low Risk
GSM773614	2549	0	664.6868	10637.46	3711.745	Low Risk
GSM773618	1520	0	885.6568	8928.845	3169.623	Low Risk
GSM773619	1375	0	1051.194	11536.53	4079.954	Low Risk
GSM773620	1756	0	416.6226	7237.145	2519.396	Low Risk
GSM773622	1714	0	256.6318	7996.502	2750.137	Low Risk
GSM773623	1637	0	514.0172	5277.043	1871.728	Low Risk
GSM773624	1653	0	751.0739	7955.447	2817.805	Low Risk
GSM773625	1196	0	661.8991	8870.019	3112.789	Low Risk
GSM773626	1702	0	1331.233	7962.764	2915.994	Low Risk
GSM773629	818	0	509.5877	8778.146	3056.551	Low Risk
GSM773631	3058	0	1425.952	8571.931	3137.898	Low Risk
GSM773632	1941	0	2073.937	7964.348	3039.057	Low Risk
GSM773634	2450	0	942.5596	8377.001	2992.143	Low Risk

ID	futime	fustat	RRM2	GAPDH	riskScore	risk
GSM773635	1881	0	937.1546	8768.98	3123.985	Low Risk
GSM773638	2134	0	221.0073	6018.91	2074.602	Low Risk
GSM773648	832	0	1698.139	9221.185	3402.654	Low Risk
GSM773650	1122	1	783.4527	11277.24	3947.984	Low Risk
GSM773653	871	0	1373.466	8321.28	3044.363	Low Risk
GSM773656	1128	0	1127.871	10847.32	3859.22	Low Risk
GSM773657	1042	1	923.4096	9686.949	3432.563	Low Risk
GSM773662	1823	0	568.7181	11811.1	4093.334	Low Risk
GSM773664	1434	0	820.347	5428.987	1973.716	Low Risk
GSM773668	1038	1	1050.54	6604.684	2409.81	Low Risk
GSM773670	740	0	875.2963	10029.42	3540.594	Low Risk
GSM773671	1042	0	901.4685	6391.72	2313.102	Low Risk
GSM773672	1267	0	1644.96	10277.57	3751.596	Low Risk
GSM773678	754	0	1337.587	10977.96	3938.056	Low Risk
GSM773681	1941	0	233.457	6032.149	2081.139	Low Risk
GSM773682	774	0	832.9404	11353.22	3981.875	Low Risk
GSM773684	1803	0	743.507	8977.392	3162.611	Low Risk
GSM773685	1912	0	623.6043	7647.86	2692.62	Low Risk
GSM773686	1481	0	619.5579	6109.621	2171.069	Low Risk
GSM773687	1231	0	686.2375	9153.297	3212.729	Low Risk
GSM773689	3352	0	520.4593	7943.85	2775.833	Low Risk
GSM773690	2158	0	1009.86	11322.71	4000.732	Low Risk
GSM773691	3466	0	761.1533	7004.107	2497.322	Low Risk
GSM773692	3263	0	699.737	7607.816	2691.62	Low Risk
GSM773693	1823	0	640.2124	7461.559	2632.274	Low Risk
GSM773694	1856	0	2636.881	10408.3	3959.507	Low Risk
GSM773695	2449	0	723.7404	9870.694	3461.843	Low Risk
GSM773696	1859	0	299.7207	4568.786	1596.542	Low Risk
GSM773698	1852	0	1011.534	9354.112	3334.394	Low Risk
GSM773699	1894	0	669.7923	5125.254	1846.027	Low Risk
GSM773700	1861	0	1077.376	8384.213	3016.827	Low Risk
GSM773701	1669	0	1022.523	8355.91	2998.193	Low Risk
GSM773703	1822	0	2896.931	8212.222	3258.765	Low Risk
GSM773704	1809	0	728.2268	5388.08	1944.666	Low Risk
GSM773705	2552	0	1009.549	8809.174	3149.538	Low Risk
GSM773706	2968	0	268.6807	6068.238	2099.171	Low Risk
GSM773707	3066	0	812.7386	11680.72	4089.44	Low Risk
GSM773709	1317	0	1137.309	5844.244	2166.622	Low Risk
GSM773710	1180	0	610.9787	6255.416	2219.024	Low Risk

ID	futime	fustat	RRM2	GAPDH	riskScore	risk
GSM773711	1688	0	452.2593	8322.686	2892.864	Low Risk
GSM773712	1417	0	488.5992	3660.649	1320.186	Low Risk
GSM773715	1848	0	1546.068	10805.82	3914.159	Low Risk
GSM773717	1780	0	2457.809	8289.456	3212.475	Low Risk
GSM773718	2084	0	580.8934	7637.865	2682.189	Low Risk
GSM773719	1879	0	422.2543	5833.199	2044.917	Low Risk
GSM773721	2806	0	616.5497	7902.774	2777.776	Low Risk
GSM773722	1663	0	877.6795	10484.42	3695.06	Low Risk
GSM773725	2179	0	1279.155	10343.73	3713.651	Low Risk
GSM773726	1917	0	821.1773	11342.62	3976.345	Low Risk
GSM773728	1162	0	698.5386	7798.798	2756.093	Low Risk
GSM773729	1231	0	1255.164	9032.086	3265.541	Low Risk
GSM773730	941	0	1236.777	11154.79	3981.305	Low Risk
GSM773731	1623	0	862.2058	6710.05	2414.419	Low Risk
GSM773732	1855	0	535.0821	4055.68	1461.621	Low Risk
GSM773733	1404	0	919.2522	6783.489	2448.698	Low Risk
GSM773734	2221	0	522.2342	4400.162	1576.151	Low Risk
GSM773735	849	0	568.113	6712.17	2366.619	Low Risk
GSM773736	831	0	823.2625	7505.094	2677.214	Low Risk
GSM773737	3219	0	2121.102	6391.263	2514.155	Low Risk
GSM773738	2585	0	1155.67	4430.864	1691.048	Low Risk
GSM773739	2442	0	355.5437	8284.165	2863.864	Low Risk
GSM773740	1910	0	2307.99	9369.332	3553.429	Low Risk
GSM773741	1834	0	639.57	9266.977	3243.524	Low Risk
GSM773742	2918	0	2545.503	6616.29	2660.369	Low Risk
GSM773743	2285	0	967.1256	7134.338	2575.401	Low Risk
GSM773744	2149	0	381.4822	5296.857	1856.572	Low Risk
GSM773745	2561	0	3175.365	8715.413	3475.091	Low Risk
GSM773747	1841	0	470.0926	10695.6	3699.329	Low Risk
GSM773748	1971	0	674.7801	5665.924	2029.933	Low Risk
GSM773750	1624	0	535.5624	6432.619	2266.587	Low Risk
GSM773752	1856	0	452.1499	5670.711	1994.826	Low Risk
GSM773753	1516	0	1084.858	8232.772	2966.779	Low Risk
GSM773754	1819	0	1134.03	10946.75	3893.908	Low Risk
GSM773755	2413	0	548.4941	7928.772	2775.352	Low Risk
GSM773757	2181	0	662.9781	9962.128	3482.78	Low Risk
GSM773758	2168	0	395.0383	8971.808	3103.232	Low Risk
GSM773759	2154	0	487.217	5850.392	2061.456	Low Risk
GSM773760	2073	0	584.1912	8856.486	3095.386	Low Risk

ID	futime	fustat	RRM2	GAPDH	riskScore	risk
GSM773761	1994	0	743.02	8168.766	2888.711	Low Risk
GSM773762	2137	0	577.4151	7527.626	2644.286	Low Risk
GSM773763	1998	0	593.3719	8121.011	2847.852	Low Risk
GSM773764	1908	0	1714.741	10489.92	3835.016	Low Risk
GSM773765	1660	0	532.8082	7308.202	2562.625	Low Risk
