# Supplementary material for: DNA damage repair-related methylated genes RRM2 and GAPDH are prognostic biomarkers associated with immunotherapy for lung adenocarcinoma
Source: Genet Mol Biol. 2025 May 9;48(2):e20240138. doi: 10.1590/1678-4685-GMB-2024-0138 (PMC12063672; doi:10.1590/1678-4685-GMB-2024-0138)
Supplement: Table S12 - [file 1415-4757-GMB-48-02-e20240138-s13.pdf]

Genetics and Molecular Biology
https://doi.org/10.1590/1678-4685-GMB-2024-0138

Supplementary Material to "DNA damage repair-related methylated genes RRM2 and GAPDH are prognostic biomarkers associated with immunotherapy for lung adenocarcinoma"

Table S12 - Information on survival time, survival status, risk score, and grouping of LUAD patients in the GSE68465 cohort.

id	OS.time	OS	RRM2	GAPDH	riskScore	risk
GSM1672281	3168	0	7.84785288	14.1997797	6.10306148	Low Risk
GSM1672282	756	0	8.37672883	13.8724825	6.07948169	Low Risk
GSM1672283	1944	1	7.1304573	14.3721441	6.04307669	Low Risk
GSM1672284	2076	0	6.19086174	13.8615522	5.71517003	Low Risk
GSM1672285	1017	1	7.72899527	14.1104017	6.05318768	Low Risk
GSM1672286	2490	0	6.81694524	13.9701868	5.85524346	Low Risk
GSM1672287	2076	0	9.06284756	13.6610655	6.12108251	Low Risk
GSM1672288	813	0	7.94501067	13.9219804	6.0250207	Low Risk
GSM1672289	174	1	8.30942633	14.123305	6.15331285	Low Risk
GSM1672290	1506	1	7.79765504	13.9618385	6.0142078	Low Risk
GSM1672291	2190	0	8.65124547	13.7085235	6.0692494	Low Risk
GSM1672292	1044	1	8.02734207	14.3780781	6.1930484	Low Risk
GSM1672293	828	1	9.40483289	14.3839068	6.42227178	High Risk
GSM1672294	1464	1	8.97178207	14.3420677	6.33666216	Low Risk
GSM1672295	1998	0	10.896075	14.5834354	6.7358524	High Risk
GSM1672296	303	1	11.5496355	14.9259114	6.9596429	High Risk
GSM1672297	261	1	10.0314255	14.5517864	6.58249094	High Risk
GSM1672298	1836	1	8.86459165	14.3686906	6.32799369	Low Risk
GSM1672299	3966	0	10.2921606	14.4014729	6.57460574	High Risk
GSM1672300	3864	1	6.80689112	14.2203557	5.93829774	Low Risk
GSM1672301	1314	1	7.08445763	13.3901421	5.70295944	Low Risk
GSM1672302	177	1	9.97262086	14.7412563	6.63694862	High Risk
GSM1672303	72	1	11.9669992	14.9119537	7.02377051	High Risk
GSM1672304	528	1	11.3272776	14.4370823	6.75743092	High Risk
GSM1672305	489	1	9.74461859	14.7991399	6.61893499	High Risk
GSM1672306	888	1	8.81274849	14.1079281	6.23114074	Low Risk

id	OS.time	OS	RRM2	GAPDH	riskScore	risk
GSM1672307	2436	1	10.3458266	14.671519	6.67490304	High Risk
GSM1672308	1770	1	8.15825623	13.8355274	6.03092562	Low Risk
GSM1672309	2778	1	9.59558758	14.6252979	6.53548188	High Risk
GSM1672310	120	1	10.7471608	14.8232727	6.79249992	High Risk
GSM1672311	504	1	9.88728765	14.470303	6.53111985	High Risk
GSM1672312	1860	0	9.52617447	14.6433489	6.53014304	High Risk
GSM1672313	1869	1	9.97889632	13.9014703	6.35361301	Low Risk
GSM1672314	2154	0	10.1586602	14.1878546	6.48024564	High Risk
GSM1672315	2520	0	11.5535765	14.5366115	6.82846715	High Risk
GSM1672316	558	1	7.28581832	14.2485428	6.02685293	Low Risk
GSM1672317	1374	1	11.2592666	14.908022	6.90568192	High Risk
GSM1672318	618	1	10.1825682	14.5026392	6.59078312	High Risk
GSM1672319	3315	0	10.1113313	14.4797929	6.57129465	High Risk
GSM1672320	4008	0	8.73886239	14.198775	6.24971433	Low Risk
GSM1672321	2115	1	7.45950539	13.8811522	5.93109978	Low Risk
GSM1672322	1563	0	7.5598818	13.3184863	5.7571276	Low Risk
GSM1672323	528	1	10.0282238	14.1301522	6.43918771	High Risk
GSM1672324	507	1	10.2693137	14.0638922	6.45652401	High Risk
GSM1672325	2604	0	7.60536839	14.0877694	6.02512868	Low Risk
GSM1672326	3102	0	8.29926298	13.5912769	5.97147916	Low Risk
GSM1672327	3528	0	10.6114491	14.2943479	6.59100494	High Risk
GSM1672328	3474	1	9.2891705	14.4614794	6.42945844	High Risk
GSM1672329	570	1	8.75847641	13.4830474	6.01058826	Low Risk
GSM1672330	2523	1	8.85499925	13.9806305	6.1950051	Low Risk
GSM1672331	4599	0	9.27656645	13.8971931	6.23629876	Low Risk
GSM1672332	4617	0	10.1965161	13.9485954	6.40547216	Low Risk
GSM1672333	4878	0	6.10836345	13.2539435	5.49580966	Low Risk
GSM1672334	1914	0	8.62413015	14.3447536	6.28021827	Low Risk
GSM1672335	3975	1	9.18202432	14.1133189	6.29388697	Low Risk
GSM1672336	3924	0	10.1433194	14.4560399	6.56852854	High Risk
GSM1672337	4788	0	8.49964661	14.5670173	6.33494536	Low Risk
GSM1672338	4731	0	9.98928095	14.2850192	6.48520471	High Risk
GSM1672339	2289	0	10.1257751	14.3728583	6.53746695	High Risk
GSM1672340	3177	0	10.3392042	14.1570232	6.4995904	High Risk
GSM1672341	846	1	10.3425636	14.5928424	6.64772306	High Risk
GSM1672342	216	1	10.0527311	14.9525593	6.72171674	High Risk
GSM1672343	2385	1	9.25551962	14.7951163	6.53688407	High Risk
GSM1672344	2469	0	7.84274004	13.8685332	5.9900503	Low Risk

id	OS.time	OS	RRM2	GAPDH	riskScore	risk
GSM1672345	3036	0	8.48392055	14.4326006	6.2868344	Low Risk
GSM1672346	5043	0	11.1772077	14.2170944	6.65818036	High Risk
GSM1672347	447	1	10.7525563	14.3313858	6.6268258	High Risk
GSM1672348	288	1	9.52832126	14.0533999	6.33072686	Low Risk
GSM1672349	2850	0	10.0395497	14.4973342	6.56539246	High Risk
GSM1672350	1872	0	10.1597578	14.3401631	6.53200186	High Risk
GSM1672351	1929	0	10.2662829	13.6569834	6.31823532	Low Risk
GSM1672352	627	1	8.02785625	13.9442637	6.04623367	Low Risk
GSM1672353	669	1	9.12993723	14.5455799	6.43166751	High Risk
GSM1672354	2037	1	7.23680829	14.5024403	6.10474308	Low Risk
GSM1672355	1089	0	10.1900345	14.652733	6.64284009	High Risk
GSM1672356	681	1	10.3233025	14.1938025	6.50942138	High Risk
GSM1672357	987	0	10.3977388	14.245367	6.53916232	High Risk
GSM1672358	1143	0	9.33903937	13.8821654	6.24151639	Low Risk
GSM1672359	1626	1	7.60817349	13.8682052	5.95124194	Low Risk
GSM1672360	2883	1	8.5427301	13.6685637	6.03781592	Low Risk
GSM1672361	1596	0	8.42269975	13.598006	5.99412159	Low Risk
GSM1672362	375	1	9.91147888	14.3150929	6.48255306	High Risk
GSM1672363	885	0	7.93061908	13.2271647	5.78736585	Low Risk
GSM1672364	3225	0	10.5384994	14.219108	6.5534922	High Risk
GSM1672365	1611	0	9.27767796	14.4457133	6.42222368	High Risk
GSM1672366	654	0	8.93707657	14.4430666	6.3651372	Low Risk
GSM1672367	1674	0	8.86579821	14.3122945	6.30909571	Low Risk
GSM1672368	630	1	8.69371345	14.5042045	6.34569142	Low Risk
GSM1672369	219	1	7.39768319	14.4782135	6.12307945	Low Risk
GSM1672370	4797	0	10.4260236	14.322407	6.56991609	High Risk
GSM1672371	1824	0	7.79427939	14.133672	6.07183769	Low Risk
GSM1672372	636	1	7.95129637	13.6742585	5.94217333	Low Risk
GSM1672373	1608	0	6.74325913	13.630506	5.72806346	Low Risk
GSM1672374	696	0	10.1346445	13.8746452	6.37022375	Low Risk
GSM1672375	1418.1	0	9.04075273	14.3643194	6.35557542	Low Risk
GSM1672376	1773.3	1	9.39950652	14.6208441	6.50162552	High Risk
GSM1672377	1547.4	0	9.23404619	14.5604642	6.4538829	High Risk
GSM1672378	1087.5	1	8.76325553	14.2709377	6.27817451	Low Risk
GSM1672379	198	1	8.3399346	14.2354309	6.1963143	Low Risk
GSM1672380	1647.9	1	11.1920092	14.3990509	6.72223689	High Risk
GSM1672381	292.2	1	8.24062917	13.9031758	6.06742228	Low Risk
GSM1672382	1766.4	0	7.65943939	13.6029192	5.8698675	Low Risk

id	OS.time	OS	RRM2	GAPDH	riskScore	risk
GSM1672383	1554.3	0	10.066291	14.3227802	6.51069608	High Risk
GSM1672384	644.4	1	8.01915146	13.7155974	5.96736595	Low Risk
GSM1672385	191.4	1	10.7555135	14.0867665	6.54447993	High Risk
GSM1672386	2070.3	1	8.35108057	14.1539478	6.17056104	Low Risk
GSM1672387	1924.2	0	9.73161263	13.8043428	6.27992811	Low Risk
GSM1672388	967.2	1	9.80943052	14.1283891	6.40249556	Low Risk
GSM1672390	1690.5	0	10.5063869	14.5829064	6.67138502	High Risk
GSM1672391	2071.5	0	8.19539682	14.1829529	6.15469914	Low Risk
GSM1672392	1689.6	0	9.69069819	14.3733818	6.46586802	High Risk
GSM1672393	1837.5	0	9.893388	14.8280522	6.65326837	High Risk
GSM1672394	1512.9	0	8.35739805	14.0068695	6.12179917	Low Risk
GSM1672395	2200.8	0	9.56272515	14.2065718	6.38827014	Low Risk
GSM1672396	2002.2	0	8.0826813	13.7881575	6.00241723	Low Risk
GSM1672397	2184	0	8.11146956	14.3309869	6.19098106	Low Risk
GSM1672398	597	1	7.93438676	13.1382734	5.75788679	Low Risk
GSM1672399	938.4	1	10.2945518	14.3699449	6.56432413	High Risk
GSM1672400	1359.9	1	9.57247665	14.1146452	6.35875041	Low Risk
GSM1672401	2518.5	0	10.7365034	14.5541751	6.69961909	High Risk
GSM1672402	378.9	1	11.2106592	14.2593313	6.67800138	High Risk
GSM1672403	2336.7	0	9.57066215	13.7378177	6.2308486	Low Risk
GSM1672404	335.4	0	9.65380346	14.5509752	6.51991859	High Risk
GSM1672405	915.9	0	8.26212774	13.3748629	5.89207006	Low Risk
GSM1672406	2207.7	0	8.45333237	13.5278557	5.97542068	Low Risk
GSM1672407	350.4	1	10.1253747	14.1933713	6.4766225	High Risk
GSM1672408	2238.3	0	9.78576795	14.0710936	6.37919027	Low Risk
GSM1672409	958.2	1	7.97568177	13.1020413	5.75243035	Low Risk
GSM1672410	67.2	1	8.90081852	14.079018	6.23588036	Low Risk
GSM1672411	265.5	1	12.0122963	14.2028508	6.79112476	High Risk
GSM1672412	2766	0	8.8986951	14.2221307	6.28399131	Low Risk
GSM1672413	2702.1	0	7.71605805	14.4159794	6.15452901	Low Risk
GSM1672414	749.1	1	9.87758783	14.9415474	6.68909387	High Risk
GSM1672415	1261.2	1	9.75872124	13.9588427	6.33671753	Low Risk
GSM1672416	366	1	10.0332716	14.4811877	6.55888915	High Risk
GSM1672417	81	1	9.60540358	14.928014	6.63960792	High Risk
GSM1672418	2880	1	9.49629456	14.6576701	6.53006314	High Risk
GSM1672419	1983	1	9.61670138	14.1770769	6.38718714	Low Risk
GSM1672420	2058	1	9.70024896	14.5561468	6.52933209	High Risk
GSM1672421	1440	1	10.6381463	14.4599823	6.65149688	High Risk

id	OS.time	OS	RRM2	GAPDH	riskScore	risk
GSM1672422	480	1	11.5444236	15.0972503	7.01680238	High Risk
GSM1672423	2160	0	10.1858418	14.281959	6.51659579	High Risk
GSM1672424	1410	0	7.69855188	13.7799553	5.93626853	Low Risk
GSM1672425	1560	1	7.70020387	13.4775812	5.83415023	Low Risk
GSM1672426	1170	1	9.76286535	15.1226784	6.7315028	High Risk
GSM1672427	1980	0	7.80286528	14.3008459	6.12986305	Low Risk
GSM1672428	1320	0	7.52321692	13.8666994	5.93671644	Low Risk
GSM1672429	2100	0	6.5940379	14.1017705	5.86302692	Low Risk
GSM1672430	2250	0	8.8282698	14.3651878	6.32081542	Low Risk
GSM1672431	2760	0	7.23780269	14.4370368	6.08276001	Low Risk
GSM1672432	1950	0	10.5754069	14.8227202	6.76397796	High Risk
GSM1672433	1290	0	9.89204944	15.033281	6.72254276	High Risk
GSM1672434	2070	0	10.9978075	15.3116313	6.99921949	High Risk
GSM1672435	1200	1	10.4864932	14.7117261	6.71172439	High Risk
GSM1672436	630	1	10.4865635	14.9764701	6.80138444	High Risk
GSM1672437	870	1	10.2242911	15.0097105	6.76937237	High Risk
GSM1672438	780	1	10.3396273	14.6076251	6.65224439	High Risk
GSM1672439	2310	0	9.55627804	14.8174434	6.59406174	High Risk
GSM1672440	60	1	10.9590674	14.9486684	6.86992078	High Risk
GSM1672441	1980	0	8.49284661	14.3635599	6.26492818	Low Risk
GSM1672442	420	1	8.84899843	14.2285707	6.27797339	Low Risk
GSM1672443	990	0	6.8312439	14.3142009	5.97409345	Low Risk
GSM1672444	2550	0	10.6336038	15.1216145	6.8747914	High Risk
GSM1672445	1980	0	8.49388746	14.8541184	6.43121432	High Risk
GSM1672446	450	0	7.41449075	14.6910837	6.19793503	Low Risk
GSM1672447	2730	0	9.75191556	14.085655	6.37853636	Low Risk
GSM1672448	2040	1	7.679276	14.4240547	6.15119544	Low Risk
GSM1672449	990	0	9.64862722	14.6285082	6.5453191	High Risk
GSM1672450	720	1	9.38176356	14.7549762	6.54411862	High Risk
GSM1672451	1860	0	6.92709052	13.9388628	5.86280751	Low Risk
GSM1672452	3420	1	9.58284763	15.4515331	6.81316241	High Risk
GSM1672453	120	1	10.1330908	15.020993	6.75814721	High Risk
GSM1672454	600	1	9.92141401	14.8168687	6.65410493	High Risk
GSM1672455	1560	1	9.86119394	14.430446	6.51331859	High Risk
GSM1672456	2790	0	8.5640542	14.7598466	6.41086737	High Risk
GSM1672457	420	1	10.6667122	15.1903687	6.9035352	High Risk
GSM1672458	3000	0	8.67267284	14.3521745	6.29073941	Low Risk
GSM1672459	990	0	8.12343341	14.0739323	6.10591018	Low Risk

id	OS.time	OS	RRM2	GAPDH	riskScore	risk
GSM1672460	960	1	7.87725959	13.9777808	6.03273886	Low Risk
GSM1672461	1800	0	8.1324586	14.0403839	6.09603883	Low Risk
GSM1672462	1500	0	7.05440353	14.9081111	6.21202061	Low Risk
GSM1672463	450	1	11.0828875	13.8576798	6.52091394	High Risk
GSM1672464	1110	0	7.37261262	14.3928138	6.09002517	Low Risk
GSM1672465	1620	0	7.25362061	13.9520678	5.92114787	Low Risk
GSM1672466	930	0	6.76625061	14.3031172	5.95961809	Low Risk
GSM1672467	1140	0	10.1254135	15.1656608	6.8058685	High Risk
GSM1672468	3450	0	7.64939272	13.9799164	5.99587	Low Risk
GSM1672469	1080	0	7.78388877	13.8932541	5.98871246	Low Risk
GSM1672470	1440	1	8.49910043	13.314824	5.91083374	Low Risk
GSM1672471	570	1	7.87903792	14.210854	6.11195619	Low Risk
GSM1672472	2730	0	7.47859167	14.3668071	6.09870246	Low Risk
GSM1672473	6120	1	9.50817904	14.9205389	6.62103721	High Risk
GSM1672474	1500	0	6.50487936	14.1941643	5.87960478	Low Risk
GSM1672475	2400	0	10.3956948	14.7996004	6.7265013	High Risk
GSM1672476	1260	1	8.24603273	14.1929477	6.1664372	Low Risk
GSM1672477	1800	0	10.0648236	15.5182444	6.9152657	High Risk
GSM1672478	780	0	9.82729348	14.8782378	6.65935851	High Risk
GSM1672479	1860	1	10.5685832	14.5294306	6.6635376	High Risk
GSM1672480	660	0	9.04452527	14.2912786	6.33146451	Low Risk
GSM1672481	1890	0	7.5131354	14.1013438	6.01450925	Low Risk
GSM1672482	2040	0	8.35603814	13.8151331	6.05664844	Low Risk
GSM1672483	210	0	8.95931229	14.2621169	6.30753177	Low Risk
GSM1672484	1560	1	8.51899142	14.1726306	6.20458834	Low Risk
GSM1672485	1080	0	7.08180829	13.8020316	5.84199766	Low Risk
GSM1672486	450	1	10.0333955	14.2665816	6.48623903	High Risk
GSM1672487	600	1	10.355285	14.9112281	6.75763449	High Risk
GSM1672488	1230	0	9.7910147	14.2097503	6.42700822	High Risk
GSM1672489	750	1	5.57412573	14.5339867	5.84112666	Low Risk
GSM1672490	1260	0	9.29391761	14.4874444	6.43903392	High Risk
GSM1672491	1530	1	9.36633752	13.8317229	6.22893888	Low Risk
GSM1672492	1500	0	9.56151517	14.9726029	6.64746632	High Risk
GSM1672493	1710	1	8.98550612	13.7678327	6.14447705	Low Risk
GSM1672494	390	1	10.3992672	14.4011662	6.59217166	High Risk
GSM1672495	2340	1	7.10794936	14.3574283	6.03438036	Low Risk
GSM1672496	1440	0	6.78408514	14.2103365	5.93114264	Low Risk
GSM1672497	990	1	8.05469656	14.0994464	6.10321008	Low Risk

id	OS.time	OS	RRM2	GAPDH	riskScore	risk
GSM1672498	1110	0	6.87010575	13.7603665	5.79296356	Low Risk
GSM1672499	510	1	8.32912808	14.4756511	6.27587562	Low Risk
GSM1672500	720	1	10.202234	14.9617933	6.74950766	High Risk
GSM1672501	1320	0	5.87012302	14.1089818	5.74604194	Low Risk
GSM1672502	900	0	11.3133706	14.7817746	6.8718574	High Risk
GSM1672503	1500	0	10.1810031	14.282712	6.51605253	High Risk
GSM1672504	2640	0	9.29118452	13.9494346	6.25640052	Low Risk
GSM1672505	1260	0	7.10722754	13.831713	5.85624196	Low Risk
GSM1672506	1380	0	8.09642527	14.2087526	6.14710779	Low Risk
GSM1672507	1680	0	7.65099427	13.8307232	5.94561395	Low Risk
GSM1672508	1200	0	8.45317184	14.2762553	6.22881954	Low Risk
GSM1672509	1140	0	7.19219417	14.1910901	5.99195258	Low Risk
GSM1672510	1290	0	10.7257923	14.9722978	6.839438	High Risk
GSM1672511	1650	0	7.45863628	14.1924238	6.03636014	Low Risk
GSM1672512	510	1	10.8305786	14.8651291	6.82043521	High Risk
GSM1672513	1080	0	7.035096	13.5566857	5.75121157	Low Risk
GSM1672514	300	1	8.8278349	14.7321144	6.44499347	High Risk
GSM1672515	690	1	8.68615732	15.1796875	6.57317899	High Risk
GSM1672516	1860	0	8.79227298	14.4629048	6.34796612	Low Risk
GSM1672517	2550	0	9.65938944	14.6000465	6.53745679	High Risk
GSM1672518	1410	1	10.2494809	15.430629	6.91606069	High Risk
GSM1672519	1440	1	6.98792338	14.7473433	6.14661347	Low Risk
GSM1672520	1710	0	7.64853016	13.8822609	5.96265931	Low Risk
GSM1672521	1290	0	7.82952613	14.3859298	6.16307276	Low Risk
GSM1672522	1230	0	7.77867512	13.6567377	5.90776243	Low Risk
GSM1672523	1290	0	7.47487404	13.3263733	5.74577427	Low Risk
GSM1672524	960	0	7.75111554	15.2593093	6.44588351	High Risk
GSM1672525	1920	0	7.11644794	13.4498708	5.72846253	Low Risk
GSM1672526	3210	0	7.07650565	14.8385343	6.19210655	Low Risk
GSM1672527	750	1	8.14845116	14.8485154	6.37232912	Low Risk
GSM1672528	2880	0	7.12870582	15.0225369	6.26302573	Low Risk
GSM1672529	2940	0	8.86068972	15.4527464	6.69443625	High Risk
GSM1672530	1110	1	9.53587799	15.9259832	6.9660734	High Risk
GSM1672531	2700	0	9.29809236	14.3866035	6.40557559	Low Risk
GSM1672532	1200	0	9.23782181	14.785335	6.53065221	High Risk
GSM1672533	2130	1	8.12553746	14.1228603	6.12282545	Low Risk
GSM1672534	270	1	9.01792747	15.7710509	6.82816159	High Risk
GSM1672535	3300	0	8.40554145	14.5896628	6.32708881	Low Risk

id	OS.time	OS	RRM2	GAPDH	riskScore	risk
GSM1672536	3300	0	8.6421853	14.7582753	6.42322486	High Risk
GSM1672537	180	1	10.0540616	15.0784427	6.76456329	High Risk
GSM1672538	2850	1	9.09869273	15.163799	6.63585624	High Risk
GSM1672539	1140	1	9.98964995	15.9119116	7.03616882	High Risk
GSM1672540	270	1	10.3208231	15.3312564	6.89418043	High Risk
GSM1672541	2610	0	8.17586388	16.0970898	6.79964758	High Risk
GSM1672542	2640	0	6.50354433	14.6927299	6.04821033	Low Risk
GSM1672543	2220	0	9.413154	16.0305357	6.98123106	High Risk
GSM1672544	240	1	9.77583412	15.1333791	6.7372658	High Risk
GSM1672545	1770	0	9.51688386	14.7497699	6.56464695	High Risk
GSM1672546	1650	0	11.2564741	14.8787742	6.89531726	High Risk
GSM1672547	1080	0	10.5709467	15.0519184	6.84085394	High Risk
GSM1672548	450	1	7.25800062	14.8409303	6.2228598	Low Risk
GSM1672549	2550	0	9.77333705	14.9937168	6.68956099	High Risk
GSM1672550	270	1	9.25324949	15.9352479	6.92258441	High Risk
GSM1672551	330	1	9.61296246	15.8174359	6.94203369	High Risk
GSM1672552	2370	1	9.73575232	14.295898	6.44706298	High Risk
GSM1672553	3150	0	8.62844919	14.1547865	6.21660354	Low Risk
GSM1672554	2910	0	10.4580169	15.960407	7.1298587	High Risk
GSM1672555	120	1	8.94233562	15.6573464	6.77718794	High Risk
GSM1672556	1080	1	10.2064772	14.8007887	6.69568778	High Risk
GSM1672557	1320	1	8.74812239	14.6578878	6.40670818	Low Risk
GSM1672558	2220	1	9.53068334	15.7852481	6.91756027	High Risk
GSM1672559	390	1	9.14773153	15.1306349	6.63271621	High Risk
GSM1672560	2700	0	8.7671019	15.1749764	6.5849374	High Risk
GSM1672561	2550	0	9.31845017	14.6858164	6.51025449	High Risk
GSM1672562	2130	1	8.9930516	14.1580889	6.27787161	Low Risk
GSM1672563	1920	1	8.05353496	14.8591125	6.36025889	Low Risk
GSM1672564	1950	1	10.3461369	15.0831967	6.81435784	High Risk
GSM1672565	2040	0	8.52958282	14.8507094	6.43594874	High Risk
GSM1672566	1920	0	7.13448793	15.2407503	6.33787174	Low Risk
GSM1672567	1890	0	9.23824709	14.2067931	6.33481475	Low Risk
GSM1672568	390	1	7.94021374	16.2813523	6.82316707	High Risk
GSM1672569	2850	0	9.5579576	15.326591	6.76674794	High Risk
GSM1672570	2220	0	9.00279252	15.7417724	6.81575035	High Risk
GSM1672571	1710	1	6.56307574	14.8692902	6.11781883	Low Risk
GSM1672572	1680	1	8.37356992	14.6187984	6.33168033	Low Risk
GSM1672573	1440	0	9.68812265	14.8929364	6.64137631	High Risk

id	OS.time	OS	RRM2	GAPDH	riskScore	risk
GSM1672574	1470	0	6.55887373	14.1993963	5.89028409	Low Risk
GSM1672575	840	0	9.88331228	14.6890753	6.60454535	High Risk
GSM1672576	1080	0	8.61780904	14.8167987	6.43902079	High Risk
GSM1672577	1200	0	8.64424927	14.7158012	6.40918264	High Risk
GSM1672578	1110	0	9.05909565	15.0286011	6.58354262	High Risk
GSM1672579	1200	0	5.35053614	14.0593614	5.6435212	Low Risk
GSM1672580	690	1	9.16151991	15.0962663	6.62335292	High Risk
GSM1672581	2280	0	9.30010313	14.6977984	6.51128507	High Risk
GSM1672582	1020	1	8.48625984	15.0095269	6.48258085	High Risk
GSM1672583	1980	0	7.66250461	15.1915489	6.40831983	Low Risk
GSM1672584	870	1	8.78386586	15.4081041	6.66664545	High Risk
GSM1672585	210	1	10.4336376	14.9003338	6.76687153	High Risk
GSM1672586	1590	0	8.20568086	14.539904	6.27726758	Low Risk
GSM1672587	1890	0	7.66083737	15.0377657	6.35597024	Low Risk
GSM1672588	690	0	9.43685102	14.2977904	6.39839294	Low Risk
GSM1672589	1860	0	10.5473091	14.8328702	6.76277961	High Risk
GSM1672590	210	1	10.7805726	15.0290931	6.86770751	High Risk
GSM1672591	780	0	8.67700428	15.0302102	6.5210525	High Risk
GSM1672592	1200	0	9.9270431	15.2338181	6.79622228	High Risk
GSM1672593	900	0	10.1264206	15.4839921	6.91382896	High Risk
GSM1672594	1200	0	9.57049139	15.1187911	6.69844983	High Risk
GSM1672595	2520	0	8.33471128	14.023815	6.12379457	Low Risk
GSM1672596	540	1	9.99966183	14.6444309	6.60862235	High Risk
GSM1672597	750	1	8.95772021	14.2812273	6.31374037	Low Risk
GSM1672598	2400	0	9.24279064	14.3931558	6.39867103	Low Risk
GSM1672599	360	1	9.65592242	14.9471027	6.65440606	High Risk
GSM1672600	960	0	9.30074379	14.7668291	6.53476616	High Risk
GSM1672601	3150	0	8.94173723	14.5987446	6.41862226	High Risk
GSM1672602	2430	0	10.7772553	15.4737692	7.01773782	High Risk
GSM1672603	2250	0	8.1188788	14.2387779	6.1609793	Low Risk
GSM1672604	2910	0	7.64141597	13.9239681	5.97560867	Low Risk
GSM1672605	540	1	8.49510351	14.7408717	6.39306699	Low Risk
GSM1672606	960	1	9.26653358	14.7405766	6.52023268	High Risk
GSM1672607	300	1	10.3621849	15.3382698	6.90337895	High Risk
GSM1672608	1348.2	1	11.1806365	14.224258	6.66117178	High Risk
GSM1672609	1042.8	1	9.24501546	14.2855106	6.36258691	Low Risk
GSM1672610	595.2	1	10.2600492	13.8792865	6.39248386	Low Risk
GSM1672611	258.3	1	9.47518016	14.0184784	6.31013476	Low Risk

id	OS.time	OS	RRM2	GAPDH	riskScore	risk
GSM1672612	15.9	1	8.13248945	13.2778308	5.83782585	Low Risk
GSM1672613	1126.5	1	10.6909757	13.7902365	6.43342099	High Risk
GSM1672614	3197.4	0	10.1473194	14.6658688	6.64024134	High Risk
GSM1672615	3699	0	9.73255922	14.2806838	6.44138431	High Risk
GSM1672616	1704	1	9.30103656	14.1109889	6.31273186	Low Risk
GSM1672617	903.6	1	9.95961513	13.6665125	6.27086999	Low Risk
GSM1672618	399.9	1	7.26871509	13.5680274	5.79359311	Low Risk
GSM1672619	3179.7	1	7.73337468	13.2394061	5.75897096	Low Risk
GSM1672620	1332.6	1	10.2606256	14.1729822	6.49203107	High Risk
GSM1672621	625.8	1	10.167506	14.0838532	6.44648769	High Risk
GSM1672622	828.9	1	8.32676653	13.9053964	6.08238463	Low Risk
GSM1672623	2225.4	1	9.80985332	13.8296435	6.30140315	Low Risk
GSM1672624	2880	1	8.31639468	12.8703146	5.730171	Low Risk
GSM1672625	1239.9	1	10.0060736	13.7758983	6.31557496	Low Risk
GSM1672626	169.5	1	9.98844327	14.2723017	6.48076007	High Risk
GSM1672627	12.9	1	9.30292446	14.4656876	6.43315245	High Risk
GSM1672628	496.8	1	10.5289034	13.8937281	6.44172798	High Risk
GSM1672629	1836.3	1	8.29291976	13.9665505	6.097509	Low Risk
GSM1672630	3134.1	0	10.3875973	14.1976391	6.52132746	High Risk
GSM1672631	2118	1	9.10508088	14.4656429	6.40049836	Low Risk
GSM1672632	105.6	1	11.3229309	14.2497061	6.69326391	High Risk
GSM1672633	526.2	1	8.95532152	13.9941501	6.21613368	Low Risk
GSM1672634	826.8	1	10.3902657	14.6936067	6.68971376	High Risk
GSM1672635	491.7	1	10.6877956	14.395909	6.63799101	High Risk
GSM1672636	2331	0	9.2692693	14.0439586	6.28479308	Low Risk
GSM1672637	2824.8	0	8.6689417	12.8931365	5.79605999	Low Risk
GSM1672638	2202	0	9.82158746	14.1416761	6.40900041	Low Risk
GSM1672639	1357.2	1	9.94288393	14.6624125	6.60534447	High Risk
GSM1672640	0.9	1	9.30689561	13.9247568	6.25063597	Low Risk
GSM1672641	2271.9	1	7.65636765	13.9586796	5.98982941	Low Risk
GSM1672642	1171.8	1	9.35054587	13.8983935	6.24890989	Low Risk
GSM1672643	2328	1	7.88972297	14.3448368	6.15908863	Low Risk
GSM1672644	68.1	1	8.85070568	14.1767809	6.26071782	Low Risk
GSM1672645	2533.2	0	8.29141477	13.9866776	6.1040762	Low Risk
GSM1672646	1265.4	1	11.5058512	14.076348	6.66473791	High Risk
GSM1672647	2569.5	1	8.11798069	13.8969378	6.0450762	Low Risk
GSM1672648	2775.6	0	10.9209483	14.5709111	6.73571483	High Risk
GSM1672649	1180.8	1	10.1626305	14.4280591	6.56223942	High Risk

id	OS.time	OS	RRM2	GAPDH	riskScore	risk
GSM1672650	2475	0	8.95937315	13.9443643	6.19994348	Low Risk
GSM1672651	951	0	9.91856066	14.0140902	6.38179492	Low Risk
GSM1672652	1260.6	1	9.21518351	13.6385604	6.13859317	Low Risk
GSM1672653	173.4	1	8.29849795	13.8332953	6.05330597	Low Risk
GSM1672654	230.7	1	10.3852477	13.7148572	6.35745878	Low Risk
GSM1672655	1437	1	9.94223884	14.1455979	6.4302327	High Risk
GSM1672656	1765.2	0	9.39706064	13.5172388	6.12751581	Low Risk
GSM1672657	2446.2	0	10.0385624	13.7485807	6.3116844	Low Risk
GSM1672658	735.3	1	9.76907581	14.0106417	6.35596612	Low Risk
GSM1672659	210.9	1	9.67915106	13.9573197	6.32307485	Low Risk
GSM1672660	288.9	1	11.0836123	14.3025318	6.67167068	High Risk
GSM1672661	1206.3	1	7.51315909	13.5471476	5.82684956	Low Risk
GSM1672662	2000.7	0	8.95481281	14.1410135	6.26578107	Low Risk
GSM1672663	1229.1	1	9.90826651	14.6889933	6.6086344	High Risk
GSM1672664	2283.6	0	10.1226598	14.2130283	6.48283089	High Risk
GSM1672665	2179.2	1	8.91257203	14.2182605	6.28497008	Low Risk
GSM1672666	359.7	1	11.1375163	14.536484	6.75978502	High Risk
GSM1672667	187.2	1	9.89088592	14.7765924	6.63543012	High Risk
GSM1672668	656.4	1	9.18457537	14.6314215	6.4697493	High Risk
GSM1672669	2365.5	0	10.1820963	14.2699156	6.51189973	High Risk
GSM1672670	2161.5	0	9.41434064	14.1696745	6.35129634	Low Risk
GSM1672671	847.5	1	9.77579297	14.6045127	6.55817267	High Risk
GSM1672672	1601.7	0	11.0077141	13.766798	6.47773767	High Risk
GSM1672673	1170.9	0	7.6846785	13.7431619	5.92152071	Low Risk
GSM1672674	1566	1	8.07275967	13.9820756	6.06644553	Low Risk
GSM1672675	473.1	1	10.5109323	13.8114449	6.41090024	High Risk
GSM1672676	829.8	1	10.2693254	14.9744056	6.76484678	High Risk
GSM1672677	890.1	1	9.35732767	14.1724041	6.34281499	Low Risk
GSM1672678	360.6	1	11.1577827	14.0043522	6.58293633	High Risk
GSM1672679	4505.4	0	11.3511214	14.4554428	6.76758182	High Risk
GSM1672680	464.1	1	11.209965	14.5539772	6.77766075	High Risk
GSM1672681	742.2	1	9.76954696	13.9371347	6.33115267	Low Risk
GSM1672682	494.7	1	10.7475804	14.492379	6.68052091	High Risk
GSM1672683	268.2	1	9.5376921	14.5092554	6.486636	High Risk
GSM1672684	791.4	1	11.0778572	13.7750752	6.49211228	High Risk
GSM1672685	546	1	10.8411555	15.1559724	6.92066636	High Risk
GSM1672686	2386.2	1	8.94864173	13.6254691	6.09018781	Low Risk
GSM1672687	921	1	8.42137059	14.2388898	6.21092037	Low Risk

id	OS.time	OS	RRM2	GAPDH	riskScore	risk
GSM1672688	2427	1	10.6019193	13.8010212	6.42238098	High Risk
GSM1672689	351	1	9.44952836	14.2765461	6.39329056	Low Risk
GSM1672690	996	1	9.37065036	13.9820577	6.28055722	Low Risk
GSM1672691	1275	1	8.14443392	14.004238	6.08577461	Low Risk
GSM1672692	3672	1	9.92745206	14.5766615	6.57376136	High Risk
GSM1672693	906	1	11.0691745	14.8602674	6.85815095	High Risk
GSM1672694	2355	1	10.5672051	14.7412984	6.7350536	High Risk
GSM1672695	2196	1	7.23737251	13.9684688	5.92402112	Low Risk
GSM1672696	1956	1	7.89011225	14.3798589	6.17101214	Low Risk
GSM1672697	4374	0	10.8817334	14.5269149	6.71434727	High Risk
GSM1672698	2211	1	8.40474137	13.9855485	6.12238976	Low Risk
GSM1672699	99	1	10.6648828	14.5327455	6.68054702	High Risk
GSM1672700	585	1	7.5452039	14.3823876	6.11496763	Low Risk
GSM1672701	252	1	10.6553787	14.5855553	6.69686169	High Risk
GSM1672702	807	1	8.63972055	14.3812252	6.2951404	Low Risk
GSM1672703	3894	0	8.93223838	15.0026304	6.55382026	High Risk
GSM1672704	588	1	9.27003129	14.73064	6.51744493	High Risk
GSM1672705	3162	1	9.66569655	14.34953	6.45366666	High Risk
GSM1672706	4725	1	11.3163381	14.7522813	6.86235985	High Risk
GSM1672707	3615	1	10.2227104	14.4515009	6.58008894	High Risk
GSM1672708	1434	1	7.64816341	14.2788632	6.09689749	Low Risk
GSM1672709	3900	1	10.7879923	14.5181122	6.69590165	High Risk
GSM1672710	1569	0	9.69246207	14.1810358	6.40102622	Low Risk
GSM1672711	2082	0	7.65021167	14.2025601	6.07139739	Low Risk
GSM1672712	778.5	1	10.2595079	14.4389413	6.58190657	High Risk
GSM1672713	3654	0	11.3158798	14.5157922	6.78220358	High Risk
GSM1672714	978	1	9.85022904	14.9893723	6.700775	High Risk
GSM1672715	1206	1	10.8834452	14.4436041	6.6864187	High Risk
GSM1672716	435	1	10.0103043	14.8918691	6.69416637	High Risk
GSM1672717	276	1	9.13460087	14.5454894	6.43240624	High Risk
GSM1672718	570	1	10.7136786	14.2231042	6.58374537	High Risk
GSM1672719	3636	0	9.87495062	14.6648028	6.59494669	High Risk
GSM1672720	270	1	11.0864813	14.7062694	6.80885882	High Risk
GSM1672721	2928	0	10.2660955	14.7978692	6.70453462	High Risk
GSM1672722	5277	0	7.87098729	13.8605245	5.9919984	Low Risk
GSM1672723	1359	1	10.1969709	14.5111226	6.59603187	High Risk
